# A Transannular Polyene Tetracyclization for Rapid Construction of the Pimarane Framework

**DOI:** 10.1002/anie.202003127

**Published:** 2020-04-01

**Authors:** Julian M. Feilner, Klaus Wurst, Thomas Magauer

**Affiliations:** ^1^ Institute of Organic Chemistry and Center for Molecular Biosciences Leopold-Franzens-University Innsbruck Innrain 80–82 6020 Innsbruck Austria; ^2^ Institute of General, Inorganic and Theoretical Chemistry Leopold-Franzens-University Innsbruck Innrain 80–82 6020 Innsbruck Austria

**Keywords:** cyclization, natural products, terpenoids, pimarane, total synthesis

## Abstract

Polyene cyclizations are one of the most powerful and fascinating chemical transformations to rapidly generate molecular complexity. However, cyclizations employing heteroatom‐substituted polyenes are rare. Described here is the tetracyclization of a dual nucleophilic aryl enol ether involving an unprecedented transannular *endo*‐termination step. In this transformation, five stereocenters, two of which are quaternary, four carbon–carbon bonds, and four six‐membered rings are formed from a readily available cyclization precursor. The realization of this cyclization enabled short synthetic access to the tricyclic diterpenoid pimara‐15‐en‐3α‐8α‐diol.

## Graphical abstract

**The tricyclic** natural product pimara‐15‐en‐3*α*‐8*α*‐diol comprises three quaternary carbon centers and six stereocenters, four of which are adjacent. S*yn*‐pentane interactions of all four axial substituents generate considerable strain in the molecule. Reported here is a 13 step synthesis that features a powerful polyene tetracyclization involving an unprecedented transannular *endo*‐termination step.

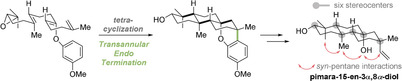

Since seminal studies by Stork and Eschenmoser in 1955, cationic polyene cyclizations have become one of the most powerful reactions to transform simple linear precursors into complex polycyclic architectures in one step.^1^ While for the initiation of the polyene cyclization a plethora of methods has been developed over the last decades,^2^ decoration along the polyene chain has mostly been limited to alkyl (methyl) groups. In scattered examples, Corey showed that silyl enol (transformation of **1** into **2**) and aryl ethers (conversion of **3** into **4**) readily participate in the cyclization, thereby acting as the terminating nucleophile (Scheme 1 A).^3^ Until then and as exemplified by the polyene **3**, termination of the cyclization involving aryl groups was only reported for the linear *endo*‐mode. Inspired by these examples, we hypothesized that installation of a central dual nucleophilic aryl enol ether unit should allow the unlocking of an unexplored *exo*‐termination pathway. The use of the aryl enol ether **5** corroborated this hypothesis to afford the meroterpenoid framework **6** as a single diastereomer in excellent yield (83 %).^4^ On the basis of these findings, we wanted to investigate a tetracyclization featuring an unprecedented transannular *endo*‐termination step. We envisioned that the realization of this transformation would enable access to polycyclic pimarane natural products such as **7** (Scheme 1 B).

**Scheme 1:**
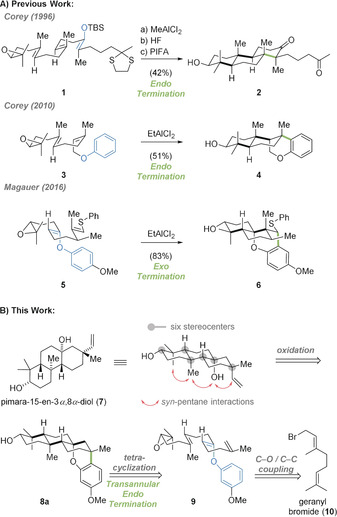
A) Cyclization of aryl/enol ethers. B) Retrosynthetic analysis of pimara‐15‐en‐3*α*‐8*α*‐diol (**7**) based on a transannular polyene tetracyclization/*endo*‐termination sequence. PIFA=phenyliodine bis(trifluoroacetate), TBS=*tert*‐butyldimethylsilyl.

The diterpenoid pimara‐15‐en‐3*α*‐8*α*‐diol (**7**) was first isolated from *G. gaudichaudianum* together with six other pimaranes in 2003.^5^ Whereas **7** was inactive in a brine shrimp assay, several related pimaranes were shown to display potent anticancer, antibiotic, and anti‐inflammatory activities.^6^ To date, the syntheses of only a few structurally simplified members—all of which lack the axially‐oriented tertiary alcohol—have been reported.^7^ A structure‐based retrosynthetic analysis of the tricyclic framework of **7** revealed *syn*‐pentane interactions of all four axial substituents which would be fully revealed upon oxidative cleavage of the bridging arene subunit of **8 a**. From a total of six stereocenters, five were envisioned to be directly generated from the tetracyclization of the polyene **9**. The crucial transannular *endo*‐termination step would install one of the two quaternary stereocenters formed in this process. Further C−C/C−O bond disconnections revealed commercially available geranyl bromide (**10**) as the starting point of the synthesis.

As depicted in Scheme 2, addition of lithiated 1‐(trimethylsilyl)propyne (**11**) to **10** afforded the dienyne **12** in 70 % yield. Conversion of **12** into the bromoacetylene **13** was accomplished in 74 % yield by subjecting a solution of **12** in acetone to *N*‐bromosuccinimide (NBS) and silver nitrate (AgNO_3_).^8^ Exposure of **13** to a suspension of 3‐methoxyphenol and Cs_2_CO_3_ in *N*,*N*‐dimethylformamide (DMF) at elevated temperatures (80 °C) for three days provided (*Z*)‐aryl enol ether **14** as a single isomer (44 % yield).^4^ The enol ether was found to be configurationally and chemically stable upon purification by column chromatography on silica gel. To further improve the yield, a broad temperature range (60 °C to 110 °C) as well as different bases [NaH; Na_2_CO_3_; K_3_PO_4_; 1,8‐diazabicyclo[5.4.0]undec‐7‐ene (DBU)] were investigated. However, the yield and purity of **14** were found to be lower for all reaction conditions examined. Regioselective asymmetric dihydroxylation of the terminal double bond was achieved by employing the Corey–Noe–Lin ligand **15** to afford the diol **16** in 73 % yield and 94 % *ee*.^9^ Mesylation of the secondary alcohol in **16** was followed by intramolecular nucleophilic substitution in the presence of K_2_CO_3_ to deliver the epoxide **17** in 80 % yield. For the crucial four‐carbon elongation, we resorted to a C(sp^2^)–C(sp^3^) Suzuki cross‐coupling reaction. For this purpose, **18** was first treated with *t*‐BuLi in presence of *B*‐methoxy‐9‐BBN. In the presence of a second‐generation SPhos precatalyst (5 mol %) and SPhos (5 mol %), the resulting boronate underwent efficient coupling with **17** to afford the cyclization precursor **9** in excellent yield (84 %).^4^, ^10^

**Scheme 2:**
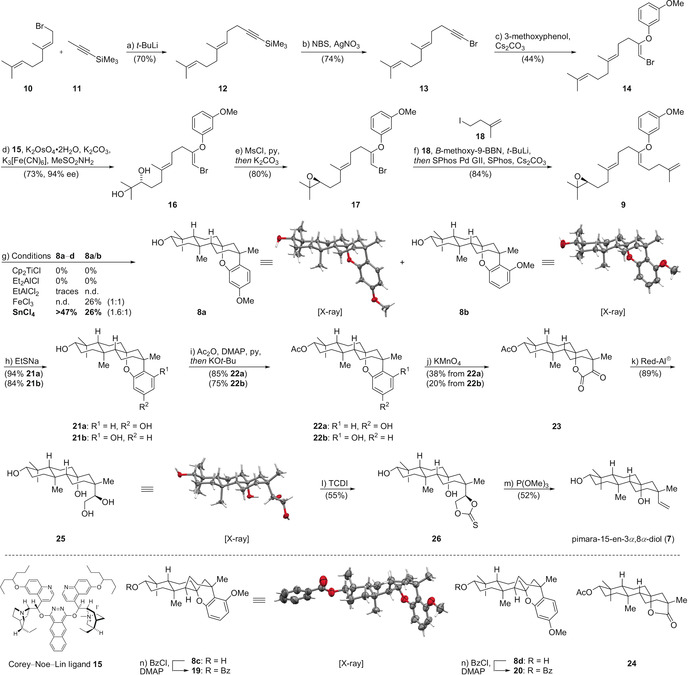
Synthesis of pimara‐15‐en‐3*α*‐8*α*‐diol (**7**). Reagents and conditions: a) *t*‐BuLi, THF, −20 °C to −5 °C, 70 %; b) NBS, AgNO_3_, acetone, 0 °C, 74 %; c) 3‐methoxyphenol, Cs_2_CO_3_, DMF, 80 °C, 44 %; d) Corey–Noe–Lin ligand **15**, MeSO_2_NH_2_, K_2_OsO_4_⋅2 H_2_O, K_2_CO_3_, K_3_[Fe(CN)_6_], *t*‐BuOH, H_2_O, 0 °C, 73 %, 94 % *ee*; e) MsCl, py, CH_2_Cl_2_, 22 °C, *then* K_2_CO_3_, MeOH, 22 °C, 80 %; f) **18**, *t*‐BuLi, *B*‐methoxy‐9‐BBN, THF, −78 °C to 22 °C, then SPhos Pd GII, SPhos, Cs_2_CO_3_, DMF, H_2_O, 40 °C, 84 %; g) SnCl_4_, CH_2_Cl_2_, −78 °C, 16 % **8 a**, 10 % **8 b**; h) EtSH, NaH, DMF, 120 °C, 94 % **21 a**, 84 % **21 b**; i) Ac_2_O, DMAP, pyridine, CH_2_Cl_2_, 22 °C, then KO*t*‐Bu, THF, *t*‐BuOH, 22 °C, 85 % **22 a**, 75 % **22 b**; j) KMnO_4_, ethyl acetate, H_2_O, 70 °C, 38 % from **22 a**, 20 % from **22 b**; k) Red‐Al, toluene, 22 °C, *then* 80 °C, 89 %; l) TCDI, DMF, 60 °C, 55 %; m) P(OMe)_3_, 110 °C, 52 %; n) BzCl, DMAP, py, 22 °C, 9 % **19** over 2 steps, 12 % **20** over 2 steps. X‐ray crystal structures are shown for **8 a**, **8 b**, **25**, and **19**.^19^ Ac=acetyl, *B*‐methoxy‐9‐BBN=9‐methoxy‐9‐borabicyclo[3.3.1]nonane, Bz=benzoyl, Cp=cyclopentadienyl, DMAP=4‐(*N*,*N*‐dimethylamino)pyridine, DMF=*N*,*N*‐dimethylformamide, *ee*=enantiomeric excess, MsCl=methanesulfonyl chloride, NBS=*N*‐bromosuccinimide, n.d.=not determined, py=pyridine, Red‐Al=sodium bis(2‐methoxyethoxy)aluminium hydride, SPhos=2‐dicyclohexylphosphino‐2′,6′‐dimethoxybiphenyl, SPhos Pd GII=chloro(2‐dicyclohexylphosphino‐2′,6′‐dimethoxy‐1,1′‐biphenyl)[2‐(2′‐amino‐1,1′‐biphenyl)]palladium(II), TCDI=1,1′‐thiocarbonyldiimidazole, THF=tetrahydrofuran.

For the promotion of the key cyclization, a panel of reaction conditions was screened. While exposure to the Nugent–RajanBabu reagent (titanocene dichloride, Mn, THF, 22 °C)^11^ led to the formation of a complex mixture of decomposition products, diethylaluminum chloride (Et_2_AlCl, CH_2_Cl_2_, −78 °C) did not lead to any transformation and **9** was left unchanged (Scheme 2). With the stronger Lewis acid ethylaluminum dichloride (EtAlCl_2_, CH_2_Cl_2_, −94 °C to −78 °C) promotion of the cyclization was observed, however, only traces of the products **8 a**/**b**/**c**/**d** were formed together with a complex mixture of inseparable byproducts.^12^ We finally found that iron(III) chloride (FeCl_3_, CH_2_Cl_2_, −50 °C to −20 °C) and tin(IV) chloride (SnCl_4_, CH_2_Cl_2_, −78 °C) led to rapid initiation of the transannular polyene tetracyclization and also promoted the challenging *endo*‐termination step to afford a mixture of products (**8 a**–**d**).^13^ Employing the latter reaction conditions, a mixture of four fully cyclized products was obtained in more than 47 % combined yield.^14^ The desired regioisomers **8 a** and **8 b**, which only differ in the position of the methoxy group (inconsequential), were isolated after purification by HPLC in 16 and 10 % yield, respectively. Their structures were validated by single‐crystal X‐ray analysis. After benzoylation of the remaining product fractions containing **8 c**/**d**, we also identified the *cis*‐decalin **19** (9 % over two steps), whose boat conformation was revealed by single‐crystal X‐ray analysis. The formation of **19** accounts for a stepwise mechanism involving a boat‐type transition state for the second ring closure. We can exclude isomerization of the enol ether prior to cyclization, as a model system lacking the epoxide (see the Supporting Information for details) did not undergo isomerization upon treatment with tin(IV) chloride. Detailed 2D NMR studies support the constitution shown for **20** (12 % over two steps). However, we are currently unable to unambiguously confirm the stereochemistry of **20** as NOE experiments were inconclusive and crystals for further structure analysis were not obtained.

To further improve the overall yield and diastereoselectivity of the tetracyclization, we turned our attention to the substitution pattern of the arene appendage. The highly modular synthesis allowed us to investigate the cyclization of several related aryl enol ethers (phenyl, 4‐methoxyphenyl, 3,5‐dimethoxyphenyl, 2,3‐dimethoxyphenyl). Surprisingly, all of these derivatives proved to be inferior to **9** (3‐methoxyphenyl) leading to lower yields and unidentified product mixtures.

Next, we investigated oxidative unmasking of the keto‐lactone motif. Initial efforts to directly oxidize the methoxybenzene ring of **8 a**/**b** with either ruthenium tetroxide (RuCl_3_⋅2 H_2_O, NaIO_4_), ozone (O_3_), or potassium permanganate (KMnO_4_) failed.^15^ Therefore, we decided to facilitate oxidation by first demethylating **8 a**/**b**. Treatment with sodium ethanethiolate (EtSNa, DMF, 120 °C) delivered the phenols **21 a**/**b** in excellent yields (94 % and 84 %). Selective protection of the secondary alcohol in presence of the phenol was necessary to prevent overoxidation in the following step. Transesterification with ethyl acetate catalyzed by *p*‐toluenesulfonic acid proved to be selective for the secondary alcohol, but low yielding because of competing substrate decomposition. We therefore developed a one‐pot procedure involving double acetylation (Ac_2_O, DMAP, pyridine) and subsequent selective deprotection of the phenol with potassium *tert*‐butoxide (KO*t*‐Bu) to provide **22 a** and **22 b** in high yields (85 % and 75 %).^16^

For the oxidative cleavage of the phenol, KMnO_4_ turned out to be the oxidant of choice.15c, 15d Under optimized reaction conditions, a solution of **22 a** in a biphasic mixture of water and ethyl acetate in the presence of excess potassium permanganate (20 equiv) was heated at 70 °C for 24 hours to afford the keto‐lactone **23** in 38 % yield. The lactone **24** was isolated as a side‐product (7 %).^17^ We found that a low concentration (24 mm) of **22 a** was crucial to suppress lactone formation and to favor formation of the keto‐lactone. To prevent stalling of the reaction, it was necessary to continuously add an aqueous solution of potassium permanganate (KMnO_4_) to the reaction mixture by syringe pump. The other regioisomer **22 b** was even more reluctant to oxidation and required extended reaction times (48 h) to reach full conversion under the reaction conditions. **23** was obtained in 20 % yield along with **24** (7 %). Deprotection and reduction of **23** was achieved by treatment with Red‐Al, affording the tetraol **25** in excellent yield (89 %). For the introduction of the missing vinyl unit and completion of the synthesis, we resorted to the Corey–Winter protocol.^18^ For the formation of the thiocarbonate **26** we initially treated a solution of **25** in chloroform with thiophosgene (CSCl_2_) and 4‐(*N*,*N*‐dimethylamino)pyridine (DMAP). However, this procedure turned out to be not reproducible and led to variable yields (0–58 %). We attribute this observation to the poor solubility of the substrate in chlorinated solvents. Performing the reaction in *N*,*N*‐dimethylformamide (60 °C) in the presence of 1,1′‐thiocarbonyldiimidazole (TCDI) reproducibly gave **26** in 55 % yield. The final elimination was induced upon heating a solution of **26** in trimethyl phosphite to 110 °C affording pimara‐15‐en‐3*α*‐8*α*‐diol (**7**) in 52 % yield. The NMR and mass‐spectroscopic data obtained for synthetic **7** were in full agreement with those reported for the natural enantiomer in the literature.

In conclusion, we accomplished a powerful polyene tetracyclization involving an unprecedented transannular *endo*‐termination step. The key cyclization forges the carboskeleton of the natural product by forming four C−C bonds and setting five stereocenters, two of which are quaternary. The highly modular, asymmetric synthesis provides the cyclization precursor in only six steps and also allows rapid structural modifications of the substitution pattern along the polyene backbone. The realization of this approach enabled the first total synthesis of the diterpenoid pimara‐15‐en‐3*α*‐8*α*‐diol in 13 steps from commercially available geranyl bromide (**10**).

## Conflict of interest

The authors declare no conflict of interest.

## Supporting information

As a service to our authors and readers, this journal provides supporting information supplied by the authors. Such materials are peer reviewed and may be re‐organized for online delivery, but are not copy‐edited or typeset. Technical support issues arising from supporting information (other than missing files) should be addressed to the authors.

Supplementary

## References

[1a] G. Stork , A. W. Burgstahler , J. Am. Chem. Soc. 1955, 77, 5068–5077;

[1b] A. Eschenmoser , L. Ruzicka , O. Jeger , D. Arigoni , Helv. Chim. Acta 1955, 38, 1890–1904.

[2a] C. N. Ungarean , E. H. Southgate , D. Sarlah , Org. Biomol. Chem. 2016, 14, 5454–5467; 27143099 10.1039/c6ob00375c

[2b] A. Barrett , T.-K. Ma , T. Mies , Synthesis 2019, 51, 67–82;

[2c] K. Hung , X. Hu , T. J. Maimone , Nat. Prod. Rep. 2018, 35, 174–202; 29417970 10.1039/c7np00065k PMC5858714

[2d] R. A. Yoder , J. N. Johnston , Chem. Rev. 2005, 105, 4730–4756. 16351060 10.1021/cr040623l PMC2575671

[3a] E. J. Corey , S. Lin , J. Am. Chem. Soc. 1996, 118, 8765–8766;

[3b] E. J. Corey , G. Luo , L. S. Lin , J. Am. Chem. Soc. 1997, 119, 9927–9928;

[3c] R. A. Shenvi , E. J. Corey , Org. Lett. 2010, 12, 3548–3551. 20670018 10.1021/ol101410g PMC2914341

[4a] K. Speck , R. Wildermuth , T. Magauer , Angew. Chem. Int. Ed. 2016, 55, 14131–14135; 10.1002/anie.201608040 27730742

[4b] K. Speck , T. Magauer , Chem. Eur. J. 2017, 23, 1157–1165. 27859768 10.1002/chem.201605029

[5] T. Meragelman , G. L. Silva , E. Mongelli , R. R. Gil , Phytochemistry 2003, 62, 569–572. 12560027 10.1016/s0031-9422(02)00611-8

[6a] J.-L. Chen , Z.-M. Zhao , X. Xue , G.-H. Tang , L.-P. Zhu , D.-P. Yang , L. Jiang , RSC Adv. 2014, 4, 14447–14456;

[6b] T. O. Kwon , S.-I. Jeong , J. W. Kwon , Y. C. Kim , S. Il Jang , Arch. Pharmacal Res. 2008, 31, 1172–1178; 10.1007/s12272-001-1285-3 18806961

[6c] S.-I. Jeong , W.-S. Han , Y.-H. Yun , K.-J. Kim , Phytother. Res. 2006, 20, 511–514; 16619343 10.1002/ptr.1894

[6d] J. Wang , K. Xie , H. Duan , Y. Wang , H. Ma , H. Fu , Bioorg. Med. Chem. Lett. 2017, 27, 1815–1819. 28302401 10.1016/j.bmcl.2017.02.051

[7a] R. F. Church , R. E. Ireland , J. A. Marshall , J. Org. Chem. 1962, 27, 1118–1125;

[7b] R. E. Ireland , P. W. Schiess , J. Org. Chem. 1963, 28, 6–16;

[7c] R. F. Church , R. E. Ireland , J. Org. Chem. 1963, 28, 17–23;

[7d] E. E. Van Tamelen , S. A. Marson , J. Am. Chem. Soc. 1975, 97, 5614–5616;

[7e] K. Mori , M. Waku , Tetrahedron 1985, 41, 5653–5660;

[7f] B. J. M. Jansen , G. C. Schepers , A. de Groot , Tetrahedron 1989, 45, 2773–2776;

[7g] A. Yajima , K. Toda , K. Okada , H. Yamane , M. Yamamoto , M. Hasegawa , R. Katsuta , T. Nukada , Tetrahedron Lett. 2011, 52, 3212–3215;

[7h] M. Zhao , J. Cheng , B. Guo , J. Duan , C.-T. Che , J. Agric. Food Chem. 2018, 66, 7859–7872. 29996047 10.1021/acs.jafc.8b02602 PMC6592423

[8] T. Nishikawa , S. Shibuya , S. Hosokawa , M. Isobe , Synlett 1994, 7, 485–486.

[9a] E. J. Corey , M. C. Noe , S. Lin , Tetrahedron Lett. 1995, 36, 8741–8744;

[9b] The enantiomeric excess of **16** was determined by Mosher ester analysis: J. A. Dale , H. S. Mosher , J. Am. Chem. Soc. 1973, 95, 512–519.

[10a] N. Miyaura , K. Yamada , A. Suzuki , Tetrahedron Lett. 1979, 20, 3437–3440.

[11a] W. A. Nugent , T. V. RajanBabu , J. Am. Chem. Soc. 1988, 110, 8561–8562;

[11b] T. V. RajanBabu , W. A. Nugent , J. Am. Chem. Soc. 1994, 116, 986–997.

[12a] G. Hilt , F. Pünner , J. Möbus , V. Naseri , M. A. Bohn , Eur. J. Org. Chem. 2011, 5962–5966;

[12b] E. J. Corey , M. Sodeoka , Tetrahedron Lett. 1991, 32, 7005–7008.

[13] S. E. Sen , S. L. Roach , S. M. Smith , Y. Zhang , Tetrahedron Lett. 1998, 39, 3969–3972.

[14] Due to inseparable impurities, the yields of **8 c** and **8 d** could only be determined over two steps after benzoylation. Therefore, the overall yield of **8 a**–**d** was estimated to be >47 %.

[15a] P. H. J. Carlsen , T. Katsuki , V. S. Martin , K. B. Sharpless , J. Org. Chem. 1981, 46, 3936–3938;

[15b] H. Klein , A. Steinmetz , Tetrahedron Lett. 1975, 16, 4249–4250;

[15c] O. Doebner , Ber. Dtsch. Chem. Ges. 1891, 24, 1753–1757;

[15d] R. Anschütz , G. Rauff , Justus Liebigs Ann. Chem. 1903, 327, 201–210.

[16] K. Iwasaki , M. Nakatani , M. Inoue , T. Katoh , Tetrahedron 2003, 59, 8763–8773.

[17] Efforts to convert the lactone **24** into **7** by reduction to the corresponding lactol and subsequent methylenation were unsuccessful.

[18] E. J. Corey , R. A. E. Winter , J. Am. Chem. Soc. 1963, 85, 2677–2678.

[19] CCDC 1987621 (**8 a**), 1987622 (**8 b**), 1987623 (**19**), and 1987624 (**25**) contain the supplementary crystallographic data for this paper. These data are provided free of charge by The Cambridge Crystallographic Data Centre.

