# Supplementary material for: A Transannular Polyene Tetracyclization for Rapid Construction of the Pimarane Framework
Source: Angew Chem Int Ed Engl. 2020 Apr 1;59(30):12436–9. doi: 10.1002/anie.202003127 (PMC7383491; doi:10.1002/anie.202003127)
Supplement: Supplementary file 1 — Supplementary [file ANIE-59-12436-s001.pdf]

Angewandte Chemie
Eine Zeitschrift der Gesellschaft Deutscher Chemiker

Supporting Information

A Transannular Polyene Tetracyclization for Rapid Construction of the Pimarane Framework

*Julian M. Feilner, Klaus Wurst, and Thomas Magauer**

anie_202003127_sm_miscellaneous_information.pdf

Content

1 General experimental details ... 3
2 Experimental part ... 5
2.1 Synthesis of pimara-15-en-3*α*-8*α*-diol (**7**) ... 5
2.1.1 (*E*)-(6,10-dimethylundeca-5,9-dien-1-yn-1-yl)trimethylsilane (**12**) ... 5
2.1.2 (*E*)-1-bromo-6,10-dimethylundeca-5,9-dien-1-yne (**13**) ... 6
2.1.3 (*Z*)-aryl enol ether **14** ... 7
2.1.4 Dienediol **16** ... 8
2.1.5 Epoxide fragment **17** ... 11
2.1.6 Cyclization precursor **9** ... 12
2.1.7 Pentacycle **8a** and **8b** ... 14
2.1.8 Phenol **21a** ... 19
2.1.9 Phenol **21b** ... 20
2.1.10 Protected pentacycle **22a** ... 21
2.1.11 Protected pentacycle **22b** ... 22
2.1.12 Ketolactone **23** ... 23
2.1.13 Tetraol **25** ... 25
2.1.14 Thiocarbonate **26** ... 26
2.1.15 Pimara-15-en-3*α*-8*α*-diol (**7**) ... 27
2.2 Synthesis of other cyclization precursors ... 28
2.2.1 (*Z*)-aryl enol ether **S6** ... 30
2.2.2 Dienediol **S7** ... 31
2.2.3 Epoxide fragment **S8** ... 32
2.2.4 Cyclization precursor **S9** ... 33
2.2.5 (*Z*)-aryl enol ether **S11** ... 34
2.2.6 Dienediol **S12** ... 35
2.2.7 Epoxide fragment **S13** ... 36
2.2.8 Cyclization precursor **S14** ... 37
2.2.9 (*Z*)-aryl enol ether **S16** ... 38
2.2.10 Dienediol **S17** ... 39
2.2.11 Epoxide fragment **S18** ... 40
2.2.12 Cyclization precursor **S19** ... 41
2.2.13 (*Z*)-aryl enol ether **S21** ... 42
2.2.14 Dienediol **S22** ... 43
2.2.15 Epoxide fragment **S23** ... 44
2.2.16 Cyclization precursor **S24** ... 45
2.3 Isomerization experiment ... 46

3 NMR comparison data for pimara-15-en-3*α*-8*α*-diol (**7**) 48
4 NMR spectra 49
5 X–Ray data 95
5.1 Minor regioisomer **8b** 95
5.2 Major regioisomer **8a**....................96
5.3 Undesired diastereomer **19** 97
5.4 Tetraol **25**....................98
6 References....................99

1 General experimental details

All reactions were carried out with magnetic stirring and, if moisture or air sensitive, under nitrogen or argon atmosphere using standard Schlenk techniques in oven-dried glassware (100 °C oven temperature). If required glassware was further dried under vacuum with a heat-gun at 650 °C. External bath thermometers were used to record all reaction temperatures. Low temperature reactions were carried out in a Dewar vessel filled with acetone and dry ice (–78 °C) or equipped with an electronically regulated cryostat in acetone (between –78 °C and 0 °C) or with distilled water and ice (0 °C). High temperature reactions were conducted in reaction vessels equipped with a reflux condenser or in a pressure tube using a heated silicon oil bath or a metal block. Tetrahydrofuran (THF) was dried over molecular sieve (4Å) prior to use. All other solvents were purchased from Acros Organics as 'extra dry' reagents. If required solvents were degassed by bubbling argon through the solvent with a balloon under sonication. All other reagents with a purity > 95% were obtained from commercial sources (Sigma Aldrich, Acros, Alfa Aesar and others) and used without further purification unless otherwise stated.

Flash column chromatography (FCC) was carried out with Merck silica gel 60 (0.040–0.063 mm). Analytical thin layer chromatography (TLC) was carried out using Merck silica gel 60 F254 aluminum foils and visualized under UV light at 254 nm. Staining was performed with ceric ammonium molybdate (CAM) or by staining with an aqueous potassium permanganate solution and subsequent heating.

High pressure liquid chromatography (HPLC) was carried out on normal-phase Varian Dynamax columns. For semipreparative separations a 250 x 21.4 mm Microsorb 60–8 Si column and for preparative separations a 250 x 41.4 mm Microsorb 60-8 Si column was used.

NMR spectra (^{1}H NMR and ^{13}C NMR) were recorded in deuterated chloroform (chloroform-*d*), deuterated benzene (benzene-d_6), deuterated dichloromethane (dichloromethane-d_2) or deuterated pyridine (pyridine-d_5) on a Bruker Avance Neo 400 MHz spectrometer, or a Bruker Avance II 600 MHz spectrometer and are reported as follows: chemical shift δ in ppm (multiplicity, coupling constant *J* in Hz, number of protons) for ^{1}H NMR spectra and chemical shift δ in ppm for ^{13}C NMR spectra. Multiplicities are abbreviated as follows: s = singlet, d = doublet, t = triplet, q = quartet, p = quintet, br = broad, m = multiplet, or combinations thereof. For ^{1}H NMR the residual protic solvent peak served as internal reference (chloroform-*d*: 7.26 ppm, benzene-d_6: 7.16 ppm, dichloromethane-d_2: 5.32 ppm, pyridine-d_5: 8.74 ppm for the signal with the highest shift). For ^{13}C NMR the central carbon resonance of chloroform-*d* (77.16 ppm or 77.00 ppm for comparison of synthetic and isolated natural products), benzene-d_6 (128.06 ppm), dichloromethane-d_2 (54.00 ppm) or pyridine-d_5 (150.35 ppm for the signal with the highest shift) served as internal reference. NMR spectra were assigned using information ascertained from COSY, HMBC, HSQC and NOESY experiments.

High resolution mass spectra (HRMS) were recorded on a Thermo Scientific™ LTQ Orbitrap XL™ Hybrid Ion Trap-Orbitrap Mass Spectrometer at the Institute of Organic Chemistry and Center for Molecular Biosciences, University of Innsbruck.

Infrared spectra (IR) were recorded from 4000 cm^{-1} to 450 cm^{-1} on a Bruker™ ALPHA FT-IR Spectrometer from Bruker. Samples were prepared as a neat film or a film by evaporation of a solution in Chloroform-*d*, Benzene-d_6 or ethyl acetate. IR data in frequency of absorption (cm^{-1}) is reported as follows: *w* = weak, *m* = medium, *s* = strong, *br* = broad or combinations thereof.

Melting points were measured with a SRS MPA120 EZ-Melt Melting Point Apparatus in open glass capillaries and are uncorrected.

Optical rotation values were recorded on a Schmidt+Haensch UniPol L1000 Peltier polarimeter. The specific rotation is calculated as follows: $[\alpha]_{\lambda}^{T} = \frac{\propto \times 100}{c \times d}$. Thereby, the wavelength λ is reported in nm and the measuring temperature in °C. *α* represents the recorded optical rotation, *c* the concentration of the analyte in 10 mg/mL and *d* the length of the cuvette in dm. Thus, the specific rotation is given in $10^{-1}\cdot deg\cdot cm^{2}\ g^{-1}$. Use of the sodium *D* line (λ = 589 nm) is indicated by *D* instead of the wavelength in nm. The sample concentration as well as the solvent is reported in the relevant section of the experimental part.

X-Ray diffraction analysis was carried out by Dr. Klaus Wurst at the Institute of Inorganic and Theoretical Chemistry and Center for Molecular Biosciences, University of Innsbruck. The data collections were performed on a Bruker D8 Quest diffractometer (Photon 100 detector) equipped with a microfocus source generator (Incoatec GmbH, Geesthacht, Germany) combined with multi-layer optics (monochromatized Mo *Kα* radiation, *λ* = 71.073 pm). The Bruker Apex III software was applied for the integration, scaling and multi-scan absorption correction of the data. The structure was solved with SHELXS[1] (version 2013/1). Structure refinement (full-matrix least-squares against F^2) with SHELXL[2] (version 2014/7). All non-hydrogen atoms were refined anisotropically. The hydrogen atoms were placed in ideal geometry riding on their parent atoms. Relevant details of the data collection and evaluation are listed in chapter 5. Supplementary crystallographic data for **8a**, **8b**, **19** and **25** may be obtained from the Cambridge Crystallographic Data Centre CCDC deposition service via www.ccdc.cam.ac.uk/structures on quoting the deposition number CCDC 1987621- 1987624. Plotting of thermal ellipsoids in this document and in the main text was carried out using MERCURY for Windows at 50% probability level.

All yields are isolated, unless otherwise specified.

2 Experimental part

2.1 Synthesis of pimara-15-en-3*α*-8*α*-diol (**7**)

2.1.1 (*E*)-(6,10-dimethylundeca-5,9-dien-1-yn-1-yl)trimethylsilane (**12**)

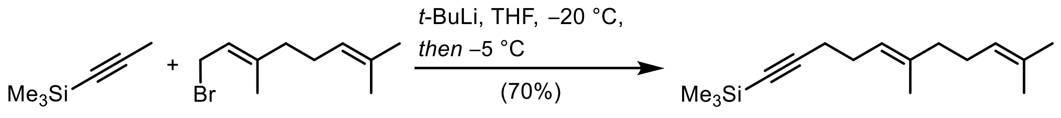

11 **10** **12**

To a solution of silane **11** (49.6 mL, 335 mmol, 2.00 equiv) in dry tetrahydrofuran (145 mL) was added *tert*-butyllithium (1.70 M in pentane, 187 mL, 1.90 equiv) at −20 °C. After 75 min, geranyl bromide (**10**) (35.4 mL, 167 mmol, 1 equiv) was added dropwise via syringe pump (100 mL/h). 30 min after the addition of **10** was complete, the reaction mixture was slowly allowed to warm to −5 °C over 80 min. After 15 min at –5 °C, the wine-red solution was poured into a 1:1 mixture of ice and water (800 mL). The mixture was extracted with pentane (3 x 500 mL), the combined organic layers were washed with saturated aqueous solution of sodium chloride (500 mL) and the washed solution was dried over magnesium sulfate. The dried solution was filtered, and the filtrate was concentrated. Purification of the residue by flash column chromatography on silica gel (0% grading to 2% diethyl ether in pentane) afforded the title compound **12** (29.1 g, 117 mmol, 70%) as a colorless liquid.[3]

TLC (pentane): R_f: 0.33.

^{1}H NMR (400 MHz, Chloroform-*d*) δ 5.27 – 5.13 (m, 1H), 5.13 – 5.01 (m, 1H), 2.28 – 2.15 (m, 4H), 2.12 – 2.03 (m, 2H), 2.03 – 1.95 (m, 2H), 1.68 (d, *J* = 0.8 Hz, 3H), 1.62 (d, *J* = 1.3 Hz, 3H), 1.60 (s, 3H), 0.14 (s, 9H).

^{13}C NMR (101 MHz, Chloroform-*d*) δ 136.7, 131.5, 124.4, 122.7, 107.6, 84.4, 39.8, 27.5, 26.8, 25.8, 20.5, 17.8, 16.3, 0.3.

IR (ATR, neat) $\tilde{\nu}_{max}$: 2962 (w), 2916 (w), 2856 (w), 2175 (w), 1445 (w), 1377 (w), 1248 (m), 1042 (w), 897 (w), 837 (s), 759 (m), 697 (w), 639 (w), 447 (w) cm^{-1}.

HRMS (ESI): calcd for $C_{16}H_{29}Si^+$ $[M+H]^+$: 249.2033; found: 249.2025.

2.1.2 (*E*)-1-bromo-6,10-dimethylundeca-5,9-dien-1-yne (**13**)

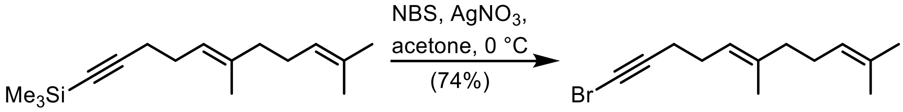

12 **13**

To a solution of silane **12** (22.4 g, 90.1 mmol, 1 equiv) in dry acetone (176 mL) was added $AgNO_3$ (7.66 g, 45.1 mmol, 0.500 equiv) and *N*-bromosuccinimide (19.3 g, 108 mmol, 1.20 equiv) successively at 0 °C.[1] After 6 h, water (500 mL) was added and the mixture was extracted with pentane (1000 mL, 2 x 250 mL). The combined organic layers were washed with saturated aqueous solution of sodium chloride (400 mL), the washed solution was dried over magnesium sulfate and the dried solution was filtered. The filtrate was concentrated and the residue was purified by flash column chromatography on silica gel (pentane) to yield the title compound **13** (17.0 g, 66.6 mmol, 74%) as a colorless oil.[4]

TLC (pentane): R_f: 0.74.

^{1}H NMR ^{1}H NMR (400 MHz, Chloroform-*d*) δ 5.22 – 5.00 (m, 2H), 2.25 – 2.18 (m, 4H), 2.11 – 1.97 (m, 4H), 1.69 (s, 3H), 1.61 (d, J = 4.2 Hz, 6H).

^{13}C NMR (101 MHz, Chloroform-d) δ 137.1, 131.6, 124.3, 122.4, 80.4, 39.8, 37.8, 27.1, 26.8, 25.8, 20.3, 17.8, 16.2.

IR (ATR, neat) $\tilde{\nu}_{max}$: 2966 (m), 2914 (s), 2855 (m), 1668 (w), 1443 (s), 1376 (m), 1325 (w), 1250 (m), 1152 (w), 1108 (m), 1046 (w), 984 (w), 839 (s), 759 (w), 697 (w), 561 (w), 524 (w), 450 (m) cm^{-1}.

HRMS (ESI): calcd for $C_{13}H_{20}Br^+$ $[M+H]^+$: 255.0743; found: 255.0735.

[1] The reaction mixture was protected from light with aluminum foil.

2.1.3 (*Z*)-aryl enol ether **14**

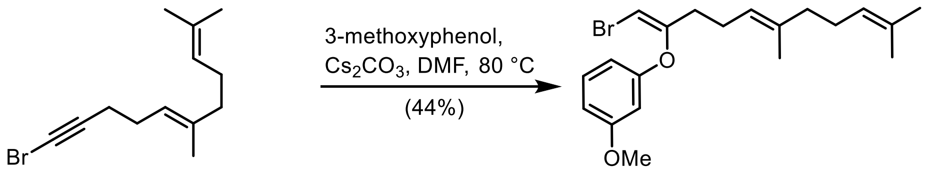

13 **14**

To a suspension of caesium carbonate (9.00 g, 27.6 mmol, 3.00 equiv) in dry dimethylformamide (24 mL) was added 3-methoxyphenol (9.15 g, 73.7 mmol, 8.00 equiv) and alkyne **13** (2.35 g, 9.21 mmol, 1 equiv). The reaction mixture was heated at 80 °C in a sealed tube for 3 d before water (20 mL) was added to the thick brown suspension. The mixture was extracted with diethyl ether (3 x 40 mL) and the combined organic layers were washed with saturated aqueous solution of sodium chloride (40 mL), the washed solution dried over magnesium sulfate and the dried solution was filtered. The filtrate was concentrated and the residue purified by flash column chromatography on silica gel (0.5% grading to 1.0% diethyl ether in pentane) yielding the title compound **14** (1.55 g, 4.09 mmol, 44%) as a colorless oil.[5]

TLC (1% diethyl ether in pentane): R_f: 0.52.

^{1}H NMR (400 MHz, Chloroform-*d*) δ 7.25 – 7.16 (m, 1H), 6.68 – 6.50 (m, 3H), 5.76 (s, 1H), 5.17 – 5.02 (m, 2H), 3.80 (s, 3H), 2.35 – 2.23 (m, 2H), 2.23 – 2.14 (m, 2H), 2.11 – 2.04 (m, 2H), 2.02 – 1.94 (m, 2H), 1.69 (d, *J* = 0.6 Hz, 3H), 1.61 (s, 3H), 1.58 (s, 3H).

^{13}C NMR (101 MHz, Chloroform-*d*) δ 161.1, 156.3, 155.0, 136.9, 131.6, 130.1, 124.3, 122.4, 109.2, 108.4, 103.3, 92.0, 55.5, 39.8, 32.5, 26.8, 25.8, 25.1, 17.8, 16.1.

IR (ATR, neat) $\tilde{\nu}_{max}$: 2964 (w), 1915 (w), 2853 (w), 1645 (w), 1602 (m), 1590 (s), 1487 (s), 1451 (m), 1376 (w), 1330 (w), 1261 (m), 1192 (m), 1165 (m), 1146 (s), 1079 (w), 1042 (m), 969 (w), 837 (w), 765 (m), 744 (w), 686 (m), 568 (w), 455 (w) cm^{-1}.

HRMS (ESI): calcd for $C_{20}H_{27}BrNaO_2^+$ [M+Na]$^+$: 401.1087; found: 401.1039.

2.1.4 Dienediol **16**

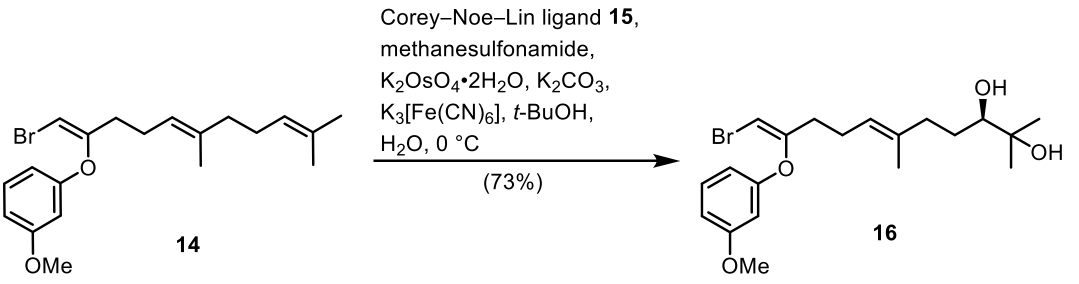

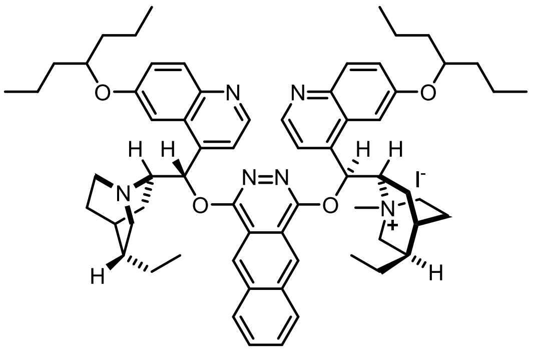

Corey-Noe-Lin-ligand **15**

Potassium carbonate (2.27 g, 16.4 mmol, 4.00 equiv) and $K_3[Fe(CN)_6]$ (5.40 g, 16.4 mmol, 4.00 equiv) were ground to a fine powder in a mortar before adding Corey–Noe–Lin ligand[6] **15** (93.4 mg, 82.0 µmol, 2.00 mol%), $K_2OsO_4•2H_2O$ (15.1 mg, 41.0 µmol, 1.00 mol%) and water (13 mL). After cooling the mixture to 0 °C, methanesulfonamide (390 mg, 4.10 mmol, 1 equiv) and a solution of alkene **14** (1.56 g, 4.10 mmol, 1 equiv) in *tert*-butanol (13 mL) were added. The biphasic suspension was sonicated at 0 °C for 15 min before stirring vigorously at 0 °C for 30 h.[2] Sodium sulfite (5.17 g, 41.0 mmol, 10.0 equiv) was added and the slurry was allowed to reach 22 °C. After 30 min, 1 M aqueous solution of sodium hydroxide was added dropwise until all solids dissolved and the green mixture was extracted with ethyl acetate (4 x 100 mL). The combined organic layers were dried over sodium sulfate, the dried solution was filtered, and the filtrate concentrated. The residue was purified by flash column chromatography on silica gel (2% grading to 40% ethyl acetate in cyclohexane) yielding diol **16** (1.24 g, 3.00 mmol, 73%) as a yellowish oil along with starting material **14** (97.5 mg, 257 µmol, 6%).[3]

Mosher ester analysis:

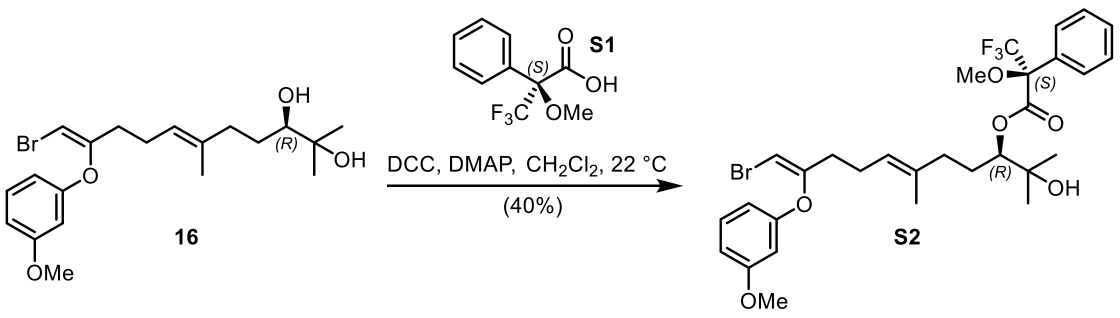

[2] Without sonication the ligand often agglutinated leading to poor yields.
[3] To avoid over-oxidation, the reaction was stopped prior to full conversion.

To a mixture of (*S*)-*α*-methoxy-*α*-(trifluoromethyl)phenylacetic acid (**S1**) (32.5 mg, 139 µmol, 3.10 equiv) and *N*,*N*-dimethylpyridin-4-amine (17.0 mg, 139 µmol, 3.10 equiv) were added a solution of diol **16** (18.5 mg, 44.8 µmol, 1 equiv) in dry dichloromethane (1.0 mL) and dicyclohexylmethanediimine (28.6 mg, 139 µmol, 3.10 equiv) sequentially. After 18 h at 22 °C, the mixture was filtered through a plug of magnesium sulfate and the plug was washed with chloroform (5 mL). The filtrate was concentrated, and the residue purified by flash column chromatography on silica gel (20% ethyl acetate in pentane), yielding (*S*)-Mosher ester **S2** (11.2 mg, 17.8 µmol, 40%) as a colorless oil. The enantiomeric ratio of the diol was determined by ^{1}H NMR (400 MHz, Benzene-d_6) analysis of the corresponding mono-(*S*)-*α*-methoxy-*α*-(trifluoromethyl)phenylacetic acid esters. Comparison of the signals corresponding to the methyl group on the α-methoxy group of the ester δ 3.47 (q, *J* = 1.3 Hz) for the major (*R*)-enantiomer, δ 3.54 (q, *J* = 1.3 Hz) for the minor (*S*)-enantiomer revealed an enantiomeric ratio of 97:3, corresponding to an enantiomeric excess of 94%.[7]

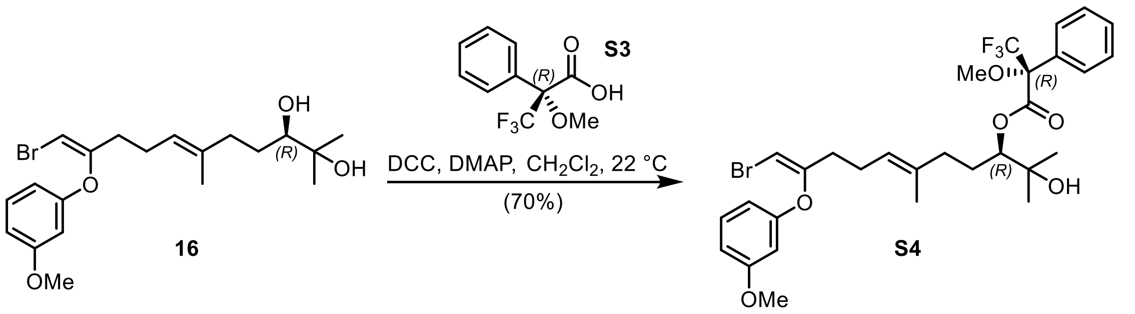

To a mixture of (*R*)-*α*-methoxy-*α*-(trifluoromethyl)phenylacetic acid (**S3**) (17.6 mg, 75.0 µmol, 3.10 equiv) and *N*,*N*-dimethylpyridin-4-amine (9.16 mg, 75.0 µmol, 3.10 equiv) were added a solution of diol **16** (10.0 mg, 24.2 µmol, 1 equiv) in dry dichloromethane (0.50 mL) and dicyclohexylmethanediimine (15.5 mg, 75.0 µmol, 3.10 equiv) sequentially. After 27 h at 22 °C, the mixture was filtered through a plug of magnesium sulfate and the plug was washed with chloroform (5 mL). The filtrate was concentrated, and the residue purified by flash column chromatography on silica gel (10% ethyl acetate in pentane), yielding (*R*)-Mosher ester **S4** (10.7 mg, 17.0 µmol, 70%) as a colorless oil. The enantiomeric ratio of the diol was determined by ^{1}H NMR (400 MHz, Benzene-d_6) analysis of the corresponding mono-(*R*)-*α*-methoxy-*α*-(trifluoromethyl)phenylacetic esters. Comparison of the signals corresponding to the methyl group on the α-methoxy group of the ester: δ 3.54 (q, *J* = 1.3 Hz) for the major (*R*)-enantiomer, δ 3.47 (q, *J* = 1.3 Hz) for the minor (*S*)-enantiomer revealed an enantiomeric ratio of 97:3, corresponding to an enantiomeric excess of 94%.

Analytical data of dienediol **16**:

TLC (50% ethyl acetate in pentane): R_f: 0.32.

^{1}H NMR (400 MHz, Chloroform-*d*) δ 7.20 (t, *J* = 8.2 Hz, 1H), 6.61 (ddd, *J* = 8.3, 2.3, 0.8 Hz, 1H), 6.59 – 6.51 (m, 2H), 5.75 (s, 1H), 5.20 – 5.08 (m, 1H), 3.80 (s, 3H), 3.33 (dd, *J* = 10.5, 1.9 Hz, 1H), 2.30 – 2.15 (m, 5H), 2.15 – 1.99 (m, 2H), 1.92 (s, 1H), 1.62 – 1.53 (m, 4H), 1.39 (dddd, *J* = 13.9, 10.5, 8.7, 5.3 Hz, 1H), 1.20 (s, 3H), 1.15 (s, 3H).

^{13}C NMR (101 MHz, Chloroform-*d*) δ 161.1, 156.2, 154.9, 136.8, 130.2, 123.1, 109.2, 108.4, 103.3, 92.1, 78.2, 73.2, 55.5, 36.8, 32.4, 29.8, 26.6, 25.1, 23.4, 16.1.

IR (ATR, neat) $\tilde{\nu}_{max}$: 3406 (br, w), 2927 (w), 1646 (w), 1602 (m), 1589 (m), 1488 (m), 1451 (m), 1382 (w), 1330 (w), 1261 (m), 1192 (m), 1165 (m), 1146 (s), 1076 (m), 1042 (m), 965 (m), 934 (w), 845 (w), 765 (m), 744 (m), 686 (m), 573 (w), 457 (w) cm^{-1}.

HRMS (ESI): calcd for $C_{20}H_{29}BrNaO_4^+$ [M+Na]$^+$: 435.1141; found: 435.1145.

$[\alpha]_D^{20}$ = +8.6 (c = 1.3, dichloromethane).

Analytical data of (*S*)-*α*-methoxy-*α*-(trifluoromethyl)phenylacetic acid ester **S2**:

TLC (20% ethyl acetate in pentane): R_f: 0.34.

^{1}H NMR (400 MHz, Benzene-*d*$_6$) δ 7.83 – 7.75 (m, 2H), 7.14 – 7.09 (m, 2H), 7.08 – 7.03 (m, 1H), 6.99 (t, *J* = 8.2 Hz, 1H), 6.69 (t, *J* = 2.3 Hz, 1H), 6.59 (ddd, *J* = 8.2, 2.4, 0.9 Hz, 1H), 6.48 (ddd, *J* = 8.4, 2.5, 0.9 Hz, 1H), 5.52 (s, 1H), 5.09 – 5.02 (m, 2H), 3.47 (q, *J* = 1.3 Hz, 3H), 3.25 (s, 3H), 2.12 – 1.96 (m, 6H), 1.71 (dddd, *J* = 14.4, 9.5, 7.4, 2.3 Hz, 1H), 1.64 – 1.53 (m, 1H), 1.43 (d, *J* = 1.4 Hz, 3H), 1.11 (s, 1H), 0.99 – 0.93 (m, 6H).

^{13}C NMR (101 MHz, Benzene-*d*$_6$) δ 166.6, 161.7, 156.8, 155.0, 135.4, 132.9, 130.4, 129.8, 128.6, 128.0*, 124.2, 109.3, 108.7, 103.6, 92.6, 85.21 (d, *J* = 27.6 Hz), 82.2, 72.0, 55.5, 54.9, 36.4, 32.3, 28.7, 25.9, 25.3, 25.1, 15.8. (The signal of the CF_3-group could not be detected; the signal marked with * overlapped with the solvent-peak, but could be assigned via the HSQC spectrum)

IR (ATR, neat) $\tilde{\nu}_{max}$: 3536 (br, w), 2946 (w), 2849 (w), 1743 (m), 1647 (w), 1604 (m), 1488 (m), 1451 (m), 1373 (w), 1261 (s), 1149 (s), 1018 (m), 920 (w), 884 (w), 845 (w), 765 (m), 716 (m), 688 (w) cm^{-1}.

HRMS (ESI): calcd for $C_{30}H_{36}BrF_3O_6Na^+$ [M+Na]$^+$: 651.1540; found: 651.1531.

$[\alpha]_D^{20}$ = –10.5 (c = 0.75, dichloromethane).

Analytical data of (*R*)-*α*-methoxy-*α*-(trifluoromethyl)phenylacetic acid ester **S4**:

TLC (20% ethyl acetate in hexanes): R_f: 0.35.

^{1}H NMR (400 MHz, Benzene-*d*$_6$) δ 7.86 (d, *J* = 7.8 Hz, 2H), 7.15 – 7.11 (m, 2H), 7.09 – 7.04 (m, 1H), 6.99 (t, *J* = 8.2 Hz, 1H), 6.69 (t, *J* = 2.4 Hz, 1H), 6.58 (ddd, *J* = 8.1, 2.3, 0.9 Hz, 1H), 6.48 (ddd, *J* = 8.3, 2.4, 0.9 Hz, 1H), 5.52 (s, 1H), 5.05 – 4.94 (m, 2H), 3.54 (q, *J* = 1.3 Hz, 3H), 3.25 (s, 3H), 2.10 – 1.98 (m, 4H), 1.88 (t, *J* = 7.7 Hz, 2H), 1.56 – 1.42 (m, 2H), 1.38 (d, *J* = 1.3 Hz, 3H), 1.14 (s, 1H), 1.02 (s, 3H), 0.93 (s, 3H).

^{13}C NMR (101 MHz, Benzene-d$_6$) δ 167.2, 161.7, 156.8, 154.9, 135.4, 133.1, 130.4, 129.8, 128.6, 128.3*, 124.0, 109.3, 108.7, 103.6, 92.6, 85.37 (d, *J* = 27.2 Hz), 82.0, 72.3, 55.6, 54.9, 36.1, 32.4, 28.8, 26.8, 25.2, 24.0, 15.8. (The signal of the CF_3-group could not be detected; the signal marked with * overlapped with the solvent-peak, but could be assigned via the HSQC spectrum)

IR (ATR, neat) $\tilde{\nu}_{max}$: 3536 (br, w), 2962 (w), 2935 (w), 2852 (w), 1743 (m), 1646 (w), 1603 (m), 1591 (m), 1489 (m), 1467 (w), 1452 (m), 1372 (w), 1329 (w), 1263(s), 1188 (s), 1166 (s), 1148 (s), 1127 (m), 1081 (w), 1040 (m), 1020 (m), 996 (m), 967 (w), 922 (w), 884 (w), 8457 (w), 766 (w), 716 (w) cm^{-1}.

HRMS (ESI): calcd for $C_{30}H_{36}BrF_3O_6Na^+$ [M+Na]$^+$: 651.1540; found: 651.1526.

$[\alpha]_D^{20}$ = +19.2 (c = 0.99, dichloromethane).

2.1.5 Epoxide fragment **17**

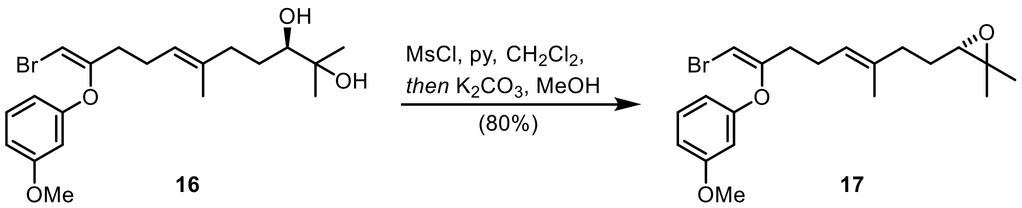

To a solution of diol **16** (2.08 g, 5.03 mmol, 1 equiv) and dry pyridine (2.03 mL, 25.1 mmol, 5.00 equiv) in dry dichloromethane (20 mL) was added methanesulfonyl chloride (584 µl, 7.54 mmol, 1.50 equiv) at 0 °C. The cooling bath was removed and the reaction mixture was allowed to warm to 22 °C.[4] After 15.5 h, the solution was cooled to 0 °C, additional methanesulfonyl chloride (118 µl, 1.51 mmol, 0.300 equiv) was added and the mixture was warmed to 22 °C. After 2.5 h, the mixture was concentrated, benzene (10 mL) was added to the residue and the solution was concentrated. Dry methanol (40 mL) and potassium carbonate (2.78 g, 20.1 mmol, 4.00 equiv) were added to the residue.[5] After 17 h, the thick slurry was concentrated to about half of its volume. Water (30 mL) was added to the suspension and the mixture was extracted three times with dichloromethane (3 x 50 mL). The combined organic layers were dried over magnesium sulfate, the dried solution was filtered, and the filtrate was concentrated. The residue was purified by flash column chromatography on silica gel (5% ethyl acetate in cyclohexane) yielding epoxide **17** (1.60 g, 4.04 mmol, 80%) as a colorless oil.

TLC (20% ethyl acetate in cyclohexane): R_f: 0.50.

^{1}H NMR (400 MHz, Benzene-d_6) δ 7.08 (t, J = 8.2 Hz, 1H), 6.79 (t, J = 2.3 Hz, 1H), 6.68 (dq, J = 8.2, 0.9 Hz, 1H), 6.59 (ddd, J = 8.3, 2.4, 0.7 Hz, 1H), 5.55 (s, 1H), 5.20 – 4.96 (m, 1H), 3.35 (s, 3H), 2.62 (dd, J = 6.7, 5.6 Hz, 1H), 2.20 – 2.02 (m, 6H), 1.66 – 1.55 (m, 2H), 1.53 (d, J = 1.0 Hz, 3H), 1.25 (s, 3H), 1.19 (s, 3H).

^{13}C NMR (101 MHz, Benzene-d_6) δ 161.7, 156.8, 155.1, 136.1, 130.4, 123.3, 109.3, 108.7, 103.6, 92.4, 63.4, 57.4, 54.9, 36.8, 32.5, 27.9, 25.3, 25.0, 18.9, 16.0.

IR (ATR, neat) $\tilde{\nu}_{max}$: 3091 (w), 2960 (w), 2923 (w), 1646 (w), 1602 (m), 1590 (s), 1488 (s), 1452 (m), 1378 (w), 1262 (m), 1193 (m), 1166 (m), 1147 (s), 1079 (w), 1042 (m), 696 (w), 849 (w), 767 (m), 686 (w), 569 (w), 458 (w) cm^{-1}.

HRMS (ESI): calcd for $C_{20}H_{27}BrKO_3^+$ [M+K]$^+$: 433.0775; found: 433.0772.

$[\alpha]_D^{20}$ = -3.2 (c = 1.8, dichloromethane).

[4] Methanesulfonyl chloride was freshly distilled from P_4O_{10} through a Vigreux column under a N_2 atmosphere.
[5] Potassium carbonate was ground to a fine powder in a mortar before use.

2.1.6 Cyclization precursor **9**

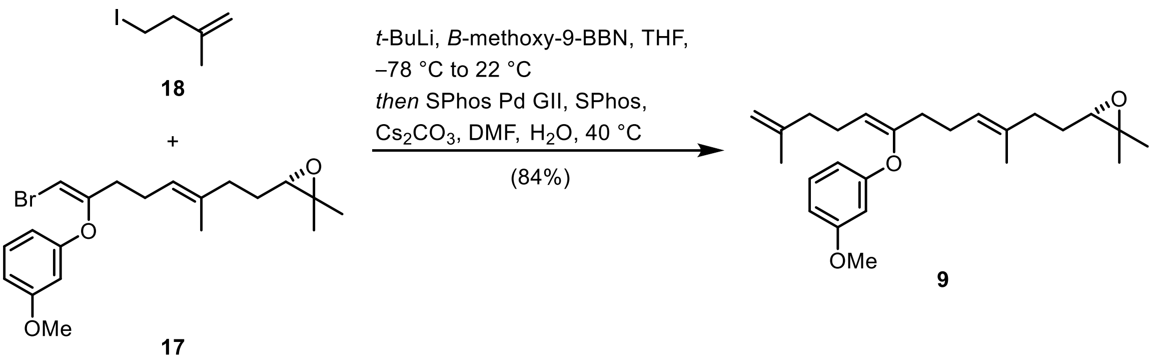

To a solution of iodide **18** (1.58 g, 8.08 mmol, 1.50 equiv) and 9-methoxy-9-borabicyclo[3.3.1]nonane (1.00 M in hexanes, 18.9 mL, 18.9 mmol, 3.50 equiv) in degassed dry tetrahydrofuran (75 mL) was added *tert*-butyllithium (1.70 M in pentane, 14.3 mL, 24.2 mmol, 4.50 equiv) dropwise at −78 °C. The solution turned yellow and then colorless. After 60 min, the cooling bath was replaced by a water bath (22 °C) and the reaction mixture was warmed to 22 °C. The reaction mixture was cooled to −78 °C after 5 min. A degassed 9:1 mixture of dimethylformamide and water (10 mL) was added to the clear solution. The cooling bath was replaced by a water bath (22 °C) and the reaction mixture was warmed to 22 °C.

A separate flask was charged with vinyl bromide **17** (2.13 g, 5.39 mmol, 1 equiv), caesium carbonate (3.51 g, 10.8 mmol, 2.00 equiv), 2-dicyclohexylphosphino-2′,6′-dimethoxybiphenyl (111 mg, 269 µmol, 5.00 mol%), chloro(2-dicyclohexylphosphino-2′,6′-dimethoxy-1,1′-biphenyl)[2-(2′-amino-1,1′-biphenyl)]palladium(II) (194 mg, 269 µmol, 5.00 mol%) and a degassed 9:1 mixture of dimethylformamide and water (136 mL). To the yellow suspension was added the preformed boronate-species via cannulation and the biphasic mixture was heated at 40 °C. After 6 h, water (75 mL) was added and the mixture was extracted with ethyl acetate (3 x 150 mL). The combined organic layers were washed with water (3 x 100 mL) and saturated aqueous solution of sodium chloride (100 mL). The solution was dried over magnesium sulfate, the dried solution filtered, and the filtrate concentrated. The residue was purified by flash column chromatography on silica gel (5% ethyl acetate in pentane) yielding cyclization precursor **9** (1.75 g, 4.55 mmol, 84%) as a colorless oil.

TLC (10% ethyl acetate in hexanes): R_f: 0.46.

^{1}H NMR (400 MHz, Benzene-*d*$_6$) δ 7.05 (t, *J* = 8.2 Hz, 1H), 6.75 (t, *J* = 2.3 Hz, 1H), 6.68 (ddd, *J* = 8.2, 2.3, 0.9 Hz, 1H), 6.48 (ddd, *J* = 8.3, 2.4, 0.9 Hz, 1H), 5.23 – 5.14 (m, 1H), 4.97 (t, *J* = 7.1 Hz, 1H), 4.86 – 4.71 (m, 2H), 3.31 (s, 3H), 2.54 (dd, *J* = 6.7, 5.7 Hz, 1H), 2.30 (q, *J* = 7.4 Hz, 2H), 2.26 – 2.16 (m, 4H), 2.15 – 1.97 (m, 4H), 1.62 – 1.46 (m, 8H), 1.14 (s, 3H), 1.09 (s, 3H).

^{13}C NMR (101 MHz, Benzene-*d*$_6$) δ 161.8, 158.6, 151.1, 145.2, 135.2, 130.4, 124.3, 116.0, 110.8, 108.7, 107.6, 102.9, 63.5, 57.4, 54.8, 37.8, 36.8, 32.9, 28.0, 25.9, 25.0, 23.8, 22.3, 18.9, 16.1.

IR (ATR, neat) $\tilde{\nu}_{max}$: 2959 (w), 2922 (w), 2852 (w), 1685 (w), 1649 (w), 1601 (m), 1591 (m), 1488 (m), 1452 (m), 1377 (w), 1327 (w), 1281 (m), 1263 (m), 1193 (m), 1165 (m), 1143 (s), 1078 (w), 1042 (m), 979 (w), 886 (m), 849 (w), 766 (m), 687 (m) cm^{-1}.

HRMS (ESI): calcd for $C_{25}H_{36}NaO_3^+$ $[M+Na]^+$: 407.2557; found: 407.2560.

$[\boldsymbol{\alpha}]_{\boldsymbol{D}}^{\mathbf{20}}$ = –2.1 (c = 1.7, dichloromethane).

2.1.7 Pentacycle **8a** and **8b**

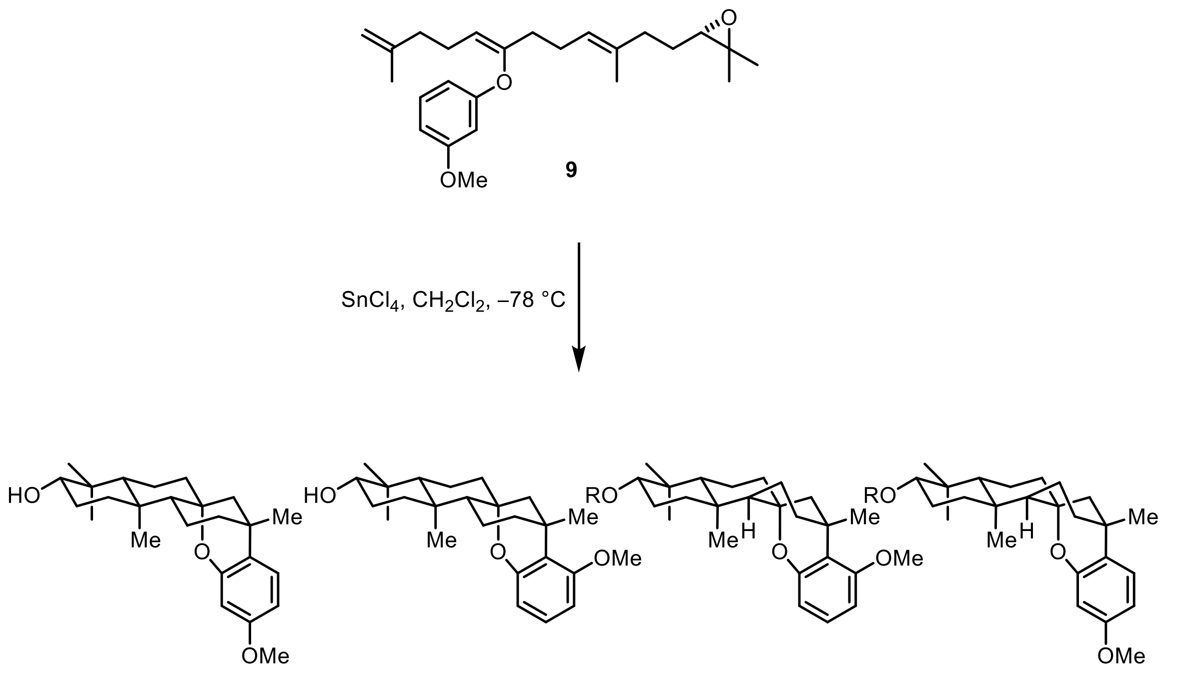

8a (16%)

8b (10%)

BzCl → **8c**: R=H; **19**: R=Bz (9% over 2 steps)

BzCl → **8d**: R=H; **20**: R=Bz (12% over 2 steps)

To a solution of cyclization precursor **9** (100 mg, 260 µmol, 1 equiv) in dry dichloromethane (34 mL) was added $SnCl_4$ (100 mM in dichloromethane, 3.90 mL, 390 µmol, 1.50 equiv) dropwise over 130 sec at –78 °C. After 20 min, triethylamine (144 µl, 1.04 mmol, 4.00 equiv) was added to the yellow solution leading to decolorization and the reaction mixture was poured into 2 M aqueous solution of sodium hydroxide (34 mL). The aqueous layer was extracted with ethyl acetate (3 x 10 mL), and the combined organic layers were dried over magnesium sulfate. The dried solution was filtered, and the filtrate was concentrated. Purification of the residue by flash column chromatography on silica gel (7.5% grading to 10% ethyl acetate in pentane) yielded 58.5 mg of a mixture of tetracyclization products **8a**, **8b**, **8c** and **8d** along with other impurities. The mixture was separated by semipreparative normal-phase HPLC (1.5% grading to 2.5% *i*-propanol in *n*-hexane over 30 min) to yield **8a** (15.8 mg, 41.1 µmol, 16%) as a colorless foam, **8b** (10.2 mg, 26.5 µmol, 10%) as a colorless solid, a mixture of **8c** with other impurities (12.8 mg) and a mixture of **8d** with other impurities (19.4 mg).

To a solution of the impure fraction of **8c** (12.8 mg, assuming 33.3 µmol, 1 equiv) in pyridine (890 µl) was sequentially added *N*,*N*-dimethylpyridin-4-amine (6.1 mg, 50 µmol, 1.5 equiv) and benzoyl chloride (7.7 µl, 67 µmol, 2.0 equiv) at 22 °C. After 29 h, the reaction mixture was concentrated, and the residue dissolved in ethyl acetate (10 mL). The solution was washed with 1 M aqueous solution of sodium hydroxide (5 mL), 1 M aqueous solution of hydrochloric acid (5 mL), saturated aqueous solution of sodium hydrogen carbonate (5 mL) and saturated aqueous solution of sodium chloride (5 mL). The solution was dried over magnesium sulfate, the dried solution was filtered, and the filtrate was concentrated. The residue was purified by flash column chromatography on silica gel (2% ethyl acetate in pentane) yielding diastereomer **19** (10.9 mg, 22.3 µmol, 9% over 2 steps) as a colorless solid.

To a solution of the impure fraction of **8d** (19.4 mg, assuming 50.4 µmol, 1 equiv) in pyridine (1.35 mL) was sequentially added *N*,*N*-dimethylpyridin-4-amine (9.2 mg, 76 µmol, 1.5 equiv) and benzoyl chloride (12 µl, 0.10 mmol, 2.0 equiv) at 22 °C. After 27 h, the reaction mixture

was concentrated and the residue was dissolved in ethyl acetate (10 mL). The solution was washed with 1 M aqueous solution of sodium hydroxide (5 mL), 1 M aqueous solution of hydrochloric acid (5 mL), saturated aqueous solution of sodium hydrogen carbonate (5 mL) and saturated aqueous solution of sodium chloride (5 mL). The solution was dried over magnesium sulfate, the dried solution was filtered, and the filtrate was concentrated. The residue was purified by flash column chromatography on silica gel (2% ethyl acetate in pentane) yielding diastereomer **20** (15.1 mg, 30.9 µmol, 12% over 2 steps) as a colorless oil.

Crystals suitable for X-RAY analysis were obtained by:

- **8a**: Recrystallisation from hot acetonitrile gave colorless crystals
- **8b**: Sublimation at 215 °C under N_2 atmosphere gave colorless crystals
- **19**: Slow evaporation of a solution in a 1:1 mixture of pentane and diethyl ether gave colorless crystals
- All attempts to crystallize **8d** or **20** failed

Cyclization with $FeCl_3$

To a suspension of iron(III) chloride (84.4 mg, 520 µmol, 2.00 equiv) in dry dichloromethane (17 mL), cyclization precursor **9** (100 mg, 260 µmol, 1 equiv) in dry dichloromethane (17 mL) was added over 120 sec at –50 °C. The reaction mixture was slowly warmed to –20 °C over 2 h within the Dewar vessel by switching off the electronically regulated cryostat. Triethylamine (162 µl, 1.17 mmol, 4.50 equiv) was added to the orange suspension leading to a color change to yellow. The reaction mixture was poured into 1 M aqueous solution of sodium hydroxide (34 mL). The aqueous layer was extracted with dichloromethane (4 x 50 mL), and the combined organic layers were dried over magnesium sulfate. The dried solution was filtered, and the filtrate was concentrated. Purification of the residue by flash column chromatography on silica gel (10% ethyl acetate in pentane) yielded a mixture of tetracyclization products **8a**, **8b**, **8c** and **8d** along with other impurities. From the mixture the desired isomers **8a** and **8b** were separated by preparative normal-phase HPLC (1.0% grading to 2.0% *i*-propanol in *n*-hexane over 120 min) to yield **8a** (12.5 mg, 32.5 µmol, 13%) as a colorless foam, **8b** (13.1 mg, 34.1 µmol, 13%) as a colorless solid, a mixture of **8c** with other impurities (14.8 mg) and a mixture of **8d** with other impurities (18.6 mg).

Large-Scale cyclization:

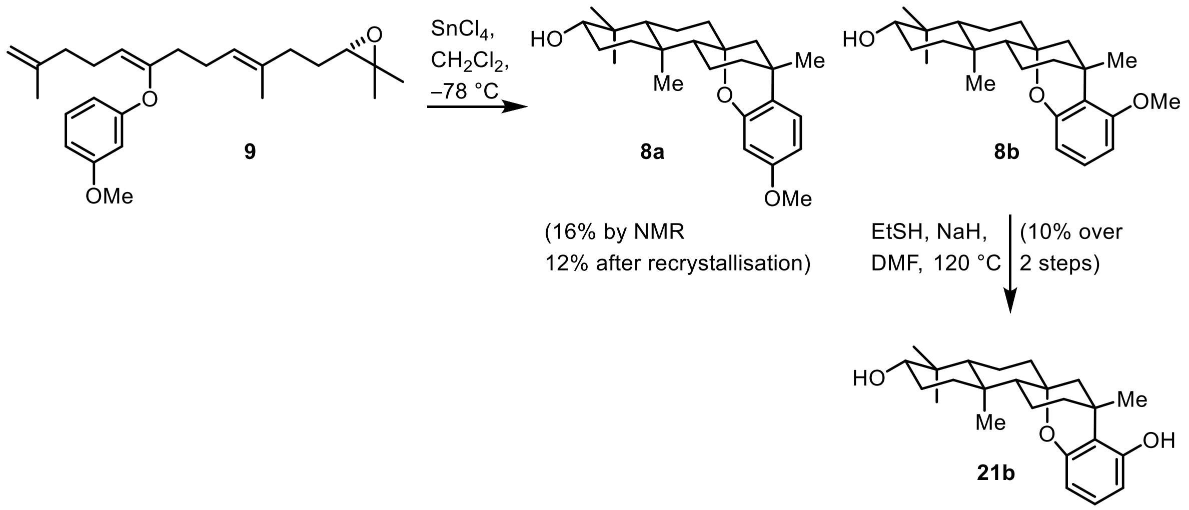

To a solution of cyclization precursor **9** (742 mg, 1.93 mmol, 1 equiv) in dry dichloromethane (276 mL) in a 1-L round bottom flask equipped with a 40-mm olive-shaped magnetic stirring

bar was added $SnCl_4$ (100 mM in dichloromethane, 28.9 mL, 2.89 mmol, 1.50 equiv) via syringe pump (60 mL/ h) at –78 °C under vigorous stirring (600 rpm). After the addition was complete, the solution was stirred for 20 min before triethylamine (1.20 mL, 8.68 mmol, 4.50 equiv) was added dropwise to the orange solution leading to decolorization. The reaction mixture was poured into 1 M aqueous solution of sodium hydroxide (280 mL). The aqueous layer was extracted with dichloromethane (2 x 400 mL) and the combined organic layers were dried over magnesium sulfate. The dried solution was filtered, and the filtrate was concentrated. The residue was purified by flash column chromatography on silica gel (3% grading to 7.5% ethyl acetate in pentane) yielding a mixture of tetracyclization products **8a**, **8b**, **8c** and **8d** along with other impurities. From the mixture the desired isomers **8a** and **8b** were isolated by preparative normal-phase HPLC (1.0% grading to 2.0% *i*-propanol in *n*-hexane over 120 min) yielding **8a** (139 mg, 85% purity by NMR, 305 µmol, 16%) as mixture with other diastereomers, which could be further purified by recrystallisation from refluxing acetonitrile (1 mL) to give clean pentacyclic product **8a** (86.0 mg, 224 µmol, 12%). The regioisomer **8b** was obtained as a mixture with other impurities (92.2 mg), which was used for the next step, after which the impurities could be removed by flash column chromatography on silica gel.

To a solution of impure **8b** (92.2 mg, assuming 240 µmol, 1 equiv) in dry dimethylformamide (4.6 mL) was added a mixture of ethanethiol (36 µl, 480 µmol, 2.0 equiv) and sodium hydride (19 mg, 60% dispersion in mineral oil, 0.48 mmol, 2.0 equiv) in dimethylformamide (1.0 mL) and heated at 120 °C. After 9.5 h, a mixture of ethanethiol (36 µl, 480 µmol, 2.0 equiv) and sodium hydride (19 mg, 60% dispersion in mineral oil, 0.48 mmol, 2.0 equiv) in dimethylformamide (1.0 mL) was added. After 24 h, saturated aqueous solution of ammonium chloride (5 mL) was added to the reaction mixture. Water was added dropwise to the suspension until all solids dissolved. The mixture was extracted with ethyl acetate (3 x 10 mL) and the combined organic layers were washed with water (2 x 10 mL) and saturated aqueous solution of sodium chloride (10 mL). The solution was dried over magnesium sulfate, the dried solution was filtered and the filtrate was concentrated. The residue was purified by flash column chromatography on silica gel (15% grading to 20% ethyl acetate in pentane) yielding phenol **21b** (71.7 mg, 194 µmol, 10% over 2 steps) as a colorless wax.

<u>Analytical data of pentacycle **8a**</u>

TLC (20% ethyl acetate in cyclohexane): R_f: 0.30.

mp: 173 °C.

^{1}H NMR (600 MHz, Chloroform-*d*) δ 7.02 (d, *J* = 8.5 Hz, 1H), 6.40 (dd, *J* = 8.5, 2.6 Hz, 1H), 6.32 (d, *J* = 2.6 Hz, 1H), 3.76 (s, 3H), 3.22 (dt, *J* = 11.5, 5.6 Hz, 1H), 1.93 – 1.82 (m, 2H), 1.67 (dd, *J* = 12.9, 2.8 Hz, 1H), 1.66 – 1.54 (m, 5H), 1.45 (tdd, *J* = 12.2, 7.2, 4.2 Hz, 3H), 1.40 (d, *J* = 12.7 Hz, 1H), 1.31 (s, 3H), 1.27 (d, *J* = 6.2 Hz, 1H), 1.13 (qd, *J* = 13.4, 12.2, 2.7 Hz, 1H), 1.08 – 1.02 (m, 4H), 1.01 – 0.95 (m, 4H), 0.90 (dd, *J* = 12.2, 2.3 Hz, 1H), 0.88 (s, 3H).

^{13}C NMR (151 MHz, Chloroform-*d*) δ 159.2, 156.6, 125.4, 122.1, 105.8, 100.1, 79.2, 76.1, 57.7, 55.7, 55.4, 46.6, 42.0, 40.8, 39.2, 37.8, 37.3, 32.6, 28.5, 27.5, 26.9, 19.2, 18.0, 15.8, 15.2.

IR (ATR, neat) $\tilde{\nu}_{max}$: 3402 (br, w), 2929 (s), 2869 (m), 2848 (m), 1738 (w), 1617 (m), 1582 (m), 1502 (s), 1442 (m), 1387 (w), 1319 (m), 1259 (m), 1202 (m), 1189 (m), 1162 (s), 1151 (s), 1151 (m), 1039 (m), 1004 (m), 984 (m), 935 (w), 882 (w), 831 (w), 786 (w), 729 (w), 633 (w), 468 (w), 445 (w) cm^{-1}.

HRMS (ESI): calcd for $C_{25}H_{36}NaO_3^+$ $[M+Na]^+$: 407.2557; found: 407.2546.

$[\alpha]_D^{20}$ = –11.1 (c = 0.41, dichloromethane).

Analytical data of pentacycle **8b** :

TLC (20% ethyl acetate in cyclohexane): R_f: 0.33.

mp: 211–219 °C: sublimation and decomposition.

^{1}H NMR (400 MHz, Chloroform-*d*) δ 7.00 (t, *J* = 8.2 Hz, 1H), 6.41 (dd, *J* = 8.2, 1.1 Hz, 1H), 6.36 (dd, *J* = 8.1, 1.1 Hz, 1H), 3.75 (s, 3H), 3.21 (dd, *J* = 10.3, 5.5 Hz, 1H), 1.99 – 1.93 (m, 1H), 1.90 – 1.81 (m, 2H), 1.74 (dd, *J* = 13.0, 2.8 Hz, 1H), 1.63 – 1.55 (m, 4H), 1.49 – 1.44 (m, 4H), 1.44 – 1.38 (m, 1H), 1.34 – 1.29 (m, 2H), 1.11 – 1.01 (m, 5H), 1.01 – 0.95 (m, 1H), 0.93 – 0.88 (m, 4H), 0.86 (s, 3H).

^{13}C NMR (101 MHz, Chloroform-*d*) δ 158.9, 157.3, 127.1, 117.5, 108.9, 102.6, 79.2, 75.1, 57.5, 55.7, 55.3, 48.7, 40.9, 39.1, 38.5, 37.7, 37.3, 33.2, 28.5, 27.6, 27.4, 19.8, 17.9, 15.7, 15.1.

IR (ATR, neat) $\tilde{\nu}_{max}$: 3401 (br, w), 2928 (m), 2869 (w), 2839 (w), 1599 (m), 1583 (m), 1464 (m), 1437 (m), 1387 (w), 1310 (w), 1264 (m), 1240 (m), 1190 (m), 1127 (w), 1087 (s), 1029 (m), 1012 (w), 939 (w), 910 (w), 888 (w), 783 (m), 733 (m), 576 (w) cm^{-1}.

HRMS (ESI): calcd for $C_{25}H_{36}NaO_3^+$ [M+Na]$^+$: 407.2557; found: 407.2546.

$[\boldsymbol{\alpha}]_{\boldsymbol{D}}^{\mathbf{20}}$ = –48.9 (c = 0.92, dichloromethane).

Analytical data of diastereomer **19**

TLC (20% ethyl acetate in cyclohexane): R_f: 0.79.

mp: 196–198 °C.

^{1}H NMR (600 MHz, Chloroform-*d*) δ 8.04 (dd, *J* = 8.2, 1.5 Hz, 2H), 7.55 (t, *J* = 7.4 Hz, 1H), 7.44 (t, *J* = 7.8 Hz, 2H), 7.04 (t, *J* = 8.2 Hz, 1H), 6.47 – 6.39 (m, 2H), 4.72 (dd, *J* = 10.6, 5.4 Hz, 1H), 3.81 (s, 3H), 2.25 (dt, *J* = 14.5, 4.2 Hz, 1H), 1.96 (ddd, *J* = 13.5, 10.4, 7.4 Hz, 1H), 1.82 – 1.71 (m, 3H), 1.70 – 1.59 (m, 4H), 1.58 – 1.52 (m, 4H), 1.42 (s, 3H), 1.21 – 1.16 (m, 2H), 1.13 – 1.08 (m, 4H), 1.07 (s, 3H), 0.99 (s, 3H).

^{13}C NMR (151 MHz, Chloroform-*d*) δ 166.4, 158.8, 154.7, 132.9, 131.1, 129.7, 128.5, 127.3, 122.7, 110.8, 103.1, 81.9, 76.8, 55.3, 48.1, 48.0, 47.2, 38.6, 37.4, 36.4, 36.4, 36.3, 30.8, 28.9, 28.5, 24.0, 24.0, 19.5, 18.4, 17.6.

IR (ATR, neat) $\tilde{\nu}_{max}$: 2950 (m), 2930 (m), 2866 (w), 1784 (w), 1715 (s), 1600 (w), 1580 (w), 1466 (m), 1450 (m), 1392 (w), 1365 (w), 1342 (w), 1314 (m), 1273 (s), 1221 (w), 1170 (w), 1113 (m), 1085 (w), 1026 (w), 970 (w), 929 (w), 891 (w), 856 (w), 784 (w), 761 (w), 730 (w), 712 (m) cm^{-1}.

HRMS (ESI): calcd for $C_{32}H_{40}NaO_4$ [M+Na]$^+$: 511.2819; found: 511.2800.

$[\boldsymbol{\alpha}]_{\boldsymbol{D}}^{\mathbf{20}}$ = +52.4 (c = 0.40, dichloromethane).

Analytical data of diastereomer **20**

TLC (20% ethyl acetate in cyclohexane): R_f: 0.76.

^{1}H NMR (400 MHz, Chloroform-*d*) δ 8.07 – 8.03 (m, 2H), 7.59 – 7.53 (m, 1H), 7.45 (t, *J* = 7.6 Hz, 2H), 7.12 (d, *J* = 8.5 Hz, 1H), 6.47 (dd, *J* = 8.6, 2.6 Hz, 1H), 6.31 (d, *J* = 2.6 Hz,

1H), 4.74 (dd, *J* = 10.5, 5.4 Hz, 1H), 3.76 (s, 3H), 2.31 – 2.16 (m, 1H), 1.83 – 1.48 (m, 12H), 1.33 – 1.25 (m, 6H), 1.14 (s, 3H), 1.10 (s, 3H), 0.98 (s, 3H).

^{13}C NMR (101 MHz, Chloroform-*d*) δ 166.5, 159.2, 154.7, 132.9, 131.0, 129.7, 128.5, 126.5, 126.0, 106.8, 101.6, 81.7, 77.9, 55.4, 49.9, 47.5, 44.7, 39.5, 38.8, 36.9, 36.7, 35.9, 30.9, 29.2, 28.8, 24.9, 24.2, 19.7, 18.2, 17.5.

IR (ATR, neat) $\tilde{\nu}_{max}$: 2954 (m), 2869 (w), 1715 (s), 1617 (m), 1580 (w), 1501 (m), 1451 (m), 1314 (m), 1274 (s), 1198 (m), 1160 (m), 1139 (m), 1113 (s), 1082 (w), 1070 (w), 1027 (m), 1003 (w), 972 (m), 712 (m), 495 (w), 455 (w) cm^{-1}.

HRMS (ESI): calcd for $C_{32}H_{40}NaO_4^+$ $[M+Na]^+$: 511.2819; found: 511.2804.

$[\alpha]_D^{20}$ = +52.8 (c = 1.0, dichloromethane).

Analytical data of phenol **21b**

TLC (20% ethyl acetate in cyclohexane): R_f: 0.53.

mp: 201–218 °C: decomposition.

^{1}H NMR (400 MHz, Dichloromethane-d_2) δ 6.86 (t, *J* = 8.0 Hz, 1H), 6.32 (dd, *J* = 8.2, 1.2 Hz, 1H), 6.17 (dd, *J* = 8.0, 1.2 Hz, 1H), 4.80 (s, 1H), 3.23 – 3.13 (m, 1H), 1.99 – 1.92 (m, 1H), 1.90 – 1.79 (m, 2H), 1.72 (dd, *J* = 13.0, 2.8 Hz, 1H), 1.63 – 1.55 (m, 4H), 1.52 – 1.40 (m, 5H), 1.39 – 1.30 (m, 3H), 1.10 – 0.96 (m, 6H), 0.93 – 0.89 (m, 4H), 0.83 (s, 3H).

^{13}C NMR (101 MHz, Dichloromethane-d_2) δ 158.3, 155.2, 127.5, 116.4, 108.6, 107.5, 79.3, 75.7, 57.7, 56.0, 48.8, 41.2, 39.5, 38.9, 38.1, 37.7, 33.4, 28.6, 27.9, 27.7, 20.0, 18.3, 15.9, 15.3.

IR (ATR, neat) $\tilde{\nu}_{max}$: 3551 (w), 3238 (br, w), 3000 (w), 2941 (m), 2926 (m), 2871 (m), 2846 (m), 1606 (w), 1589 (m), 1454 (s), 1380 (w), 1349 (w), 1285 (m), 1243 (m), 1226 (w), 1204 (w), 1186 (m), 1126 (w), 1094 (w), 1066 (w), 1023 (s), 977 (w), 960 (w), 938 (w), 909(m), 889 (w), 852 (w), 786 (m), 734 (s), 649 (w), 576 (w), 504 (w) cm^{-1}.

HRMS (ESI): calcd for $C_{24}H_{35}O_3^+$ $[M+H]^+$: 371.2581; found: 371.2573.

$[\alpha]_D^{20}$ = –44.8 (c = 0.47, dichloromethane).

2.1.8 Phenol **21a**

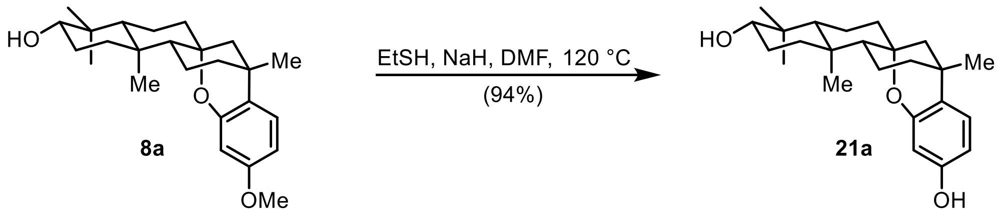

To cyclization product **8a** (83.0 mg, 216 µmol, 1 equiv) was added a mixture of ethanethiol (78 µl, 1.1 mmol, 5.0 equiv) and sodium hydride (43 mg, 60% dispersion in mineral oil, 1.1 mmol, 5.0 equiv) in dry dimethylformamide (4.3 mL) and the suspension was heated at 120 °C. After 16 h, a mixture of ethanethiol (78 µl, 1.1 mmol, 5.0 equiv) and sodium hydride (43 mg, 60% dispersion in mineral oil, 1.1 mmol, 5.0 equiv) in dry dimethylformamide (4.3 mL) was added. The mixture was heated at 120 °C for 21 h before saturated aqueous solution of ammonium chloride (20 mL) was added. Water was added dropwise until all solids dissolved and the mixture was extracted with ethyl acetate (3 x 20 mL). The combined organic layers were washed with water (3 x 10 mL) and saturated aqueous solution of sodium chloride (10 mL) and dried over magnesium sulfate. The dried solution was filtered, and the filtrate was concentrated. Purification of the residue by flash column chromatography on silica gel (20% grading to 30% ethyl acetate in pentane) afforded phenol **21a** (75.2 mg, 203 µmol, 94%) as a colorless foam.

TLC (40% ethyl acetate in cyclohexane): R_f: 0.57.

mp: 194–196 °C.

^{1}H NMR (400 MHz, Chloroform-*d*) δ 6.97 (d, *J* = 8.3 Hz, 1H), 6.32 (dd, *J* = 8.3, 2.6 Hz, 1H), 6.25 (d, *J* = 2.5 Hz, 1H), 5.12 (s, 1H), 3.25 (dd, *J* = 10.5, 5.3 Hz, 1H), 1.94 – 1.78 (m, 2H), 1.70 – 1.51 (m, 7H), 1.48 – 1.37 (m, 4H), 1.29 (s, 3H), 1.19 – 1.08 (m, 1H), 1.07 – 1.01 (m, 4H), 1.01 – 0.92 (m, 4H), 0.92 – 0.86 (m, 4H).

^{13}C NMR (101 MHz, Chloroform-*d*) δ 156.7, 154.9, 125.7, 122.3, 106.6, 101.8, 79.2, 76.1, 57.6, 55.6, 46.4, 42.0, 40.7, 39.1, 37.8, 37.3, 32.6, 28.5, 27.4, 26.9, 19.1, 17.9, 15.8, 15.1.

IR (ATR, neat) $\tilde{\nu}_{max}$: 3368 (br, m), 2944 (s), 2871 (m), 2847 (m), 1721 (m), 1619 (m), 1593 (m), 1504 (s), 1455 (s), 1374 (m), 1286 (m), 1258 (m), 1166 (s), 1145 (s), 1102 (m), 1068 (w), 1029 (m), 1005 (s), 988 (m), 963 (w), 935 (w), 883 (w), 844 (w)m), 799 (w), 747 (w), 632(w), 613 (w), 509 (w), 418 (w) cm^{-1}.

HRMS (ESI): calcd for $C_{24}H_{35}O_3^+$ $[M+H]^+$:371.2581; found: 371.2577.

$[\alpha]_D^{20}$ = –11.6 (c = 1.0, dichloromethane).

2.1.9 Phenol **21b**

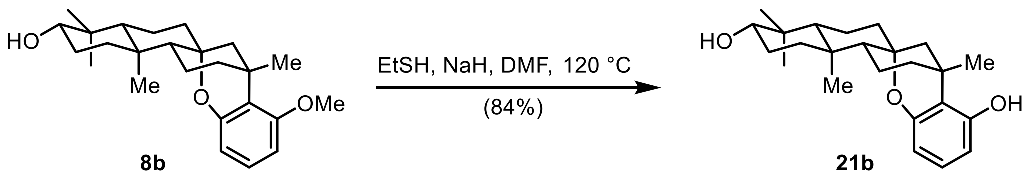

To cyclizationproduct **8b** (36.8 mg, 95.7 µmol, 1 equiv) was added a mixture of ethanethiol (14 µl, 0.19 mmol, 2.0 equiv) and sodium hydride (7.7 mg, 60% dispersion in mineral oil, 0.19 mmol, 2.0 equiv) in dry dimethylformamide (1.8 mL) and heated at 120 °C. After 20 h, a mixture of ethanethiol (7.1 µl, 96 µmol, 1.0 equiv) and sodium hydride (3.8 mg, 60% dispersion in mineral oil, 96 µmol, 1.0 equiv) in dry dimethylformamide (900 µL) was added. After 7 h at 120 °C, saturated aqueous solution of ammonium chloride (1 mL) was added. Water was added dropwise until all solids dissolved. The mixture was extracted with ethyl acetate (3 x 15 mL) and the combined organic layers were washed with water (10 mL) and saturated aqueous solution of sodium chloride (10 mL) and the washed solution dried over magnesium sulfate. The dried solution was filtered and the filtrate was concentrated. Flash column chromatography on silica gel (10% ethyl acetate in pentane) of the residue yielded phenol **21b** (29.9 mg, 80.7 µmol, 84%) as a colorless wax.

Compare chapter 2.1.7 for analytical data

2.1.10 Protected pentacycle **22a**

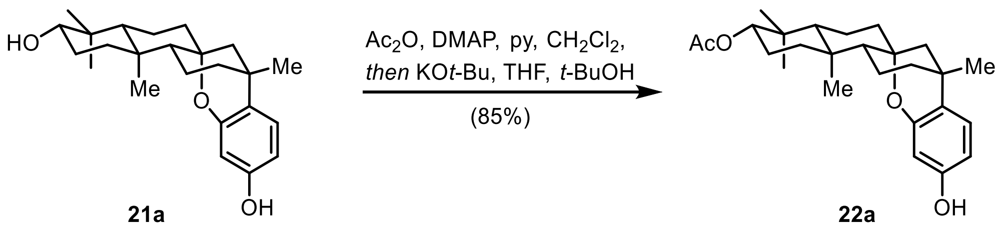

To a solution of phenol **21a** (81.6 mg, 220 µmol, 1 equiv) in dry dichloromethane were sequentially added *N*,*N*-dimethylpyridin-4-amine (26.9 mg, 220 µmol, 1.00 equiv), acetic anhydride (62 µl, 0.66 mmol, 3.0 equiv) and pyridine (89 µl, 1.1 mmol, 5.0 equiv) at 22 °C. After 2 h, the mixture was concentrated and dry benzene (3 mL) was added to the residue. The mixture was concentrated before dry tetrahydrofuran (4.9 mL) and potassium *tert*-butoxide (906 mM in *tert*-butanol, 972 µl, 881 µmol, 4.00 equiv) were sequentially added to the residue at 22 °C sequentially. After 20 min, potassium *tert*-butoxide (906 mM in *tert*-butanol, 49 µl, 44 µmol, 0.20 equiv) was added. After 10 min, saturated aqueous solution of ammonium chloride (5 mL) was added and the mixture was extracted with ethyl acetate (3 x 10 mL). The combined organic layers were dried over magnesium sulfate, the dried solution was filtered and the filtrate was concentrated. The residue was purified by flash column chromatography on silica gel (7.5% grading to 10% ethyl acetate in pentane) yielding phenol **22a** (77.2 mg, 187 µmol, 85%) as a colorless oil.[8]

TLC (20% ethyl acetate in cyclohexane): R_f: 0.42.

^{1}H NMR (400 MHz, Chloroform-*d*) δ 6.98 (d, *J* = 8.3 Hz, 1H), 6.32 (dd, *J* = 8.3, 2.6 Hz, 1H), 6.24 (d, *J* = 2.6 Hz, 1H), 4.65 (s, 1H), 4.50 (dd, *J* = 8.9, 7.1 Hz, 1H), 2.06 (s, 3H), 1.93 – 1.78 (m, 2H), 1.68 – 1.60 (m, 4H), 1.59 – 1.53 (m, 2H), 1.49 – 1.38 (m, 4H), 1.30 (s, 3H), 1.19 – 1.01 (m, 3H), 1.00 – 0.96 (m, 4H), 0.94 (s, 3H), 0.90 (s, 3H).

^{13}C NMR (101 MHz, Chloroform-*d*) δ 171.3, 156.6, 154.9, 125.7, 122.4, 106.6, 101.8, 81.1, 76.0, 57.5, 55.7, 46.4, 41.9, 40.6, 38.0, 37.4, 37.2, 32.5, 28.5, 26.9, 23.7, 21.5, 19.1, 17.8, 16.9, 15.2.

IR (ATR, neat) $\tilde{\nu}_{max}$: 3396 (br, w), 2947 (m), 2873 (w), 2845 (w), 1705 (m), 1618 (m), 1593 (w), 1504 (m), 1450 (m), 1375 (m), 1248 (s), 1210 (m), 1167 (m), 1142 (s), 1103 (m), 1068 (w), 1014 (m), 988 (s), 907 (s), 883 (w), 844 (m), 800 (w), 729 (s), 648 (w), 558 (w), 511 (w) cm^{-1}.

HRMS (ESI): calcd for $C_{26}H_{36}O_4Na^+$ [M+Na]$^+$: 435.2506; found: 435.2491.

$[\alpha]_D^{20}$ = +2.1 (c = 0.62, dichloromethane).

2.1.11 Protected pentacycle **22b**

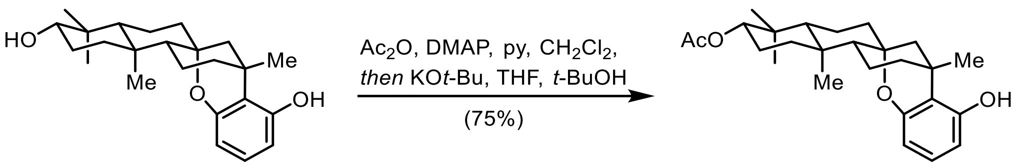

21b **22b**

To a solution of phenol **21b** (71.7 mg, 194 µmol, 1 equiv) in dry dichloromethane was added *N*,*N*-dimethylpyridin-4-amine (23.6 mg, 194 µmol, 1 equiv), acetic anhydride (55 µl, 0.58 mmol, 3.0 equiv) and pyridine (78 µl, 0.97 mmol, 5.0 equiv) at 22 °C. After 2 h, the mixture was concentrated and dry benzene (3 mL) was added to the residue. The mixture was concentrated again before dry tetrahydrofuran (4.3 mL) and potassium *tert*-butoxide (907 mM in *tert*-butanol, 853 µl, 774 µmol, 4.00 equiv) were added to the residue at 22 °C. After 4 h, saturated aqueous solution of ammonium chloride (5 mL) was added and the mixture was extracted with ethyl acetate (3 x 10 mL). The combined organic layers were dried over magnesium sulfate, the dried solution was filtered, and the filtrate concentrated. The residue was purified by flash column chromatography on silica gel (10% ethyl acetate in pentane) yielding phenol **22b** (60.0 mg, 145 µmol, 75%) as a yellow wax.[8]

TLC (10% ethyl acetate in hexanes): R_f: 0.24.

^{1}H NMR (400 MHz, Chloroform-*d*) δ 6.89 (t, *J* = 8.0 Hz, 1H), 6.36 (dd, *J* = 8.2, 1.2 Hz, 1H), 6.17 (dd, *J* = 7.9, 1.2 Hz, 1H), 4.75 (s, 1H), 4.53 – 4.45 (m, 1H), 2.06 (s, 3H), 1.98 (dt, *J* = 13.0, 3.1 Hz, 1H), 1.93 – 1.81 (m, 2H), 1.76 (dd, *J* = 13.0, 2.8 Hz, 1H), 1.67 – 1.55 (m, 4H), 1.54 (s, 3H), 1.50 – 1.39 (m, 2H), 1.39 – 1.31 (m, 2H), 1.16 – 1.02 (m, 3H), 1.01 – 0.95 (m, 4H), 0.93 (s, 3H), 0.90 (s, 3H).

^{13}C NMR (101 MHz, Chloroform-*d*) δ 171.3, 157.8, 154.6, 127.2, 116.0, 108.4, 107.2, 81.1, 75.2, 57.3, 55.8, 48.4, 40.7, 38.5, 38.1, 37.4, 37.2, 33.1, 28.4, 27.4, 23.7, 21.5, 19.7, 17.8, 16.9, 15.1.

IR (ATR, neat) $\tilde{\nu}_{max}$: 3432 (br, w), 2925 (s), 2871 (m), 2849 (m), 1732 (m), 1709 (s), 1608 (w), 1588 (m), 1456 (s), 1375 (m), 1320 (w), 1308 (w), 1267 (s), 1246 (s), 1203 (w), 1188 (m), 1144 (w), 1125 (w), 1088 (w), 1067 (m), 1033 (s), 1011 (m), 978 (m), 948 (w), 906 (w), 889 (w), 852 (w), 785 (m), 734 (m), 658 (w), 610 (w), 576 (w) cm^{-1}.

HRMS (ESI): calcd for $C_{26}H_{37}O_4^+$ $[M+H]^+$: 413.2686; found: 413.2642.

$[\alpha]_D^{20}$ = –35.2 (c = 0.39, dichloromethane).

2.1.12 Ketolactone **23**

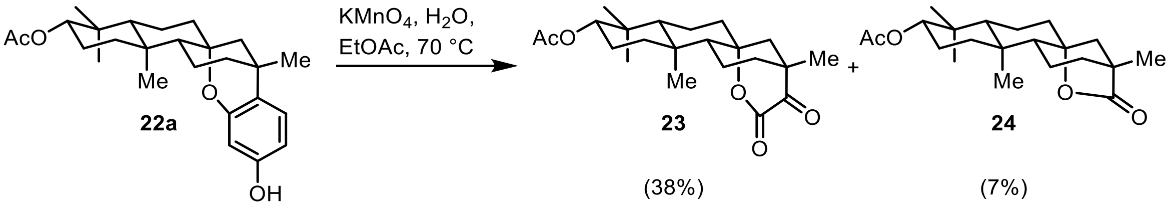

To a solution of phenol **22a** (63.8 mg, 155 µmol, 1 equiv) in ethyl acetate (6.5 mL) was added $KMnO_4$ (489 mg, 3.09 mmol, 20.0 equiv) and water (13 mL). The mixture was heated at 70 °C and a solution of $KMnO_4$ (258 mM in water, 12.0 mL, 3.10 mmol, 20.0 equiv) was added via syringe pump (500 µl/ h). After 24 h, saturated aqueous solution of sodium thiosulfate (16 mL) was added and the suspension was filtered. The filter cake was washed with water (5 mL) and ethyl acetate (5 mL). The aqueous layer of the filtrate was extracted with ethyl acetate (4 x 20 mL) and the combined organic layers were washed with saturated aqueous solution of sodium chloride (20 mL) and the washed solution was dried over sodium sulfate. The dried solution was filtered and the filtrate was concentrated. The residue was purified by flash column chromatography on silica gel (15% grading to 25% ethyl acetate in pentane) yielding ketolactone **23** (22.0 mg, 58.4 µmol, 38%) and lactone **24** (3.7 mg, 11 µmol, 7%) as colorless solids.

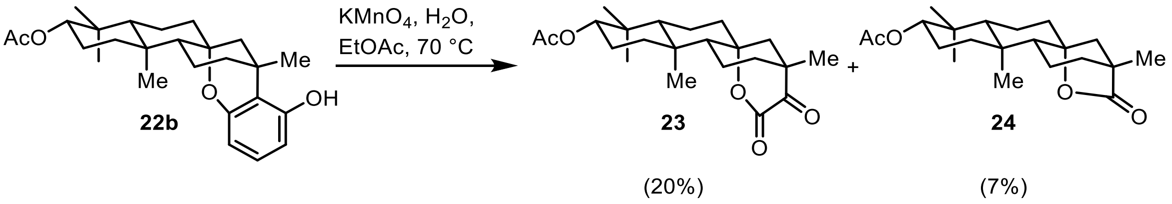

To a solution of phenol **22b** (60.0 mg, 145 µmol, 1 equiv) in ethyl acetate (6.1 mL) was added $KMnO_4$ (460 mg, 2.91 mmol, 20.0 equiv) and water (12 mL). The mixture was heated at 70 °C and a solution of $KMnO_4$ (242 mM in water, 24.0 mL, 5.80 mmol, 40.0 equiv) was added via syringe pump (500 µl/ h). After 48 h, saturated aqueous solution of sodium thiosulfate (30 mL) was added and the suspension was filtered. The filter cake was washed with water (10 mL) and ethyl acetate (10 mL). The aqueous layer of the filtrate was extracted with ethyl acetate (4 x 50 mL) and the combined organic layers were washed with saturated aqueous solution of sodium chloride (50 mL) and the washed solution was dried over sodium sulfate. The dried solution was filtered and the filtrate was concentrated. The residue was purified by flash column chromatography on silica gel (15% grading to 25% ethyl acetate in pentane) yielding ketolactone **23** (11.1 mg, 29.5 µmol, 20%) and lactone **24** (3.6 mg, 10 µmol, 7%) as colorless solids.

<u>Analytical data of ketolactone **23**</u>

TLC (40% ethyl acetate in pentane): R_f: 0.61.

mp: decomposition above 191 °C.

^{1}H NMR (400 MHz, Chloroform-*d*) δ 4.48 (dd, *J* = 11.1, 4.7 Hz, 1H), 2.05 (s, 4H), 1.86 (dd, *J* = 11.5, 2.9 Hz, 1H), 1.80 – 1.72 (m, 2H), 1.70 – 1.55 (m, 7H), 1.53 – 1.40 (m, 2H), 1.17 (s, 4H), 1.12 – 1.04 (m, 1H), 1.00 (s, 3H), 0.96 – 0.91 (m, 4H), 0.89 (s, 3H).

^{13}C NMR (101 MHz, Chloroform-*d*) δ 180.5, 171.2, 83.2, 80.8, 54.5, 52.5, 51.6, 43.9, 37.9, 37.6, 37.4, 36.7, 34.2, 28.7, 23.5, 21.5, 20.6, 19.6, 18.7, 17.2, 14.9.

IR (ATR, neat) $\tilde{\nu}_{max}$: 2923 (s), 2874 (w), 2851 (w), 1733 (s), 1456 (w), 1393 (w), 1373 (w), 1246 (s), 1204 (w), 1171 (w), 1149 (w), 1085 (w), 1029 (w), 1010 (w), 982 (w), 949 (w), 927 (w), 899 (w) cm^{-1}.

HRMS (ESI): calcd for $C_{22}H_{32}O_5Na^+$ $[M+Na]^+$: 399.2142; found: 399.2121.

$[\boldsymbol{\alpha}]_{\boldsymbol{D}}^{\mathbf{20}}$ = –1.6 (c = 0.50, dichloromethane).

Analytical data of lactone **24**

TLC (20% ethyl acetate in pentane): R_f: 0.47.

mp: 186–188 °C.

^{1}H NMR (400 MHz, Chloroform-*d*) δ 4.49 (dd, *J* = 11.2, 4.8 Hz, 1H), 2.22 (dd, *J* = 14.4, 2.6 Hz, 1H), 2.12 – 2.05 (m, 4H), 2.03 – 1.95 (m, 1H), 1.89 – 1.74 (m, 3H), 1.72 – 1.50 (m, 6H), 1.40 – 1.28 (m, 2H), 1.15 (s, 3H), 1.13 – 1.05 (m, 1H), 1.04 – 0.97 (m, 4H), 0.93 – 0.88 (m, 6H).

^{13}C NMR (101 MHz, Chloroform-*d*) δ 194.9, 171.2, 156.3, 83.1, 80.5, 55.1, 54.7, 46.0, 44.3, 40.1, 38.2, 38.0, 37.3, 37.1, 28.4, 23.5, 23.2, 21.4, 18.7, 17.5, 16.9, 15.0.

IR (ATR, neat) $\tilde{\nu}_{max}$: 2947 (m), 2927 (m), 2874 (m), 2857 (w), 1773 (s), 1732 (s), 1457 (m), 1395 (w), 1375 (m), 1366 (m), 1252 (s), 1245 (2), 1220 (m), 1208 (w), 1148 (m), 1128 (w), 1077 (m), 1028 (m), 1008 (m), 979 (w), 948 (w), 916 (w), 901 (w) cm^{-1}.

HRMS (ESI): calcd for $C_{21}H_{32}O_4Na^+$ $[M+Na]^+$: 371.2193; found: 371.2166.

$[\boldsymbol{\alpha}]_{\boldsymbol{D}}^{\mathbf{20}}$ = +4 (c = 0.15, dichloromethane).

2.1.13 Tetraol **25**

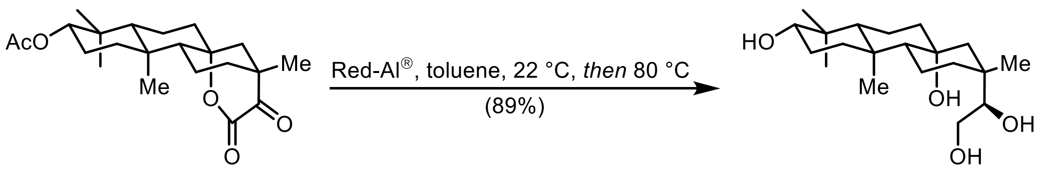

23 **25**

To a solution of keto lactone **23** (22.4 mg, 59.5 µmol, 1 equiv) in dry toluene (2.8 mL) was added sodium bis(2-methoxyethoxy)aluminium hydride (60 wt% in toluene, 0.39 mL, 1.2 mmol, 20 equiv) at 0 °C. The cooling bath was removed, and the reaction mixture was allowed to warm to 22 °C. After 14 h, the mixture was heated at 80 °C for 2 h before ethyl acetate (50 µl) was added dropwise at –78 °C. The reaction mixture was warmed to 22 °C, treated with 2 M aqueous solution of sodium hydroxide (5 mL) and extracted with ethyl acetate (4 x 10 mL). The combined organic layers were dried over sodium sulfate, the dried solution was filtered, and the filtrate was concentrated. The residue was purified by flash column chromatography on silica gel (5% methanol in dichloromethane) yielding tetraol **25** (18.1 mg, 53.2 mmol, 89%) as a colorless solid.

TLC (10% methanol in dichloromethane): R_f: 0.48.

mp: slow decomposition above 129 °C.

^{1}H NMR (400 MHz, Pyridine-d_5) δ 6.64 (s, 1H), 5.72 (s, 1H), 5.38 (s, 1H), 4.39 (dd, *J* = 8.4, 2.9 Hz, 1H), 4.19 – 3.98 (m, 2H), 3.55 (dd, *J* = 11.1, 5.0 Hz, 1H), 2.29 – 2.11 (m, 2H), 2.10 – 1.87 (m, 5H), 1.74 (dt, *J* = 12.9, 3.5 Hz, 1H), 1.63 (ddt, *J* = 14.8, 6.5, 3.0 Hz, 1H), 1.51 (dq, *J* = 13.0, 3.5 Hz, 1H), 1.44 – 1.36 (m, 1H), 1.30 (d, *J* = 4.1 Hz, 7H), 1.16 – 1.08 (m, 4H), 1.05 (dd, *J* = 12.9, 4.5 Hz, 1H), 1.02 – 0.99 (m, 3H), 0.96 (dd, *J* = 12.0, 2.2 Hz, 1H), 0.84 (dd, *J* = 12.6, 3.2 Hz, 1H).

^{13}C NMR (151 MHz, Pyridine-d_5) δ 79.2, 78.9, 71.1, 64.6, 57.5, 56.6, 50.6, 43.8, 40.1, 39.8, 38.9, 38.0, 37.0, 29.5, 29.0, 28.7, 19.8, 19.1, 17.1, 16.7.

IR (ATR, neat) $\tilde{\nu}_{max}$: 3402 (br, m), 2925 (m), 2872 (m), 2851 (m), 1737 (w), 1637 (w), 1442 (s), 1339 (s), 1192 (m), 1149 (m), 1087 (w), 1041 (m), 1011 (m), 833 (w) cm^{-1}.

HRMS (ESI): calcd for $C_{20}H_{37}O_4^+$ [M+H]$^+$: 341.2686; found: 341.2630.

$[\alpha]_D^{20}$ = –8.0 (c = 0.57, methanol).

2.1.14 Thiocarbonate **26**

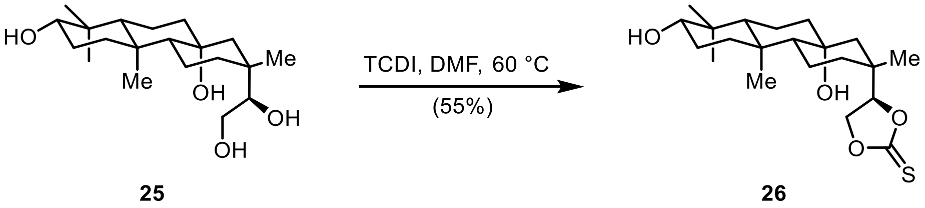

A solution of of di(imidazol-1-yl)methanethione (3.7 mg, 21 µmol, 1.4 equiv) in dry dimethylformamide (1.3 mL) was added to tetraol **25** (5.0 mg, 15 µmol, 1 equiv) and the yellow solution was heated at 60 °C. After 42 h, di(imidazol-1-yl)methanethione (1.3 mg, 7.3 µmol, 0.50 equiv) was added and heated at 60 °C for 30 h, before water (5 mL) was added. The mixture was extracted with ethyl acetate (3 x 5 mL) and the combined organic layers washed with water (5 mL) and saturated aqueous solution of sodium chloride (5 mL) and the washed solution was dried over magnesium sulfate. The dried solution was filtered, and the filtrate was concentrated. The residue was purified by flash column chromatography on silica gel (30% grading to 40% ethyl acetate in pentane) yielding thiocarbonate **26** (3.1 mg, 8.1 µmol, 55%) as a colorless wax.

TLC (40% ethyl acetate in pentane): R_f: 0.52.

^{1}H NMR (400 MHz, Chloroform-*d*) δ 6.11 (dd, *J* = 8.7, 7.0 Hz, 1H), 4.58 (t, *J* = 8.9 Hz, 1H), 4.44 (dd, *J* = 8.8, 7.0 Hz, 1H), 3.20 (dd, *J* = 10.9, 5.3 Hz, 1H), 2.00 (dd, *J* = 14.7, 2.4 Hz, 1H), 1.75 – 1.60 (m, 5H), 1.59 – 1.50 (m, 4H), 1.48 – 1.44 (m, 2H), 1.39 (dd, *J* = 13.3, 4.8 Hz, 1H), 1.34 – 1.29 (m, 1H), 1.12 (d, *J* = 14.7 Hz, 1H), 1.00 (s, 3H), 0.98 – 0.93 (m, 4H), 0.87 – 0.84 (m, 4H), 0.84 – 0.80 (m, 4H).

^{13}C NMR (151 MHz, Chloroform-*d*) δ 192.5, 85.1, 79.0, 72.5, 69.8, 56.5, 55.6, 49.0, 43.4, 39.1, 37.9, 37.1, 36.8, 35.6, 28.4, 27.3, 22.6, 17.6, 17.3, 15.8, 15.6.

IR (ATR, neat) $\tilde{\nu}_{max}$: 3433 (br, w), 2938 (m), 2853 (w), 1451 (w), 1388 (w), 1348 (w), 1295 (s), 1173 (m), 1091 (w), 1031 (w), 1002 (w), 969 (w), 953 (w), 911 (w), 733 (w) cm^{-1}.

HRMS (ESI): calcd for $C_{21}H_{34}O_4SNa^+$ $[M+Na]^+$: 405.2070; found:405.2030.

$[\alpha]_D^{20}$ = +41 (c = 0.21, dichloromethane).

2.1.15 Pimara-15-en-3*α*-8*α*-diol (**7**)

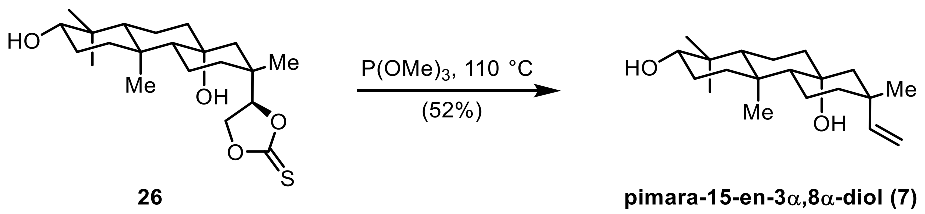

26 **pimara-15-en-3α,8α-diol (7)**

A solution of thiocarbonate **23** (3.1 mg, 8.1 µmol, 1 equiv) in trimethyl phosphite[6] (960 µl) was heated at 110 °C for 32 h. The reaction mixture was concentrated, and the residue was purified by flash column chromatography on silica gel (10% ethyl acetate in pentane) yielding pimara-15-en-3*α*-8*α*-diol (**7**) (1.3 mg, 4.2 µmol, 52%) as an amorphous colorless solid.

TLC (20% ethyl acetate in hexanes): R_f: 0.63.

mp: 143 °C.[7]

^{1}H NMR (600 MHz, Chloroform-*d*) δ 5.98 (dd, *J* = 17.9, 10.9 Hz, 1H), 5.13 (d, *J* = 17.9 Hz, 1H), 5.08 (d, *J* = 10.9 Hz, 1H), 3.20 (dd, *J* = 11.1, 5.2 Hz, 1H), 2.00 (dq, *J* = 13.6, 3.1 Hz, 1H), 1.91 (s, 1H), 1.77 (dt, *J* = 13.3, 3.2 Hz, 1H), 1.73 – 1.66 (m, 2H), 1.64 – 1.56 (m, 3H), 1.50 – 1.43 (m, 3H), 1.24 – 1.18 (m, 3H), 0.99 (s, 3H), 0.99 – 0.95 (m, 1H), 0.93 (s, 3H), 0.90 (s, 3H), 0.85 – 0.81 (m, 2H), 0.80 (s, 3H).

^{13}C NMR (151 MHz, Chloroform-*d*) δ 147.5, 112.0, 79.1, 72.3, 56.2, 55.7, 53.4, 42.0, 39.0, 37.8, 37.0, 36.6, 36.1, 32.4, 28.3, 27.3, 17.8, 17.5, 15.51, 15.47.

IR (ATR, neat) $\tilde{\nu}_{max}$: 3570 (w), 3314 (br, w), 3078 (w), 2939 (s), 2926 (s), 2867 (m), 2852 (m), 1712 (w), 1633 (w), 1453 (m), 1411 (w), 1384 (w), 13133 (w), 1280 (w), 1201 (w), 1146 (w), 1127 (w), 1113 (w), 1085 (w), 1032 (m), 1001 (m), 978 (w), 959 (w), 943 (w), 920 (m), 846 (w), 734 (w), 703 (w), 685 (w) cm^{-1}.

HRMS (ESI): calcd for $C_{20}H_{34}O_2Na^+$ [M+Na]$^+$: 329.2451; found: 329.2440.

$[\alpha]_D^{20}$ = +6 (c = 0.05, chloroform).

---

[6] Trimethyl phosphite was freshly distilled from sodium.
[7] The amorphous sample was heated on a Kofler hot stage microscope where it revealed a broad melting range (95–139 °C). Upon cooling, crystals formed, which had a melting point of 143 °C.

2.2 Synthesis of other cyclization precursors

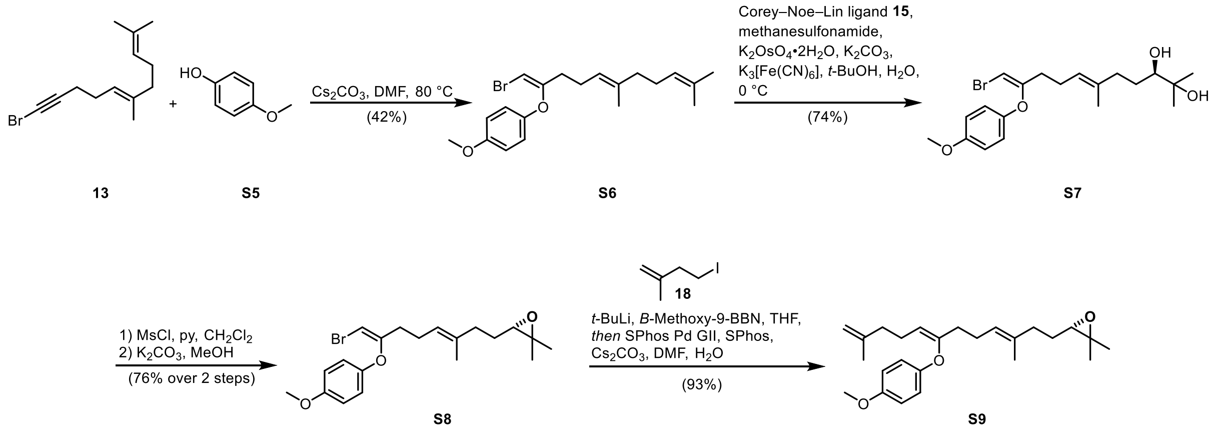

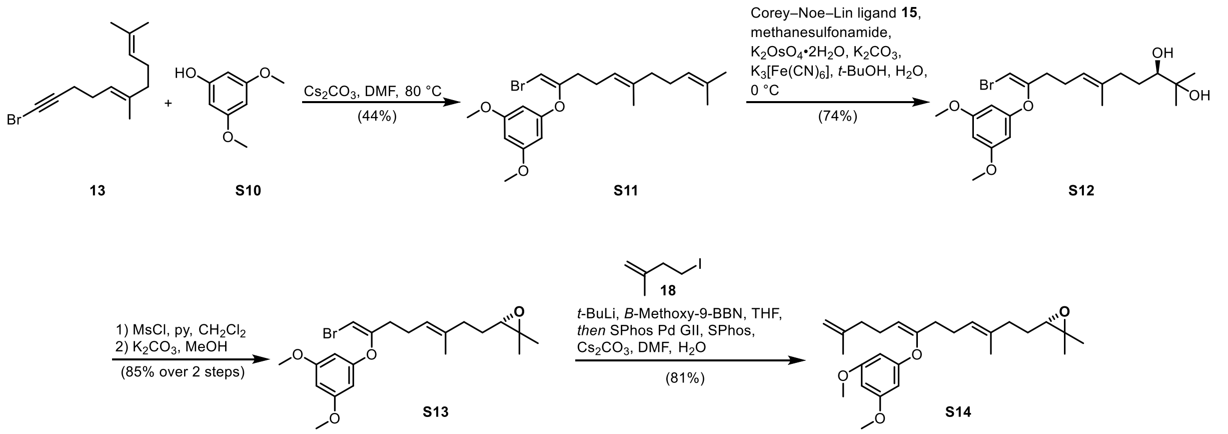

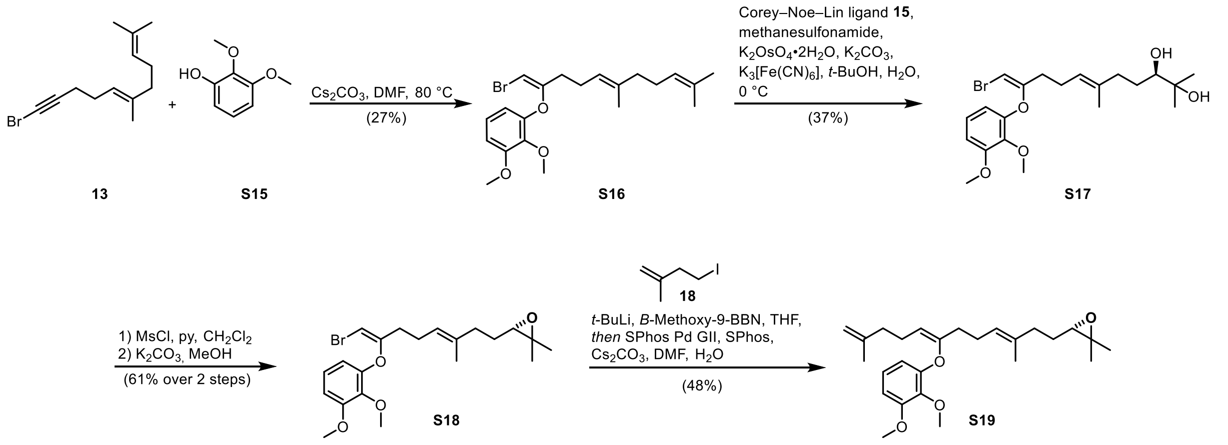

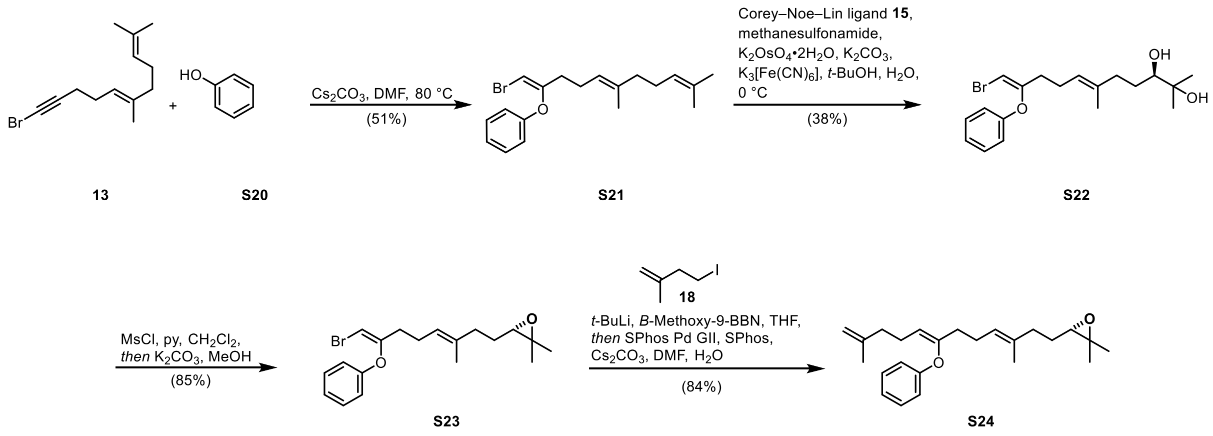
Br
HO
+
Cs2CO3, DMF, 80 °C
(51%)
Corey–Noe–Lin ligand 15,
methanesulfonamide,
K2OsO4•2H2O, K2CO3,
K3[Fe(CN)6], t-BuOH, H2O,
0 °C
(38%)
OH
OH
O
13
S20
S21
S22
MsCl, py, CH2Cl2,
then K2CO3, MeOH
(85%)
I
18
t-BuLi, B-Methoxy-9-BBN, THF,
then SPhos Pd GII, SPhos,
Cs2CO3, DMF, H2O
(84%)
S23
S24

2.2.1 (*Z*)-aryl enol ether **S6**

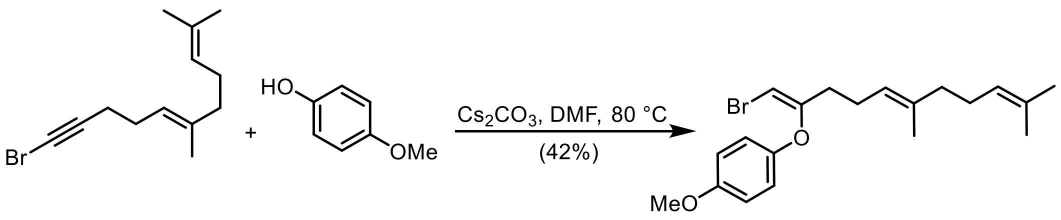

13 **S5** **S6**

To a mixture of phenol **S5** (4.86 g, 39.2 mmol, 10.0 equiv) and caesium carbonate (3.83 g, 11.8 mmol, 3.00 equiv) in dry dimethylformamide (5.0 mL) was added alkyne **13** (1.00 g, 3.92 mmol, 1 equiv) and the suspension was heated at 80 °C for 54 h. Water (20 mL) was added to the reaction mixture and extracted with diethyl ether (3 x 50 mL). The combined organic layers were dried over magnesium sulfate, the dried solution was filtered, and the filtrate was concentrated. Flash column chromatography on silica gel (0.3% grading to 1% diethyl ether in pentane) of the residue yielded enol ether **S6** (617 mg, 1.63 mmol, 42%) as a colorless oil.

TLC (1% diethyl ether in pentane): R_f: 0.32.

^{1}H NMR (400 MHz, Chloroform-*d*) δ 6.94 – 6.87 (m, 2H), 6.86 – 6.81 (m, 2H), 5.65 (s, 1H), 5.11 – 5.00 (m, 2H), 3.78 (s, 3H), 2.23 – 2.11 (m, 4H), 2.04 (q, *J* = 7.4 Hz, 2H), 1.99 – 1.93 (m, 2H), 1.69 – 1.65 (m, 3H), 1.59 (s, 3H), 1.55 (s, 3H).

^{13}C NMR (101 MHz, Benzene-d_6) δ 156.0, 155.8, 149.3, 136.6, 131.3, 124.8, 122.9, 118.7, 115.0, 90.7, 55.2, 40.1, 32.4, 27.1, 25.9, 25.4, 17.8, 16.0.

IR (ATR, neat) $\tilde{\nu}_{max}$: 2913 (w), 2854 (w), 1644 (w), 1501 (s), 1441 (m), 1376 (w), 1333 (w), 1296 (w), 1271 (w), 1245 (m), 1204 (s), 1180 (m), 1162 (w), 1133 (m), 1101 (w), 1036 (m), 1108 (w), 956 (w), 925 (w), 884 (w), 827 (m), 742 (m), 720 (w), 696 (w), 650 (w), 595 (w), 517 (m), 441 (w) cm^{-1}.

HRMS (ESI): calcd for $C_{20}H_{28}BrO_2^+$ [M+H]$^+$: 379.1267; found: 379.1242.

2.2.2 Dienediol **S7**

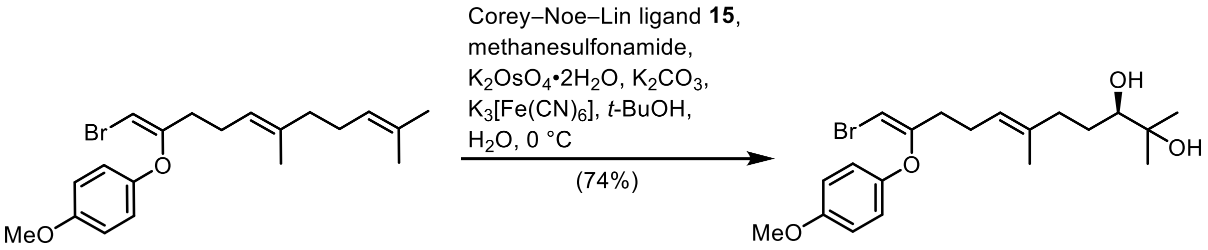

S6 **S7**

Potassium carbonate (2.91 g, 21.1 mmol, 4.00 equiv) and $K_3[Fe(CN)_6]$ (6.94 g, 21.1 mmol, 4.00 equiv) were ground to a fine powder in a mortar before adding Corey–Noe–Lin ligand **15** (120 mg, 105 µmol, 2.00 mol%), K_2OsO_4•$2H_2O$ (19.4 mg, 52.7 µmol, 1.00 mol%) and water (19.5 mL). After cooling the mixture to 0 °C, methanesulfonamide (502 mg, 5.27 mmol, 1 equiv) and a solution of alkene **S6** (2.00 g, 5.27 mmol, 1 equiv) in *tert*-butanol (19.5 mL) were added and the biphasic suspension was sonicated at 0 °C for 15 min before stirring vigorously at 0 °C for 17.5 h.[8] Sodium sulfite (6.65 g, 52.7 mmol, 10.0 equiv) was added, the cooling bath was removed and the slurry was allowed to reach 22 °C. After 20 min, 1 M aqueous solution of sodium hydroxide was added dropwise until all solids dissolved and the green mixture was extracted with ethyl acetate (4 x 50 mL). The combined organic layers were dried over sodium sulfate, the dried solution was filtered, and the filtrate was concentrated. The residue was purified by flash column chromatography on silica gel (2% grading to 35% ethyl acetate in cyclohexane) yielding diol **S7** (1.62 g, 3.91 mmol, 74%) as a yellowish oil along with starting material **S6** (80.0 mg, 211 µmol, 4%).[9]

TLC (40% ethyl acetate in cyclohexane): R_f: 0.26.

^{1}H NMR (400 MHz, Benzene-d_6) δ 6.87 – 6.81 (m, 2H), 6.70 – 6.63 (m, 2H), 5.46 (s, 1H), 5.15 – 5.07 (m, 1H), 3.34 – 3.29 (m, 4H), 3.18 (s, 1H), 2.95 (s, 1H), 2.34 (ddd, J = 14.2, 9.4, 5.2 Hz, 1H), 2.15 – 1.99 (m, 5H), 1.55 – 1.36 (m, 5H), 1.13 (s, 6H).

^{13}C NMR (101 MHz, Benzene-d_6) δ 155.9, 155.8, 149.2, 136.8, 123.1, 118.7, 115.1, 90.8, 78.3, 73.2, 55.3, 37.2, 32.3, 30.4, 26.7, 25.4, 23.6, 16.1.

IR (ATR, neat) $\tilde{\nu}_{max}$: 3411 (br, w), 2926 (w), 2854 (w), 1734 (w), 1644 (w), 1502 (s), 1464 (w), 1442 (w), 1382 (w), 1296 (w), 1246 (m), 1207 (s), 1180 (m), 1162 (w), 1135 (w), 1101 (w), 1076 (w), 1036 (m), 958 (w), 926 (w), 832 (w), 743 (w), 697 (w), 650 (w), 592 (w), 519 (w) cm^{-1}.

HRMS (ESI): calcd for $C_{20}H_{29}BrO_4Na^+$ $[M+Na]^+$: 435.1141; found: 435.1145.

$[\alpha]_D^{20}$ = +9.9 (c = 2.0, dichloromethane).

[8] Without sonication the ligand often agglutinated leading to poor yields.
[9] The reaction was stopped before complete conversion was reached to avoid over-oxidation.

2.2.3 Epoxide fragment **S8**

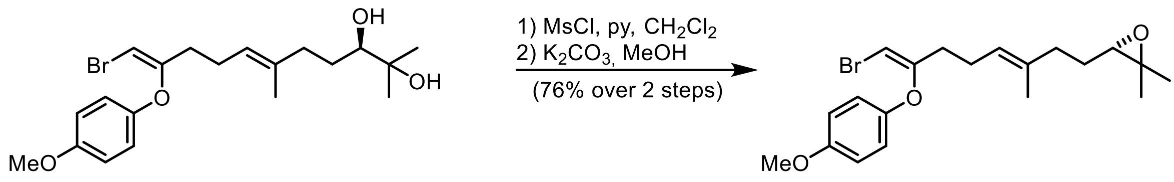

S7 **S8**

To a solution of diol **S7** (1.60 g, 3.87 mmol, 1 equiv) and pyridine (1.56 mL, 19.4 mmol, 5.00 equiv) in dry dichloromethane (16 mL) was added methanesulfonyl chloride[10] (449 µl, 5.8 mmol, 1.50 equiv) at 0 °C. The cooling bath was removed, and the reaction mixture was allowed to warm to 22 °C. After 19 h, water (40 mL) was added and the aqueous layer was extracted with dichloromethane (3 x 40 mL). The combined organic layers were dried over sodium sulfate, the dried solution was filtered, and the filtrate was concentrated. To the residue was added benzene (15 mL) and solution was concentrated.

The residue (assuming 1.90 g, 3.87 mmol, 1 equiv) was dissolved in dry methanol (50 mL) before potassium carbonate (1.07 g, 7.74 mmol, 2.00 equiv) was added.[11] After 2 h, water (50 mL) was added and the aqueous layer was extracted with dichloromethane (100 mL, then 2 x 50 mL). The combined organic layers were washed with saturated aqueous solution of sodium chloride (50 mL), the washed solution was dried over magnesium sulfate, the dried solution was filtered, and the filtrate was concentrated. The residue was purified by flash column chromatography on silica gel (5% grading to 7.5% ethyl acetate in pentane) yielding epoxide **S8** (1.16 g, 2.94 mmol, 76% over 2 steps) as a colorless oil.

TLC (20% ethyl acetate in cyclohexane): R_f: 0.46.

^{1}H NMR (400 MHz, Chloroform-*d*) δ 6.96 – 6.87 (m, 2H), 6.87 – 6.79 (m, 2H), 5.65 (s, 1H), 5.09 (t, *J* = 6.8 Hz, 1H), 3.78 (s, 3H), 2.67 (t, *J* = 6.2 Hz, 1H), 2.24 – 2.01 (m, 6H), 1.63 – 1.56 (m, 5H), 1.29 (s, 3H), 1.24 (s, 3H).

^{13}C NMR (101 MHz, Benzene-d_6) δ 156.0, 155.7, 149.3, 136.0, 123.3, 118.7, 115.1, 90.8, 63.4, 57.4, 55.2, 36.8, 32.3, 27.9, 25.3, 25.0, 18.9, 16.0.

IR (ATR, neat) $\tilde{\nu}_{max}$: 2958 (w), 2925 (w), 2836 (w), 1644 (w), 1502 (s), 1442 (w), 1378 (w), 1296 (w), 1246 (m), 1206 (s), 1180 (w), 1134 (w), 1102 (w), 1036 (m), 956 (w), 924 (w), 873 (w), 832 (m), 743 (w), 684 (w), 651 (w), 520 (w) cm^{-1}.

HRMS (ESI): calcd for $C_{20}H_{27}BrO_3Na^+$ $[M+Na]^+$: 417.1036; found: 417.1067.

$[\boldsymbol{\alpha}]_D^{20}$ = –2.2 (c = 2.5, dichloromethane).

[10] Methanesulfonyl chloride was freshly distilled from P_4O_{10} through a Vigreux column under a N_2 atmosphere.
[11] Potassium carbonate was ground to a fine powder in a mortar before use.

2.2.4 Cyclization precursor **S9**

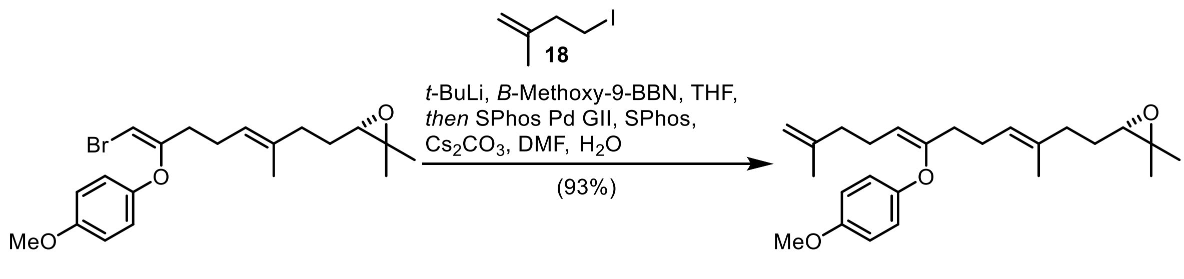

S8 **S9**

To a solution of iodide **18** (74.4 mg, 379 µmol, 1.50 equiv) and 9-methoxy-9-borabicyclo[3.3.1]nonane (1.00 M in hexanes, 885 µl, 885 µmol, 3.50 equiv) in degassed dry tetrahydrofuran (1.5 mL) was added *tert*-butyllithium (1.84 M in pentane, 619 µL, 1.14 mmol, 4.50 equiv) dropwise at −78 °C. The solution turned yellow and then colorless. After 5 min, the cooling bath was replaced by a water bath (22 °C) and the mixture was warmed to 22 °C. After 5 min, the reaction mixture was cooled to −78 °C. A degassed 9:1 mixture of dimethylformamide and water (100 µl) was added to the clear solution and the cooling bath was removed. The reaction mixture was allowed to warm to 22 °C.

A separate flask was charged with vinyl bromide **S8** (100 mg, 253 µmol, 1 equiv), caesium carbonate (165 mg, 506 µmol, 2.00 equiv), 2-dicyclohexylphosphino-2′,6′-dimethoxybiphenyl (5.2 mg, 13 µmol, 5.0 mol%), chloro(2-dicyclohexylphosphino-2′,6′-dimethoxy-1,1′-biphenyl)[2-(2′-amino-1,1′-biphenyl)]palladium(II) (9,1 mg, 13 µmol, 5.0 mol%) and a degassed 9:1 mixture of dimethylformamide and water (2.7 mL). To the yellow suspension was added the preformed boronate-species via cannulation and the biphasic mixture was heated at 40 °C. After 16 h, water (5 mL) was added and the mixture was extracted with ethyl acetate (4 x 15 mL). The combined organic layers were washed with water (3 x 10 mL) and saturated aqueous solution of sodium chloride (10 mL). The solution was dried over magnesium sulfate, the dried solution was filtered, and the filtrate was concentrated. The residue was purified by flash column chromatography on silica gel (3% ethyl acetate in cyclohexane) yielding cyclization precursor **S9** (90.0 mg, 234 µmol, 93%) as a colorless oil.

TLC (20% ethyl acetate in cyclohexane): R_f: 0.59.

^{1}H NMR (400 MHz, Benzene-*d*$_6$) δ 6.97 – 6.90 (m, 2H), 6.76 – 6.70 (m, 2H), 5.20 (dddd, J = 6.9, 5.6, 2.7, 1.4 Hz, 1H), 4.95 (dd, J = 7.5, 6.6 Hz, 1H), 4.84 – 4.77 (m, 2H), 3.31 (s, 3H), 2.55 (dd, J = 6.7, 5.7 Hz, 1H), 2.36 (q, J = 7.3 Hz, 2H), 2.27 – 2.16 (m, 4H), 2.14 – 1.97 (m, 4H), 1.63 – 1.50 (m, 8H), 1.14 (s, 3H), 1.10 (s, 3H).

^{13}C NMR (101 MHz, Benzene-*d*$_6$) δ 155.2, 151.7, 151.0, 145.3, 135.1, 124.3, 117.6, 115.1, 115.0, 110.7, 63.5, 57.4, 55.2, 38.0, 36.8, 32.7, 28.0, 26.0, 25.0, 23.8, 22.4, 18.9, 16.1.

IR (ATR, neat) $\tilde{\nu}_{max}$: 2924 (w), 2853 (w), 1683 (w), 1649 (w), 1503 (s), 1443 (w), 1377 (w), 1325 (w), 1296 (w), 1244 (w), 1208 (s), 1180 (w), 1131 (w), 1102 (w), 1038 (m), 975 (w), 886 (w), 828 (m), 733 (w), 678 (w), 521 (w) cm^{-1}.

HRMS (ESI): calcd for $C_{25}H_{36}O_3Na^+$ [M+Na]$^+$: 407.2557; found: 407.2587.

$[\alpha]_D^{20}$ = –2.0 (c = 1.8, dichloromethane).

2.2.5 (*Z*)-aryl enol ether **S11**

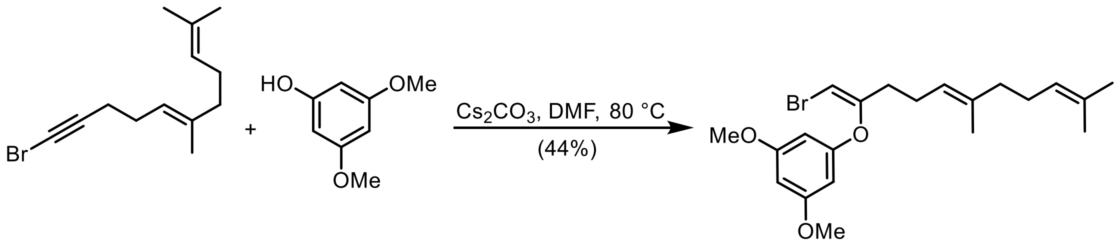

13 **S10** **S11**

To a suspension of caesium carbonate (3.83 g, 11.8 mmol, 3.00 equiv) in dry dimethylformamide (10 mL) was added phenol **S10** (6.04 g, 39.2 mmol, 10.0 equiv) and alkyne **13** (1.00 g, 3.92 mmol, 1 equiv). The reaction mixture was heated at 80 °C in a sealed tube for 89 h before water (20 mL) was added to the thick brown suspension. The mixture was extracted with diethyl ether (3 x 30 mL) and the combined organic layers were washed with water (40 mL), saturated aqueous solution of sodium chloride (40 mL) and the solution was dried over magnesium sulfate. The dried solution was filtered, and the filtrate was concentrated. Purification of the residue by flash column chromatography on silica gel (0.5% grading to 5.0% diethyl ether in pentane) yielded the title compound **S11** (700 mg, 1.71 mmol, 44%) as a colorless oil.

TLC (1% diethyl ether in cyclohexane): R_f: 0.27.

^{1}H NMR (400 MHz, Benzene-d_6) δ 6.29 (d, J = 2.2 Hz, 2H), 6.22 (t, J = 2.2 Hz, 1H), 5.46 (s, 1H), 5.19 – 5.08 (m, 1H), 5.02 (tdd, J = 5.6, 3.1, 1.4 Hz, 1H), 3.30 (s, 6H), 2.19 – 2.03 (m, 6H), 2.03 – 1.94 (m, 2H), 1.66 (d, J = 0.8 Hz, 3H), 1.54 (s, 3H), 1.48 (s, 3H).

^{13}C NMR (101 MHz, Benzene-d_6) δ 162.3, 157.5, 155.2, 136.7, 131.3, 124.8, 122.9, 96.0, 95.5, 92.5, 55.0, 40.1, 32.6, 27.1, 25.9, 25.4, 17.8, 16.1.

IR (ATR, neat) $\tilde{\nu}_{max}$: 2917 (w), 1596 (m), 1474 (w), 1443 (w), 1377 (w), 1264 (w), 1205 (m), 1152 (s), 1126 (w), 1064 (w), 823 (w), 678 (w) cm^{-1}.

HRMS (ESI): calcd for $C_{21}H_{30}BrO_3^+$ [M+H]$^+$: 409.1373; found: 409.1355.

2.2.6 Dienediol **S12**

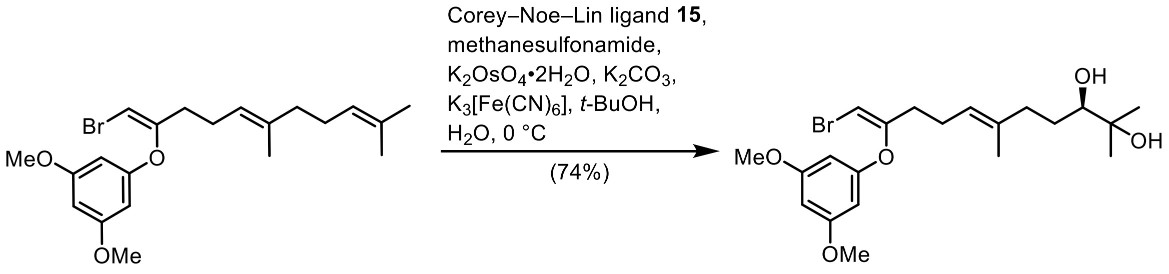

Potassium carbonate (1.30 g, 9.43 mmol, 4.00 equiv) and $K_3[Fe(CN)_6]$ (3.10 g, 9.43 mmol, 4.00 equiv) were ground to a fine powder in a mortar before adding Corey–Noe–Lin ligand **15** (53.7 mg, 47.1 µmol, 2.00 mol%), K_2OsO_4•$2H_2O$ (8.7 mg, 24 µmol, 1.0 mol%) and water (8.7 mL). After cooling the mixture to 0 °C, methanesulfonamide (224 mg, 2.36 mmol, 1 equiv) and a solution of alkene **S11** (965 mg, 2.36 mmol, 1 equiv) in *tert*-butanol (8.7 mL) were added and the biphasic suspension was sonicated at 0 °C for 15 min before stirring vigorously at 0 °C for 24 h.[12] Sodium sulfite (2.97 g, 23.6 mmol, 10.0 equiv) was added and the cooling bath was removed. The slurry was allowed to reach 22 °C. After 9 h, 1 M aqueous solution of sodium hydroxide was added dropwise until all solids dissolved and the green mixture was extracted with ethyl acetate (4 x 50 mL). The combined organic layers were dried over sodium sulfate, the dried solution was filtered, and the filtrate was concentrated. The residue purified by flash column chromatography on silica gel (2% grading to 35% ethyl acetate in cyclohexane) yielding diol **S12** (770 mg, 1.74 mmol, 74%) as a yellowish oil along with starting material S**11** (15.0 mg, 36.6 µmol, 2%).[13]

TLC (50% ethyl acetate in cyclohexanes): R_f: 0.34.

^{1}H NMR (400 MHz, Benzene-d_6) δ 6.36 (d, J = 2.2 Hz, 2H), 6.26 (t, J = 2.2 Hz, 1H), 5.45 (s, 1H), 5.16 – 5.04 (m, 1H), 3.27 (s, 6H), 3.22 (dd, J = 10.3, 2.2 Hz, 1H), 2.27 (ddd, J = 14.0, 9.0, 5.2 Hz, 1H), 2.18 (s, 1H), 2.12 – 2.07 (m, 4H), 2.03 (dd, J = 14.2, 8.0 Hz, 1H), 1.74 (s, 1H), 1.50 (d, J = 1.4 Hz, 3H), 1.48 – 1.32 (m, 2H), 1.06 (s, 3H), 1.05 (s, 3H).

^{13}C NMR (101 MHz, Benzene-d_6) δ 162.4, 157.5, 155.1, 136.8, 123.2, 96.0, 95.5, 92.7, 78.1, 72.8, 55.0, 37.1, 32.5, 30.2, 26.5, 25.4, 23.6, 16.0.

IR (ATR, neat) $\tilde{\nu}_{max}$: 3412 (br, w), 2965 (w), 2934 (w), 2840 (w), 1596 (s), 1473 (m), 1383 (w), 1330 (w), 1267 (w), 1204 (m), 1151 (s), 1063 (m), 990 (w), 930 (w), 892 (w), 824 (w), 761 (w), 735 (w), 677 (w) cm^{-1}.

HRMS (ESI): calcd for $C_{21}H_{32}BrO_5^+$ [M+H]$^+$: 443.1428; found: 443.1416.

$[\boldsymbol{\alpha}]_{\boldsymbol{D}}^{\mathbf{20}}$ = +5.4 (c = 1.5, dichloromethane).

12 Without sonication the ligand often agglutinated leading to poor yields.
13 The reaction was stopped before complete conversion was reached to avoid over-oxidation.

2.2.7 Epoxide fragment **S13**

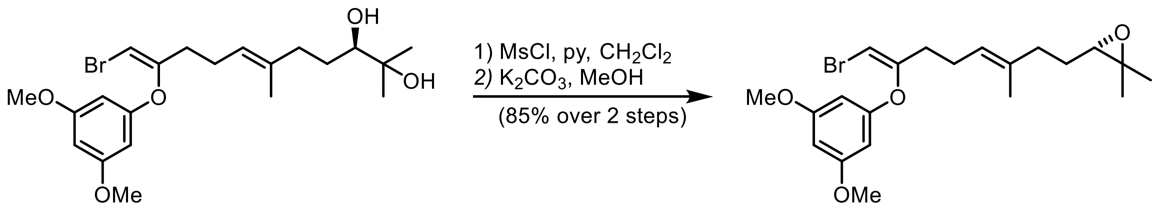

S12 **S13**

To a solution of diol **S12** (408 mg, 920 µmol, 1 equiv) and pyridine (371 µL, 4.60 mmol, 5.00 equiv) in dry dichloromethane (4.0 mL) was added methanesulfonyl chloride[14] (107 µl, 1.38 mmol, 1.50 equiv) at 0 °C. The cooling bath was removed and the reaction mixture was allowed to warm to 22 °C. After 23 h, water (4.0 mL) was added and the aqueous layer was extracted with dichloromethane (3 x 4 mL). The combined organic layers were dried over sodium sulfate, the dried solution was filtered, and the filtrate was concentrated. To the residue was added benzene (15 mL) and the solution was concentrated.

The residue (assuming 480 mg, 920 µmol, 1 equiv) was dissolved in dry methanol (10 mL) before potassium carbonate (254 mg, 1.84 mmol, 2.00 equiv) was added.[15] After 1.5 h, water (10 mL) was added and the mixture was extracted with dichloromethane (20 mL, then 2 x 10 mL). The combined organic layers were washed with saturated aqueous solution of sodium chloride (10 mL), the solution was dried over magnesium sulfate, the dried solution was filtered, and the filtrate was concentrated. The residue was purified by flash column chromatography on silica gel (5% ethyl acetate in pentane) yielding epoxide **S13** (334 mg, 785 µmol, 85% over 2 steps) as a colorless oil.

TLC (10% ethyl acetate in cyclohexane): R_f: 0.32.

^{1}H NMR (400 MHz, Benzene-d_6) δ 6.35 (d, J = 2.2 Hz, 2H), 6.26 (t, J = 2.2 Hz, 1H), 5.46 (s, 1H), 5.06 – 4.98 (m, 1H), 3.27 (s, 6H), 2.56 – 2.48 (m, 1H), 2.11 – 1.93 (m, 6H), 1.54 – 1.46 (m, 2H), 1.45 (d, J = 1.4 Hz, 3H), 1.15 (s, 3H), 1.09 (s, 3H).

^{13}C NMR (101 MHz, Benzene-d_6) δ 162.4, 157.5, 155.1, 136.1, 123.3, 96.0, 95.5, 92.6, 63.4, 57.4, 55.0, 36.8, 32.5, 27.9, 25.4, 25.0, 18.9, 16.0.

IR (ATR, neat) $\tilde{\nu}_{max}$: 2959 (w), 2928 (w), 2840 (w), 1596 (m), 1475 (w), 1377 (w), 1324 (w), 1267 (w), 1206 (m), 1153 (s), 1063 (w), 988 (w), 930 (w), 873 (w), 824 (w), 771 (w), 735 (w), 679 (w) cm^{-1}.

HRMS (ESI): calcd for $C_{21}H_{30}BrO_4^+$ $[M+H]^+$: 425.1322; found: 425.1308.

$[\alpha]_D^{20}$ = –2.9 (c = 2.0, dichloromethane).

[14] Methanesulfonyl chloride was freshly distilled from P_4O_{10} through a Vigreux column under a N_2 atmosphere.
[15] Potassium carbonate was ground to a fine powder in a mortar before use.

2.2.8 Cyclization precursor **S14**

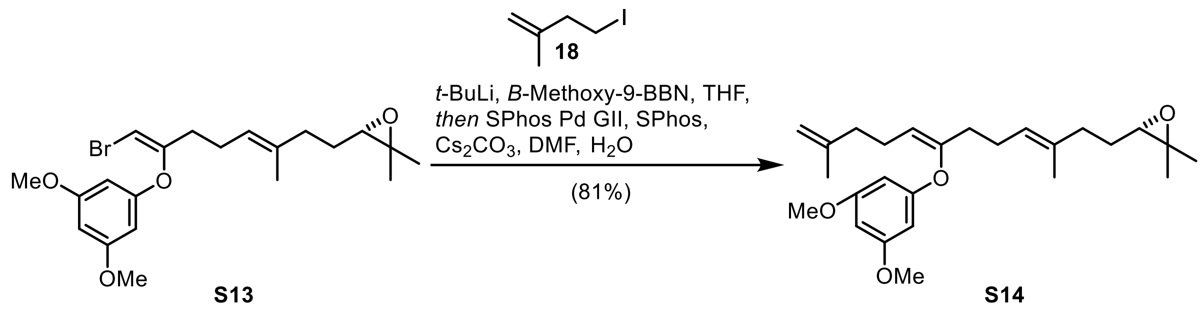

To a solution of iodide **18** (39.4 mg, 201 µmol, 1.50 equiv) and 9-methoxy-9-borabicyclo[3.3.1]nonane (1.00 M in hexanes, 469 µl, 469 µmol, 3.50 equiv) in degassed dry tetrahydrofuran (875 µL) was added *tert*-butyllithium (1.60 M in pentane, 377 µL, 603 µmol, 4.50 equiv) dropwise at −78 °C. The solution turned yellow and then colorless. After 5 min, the cooling bath was replaced by a water bath (22 °C) and the mixture was warmed to 22 °C. After 5 min the reaction mixture was cooled to −78 °C. A degassed 9:1 mixture of dimethylformamide and water (50 µl) was added to the clear solution and the cooling bath was removed. The reaction mixture was allowed to warm to 22 °C.

A separate flask was charged with vinyl bromide **S13** (57.0 mg, 134 µmol, 1 equiv), caesium carbonate (87.3 mg, 268 µmol, 2.00 equiv), 2-dicyclohexylphosphino-2′,6′-dimethoxybiphenyl (2.8 mg, 7.0 µmol, 5.0 mol%), chloro(2-dicyclohexylphosphino-2′,6′-dimethoxy-1,1′-biphenyl)[2-(2′-amino-1,1′-biphenyl)]palladium(II) (4.8 mg, 7.0 µmol, 5.0 mol%) and a degassed 9:1 mixture of dimethylformamide and water (1.6 mL). To the yellow suspension was added the preformed boronate-species via cannulation and the biphasic mixture was heated at 40 °C. After 3.5 h, water (5 mL) was added and the mixture was extracted with ethyl acetate (4 x 15 mL). The combined organic layers were washed with water (3 x 10 mL) and saturated aqueous solution of sodium chloride (10 mL). The solution was dried over magnesium sulfate, the dried solution was filtered, and the filtrate was concentrated. The residue was purified by flash column chromatography on silica gel (3% ethyl acetate in pentane) yielding cyclization precursor **14** (45.1 mg, 109 µmol, 81%) as a colorless oil.

TLC (20% ethyl acetate in cyclohexane): R_f: 0.53.

^{1}H NMR (400 MHz, Benzene-d_6) δ 6.44 (d, *J* = 2.2 Hz, 2H), 6.27 (t, *J* = 2.2 Hz, 1H), 5.24 – 5.17 (m, 1H), 4.98 (t, *J* = 7.1 Hz, 1H), 4.79 (s, 2H), 3.31 (s, 6H), 2.58 – 2.53 (m, 1H), 2.33 (q, *J* = 7.3 Hz, 2H), 2.30 – 2.22 (m, 4H), 2.15 – 1.97 (m, 4H), 1.61 – 1.50 (m, 8H), 1.14 (s, 3H), 1.10 (s, 3H).

^{13}C NMR (101 MHz, Benzene-d_6) δ 162.5, 159.3, 151.1, 145.2, 135.2, 124.3, 116.2, 110.8, 95.4, 94.5, 63.5, 57.4, 54.9, 37.8, 36.8, 33.0, 28.0, 26.0, 25.0, 23.9, 22.3, 18.9, 16.1.

IR (ATR, neat) $\tilde{\nu}_{max}$: 3071 (w), 2924 (w), 2853 (w), 1685 (w), 1594 (s), 1459 (m), 1377 (w), 1324 (w), 1239 (w), 1204 (m), 1145 (s), 1057 (m), 1004 (w), 930 (w), 886 (w), 824 (m), 753 (w), 728 (w), 682 (w) cm^{-1}.

HRMS (ESI): calcd for $C_{26}H_{38}O_4Na^+$ $[M+Na]^+$: 437.2662; found: 437.2650.

$[\alpha]_D^{20}$ = –1.9 (c = 0.74, dichloromethane).

2.2.9 (*Z*)-aryl enol ether **S16**

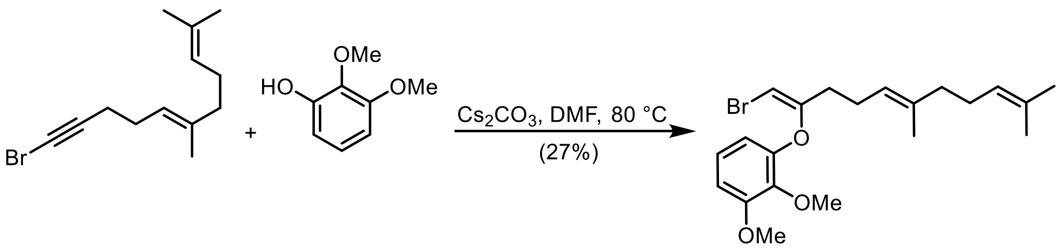

13 **S15** **S16**

To a suspension of caesium carbonate (8.70 g, 26.7 mmol, 3.00 equiv) in dry dimethylformamide (5.5 mL) was added phenol **S15** (4.12 g, 26.7 mmol, 3.00 equiv) and alkyne **13** (2.27 g, 8.90 mmol, 1 equiv). The reaction mixture was heated at 80 °C in a sealed tube for 24 h before water (100 mL) was added to the thick brown suspension. The mixture was extracted with diethyl ether (4 x 100 mL) and the combined organic layers were washed with saturated aqueous solution of sodium chloride (100 mL), the solution was dried over magnesium sulfate, the dried solution was filtered, and the filtrate was concentrated. Purification of the residue by flash column chromatography on silica gel (3% grading to 4% diethyl ether in pentane) yielded the title compound **S16** (990 mg, 2.42 mmol, 27%) as a colorless oil.

TLC (10% ethyl acetate in pentane): R_f: 0.78.

^{1}H NMR (600 MHz, Benzene-d_6) δ 6.73 (t, *J* = 8.3 Hz, 1H), 6.62 (dd, *J* = 8.3, 1.4 Hz, 1H), 6.31 (dd, *J* = 8.3, 1.4 Hz, 1H), 5.35 (s, 1H), 5.17 (ddt, *J* = 8.5, 7.0, 1.5 Hz, 1H), 5.03 – 4.96 (m, 1H), 3.84 (s, 3H), 3.29 (s, 3H), 2.11 – 2.06 (m, 6H), 1.97 (t, *J* = 7.7 Hz, 2H), 1.67 (d, *J* = 1.6 Hz, 3H), 1.54 (s, 3H), 1.47 (s, 3H).

^{13}C NMR (151 MHz, Benzene-d_6) δ 156.2, 154.8, 149.3, 141.0, 136.5, 131.2, 124.8, 123.7, 123.0, 111.9, 108.0, 88.5, 61.0, 55.6, 40.1, 32.5, 27.1, 25.9, 25.4, 17.8, 16.0.

IR (ATR, neat) $\tilde{\nu}_{max}$: 3095 (w), 2965 (w), 2930 (m), 2853 (w), 1647 (w), 1594 (m), 1471 (s), 1439 (m), 1375 (w), 1277 (m), 1247 (s), 1171 (w), 1141 (m), 1090 (s), 1008 (m), 886 (w), 837 (w), 790 (w), 739 (m), 653 (w) cm^{-1}.

HRMS (ESI): calcd for $C_{21}H_{29}BrO_3Na^+$ [M+Na]$^+$: 431.1192; found: 431.1186.

2.2.10 Dienediol **S17**

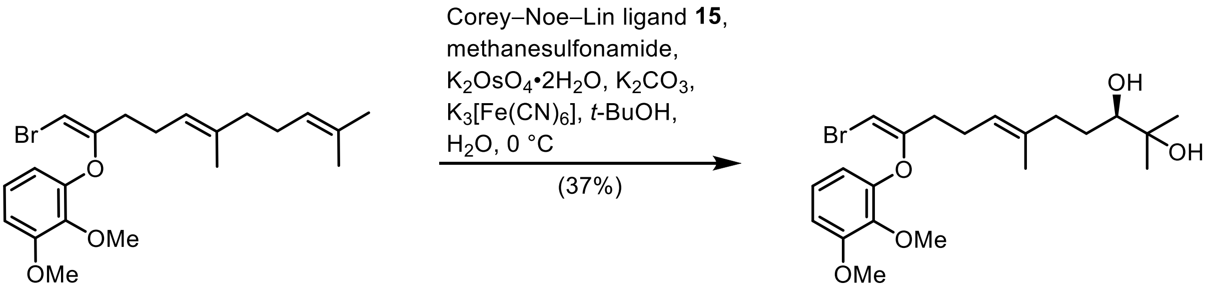

Potassium carbonate (689 mg, 4.98 mmol, 4.00 equiv) and $K_3[Fe(CN)_6]$ (1.64 g, 4.98 mmol, 4.00 equiv) were ground to a fine powder in a mortar before adding Corey–Noe–Lin ligand **15** (28.4 mg, 24.9 µmol, 2.00 mol%), K_2OsO_4•$2H_2O$ (4.6 mg, 13 µmol, 1.0 mol%) and water (4.0 mL). After cooling the mixture to 0 °C, methanesulfonamide (119 mg, 1.25 mmol, 1 equiv) and a solution of alkene **S16** (510 mg, 1.25 mmol, 1 equiv) in *tert*-butanol (4.0 mL) were added. The biphasic suspension was sonicated at 0 °C for 15 min before stirring vigorously at 0 °C for 38 h.[16] Sodium sulfite (1.57 g, 12.5 mmol, 10.0 equiv) was added and the cooling bath was removed. The slurry was allowed to warm to 22 °C. After 30 min, 2 M aqueous solution of sodium hydroxide was added dropwise until all solids dissolved and the green mixture was extracted with ethyl acetate (4 x 50 mL). The combined organic layers were dried over sodium sulfate, the dried solution was filtered, and the filtrate was concentrated. The residue was purified by flash column chromatography on silica gel (2% grading to 35% ethyl acetate in cyclohexane) yielding diol **S17** (208 mg, 470 mmol, 38%) as a yellowish oil along with starting material **S16** (281 mg, 686 µmol, 55%).[17]

TLC (80% ethyl acetate in pentane): R_f: 0.55.

^{1}H NMR (400 MHz, Benzene-*d*$_6$) δ 6.73 (t, *J* = 8.3 Hz, 1H), 6.61 (dd, *J* = 8.3, 1.4 Hz, 1H), 6.31 (dd, *J* = 8.3, 1.4 Hz, 1H), 5.34 (s, 1H), 5.10 – 4.99 (m, 1H), 3.84 (s, 3H), 3.29 (s, 3H), 3.17 (ddd, *J* = 10.4, 4.0, 2.1 Hz, 1H), 2.21 (ddd, *J* = 14.3, 9.1, 5.2 Hz, 1H), 2.13 – 2.04 (m, 4H), 2.04 – 1.95 (m, 1H), 1.90 (d, *J* = 4.2 Hz, 1H), 1.48 (d, *J* = 1.4 Hz, 4H), 1.45 – 1.39 (m, 1H), 1.32 (dddd, *J* = 13.8, 10.3, 8.9, 5.3 Hz, 1H), 1.03 (s, 3H), 1.03 (s, 3H).

^{13}C NMR (101 MHz, Benzene-*d*$_6$) δ 156.2, 154.8, 149.2, 140.9, 136.7, 123.7, 123.3, 111.8, 108.0, 88.7, 78.1, 72.6, 61.0, 55.6, 37.0, 32.4, 30.1, 26.5, 25.4, 23.6, 15.9.

IR (ATR, neat) $\tilde{\nu}_{max}$: 3426 (w), 3099 (w), 2932 (m), 2855 (w), 1647 (w), 1594 (m), 1471 (s), 1381 (w), 1277 (m), 1247 (s), 1141 (m), 1086 (s), 1006 (m), 935 (w), 884 (w), 794 (w), 739 (w) cm^{-1}.

HRMS (ESI): calcd for $C_{21}H_{31}BrO_5Na^+$ $[M+Na]^+$: 465.1247; found: 465.1246.

$[\alpha]_D^{20}$ = +8.6 (c = 0.57, dichloromethane).

[16] Without sonication the ligand often agglutinated leading to poor yields.
[17] The reaction was stopped before complete conversion was reached to avoid over-oxidation.

2.2.11 Epoxide fragment **S18**

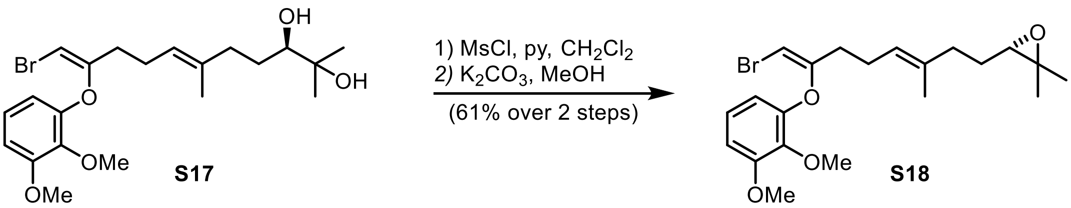

To a solution of diol **S17** (171 mg, 386 µmol, 1 equiv) and pyridine (155 µL, 1.93 mmol, 5.00 equiv) in dry dichloromethane (1.7 mL) was added methanesulfonyl chloride[18] (45.1 µl, 579 µmol, 1.50 equiv) at 0 °C. The cooling bath was removed, and the reaction mixture was allowed to warm to 22 °C. After 17 h, water (2 mL) was added and the aqueous layer was extracted with dichloromethane (3 x 4 mL). The combined organic layers were dried over sodium sulfate, the dried solution was filtered, and the filtrate was concentrated. To the residue was added benzene (15 mL) and the solution was concentrated.

The residue (assuming 201 mg, 386 µmol, 1 equiv) was dissolved in dry methanol (3.9 mL) before potassium carbonate (107 mg, 771 µmol, 2.00 equiv) was added.[19] After 1.5 h, water (5 mL) was added and the aqueous layer was extracted with dichloromethane (3 x 10 mL). The combined organic layers were dried over magnesium sulfate, the dried solution was filtered, and the filtrate was concentrated. The residue was purified by flash column chromatography on silica gel (5% ethyl acetate in cyclohexane) yielding epoxide **S18** (100 mg, 235 µmol, 61% over 2 steps) as a colorless oil.

TLC (20% ethyl acetate in cyclohexane): R_f: 0.42.

^{1}H NMR (400 MHz, Benzene-*d*$_6$) δ 6.74 (t, *J* = 8.3 Hz, 1H), 6.61 (dd, *J* = 8.3, 1.5 Hz, 1H), 6.32 (dd, *J* = 8.3, 1.4 Hz, 1H), 5.36 (s, 1H), 4.96 (qt, *J* = 4.4, 2.1 Hz, 1H), 3.83 (s, 3H), 3.30 (s, 3H), 2.50 (dd, *J* = 6.7, 5.6 Hz, 1H), 2.06 (d, *J* = 3.2 Hz, 4H), 2.03 – 1.89 (m, 2H), 1.55 – 1.41 (m, 5H), 1.14 (s, 3H), 1.08 (s, 3H).

^{13}C NMR (101 MHz, Benzene-*d*$_6$) δ 156.1, 154.8, 149.2, 141.0, 135.9, 123.7, 123.4, 111.9, 108.0, 88.5, 63.5, 61.0, 57.4, 55.7, 36.8, 32.4, 27.9, 25.4, 25.0, 18.9, 15.9.

IR (ATR, neat) $\tilde{\nu}_{max}$: 3095 (w), 2934 (w), 2836 (w), 1647 (w), 1592 (m), 1469 (s), 1377 (w), 1277 (m), 1245 (s), 1171 (m), 1139 (m), 1086 (s), 1006 (m), 871 (w), 775 (w), 737 (m) cm^{-1}.

HRMS (ESI): calcd for $C_{21}H_{29}BrO_4Na^+$ $[M+Na]^+$: 447.1141; found: 447.1111.

$[\alpha]_D^{20}$ = –2.2 (c = 1.77, dichloromethane).

[18] Methanesulfonyl chloride was freshly distilled from P_4O_{10} through a Vigreux column under a N_2 atmosphere.
[19] Potassium carbonate was ground to a fine powder in a mortar before use.

2.2.12 Cyclization precursor **S19**

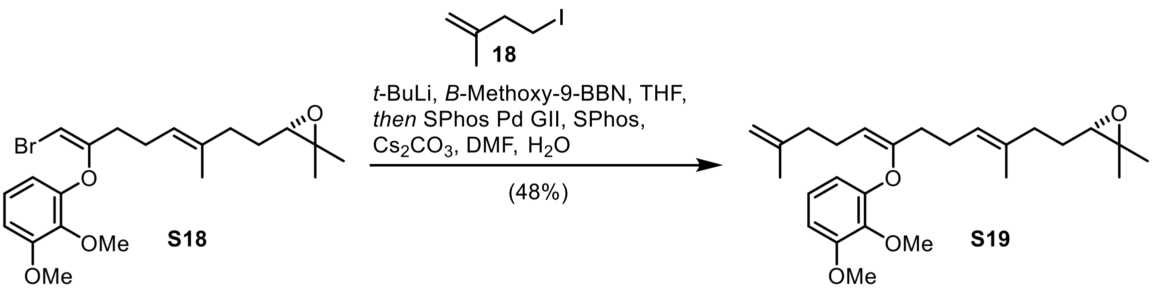

To a solution of iodide **18** (34.6 mg, 176 µmol, 1.50 equiv) and 9-methoxy-9-borabicyclo[3.3.1]nonane (1.00 M in hexanes, 411 µl, 411 µmol, 3.50 equiv) in degassed dry tetrahydrofuran (718 µL) was added *tert*-butyllithium (1.84 M in pentane, 287 µL, 529 µmol, 4.50 equiv) dropwise at −78 °C. The solution turned yellow and then colorless. After 5 min, the cooling bath was replaced by a water bath (22 °C) and the reaction mixture was warmed to 22 °C. After 5 min the reaction mixture was cooled to −78 °C. A degassed 9:1 mixture of dimethylformamide and water (50 µl) was added to the clear solution and the cooling bath was removed. The reaction mixture was allowed to warm to 22 °C.

A separate flask was charged with vinyl bromide **S18** (50.0 mg, 118 µmol, 1 equiv), caesium carbonate (76.6 mg, 235 µmol, 2.00 equiv), 2-dicyclohexylphosphino-2′,6′-dimethoxybiphenyl (2.4 mg, 5.9 µmol, 5.0 mol%), chloro(2-dicyclohexylphosphino-2′,6′-dimethoxy-1,1′-biphenyl)[2-(2′-amino-1,1′-biphenyl)]palladium(II) (4.2 mg, 5.9 µmol, 5.0 mol%) and a degassed 9:1 mixture of dimethylformamide and water (1.2 mL). To the yellow suspension was added the preformed boron-species via cannulation and the biphasic mixture was heated at 40 °C. After 18 h, water (5 mL) was added and the mixture was extracted with ethyl acetate (3 x 10 mL). The combined organic layers were dried over magnesium sulfate, the dried solution was filtered, and the filtrate was concentrated. The residue was purified by flash column chromatography on silica gel (5% ethyl acetate in pentane) yielding cyclization precursor **S19** (23.5 mg, 56.7 µmol, 48%) as a colorless oil.

TLC (20% ethyl acetate in cyclohexane): R_f: 0.49.

^{1}H NMR (400 MHz, Benzene-d_6) δ 6.81 (t, J = 8.3 Hz, 1H), 6.68 (dd, J = 8.3, 1.5 Hz, 1H), 6.34 (dd, J = 8.3, 1.4 Hz, 1H), 5.22 – 5.12 (m, 1H), 4.95 (t, J = 7.1 Hz, 1H), 4.84 – 4.77 (m, 2H), 3.88 (s, 3H), 3.36 (s, 3H), 2.54 (dd, J = 6.6, 5.8 Hz, 1H), 2.39 – 2.32 (m, 2H), 2.30 – 2.19 (m, 4H), 2.11 – 1.95 (m, 4H), 1.62 – 1.49 (m, 8H), 1.14 (s, 3H), 1.09 (s, 3H).

^{13}C NMR (101 MHz, Benzene-d_6) δ 154.9, 151.7, 151.0, 145.4, 140.4, 135.1, 124.3, 123.5, 114.6, 110.7, 109.7, 106.8, 63.5, 60.7, 57.4, 55.7, 38.0, 36.8, 32.9, 28.0, 26.0, 25.0, 23.8, 22.4, 18.9, 16.1.

IR (ATR, neat) $\tilde{\nu}_{max}$: 3073 (w), 2926 (m), 2853 (w), 1685 (w), 1649 (w), 1594 (m), 1471 (s), 1377 (w), 1286 (m), 1247 (s), 1171 (w), 1135 (m), 1094 (s), 1010 (m), 886 (w), 777 (w), 737 (w) cm^{-1}.

HRMS (ESI): calcd for $C_{26}H_{38}O_4^+$ [M+Na]$^+$: 437.2662; found: 437.2651.

$[\alpha]_D^{20}$ = –1.2 (c = 0.85, dichloromethane).

2.2.13 (*Z*)-aryl enol ether **S21**

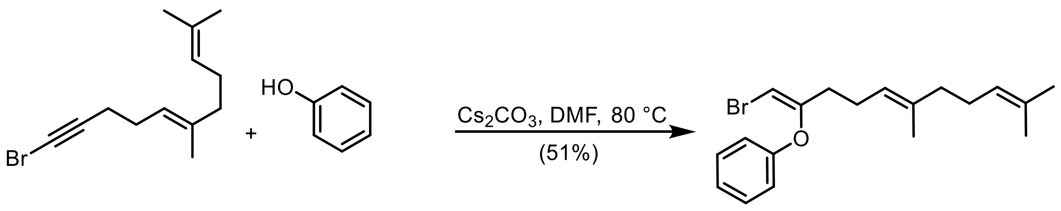

13 **S20** **S21**

To a suspension of caesium carbonate (14.0 g, 42.9 mmol, 3.00 equiv) in dry dimethylformamide (38 mL) was added phenol **S20** (10.8 g, 114 mmol, 8.00 equiv) and alkyne **13** (3.65 g, 14.3 mmol, 1 equiv). The reaction mixture was heated at 80 °C in a sealed tube for 65 h before water (50 mL) was added to the thick brown suspension. The mixture was extracted with diethyl ether (4 x 50 mL) and the combined organic layers were washed with water (100 mL) and saturated aqueous solution of sodium chloride (2 x 50 mL). The solution was dried over magnesium sulfate, the dried solution was filtered, and the filtrate was concentrated. Purification of the residue by flash column chromatography on silica gel (1% diethyl ether in pentane) yielded the title compound **S21** (2.54 g, 7.27 mmol, 51%) as a yellowish oil.

TLC (1% diethyl ether in pentane): R_f: 0.67.

^{1}H NMR (400 MHz, Benzene-*d*$_6$) δ 7.08 – 7.00 (m, 2H), 6.95 – 6.86 (m, 2H), 6.86 – 6.77 (m, 1H), 5.43 (s, 1H), 5.19 (ddp, *J* = 6.8, 5.5, 1.3 Hz, 1H), 5.01 (ddt, *J* = 6.9, 5.6, 1.3 Hz, 1H), 2.11 (q, *J* = 7.1 Hz, 2H), 2.07 – 1.96 (m, 6H), 1.69 (d, *J* = 1.4 Hz, 3H), 1.56 (s, 3H), 1.46 (s, 3H).

^{13}C NMR (101 MHz, Benzene-*d*$_6$) δ 155.7, 155.2, 136.6, 131.3, 129.9, 124.8, 122.9, 122.8, 117.2, 92.2, 40.1, 32.5, 27.1, 25.9, 25.2, 17.8, 16.0.

IR (ATR, neat) $\tilde{\nu}_{max}$: 2965 (w), 2914 (w), 2855 (w), 1645 (w), 1592 (m), 1490 (s), 1445 (w), 1377 (w), 1332 (w), 1267 (w), 1214 (s), 1163 (m), 1132 (m), 1073 (w), 1024 (w), 955 (w), 922 (w), 890 (w), 830 (w), 749 (s), 690 (s), 565 (w), 496 (m) cm^{-1}.

HRMS (ESI): calcd for $C_{19}H_{25}BrONa^+$ $[M+Na]^+$: 471.0981; found: 471.0973.

2.2.14 Dienediol **S22**

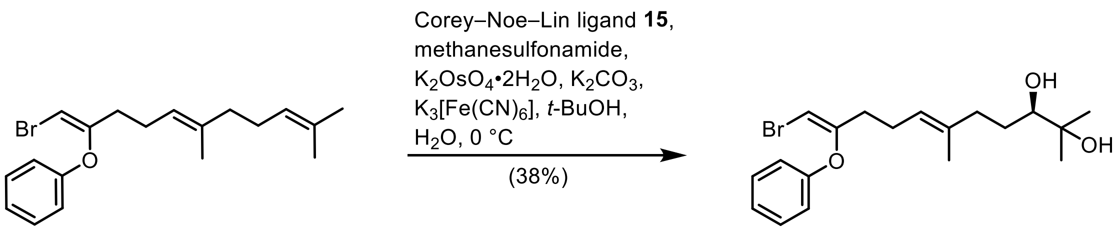

S21 **S22**

Potassium carbonate (6.12 g, 44.3 mmol, 4.00 equiv) and $K_3[Fe(CN)_6]$ (14.6 g, 44.1 mmol, 4.00 equiv) were ground to a fine powder in a mortar before adding Corey–Noe–Lin ligand **15** (315 mg, 277 µmol, 2.50 mol%), K_2OsO_4•$2H_2O$ (40.8 mg, 111 µmol, 1.00 mol%) and water (30 mL). After cooling the mixture to 0 °C, methanesulfonamide (1.05 g, 11.1 mmol, 1 equiv) and a solution of alkene **S21** (3.87 g, 11.1 mmol, 1 equiv) in *tert*-butanol (30 mL) were added and the biphasic suspension was sonicated at 0 °C for 15 min before stirring vigorously at 0 °C for 46 h.[20] Sodium sulfite (14.0 g, 111 mmol, 10.0 equiv) was added and the cooling bath was removed. The slurry was allowed to warm to 22 °C. After 30 min, 1 M aqueous solution of sodium hydroxide was added dropwise until all solids dissolved and the green mixture was extracted with ethyl acetate (4 x 100 mL). The combined organic layers were dried over sodium sulfate, the dried solution was filtered, and the filtrate was concentrated. The residue purified by flash column chromatography on silica gel (2% grading to 30% ethyl acetate in cyclohexane) yielding diol **S22** (1.60 g, 4.19 mmol, 38%) as a yellowish oil along with starting material **S21** (1.27 g, 3.65 mmol, 33%).[21]

TLC (50% ethyl acetate in cyclohexane): R_f: 0.38.

^{1}H NMR (400 MHz, Benzene-d_6) δ 7.08 – 7.02 (m, 2H), 6.94 – 6.89 (m, 2H), 6.85 – 6.79 (m, 1H), 5.43 (s, 1H), 5.09 – 5.00 (m, 1H), 3.18 (ddd, *J* = 10.4, 3.9, 2.1 Hz, 1H), 2.25 (ddd, *J* = 14.3, 9.2, 5.3 Hz, 1H), 2.06 – 1.96 (m, 5H), 1.87 (d, *J* = 4.1 Hz, 1H), 1.49 – 1.41 (m, 4H), 1.37 – 1.29 (m, 1H), 1.03 (s, 3H), 1.02 (s, 3H).

^{13}C NMR (101 MHz, Benzene-d_6) δ 155.6, 155.1, 136.9, 129.9, 123.1, 122.9, 117.2, 92.3, 78.1, 72.6, 37.1, 32.4, 30.1, 26.5, 25.2, 23.6, 16.0.

IR (ATR, neat) $\tilde{\nu}_{max}$: 3465 (br, w), 2977 (w), 2928 (w), 2855 (w), 1736 (m), 1647 (w), 1592 (m), 1490 (m), 1447 (w), 1373 (m), 1214 (s), 1163 (m), 1135 (m), 1045 (s), 924 (m), 892 (w), 847 (w), 751 (s), 692 (m), 635 (w), 608 (m), 582 (w), 498 (m) cm^{-1}.

HRMS (ESI): calcd for $C_{19}H_{27}BrO_3Na^+$ $[M+Na]^+$: 405.1036; found: 405.1025.

$[\boldsymbol{\alpha}]_{\boldsymbol{D}}^{\mathbf{20}}$ = +8.1 (c = 0.92, dichloromethane).

---

[20] Without sonication the ligand often agglutinated leading to poor yields.
[21] The reaction was stopped before complete conversion was reached to avoid over-oxidation.

2.2.15 Epoxide fragment **S23**

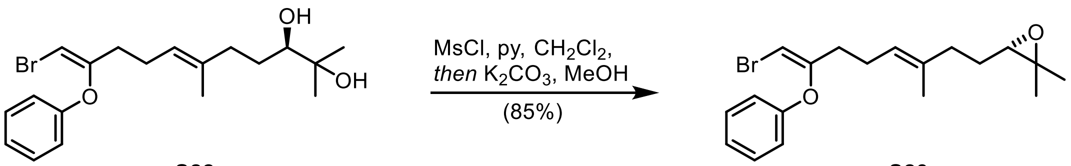

To a solution of diol **S22** (1.35 g, 3.51 mmol, 1 equiv) and dry pyridine (1.42 mL, 17.6 mmol, 5.00 equiv) in dry dichloromethane (15 mL) was added methanesulfonyl chloride (408 µl, 5.27 mmol, 1.50 equiv) at 0 °C.[22] The cooling bath was removed and the reaction mixture was allowed to warm to 22 °C. After 15.5 h, the solution was cooled to 0 °C, methanesulfonyl chloride (118 µl, 1.51 mmol, 0.300 equiv) was added and the cooling bath was removed. The mixture was allowed to warm to 22 °C. After 21 h, the mixture was concentrated, benzene (7 mL) was added to the residue and the solution was concentrated before dry methanol (30 mL) and potassium carbonate (1.94 g, 14.1 mmol, 4.00 equiv) were added.[23] After 1 h, the thick slurry was concentrated to about half of its volume. Water (15 mL) was added and the mixture was extracted with dichloromethane (3 x 50 mL). The combined organic layers were dried over magnesium sulfate, the dried solution was filtered, and the filtrate was concentrated. The residue was purified by flash column chromatography on silica gel (5% ethyl acetate in cyclohexane) yielding epoxide **S23** (1.09 g, 2.99 mmol, 85%) as acolorless oil.

TLC (10% ethyl acetate in cyclohexane): R_f: 0.46.

^{1}H NMR (400 MHz, Benzene-*d*$_6$) δ 7.08 – 7.02 (m, 2H), 6.94 – 6.87 (m, 2H), 6.86 – 6.80 (m, 1H), 5.45 (s, 1H), 4.99 (tdd, *J* = 5.7, 2.7, 1.3 Hz, 1H), 2.52 (dd, *J* = 6.7, 5.6 Hz, 1H), 2.10 – 1.93 (m, 6H), 1.56 – 1.45 (m, 2H), 1.42 (d, *J* = 1.3 Hz, 3H), 1.16 (s, 3H), 1.09 (s, 3H).

^{13}C NMR (101 MHz, Benzene-*d*$_6$) δ 155.6, 155.1, 136.0, 129.9, 123.2, 122.9, 117.2, 92.2, 63.4, 57.4, 36.8, 32.4, 27.9, 25.2, 25.0, 18.9, 15.9.

IR (ATR, neat) $\tilde{\nu}_{max}$: 2959 (w), 2924 (w), 2855 (w), 1645 (w), 1592 (m), 1490 (s), 1455 (w), 1377 (m), 1332 (w), 1267 (w), 1214 (s), 1163 (m), 1132 (m), 1073 (w), 1022 (w), 955 (w), 922 (w), 873 (m), 749 (s), 690 (s), 575 (w), 498 (m) cm^{-1}.

HRMS (ESI): calcd for $C_{19}H_{25}BrO_2Na^+$ $[M+Na]^+$: 387.0930; found: 387.0918.

$[\alpha]_D^{20}$ = –2.0 (c = 0.99, dichloromethane).

[22] Methanesulfonyl chloride was freshly distilled from P_4O_{10} through a Vigreux column under a N_2 atmosphere.
[23] Potassium carbonate was ground to a fine powder in a mortar before use.

2.2.16 Cyclization precursor **S24**

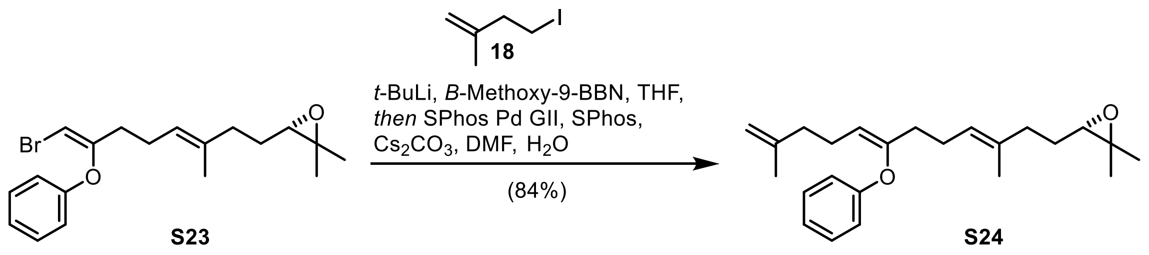

To a solution of iodide **18** (80.5 mg, 411 µmol, 1.50 equiv) and 9-methoxy-9-borabicyclo[3.3.1]nonane (1.00 M in hexanes, 958 µL, 958 µmol, 3.50 equiv) in degassed dry tetrahydrofuran (1.7 mL) was added *tert*-butyllithium (1.60 M in pentane, 770 µL, 1.23 mmol, 4.50 equiv) dropwise at −78 °C. The solution turned yellow and then colorless. After 30 min, the cooling bath was replaced by a water bath (22 °C) and the mixture was warmed to 22 °C. After 5 min the mixture was cooled to –78 °C. A degassed 9:1 mixture of dimethylformamide and water (1 mL) was added to the clear solution and the cooling bath was removed. The reaction mixture was allowed to warm to 22 °C.

A separate flask was charged with vinyl bromide **S23** (100 mg, 274 µmol, 1 equiv), caesium carbonate (178 mg, 547 µmol, 2.00 equiv), 2-dicyclohexylphosphino-2′,6′-dimethoxybiphenyl (5.6 mg, 14 µmol, 5.0 mol%), Chloro(2-dicyclohexylphosphino-2′,6′-dimethoxy-1,1′-biphenyl)[2-(2′-amino-1,1′-biphenyl)]palladium(II) (9.9 mg, 14 µmol, 5.0 mol%) and a degassed 9:1 mixture of dimethylformamide and water (4 mL). To the yellow suspension was added the preformed boronate-species via cannulation and the biphasic mixture was heated at 40 °C. After 5 h, water (5 mL) was added and the mixture was extracted with ethyl acetate (3 x 15 mL). The combined organic layers were washed with water (15 mL) and saturated aqueous solution of sodium chloride (15 mL). The solution was dried over magnesium sulfate, the dried solution was filtered, and the filtrate was concentrated. The residue was purified by flash column chromatography on silica gel (5% ethyl acetate in pentane) yielding cyclization precursor **S24** (81.5 mg, 230 µmol, 84%) as a colorless oil.

TLC (10% ethyl acetate in cyclohexane): R_f: 0.75.

^{1}H NMR (400 MHz, Benzene-*d*$_6$) δ 7.14 – 7.08 (m, 2H), 7.03 – 6.96 (m, 2H), 6.83 (tt, *J* = 7.3, 1.2 Hz, 1H), 5.17 (ddt, *J* = 6.8, 5.5, 1.4 Hz, 1H), 4.97 (t, *J* = 7.1 Hz, 1H), 4.78 (d, *J* = 1.1 Hz, 2H), 2.54 (dd, *J* = 6.7, 5.6 Hz, 1H), 2.32 – 2.24 (m, 2H), 2.23 – 2.13 (m, 4H), 2.13 – 1.96 (m, 4H), 1.61 – 1.47 (m, 8H), 1.14 (s, 3H), 1.09 (s, 3H).

^{13}C NMR (101 MHz, Benzene-*d*$_6$) δ 157.3, 151.0, 145.2, 135.2, 129.9, 124.3, 121.9, 116.5, 115.8, 110.8, 63.5, 57.4, 37.8, 36.8, 32.8, 28.0, 25.9, 25.0, 23.8, 22.3, 18.9, 16.1.

IR (ATR, neat) $\tilde{\nu}_{max}$: 2961 (m), 2924 (m), 2853 (w), 1683 (w), 1651 (w), 1596 (m), 1490 (s), 1455 (w), 1377 (m), 1324 (w), 1292 (w), 1220 (s), 1163 (w), 1130 (w), 1073 (w), 1026 (w), 973 (w), 888 (m), 828 (w), 751 (m), 692 (m) cm^{-1}.

HRMS (ESI): calcd for $C_{24}H_{34}O_2Na^+$ $[M+Na]^+$: 377.2451; found: 377.2448.

$[\alpha]_D^{20}$ = –2.1 (c = 1.0, dichloromethane).

2.3 Isomerization experiment

In order to examine, if isomerization of the (*Z*)-aryl enol ether occurs during polyene cyclization, the following variant of the cyclization precursor without the epoxide was prepared:

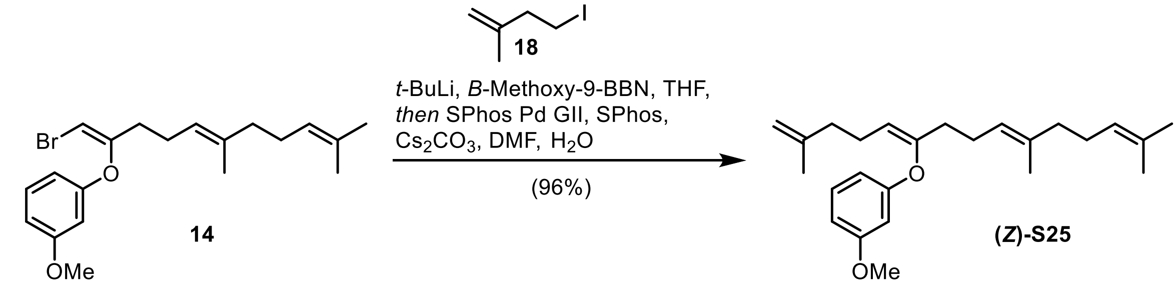

To a solution of iodide **18** (233 mg, 1.19 mmol, 1.50 equiv) and 9-methoxy-9-borabicyclo[3.3.1]nonane (1.00 M in hexanes, 2.77 mL, 2.77 mmol, 3.50 equiv) in degassed dry tetrahydrofuran (4.6 mL) was added *tert*-butyllithium (1.70 M in pentane, 2.09 mL, 3.45 mmol, 4.50 equiv) dropwise at −78 °C. The solution turned yellow and then colorless. After 10 min, the cooling bath was replaced by a water bath (22 °C) and the mixture was warmed to 22 °C. After 25 min the reaction mixture was cooled to −78 °C. A degassed 9:1 mixture of dimethylformamide and water (1 mL) was added to the clear solution and the cooling bath was removed. The reaction mixture was allowed to warm to 22 °C.

A separate flask was charged with vinyl bromide **14** (300 mg, 791 µmol, 1 equiv), caesium carbonate (515 mg, 1.58 mmol, 2.00 equiv), 2-dicyclohexylphosphino-2′,6′-dimethoxybiphenyl (16.2 mg, 39.5 µmol, 5.00 mol%), Chloro(2-dicyclohexylphosphino-2′,6′-dimethoxy-1,1′-biphenyl)[2-(2′-amino-1,1′-biphenyl)]palladium(II) (28.5 mg, 39.5 µmol, 5.00 mol%) and a degassed 9:1 mixture of dimethylformamide and water (8 mL). To the yellow suspension was added the preformed boronate-species via cannulation and the biphasic mixture was heated at 40 °C. After 20 h, water (30 mL) was added and the mixture was extracted with diethyl ether (3 x 30 mL). The combined organic layers were washed with water (30 mL) and saturated aqueous solution of sodium chloride (30 mL). The solution was dried over magnesium sulfate, the dried solution was filtered, and the filtrate was concentrated. The residue was purified by flash column chromatography on silica gel (1% diethyl ether in pentane) yielding cyclization precursor **(*Z*)-S25** (281 mg, 762 µmol, 96%) as a colorless oil.

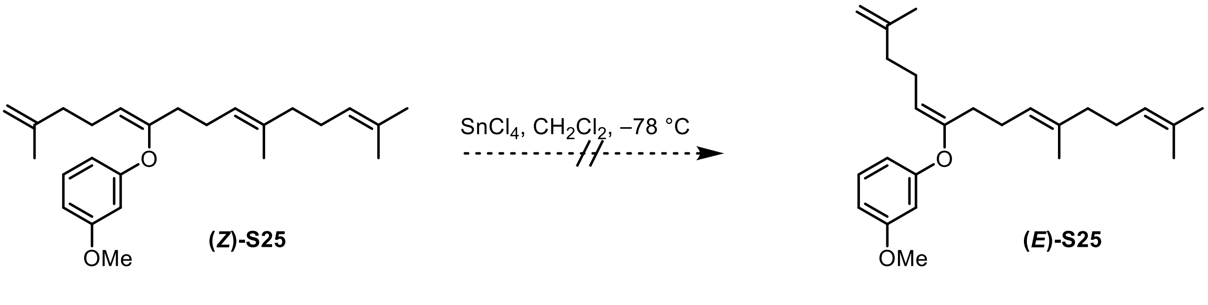

To a solution of enol ether **(*Z*)-S25** (20.0 mg, 54.3 µmol, 1 equiv) in dry dichloromethane (7.8 mL) was added $SnCl_4$ (100 mM in dichloromethane, 814 µL, 81.4 µmol, 1.50 equiv) dropwise over 48 sec at –78 °C. After 20 min, triethylamine (33.8 µl, 244 µmol, 4.50 equiv) was added to the yellow solution leading to decolorization and the reaction mixture was poured into 2 M aqueous solution of sodium hydroxide (7 mL). The aqueous layer was extracted with dichloromethane (2 x 10 mL), and the combined organic layers were dried over magnesium sulfate. The dried solution was filtered, and the filtrate was concentrated. ^{1}H NMR analysis of the residue revealed **(Z)-S25** as the major component (75% NMR purity) along with decomposition products:

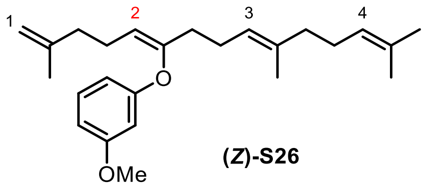

(*Z*)-S26

^{1}H NMR (400 MHz, chloroform-*d*) of **(*Z*)-S26** before treatment with $SnCl_4$

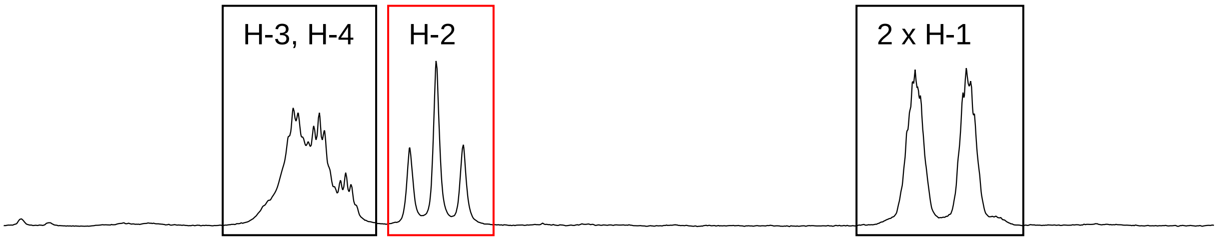

^{1}H NMR (400 MHz, chloroform-*d*) of crude **(*Z*)-S26** after treatment with $SnCl_4$

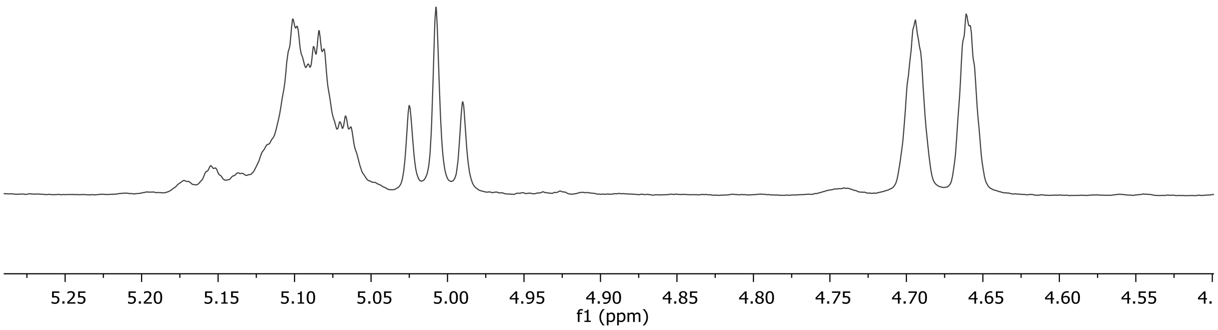

Therefore, we conclude, that isomerization of the (*Z*)-enol ether is at most a minor side-reaction.

<u>Analytical data of **(*Z*)-S25**:</u>

TLC (2% ethyl acetate in pentane): R_f: 0.34.

^{1}H NMR (400 MHz, Benzene-*d*$_6$) δ 7.04 (t, *J* = 8.2 Hz, 1H), 6.77 (t, *J* = 2.3 Hz, 1H), 6.69 (ddd, *J* = 8.1, 2.3, 0.9 Hz, 1H), 6.49 (ddd, *J* = 8.2, 2.4, 0.9 Hz, 1H), 5.27 – 5.17 (m, 2H), 4.98 (t, *J* = 7.1 Hz, 1H), 4.78 (s, 2H), 3.30 (s, 3H), 2.35 – 2.19 (m, 6H), 2.18 – 2.12 (m, 2H), 2.10 – 1.99 (m, 4H), 1.67 (d, *J* = 0.9 Hz, 3H), 1.59 (s, 3H), 1.55 (s, 6H).

^{13}C NMR (101 MHz, Benzene-*d*$_6$) δ 161.8, 158.7, 151.2, 145.2, 135.8, 131.2, 130.3, 124.9, 123.9, 115.9, 110.8, 108.7, 107.6, 102.8, 54.8, 40.2, 37.9, 33.0, 27.2, 25.9, 25.9, 23.8, 22.3, 17.8, 16.1.

IR (ATR, neat) $\tilde{\nu}_{max}$: 2965 (w), 2916 (m), 2855 (w), 1685 (w), 1649 (w), 1592 (m), 1488 (m), 1451 (m), 1375 (w), 1328 (w), 1281 (m), 1194 (m), 1145 (s), 1077 (w), 1043 (w), 979 (w), 888 (w), 835 (w), 765 (w), 688 (w) cm^{-1}.

HRMS (ESI): calcd for $C_{25}H_{37}O_2^+$ $[M+H]^+$: 369.2788; found: 369.2785.

3 NMR comparison data for pimara-15-en-3*α*-8*α*-diol (7)

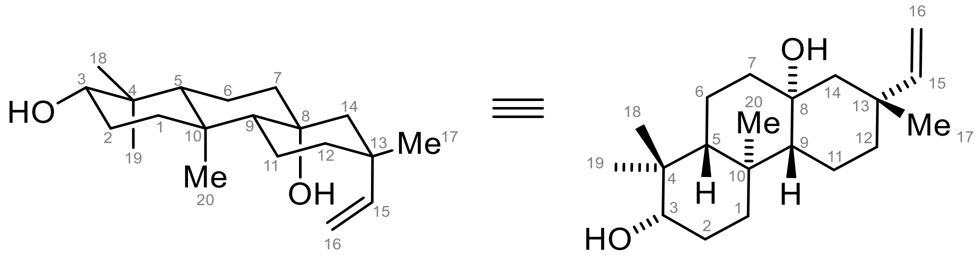

position	^{1}H NMR (600 MHz, Chloroform-*d*) isolated **7** [ppm]	^{1}H NMR (600 MHz, Chloroform-*d*) synthetic **7** [ppm]	Δ ppm	^{13}C NMR (50.32 MHz, Chloroform-*d*) isolated **7** [ppm]	^{13}C NMR (151 MHz, Chloroform-*d*) synthetic **7** [ppm]	Δ ppm
1*α*	1.71 (dt, *J* = 13.1, 3.5 Hz)	1.71*	0.00	37.8	37.8	0.0
1*β*	0.98	0.99 – 0.95 (m, 1H)	--			
2*α*	1.61 (m)	1.62*	+0.01	27.2	27.3	+0.1
2*β*	1.61 (m)	1.62*	+0.01			
3*β*	3.21 (dd, *J* = 11.1, 5.2 Hz)	3.20 (dd, *J* = 11.1, 5.2 Hz)	–0.01	79.1	79.1	0.0
4				38.9	39.0	+0.1
5*β*	0.82	0.85 – 0.81 (m, 2H)	--	55.6	55.7	+0.1
6*α*	1.63 (qd, *J* = 13.4, 3.7 Hz)	1.63*	0.00	17.8	17.8	0.0
6*β*	1.49	1.50 – 1.43 (m, 3H)	--			
7*α*	1.78 (dt, *J* = 13.4, 3.2 Hz)	1.77 (dt, *J* = 13.3, 3.2 Hz)	–0.01	42.0	42.0	0.0
7*β*	1.22	1.24 – 1.18 (m, 3H)	--			
8				72.3	72.3	0.0
9*β*	0.85	0.85 – 0.81 (m, 2H)	--	56.2	56.2	0.0
10				37.0	37.0	0.0
11*α*	1.47 (qd, *J* = 13.4, 3.1 Hz)	1.50 – 1.43 (m, 3H)	--	17.4	17.5	+0.1
11*β*	1.47 (m)	1.50 – 1.43 (m, 3H)	--			
12*α*	2.01 (dq, *J* = 13.7, 3.1 Hz)	2.00 (dq, *J* = 13.6, 3.1 Hz)	–0.01	36.1	36.1	0.0
12*β*	1.21 (dd, *J* = 13.7, 4.4 Hz)	1.22*	+0.01			
13				36.5	36.6	+0.1
14*α*	1.68 (dd, *J* = 14.0, 3.1 Hz)	1.68*	0.00	53.4	53.4	0.0
14*β*	1.23	1.24 – 1.18 (m, 3H)	--			
15	5.98 (dd, *J* = 17.9, 11.0 Hz)	5.98 (dd, *J* = 17.9, 10.9 Hz)	0.00	147.5	147.5	0.0
16A	5.09 (dd, *J* = 11.0, 1.2 Hz)	5.08 (d, *J* = 10.9 Hz)	–0.01	112.0	112.0	0.0
16B	5.14 (dd, *J* = 17.9, 1.2 Hz)	5.13 (d, *J* = 17.9 Hz)	–0.01			
17	0.91 (s)	0.90 (s)	–0.01	28.3	28.3	0.0
18	0.99 (s)	0.99 (s)	0.00	32.4	32.4	0.0
19	0.81 (s)	0.80 (s)	–0.01	15.5	15.51	0.0
20	0.93 (s)	0.93 (s)	0.00	15.5	15.47	0.0

* The signal overlapped with at least one other signal, but the chemical shift could be assigned via the HSQC spectrum.

4 NMR spectra

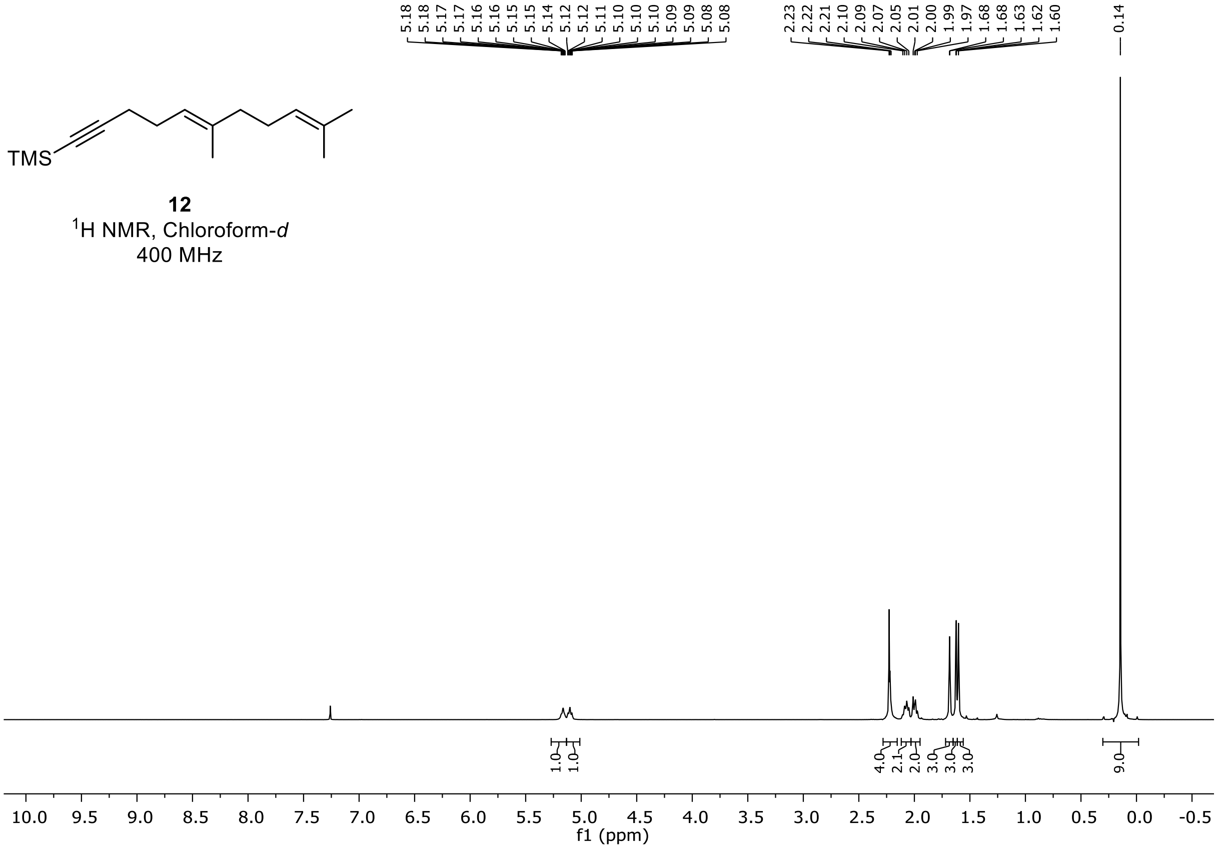

12
^{1}H NMR, Chloroform-*d*
400 MHz

12
^{13}C NMR, Chloroform-*d*
101 MHz

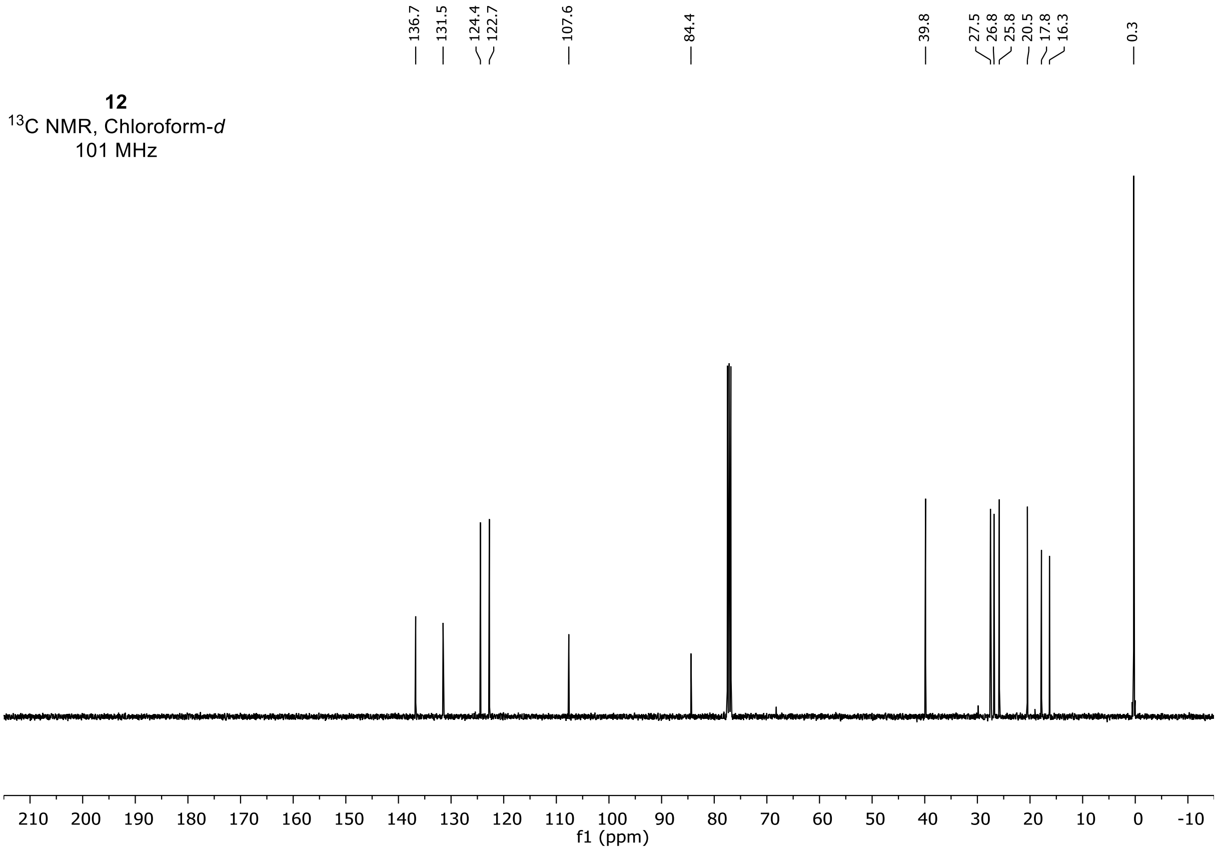

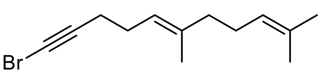

13
^{1}H NMR, Chloroform-*d*
400 MHz

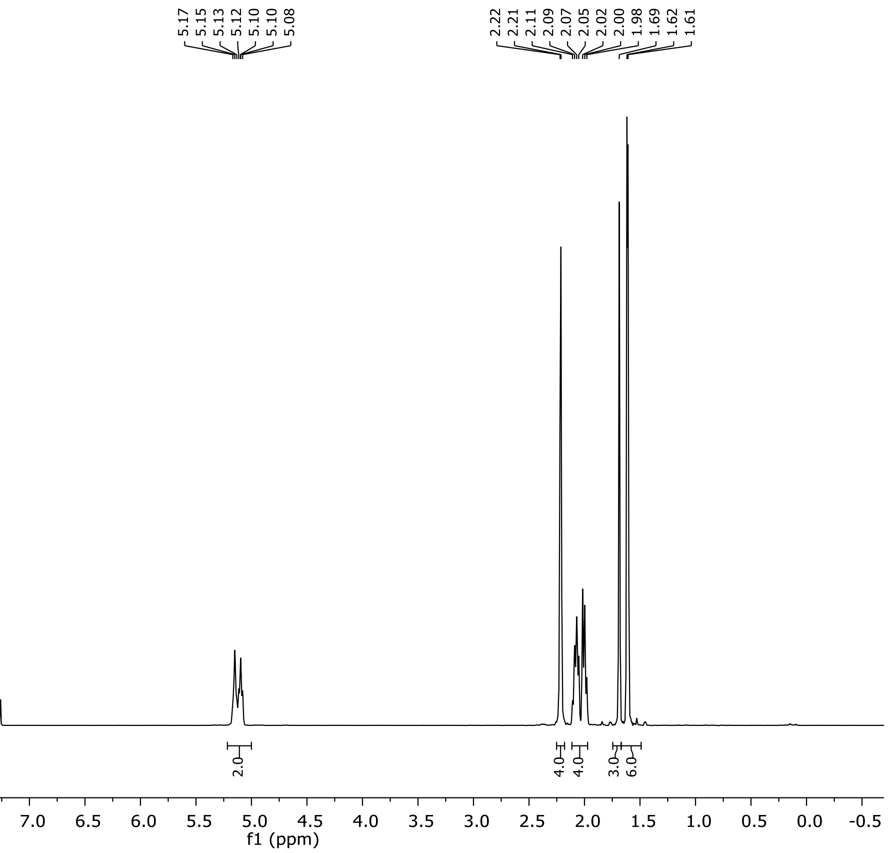

13
^{13}C NMR, Chloroform-*d*
101 MHz

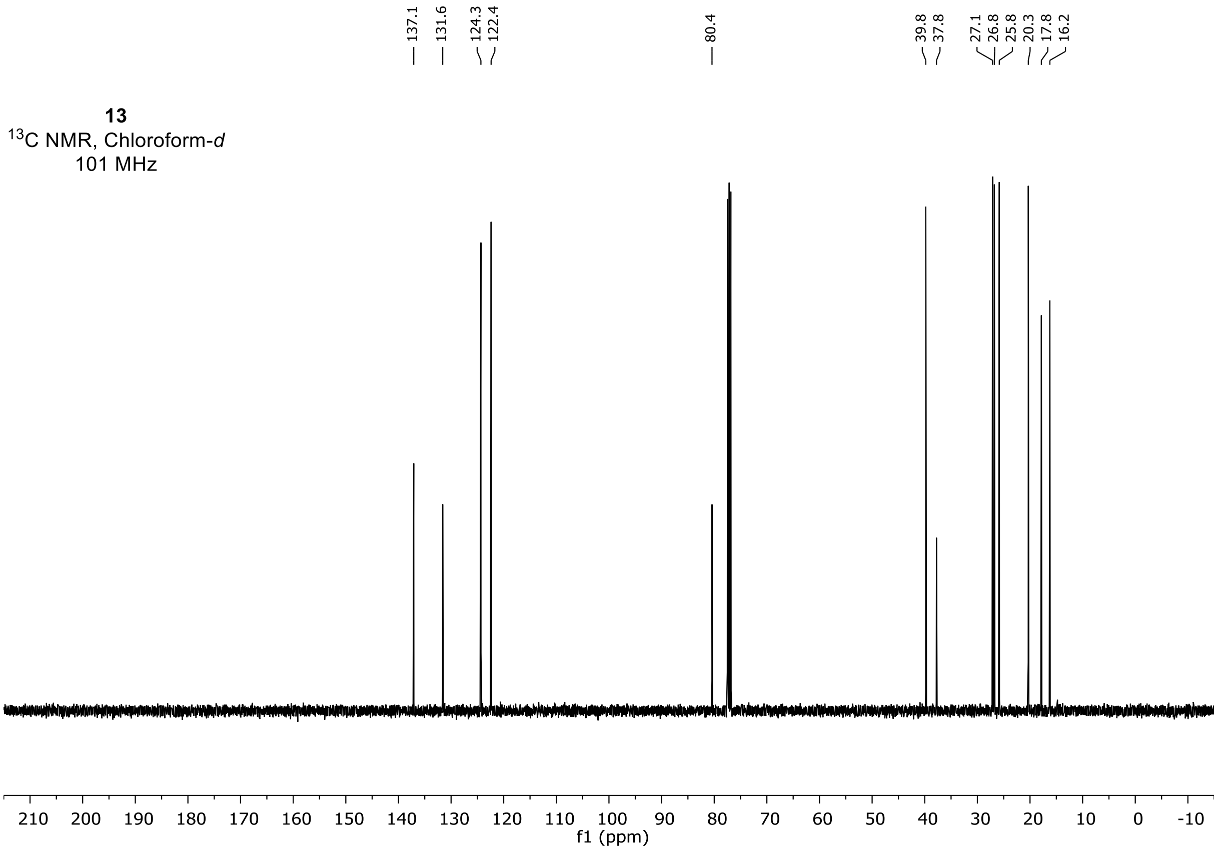

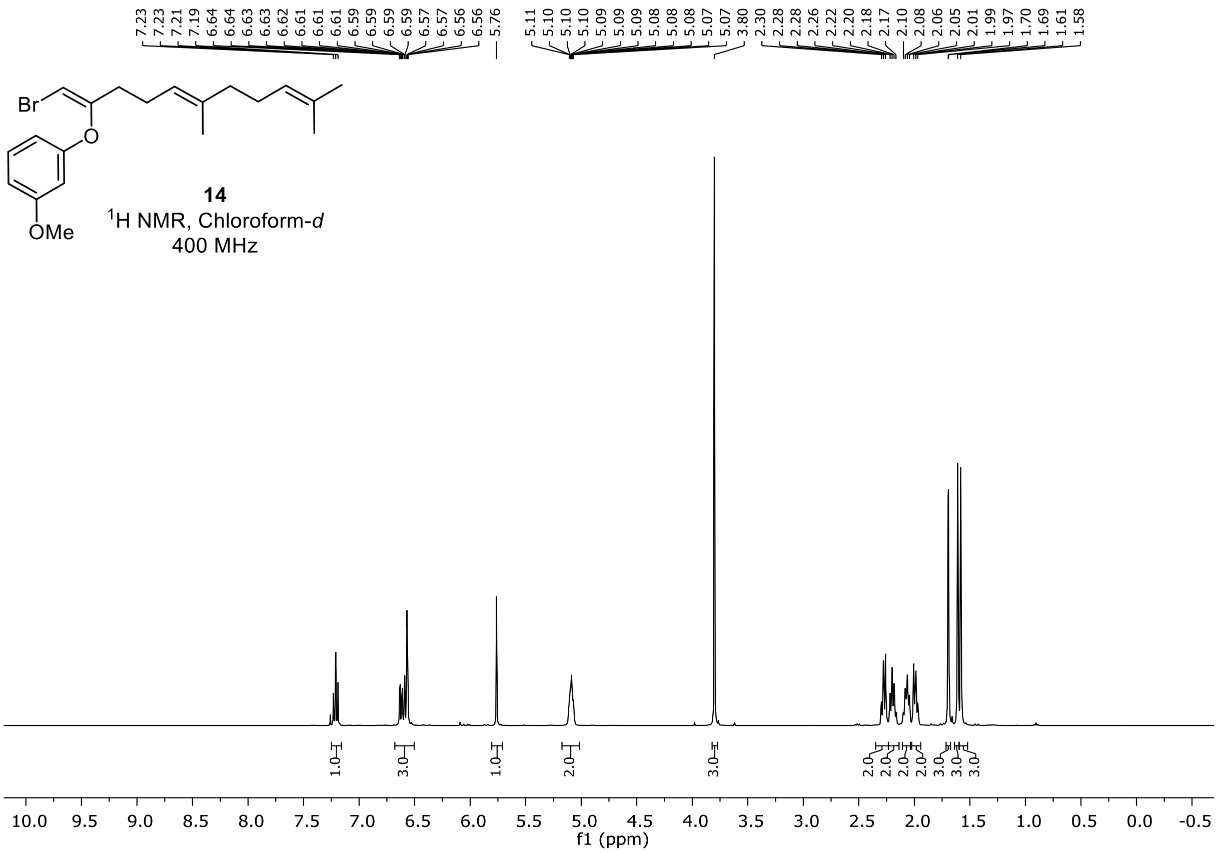
7.23 7.23 7.21 7.19 6.64 6.64 6.63 6.63 6.62 6.61 6.61 6.61 6.59 6.59 6.59 6.59 6.57 6.57 6.56 6.56 5.76 5.11 5.10 5.10 5.10 5.09 5.09 5.09 5.08 5.08 5.07 5.07 3.80 2.30 2.28 2.26 2.22 2.20 2.18 2.17 2.10 2.08 2.06 2.05 2.01 1.99 1.97 1.70 1.69 1.61 1.58
Br
O
OMe
14
^{1}H NMR, Chloroform-*d*
400 MHz
1.0 3.0 1.0 2.0 3.0 2.0 2.0 2.0 2.0 3.0 3.0 3.0
10.0 9.5 9.0 8.5 8.0 7.5 7.0 6.5 6.0 5.5 5.0 4.5 4.0 3.5 3.0 2.5 2.0 1.5 1.0 0.5 0.0 -0.5
f1 (ppm)

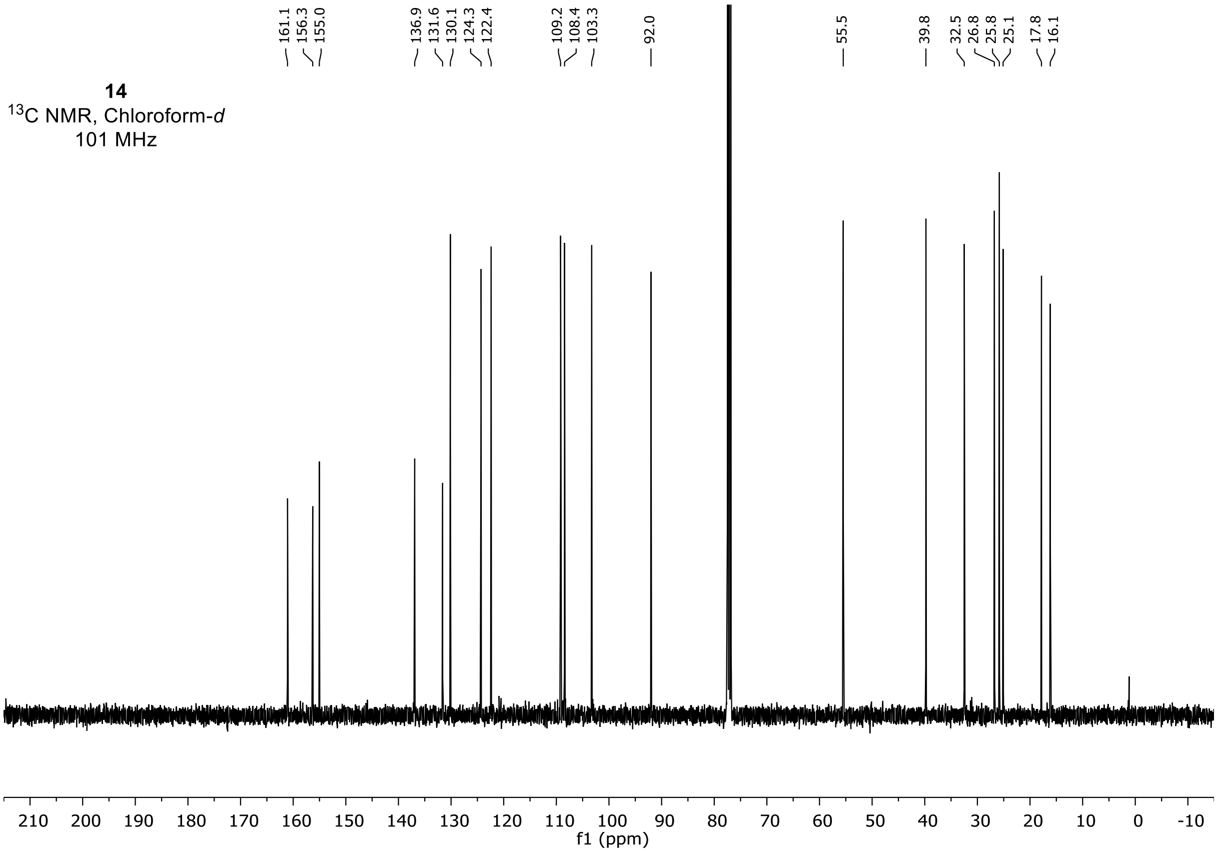
161.1 156.3 155.0 136.9 131.6 130.1 124.3 122.4 109.2 108.4 103.3 92.0 55.5 39.8 32.5 26.8 25.8 25.1 17.8 16.1
14
^{13}C NMR, Chloroform-*d*
101 MHz
210 200 190 180 170 160 150 140 130 120 110 100 90 80 70 60 50 40 30 20 10 0 -10
f1 (ppm)

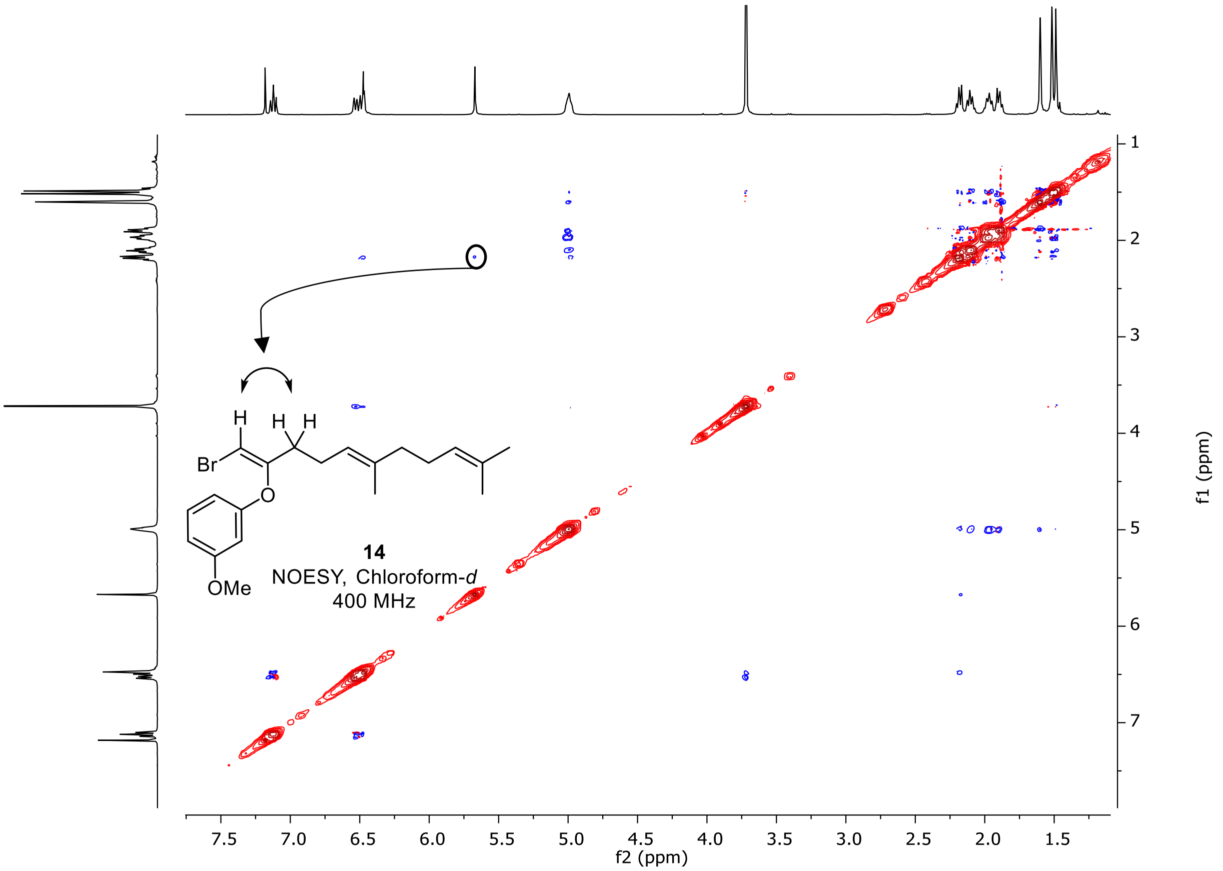
Br
O
OMe
H
H H
14
NOESY, Chloroform-d
400 MHz
f2 (ppm)
f1 (ppm)

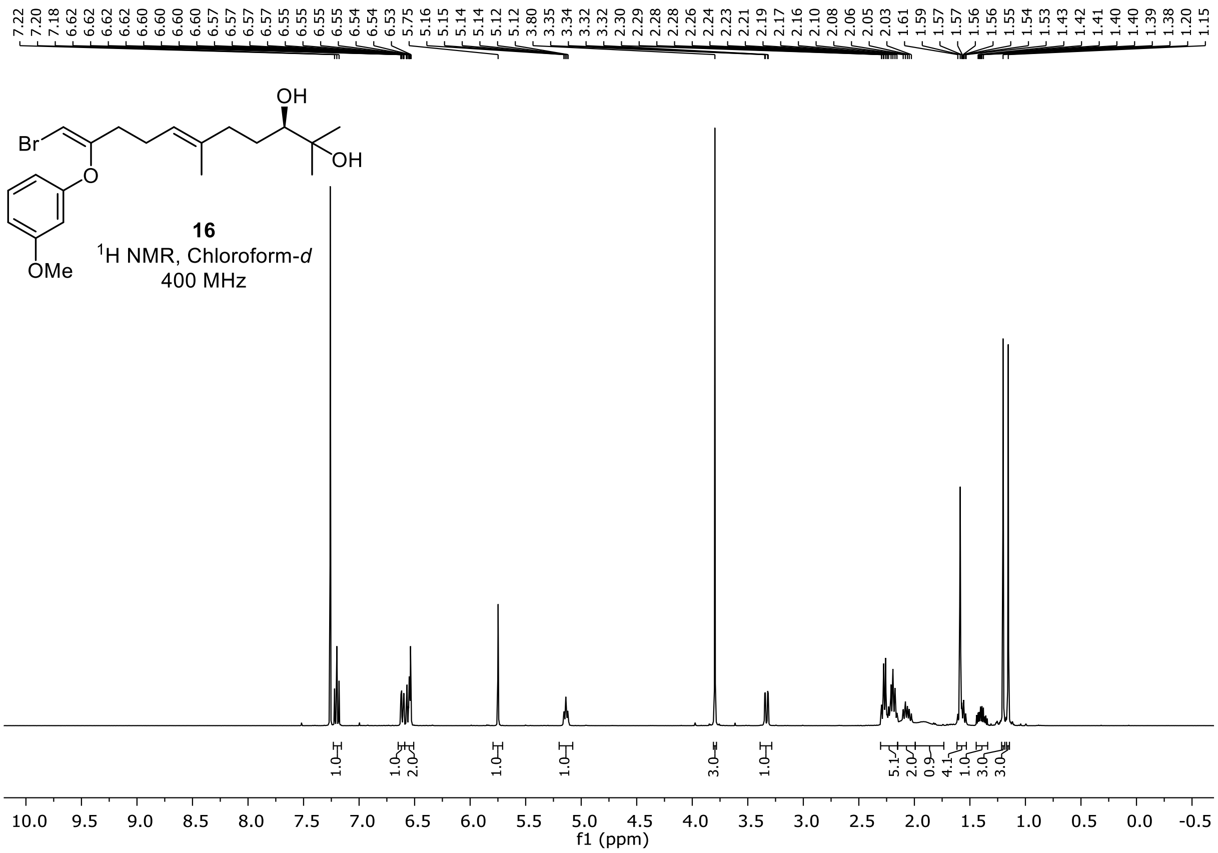
7.22
7.20
7.18
6.62
6.60
6.57
6.55
6.54
6.53
5.75
5.16
5.15
5.14
5.12
3.80
3.35
3.34
3.32
2.30
2.29
2.28
2.26
2.24
2.23
2.21
2.19
2.17
2.16
2.10
2.08
2.06
2.05
2.03
1.61
1.59
1.57
1.56
1.55
1.54
1.53
1.43
1.42
1.41
1.40
1.39
1.38
1.20
1.15
Br
O
OMe
OH
OH
16
^{1}H NMR, Chloroform-*d*
400 MHz
1.0
1.0
2.0
1.0
1.0
3.0
1.0
5.1
2.0
0.9
4.1
1.0
3.0
3.0
f1 (ppm)

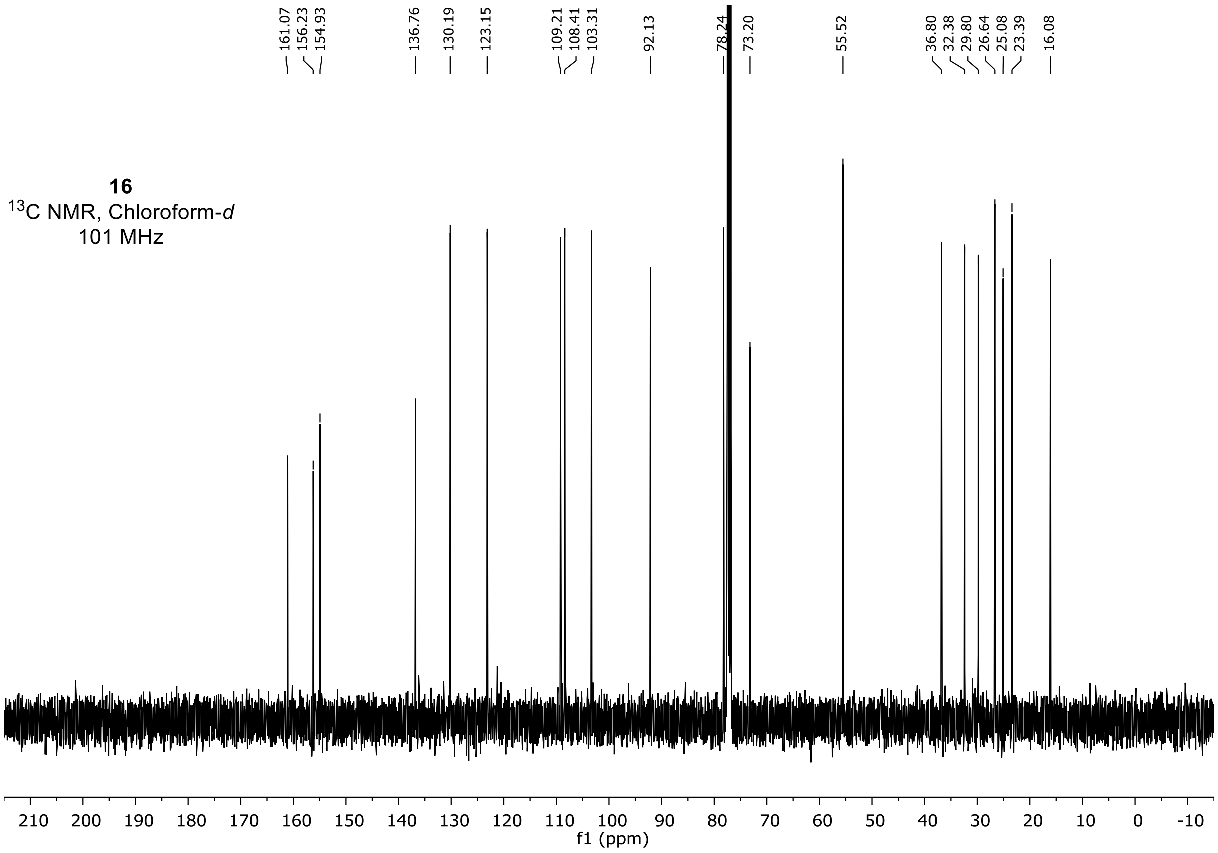
161.07
156.23
154.93
136.76
130.19
123.15
109.21
108.41
103.31
92.13
78.24
73.20
55.52
36.80
32.38
29.80
26.64
25.08
23.39
16.08
16
^{13}C NMR, Chloroform-*d*
101 MHz
f1 (ppm)

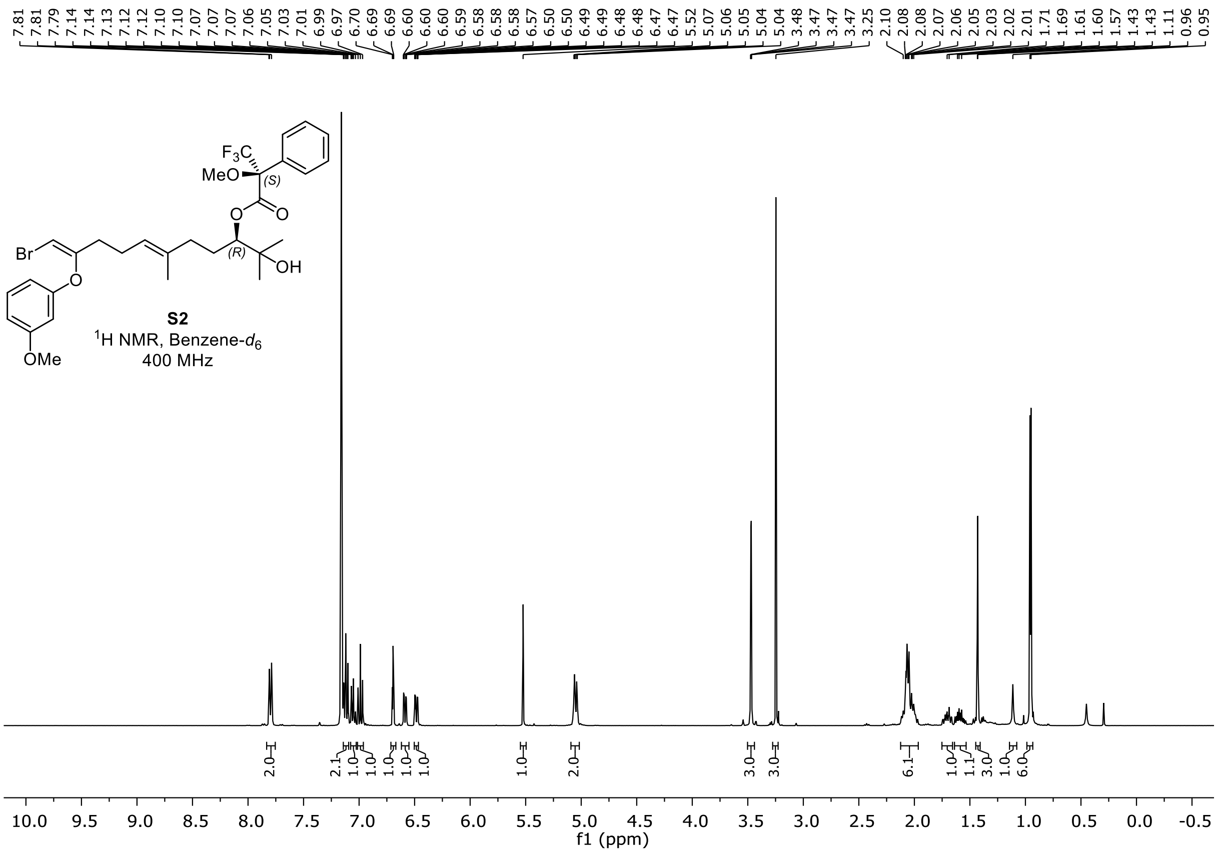
S2
^{1}H NMR, Benzene-d_6
400 MHz
f1 (ppm)

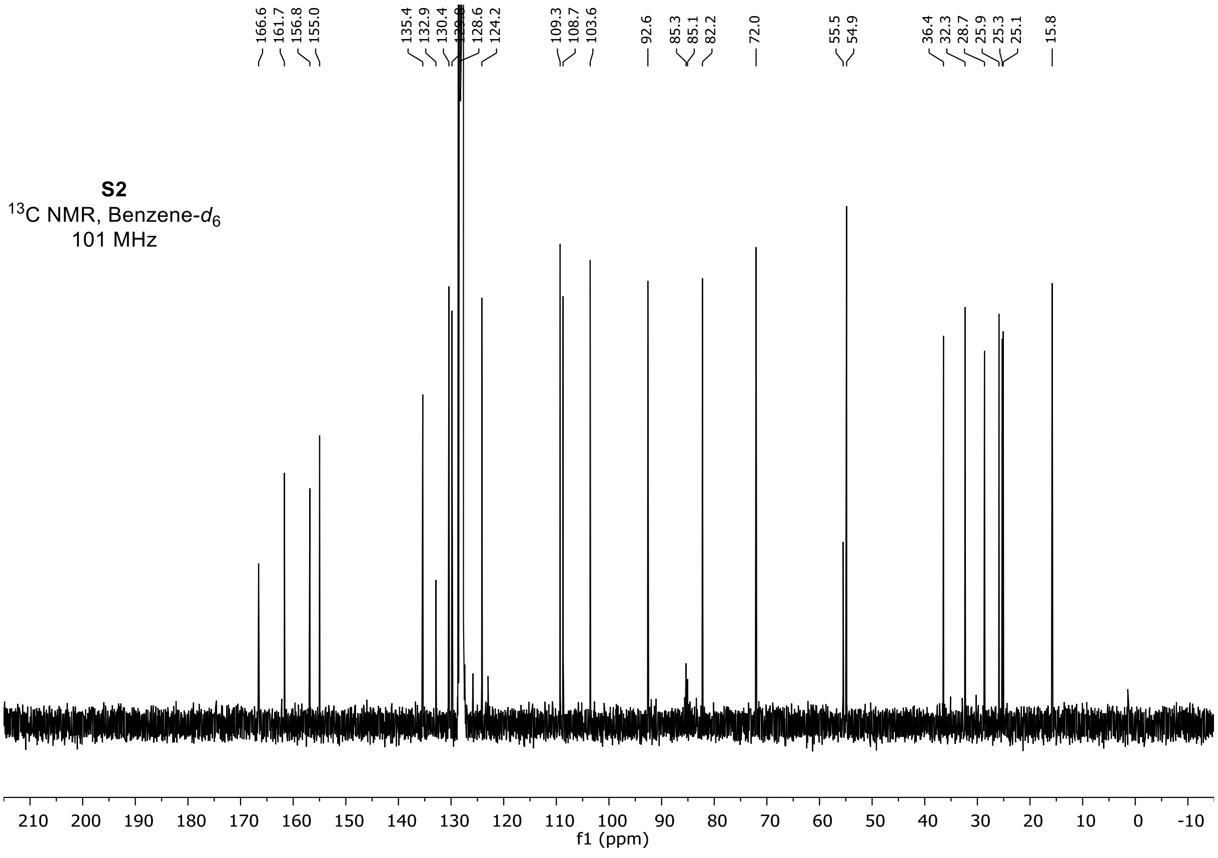
S2
^{13}C NMR, Benzene-d_6
101 MHz
f1 (ppm)

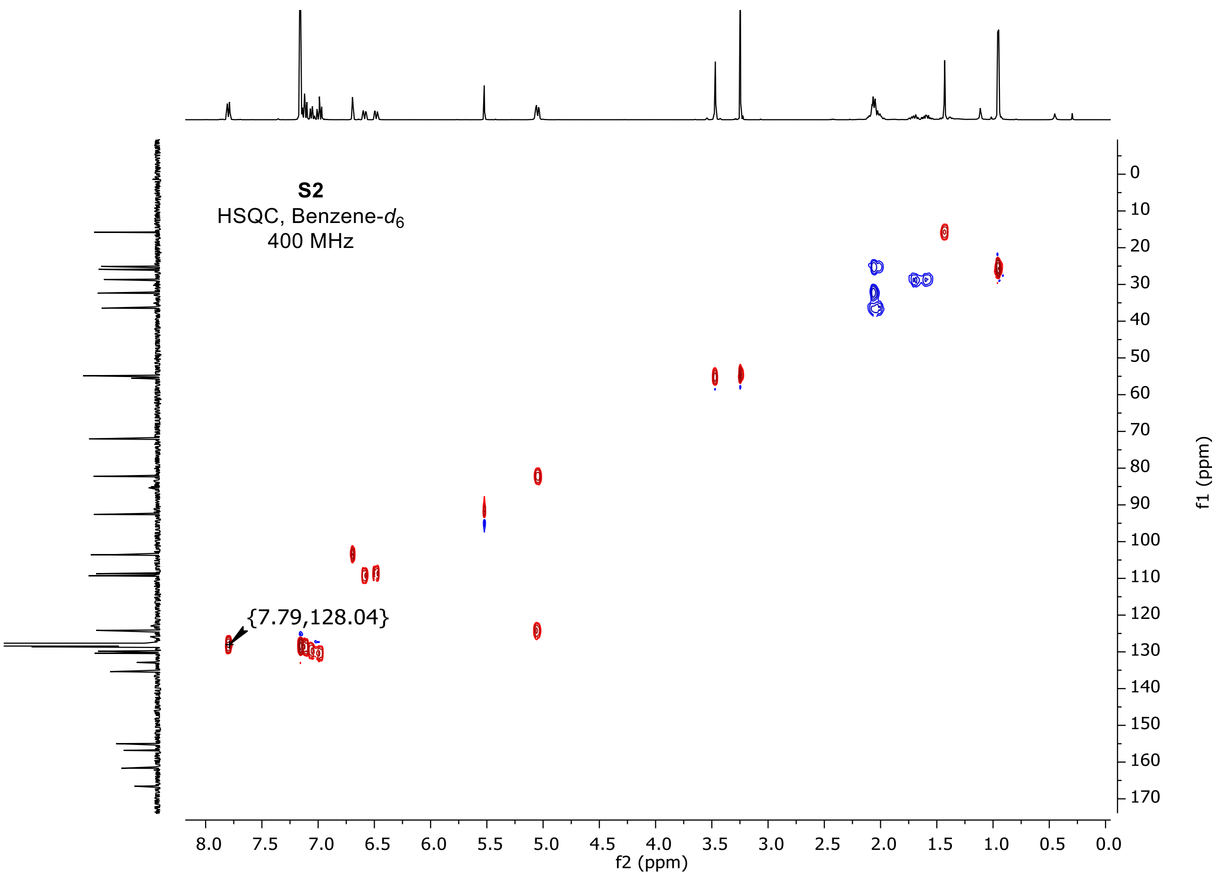
S2
HSQC, Benzene-d_6
400 MHz
{7.79,128.04}
f2 (ppm)
f1 (ppm)

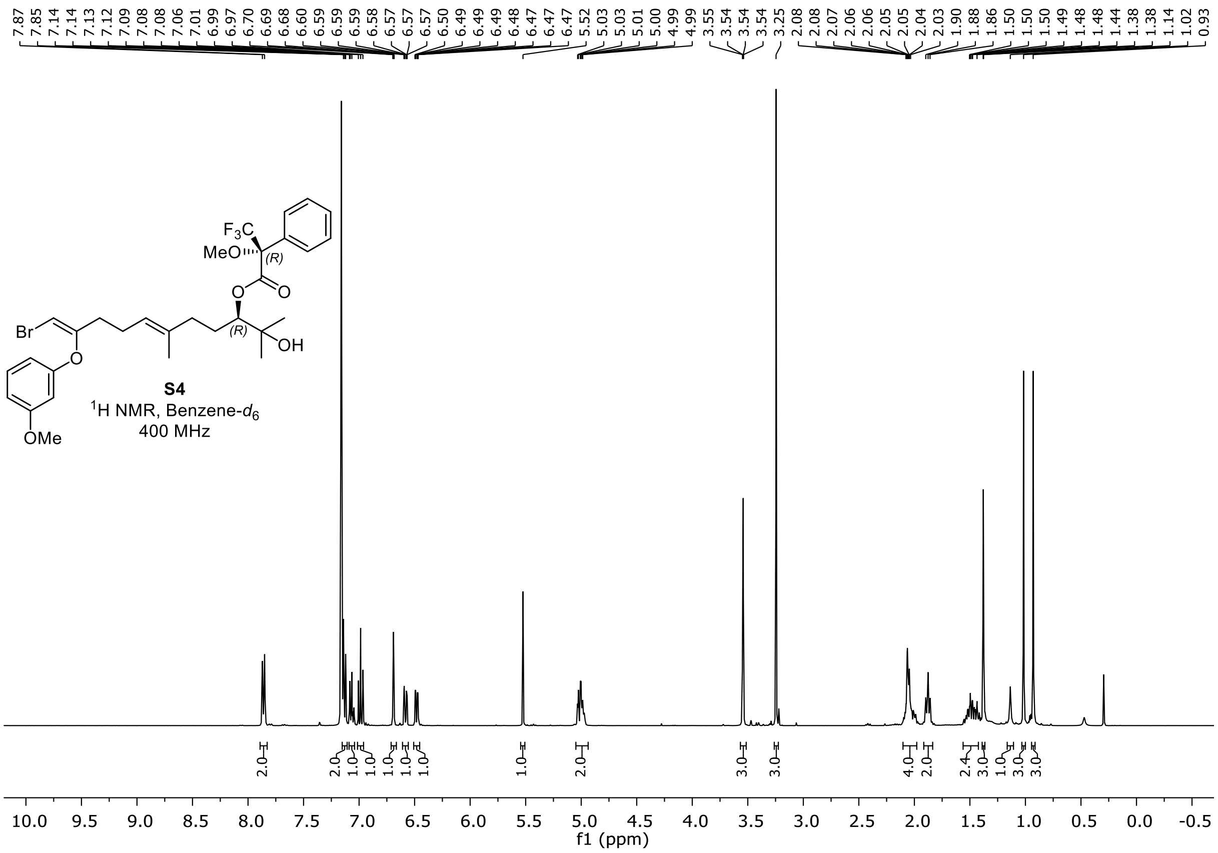

F3C
MeO
(R)
O
O
Br
O
(R)
OH
OMe
S4
1H NMR, Benzene-d6
400 MHz
f1 (ppm)

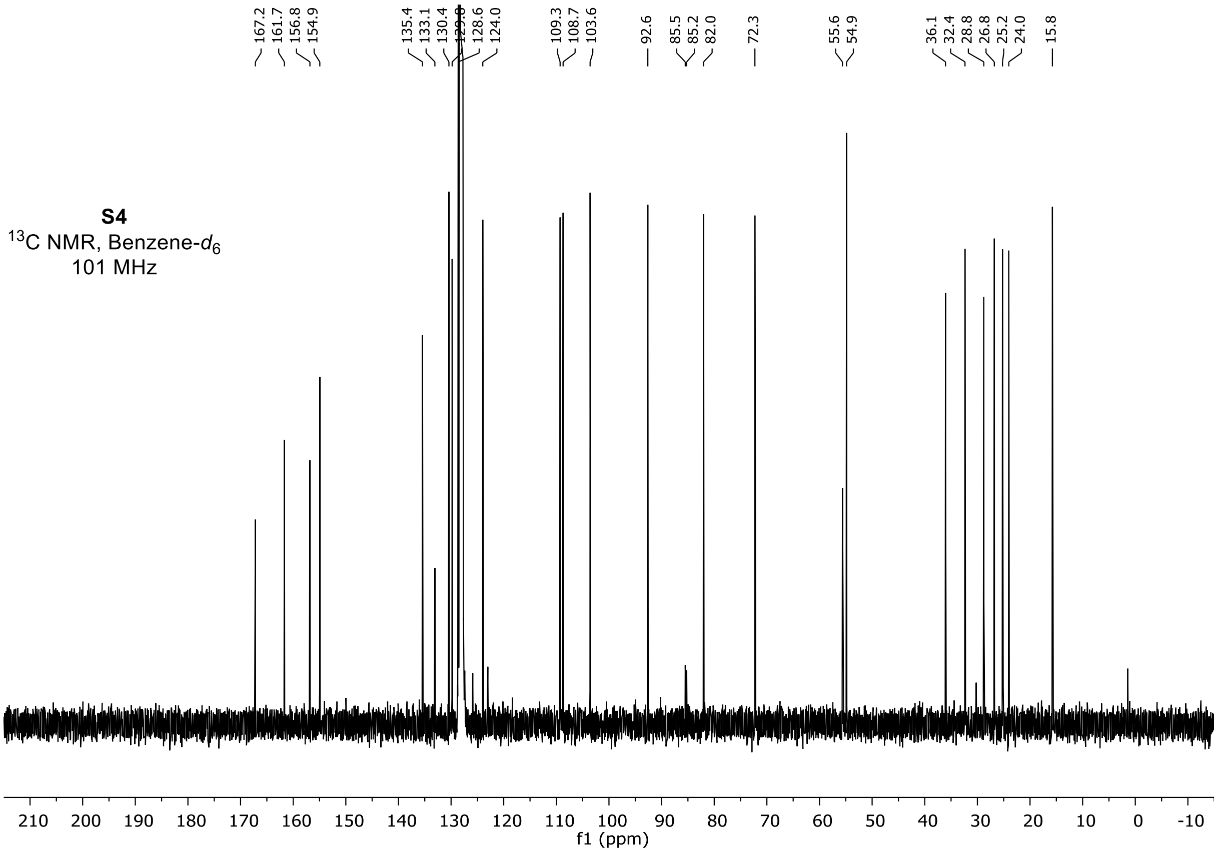

S4
13C NMR, Benzene-d6
101 MHz
167.2
161.7
156.8
154.9
135.4
133.1
130.4
128.6
124.0
109.3
108.7
103.6
92.6
85.5
85.2
82.0
72.3
55.6
54.9
36.1
32.4
28.8
26.8
25.2
24.0
15.8
f1 (ppm)

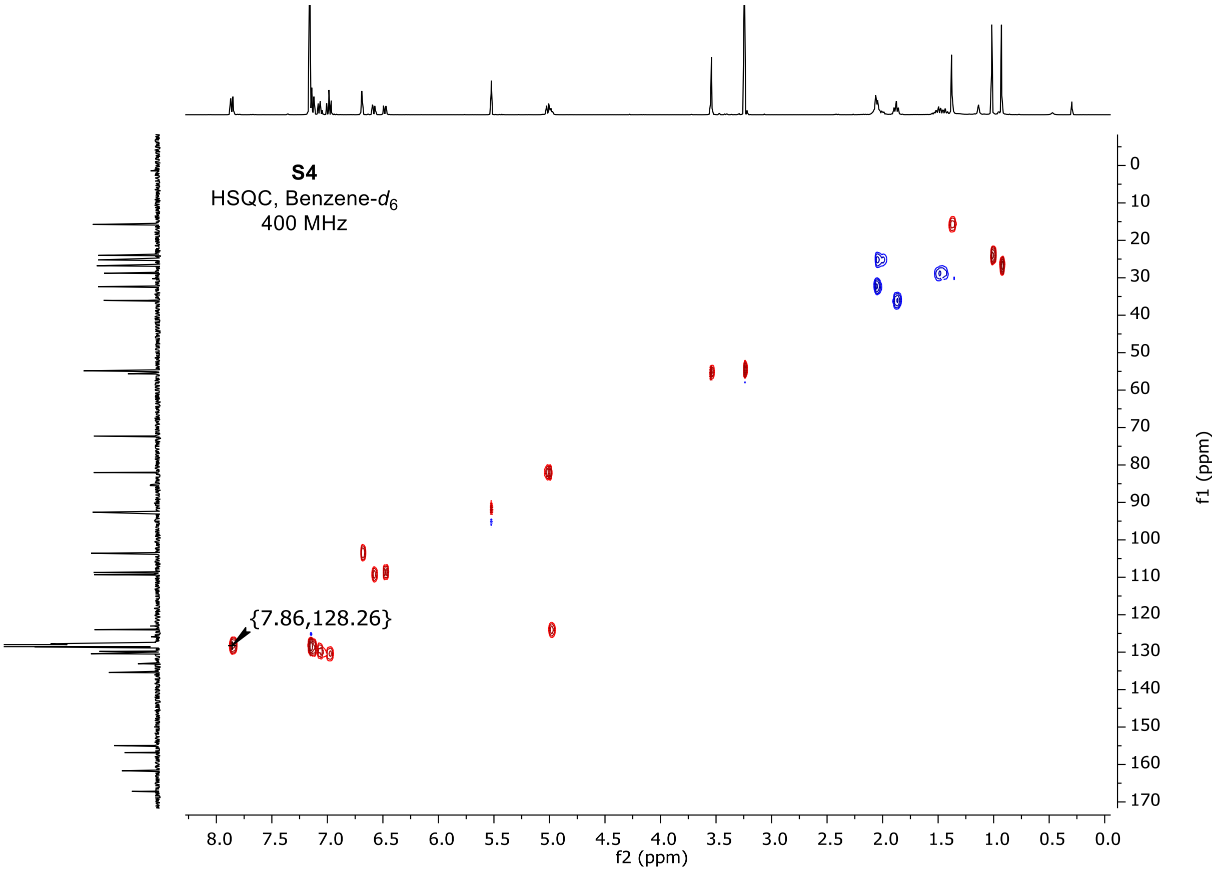
S4
HSQC, Benzene-d6
400 MHz
{7.86,128.26}
f2 (ppm)
f1 (ppm)

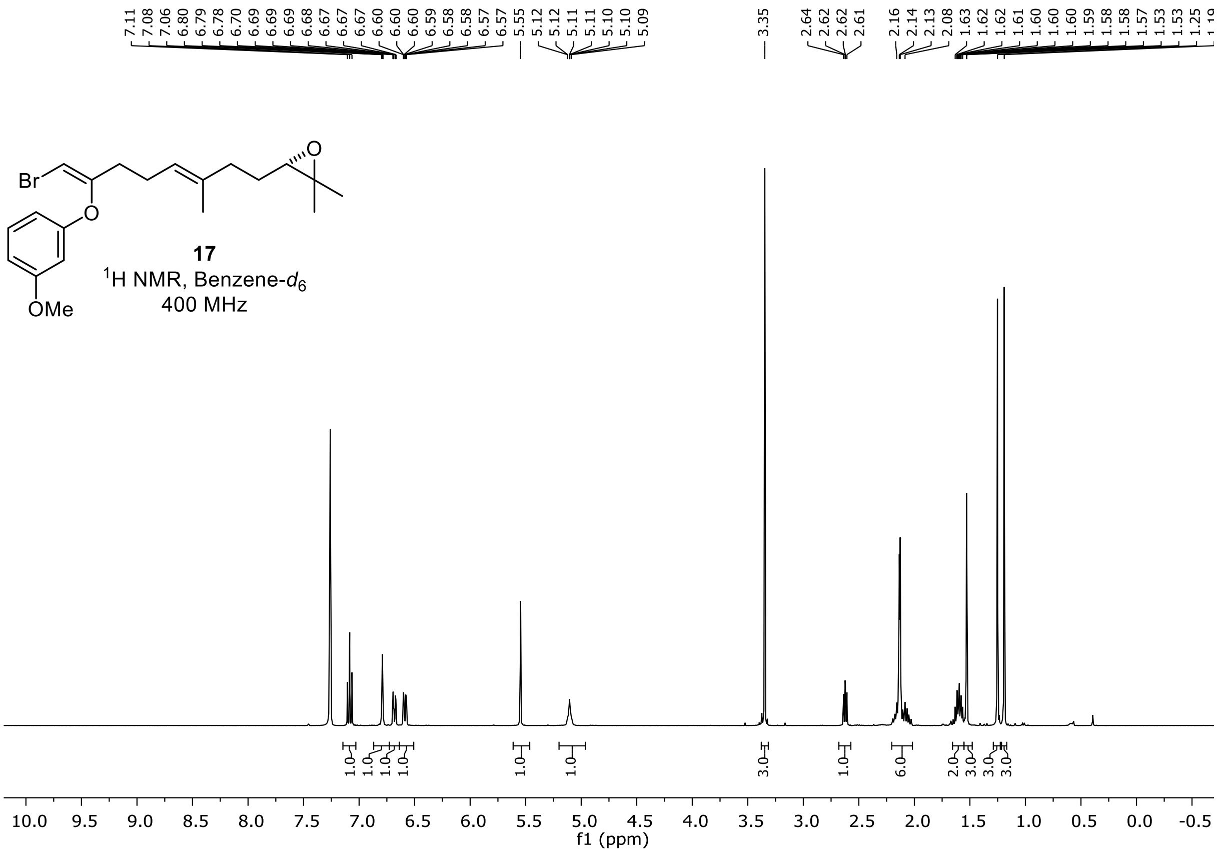
Br
O
OMe
O
17
^{1}H NMR, Benzene-d_6
400 MHz
f1 (ppm)

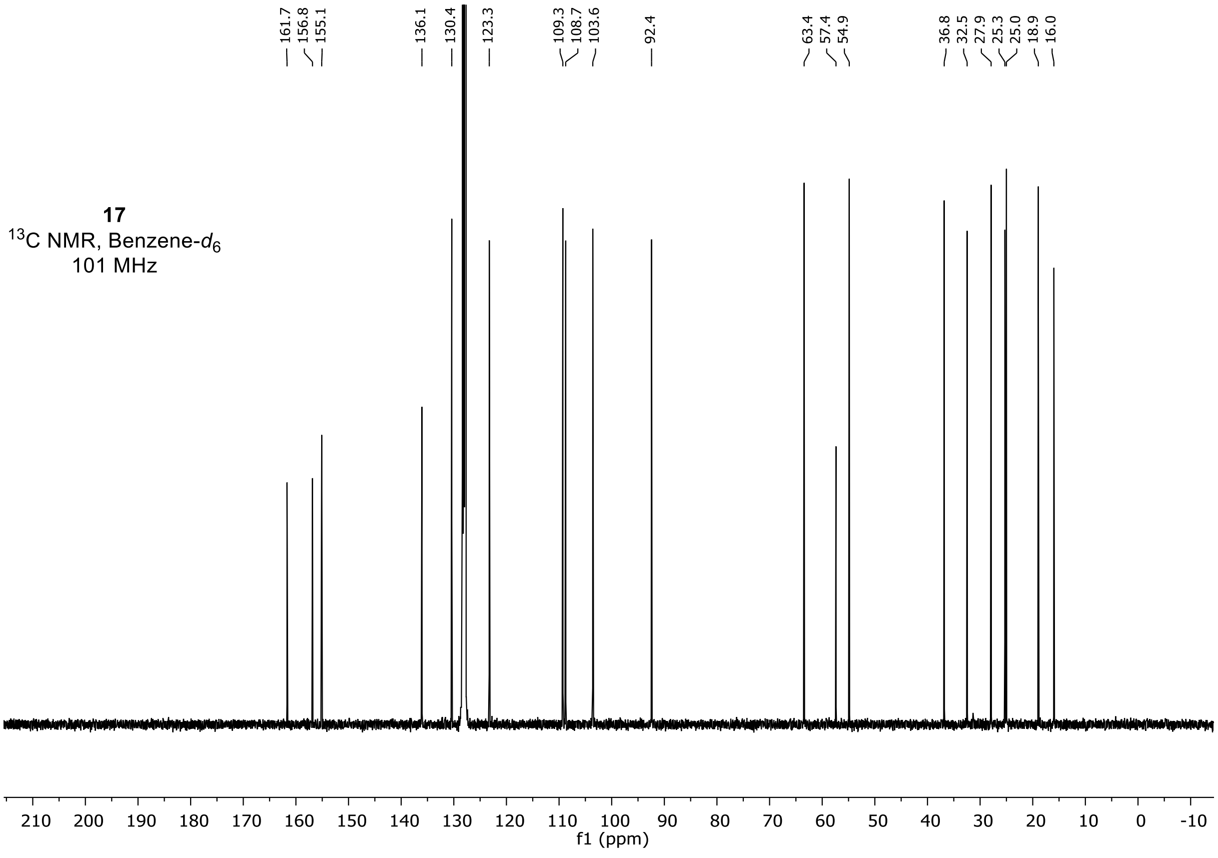
17
^{13}C NMR, Benzene-d_6
101 MHz
f1 (ppm)

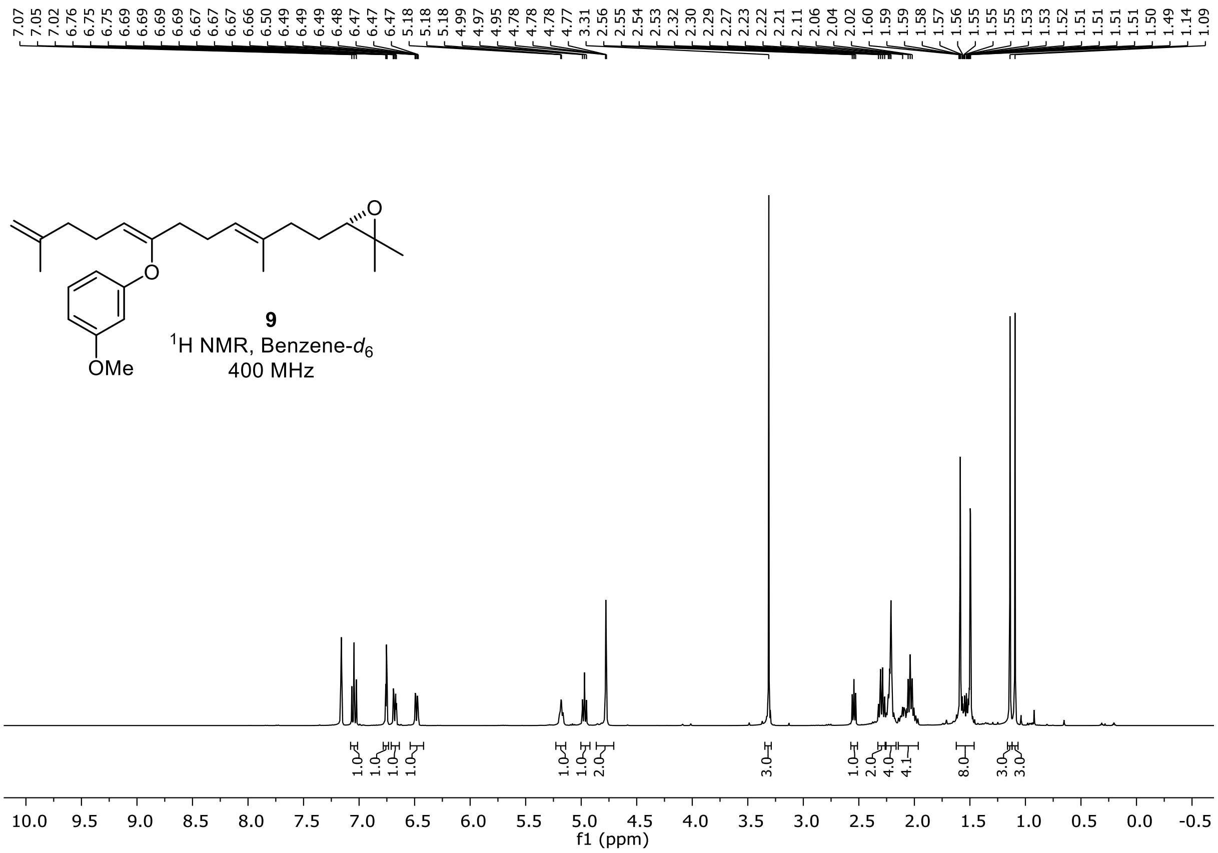
7.07 7.05 7.02 6.76 6.75 6.69 6.69 6.69 6.69 6.67 6.67 6.66 6.50 6.49 6.49 6.49 6.48 6.47 6.47 6.47 5.18 5.18 5.18 4.99 4.97 4.95 4.78 4.78 4.78 4.77 3.31 2.56 2.55 2.54 2.53 2.32 2.30 2.29 2.27 2.23 2.22 2.22 2.21 2.11 2.06 2.04 2.02 1.60 1.59 1.59 1.58 1.57 1.56 1.55 1.55 1.55 1.53 1.53 1.52 1.51 1.51 1.51 1.51 1.50 1.49 1.14 1.09
O
O
OMe
9
^{1}H NMR, Benzene-d_6
400 MHz
1.0 1.0 1.0 1.0 1.0 1.0 2.0 3.0 1.0 2.0 4.0 4.1 8.0 3.0 3.0
10.0 9.5 9.0 8.5 8.0 7.5 7.0 6.5 6.0 5.5 5.0 4.5 4.0 3.5 3.0 2.5 2.0 1.5 1.0 0.5 0.0 -0.5
f1 (ppm)

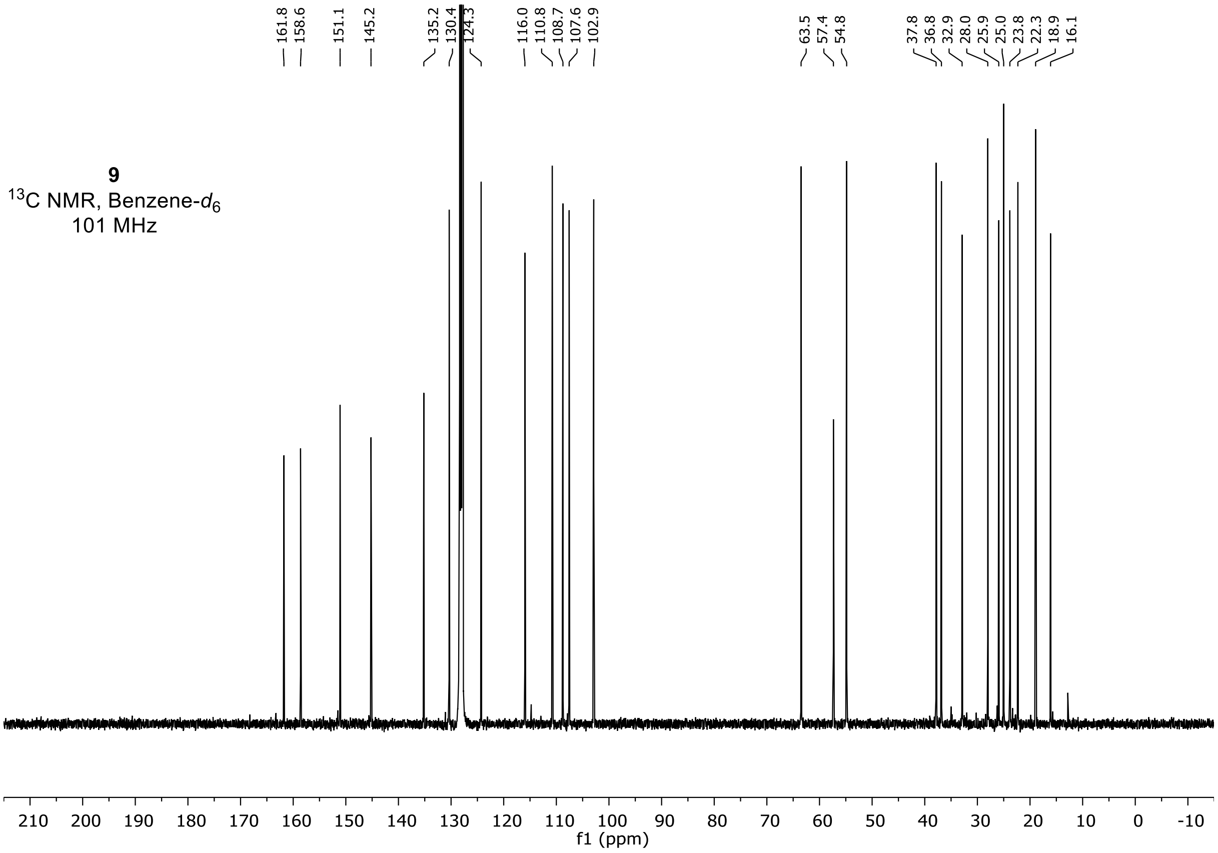
161.8 158.6 151.1 145.2 135.2 130.4 124.3 116.0 110.8 108.7 107.6 102.9 63.5 57.4 54.8 37.8 36.8 32.9 28.0 25.9 25.0 23.8 22.3 18.9 16.1
9
^{13}C NMR, Benzene-d_6
101 MHz
210 200 190 180 170 160 150 140 130 120 110 100 90 80 70 60 50 40 30 20 10 0 -10
f1 (ppm)

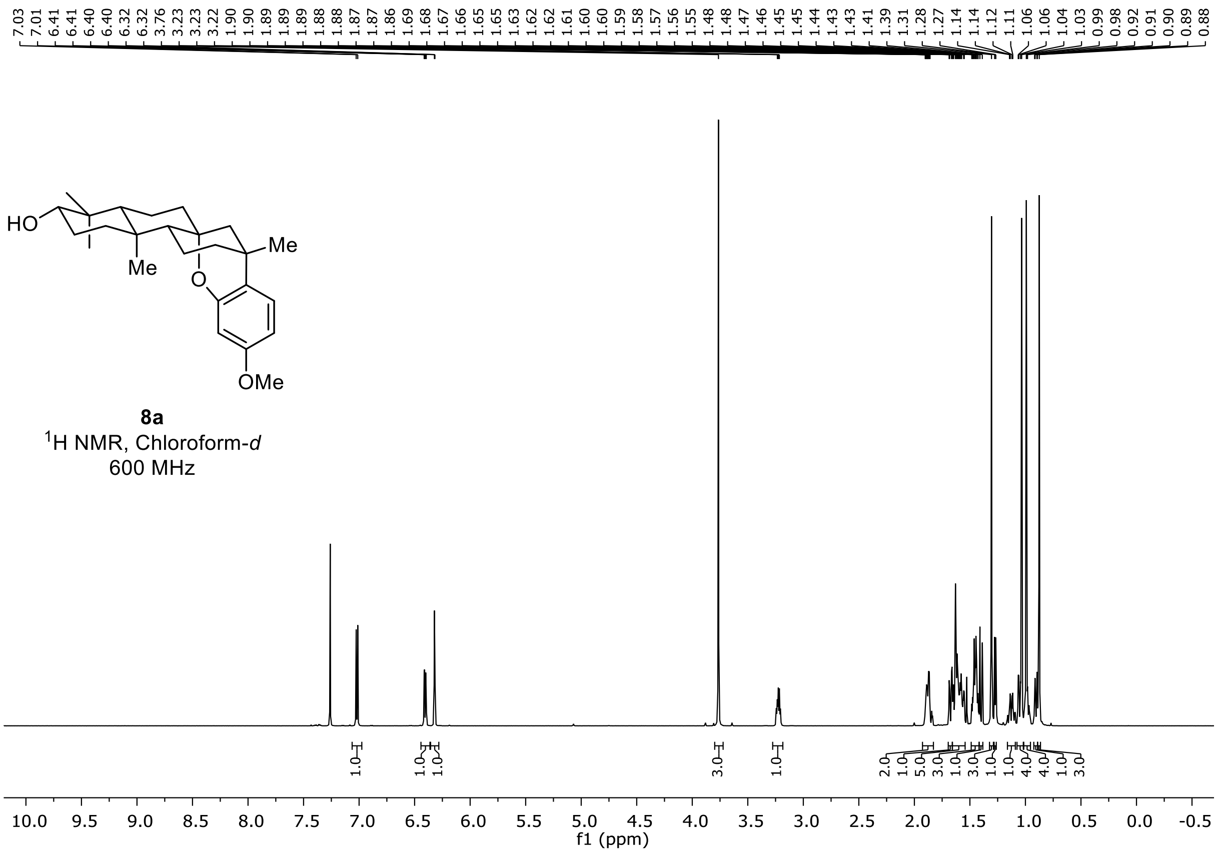

8a
^{1}H NMR, Chloroform-*d*
600 MHz

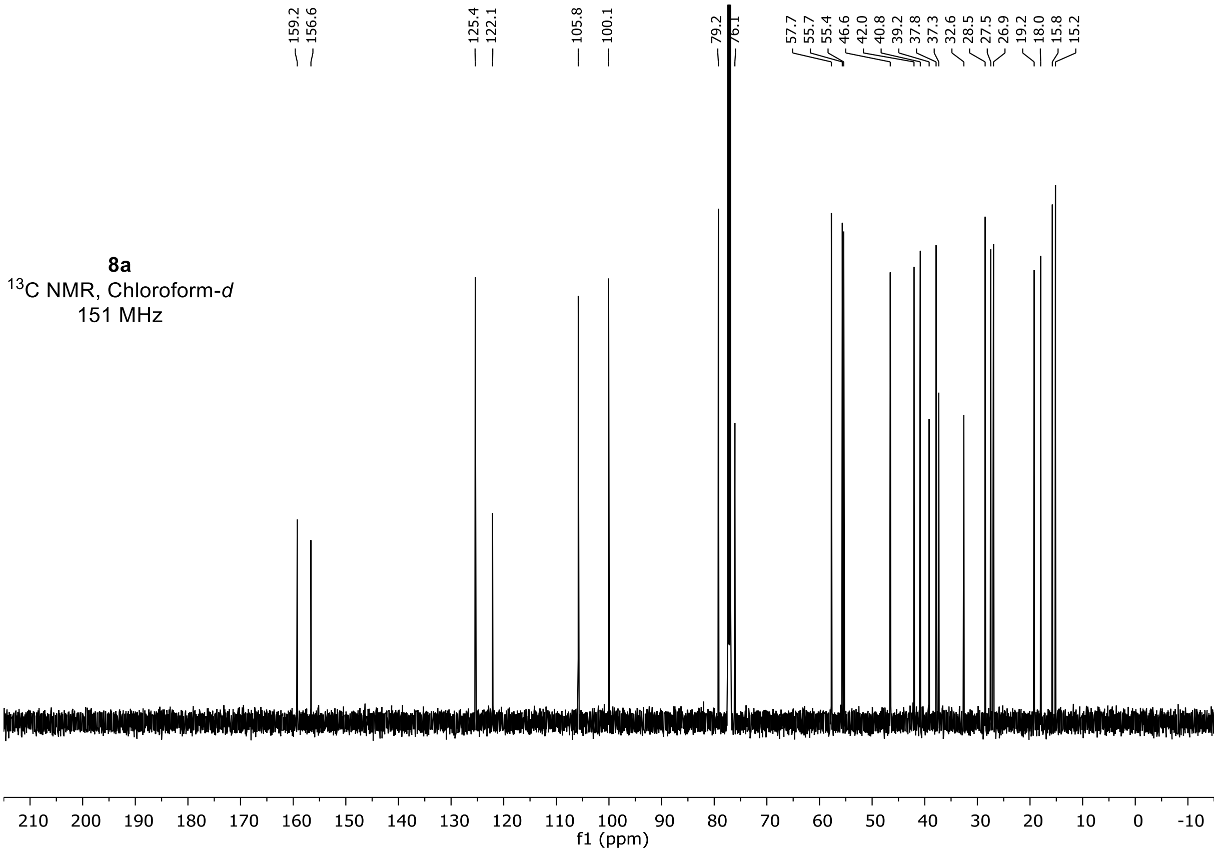

8a
^{13}C NMR, Chloroform-*d*
151 MHz

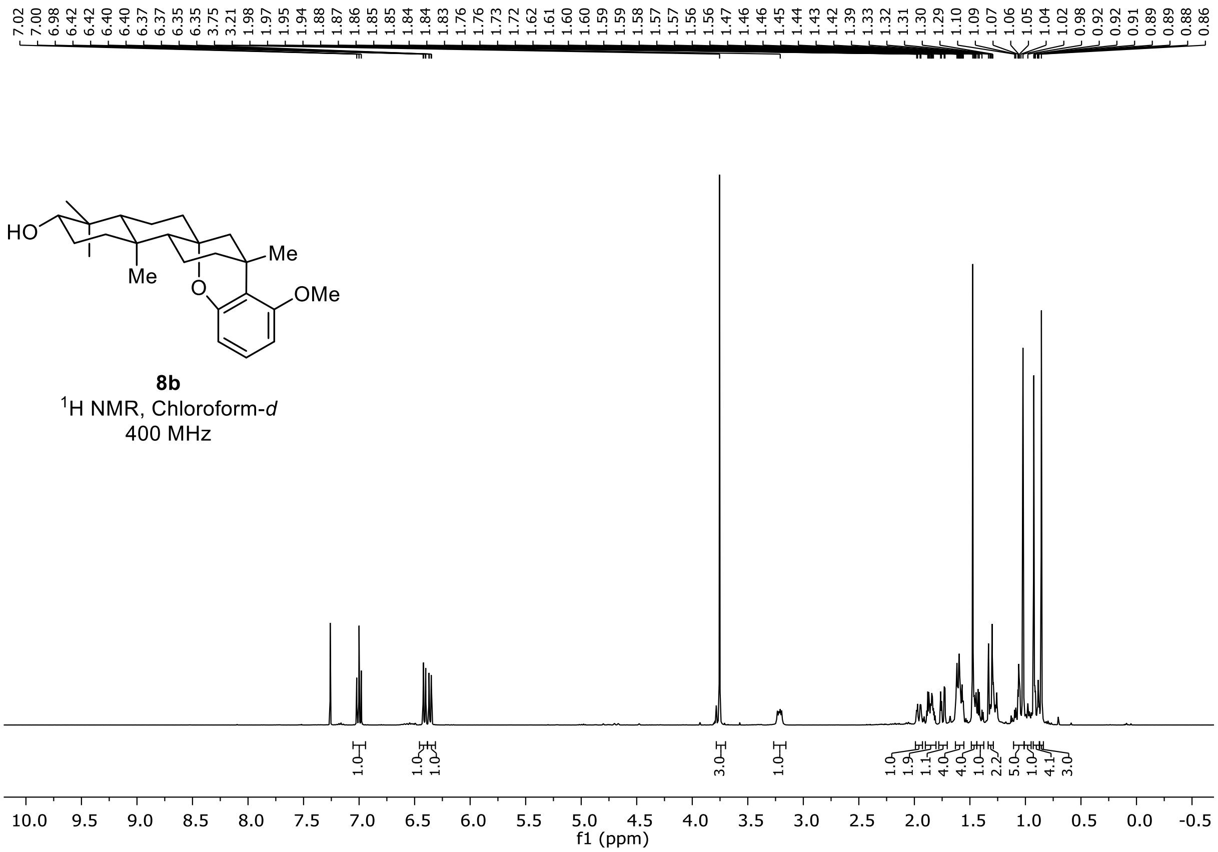
7.02 7.00 6.98 6.42 6.42 6.40 6.40 6.37 6.37 6.35 6.35 3.75 3.21 1.98 1.97 1.95 1.94 1.88 1.87 1.86 1.85 1.85 1.84 1.84 1.83 1.76 1.76 1.73 1.72 1.62 1.61 1.60 1.60 1.59 1.59 1.58 1.57 1.57 1.56 1.56 1.47 1.46 1.46 1.45 1.44 1.43 1.42 1.39 1.33 1.32 1.31 1.30 1.29 1.10 1.09 1.07 1.06 1.05 1.04 1.02 0.98 0.92 0.92 0.91 0.89 0.88 0.86
HO
Me
Me
O
OMe
8b
^{1}H NMR, Chloroform-*d*
400 MHz
1.0 1.0 1.0 3.0 1.0 1.0 1.9 1.1 4.0 4.0 1.0 2.2 5.0 1.0 4.1 3.0
10.0 9.5 9.0 8.5 8.0 7.5 7.0 6.5 6.0 5.5 5.0 4.5 4.0 3.5 3.0 2.5 2.0 1.5 1.0 0.5 0.0 -0.5
f1 (ppm)

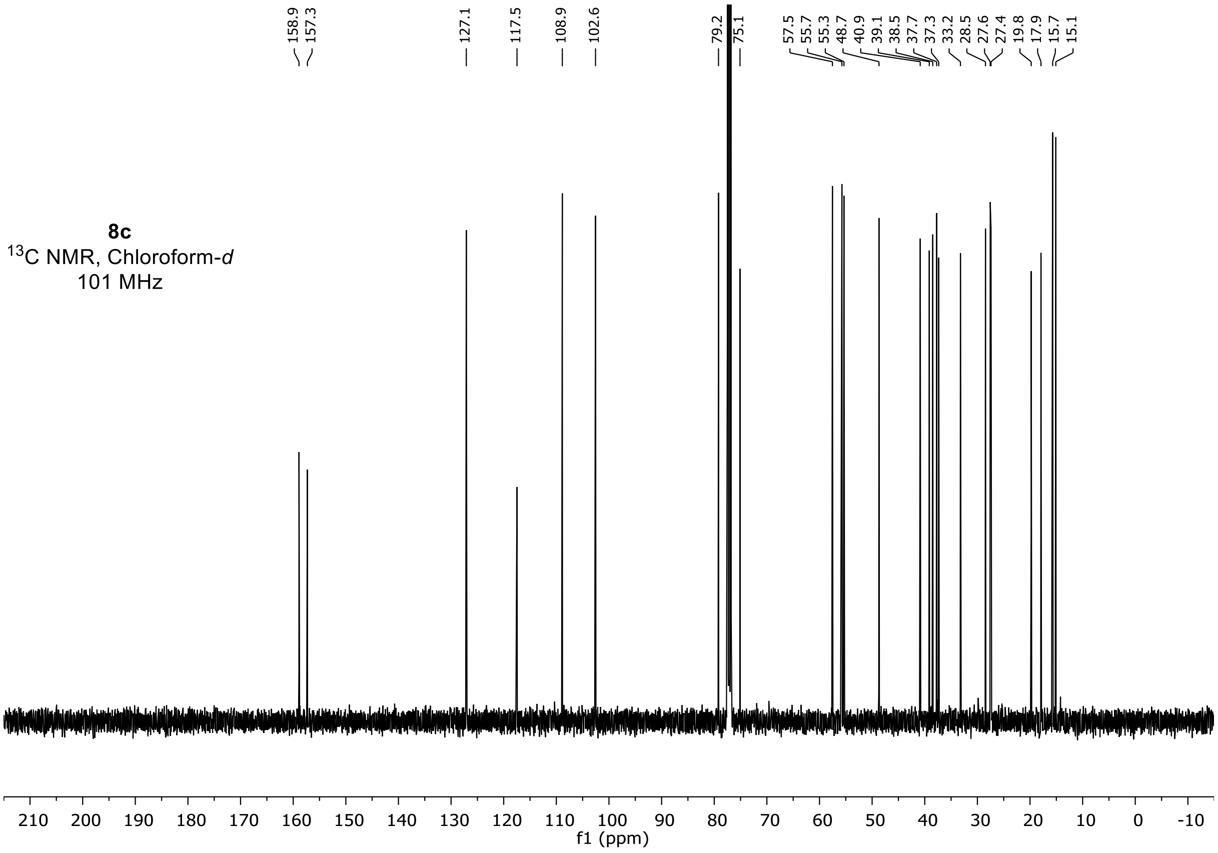
158.9 157.3 127.1 117.5 108.9 102.6 79.2 75.1 57.5 55.7 55.3 48.7 40.9 39.1 38.5 37.7 37.3 33.2 28.5 27.6 27.4 19.8 17.9 15.7 15.1
8c
^{13}C NMR, Chloroform-*d*
101 MHz
210 200 190 180 170 160 150 140 130 120 110 100 90 80 70 60 50 40 30 20 10 0 -10
f1 (ppm)

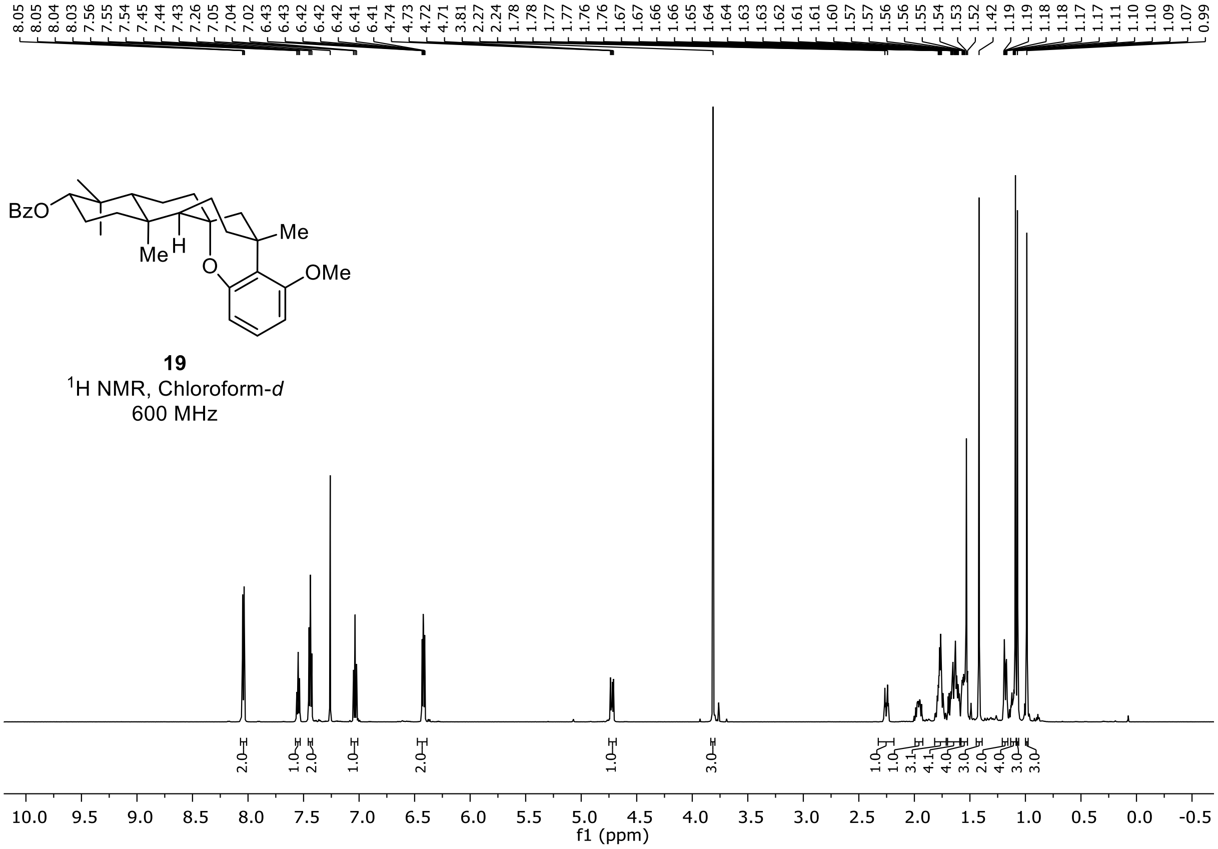

19
^{1}H NMR, Chloroform-*d*
600 MHz

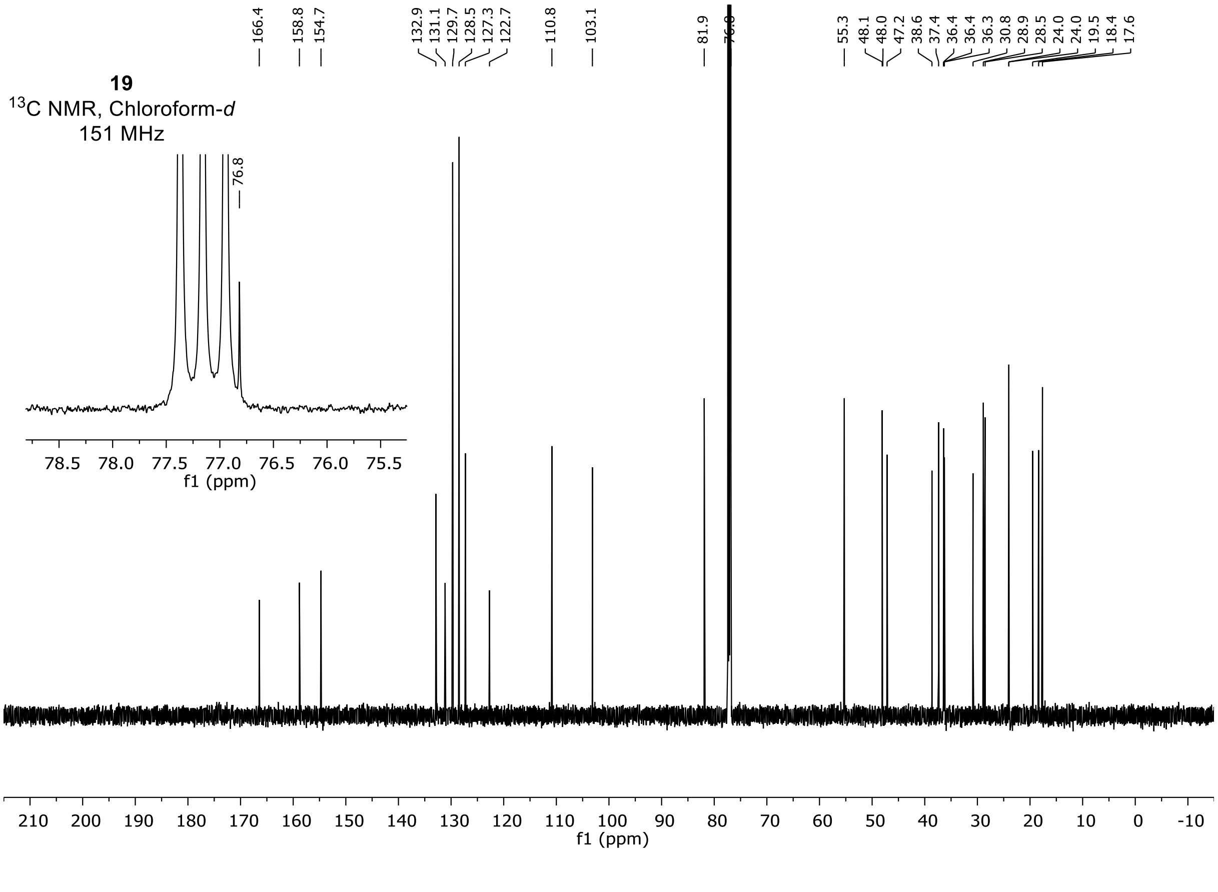

19
^{13}C NMR, Chloroform-*d*
151 MHz

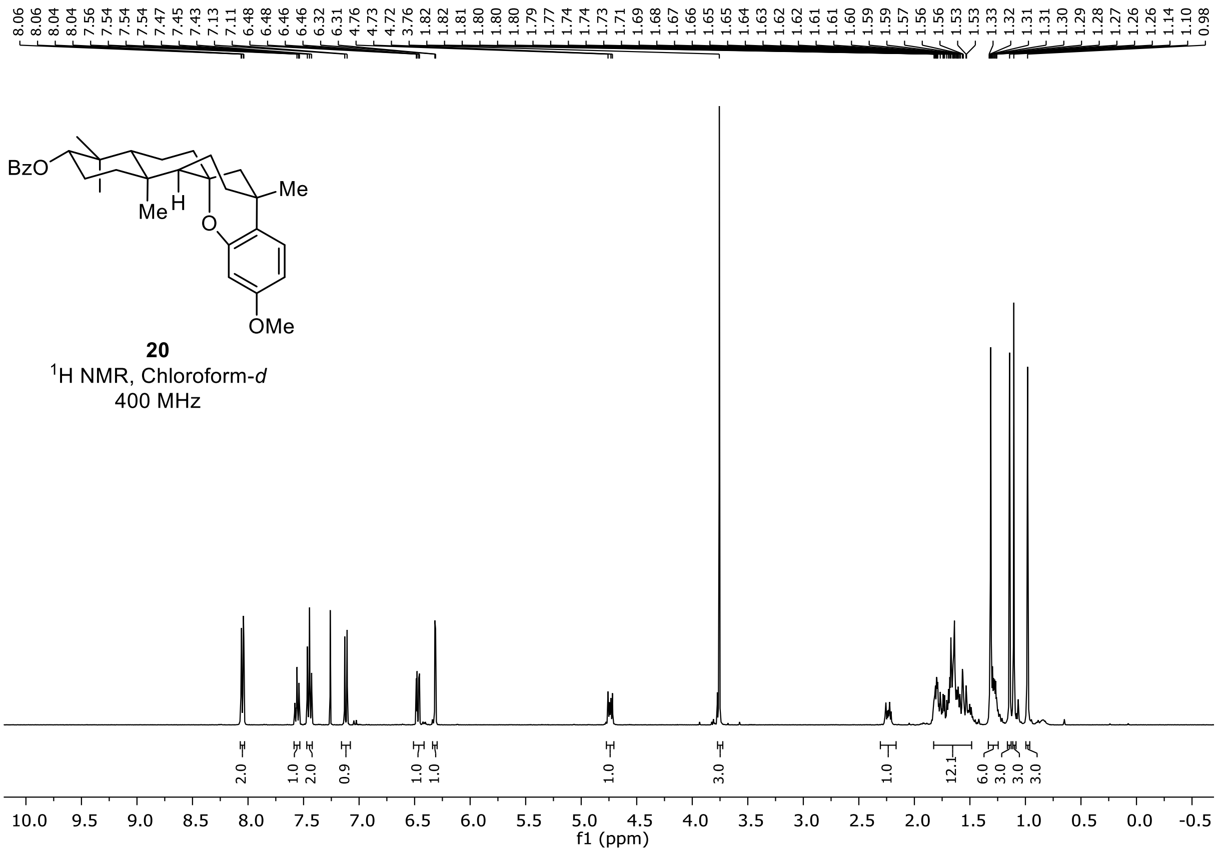

20
^{1}H NMR, Chloroform-*d*
400 MHz

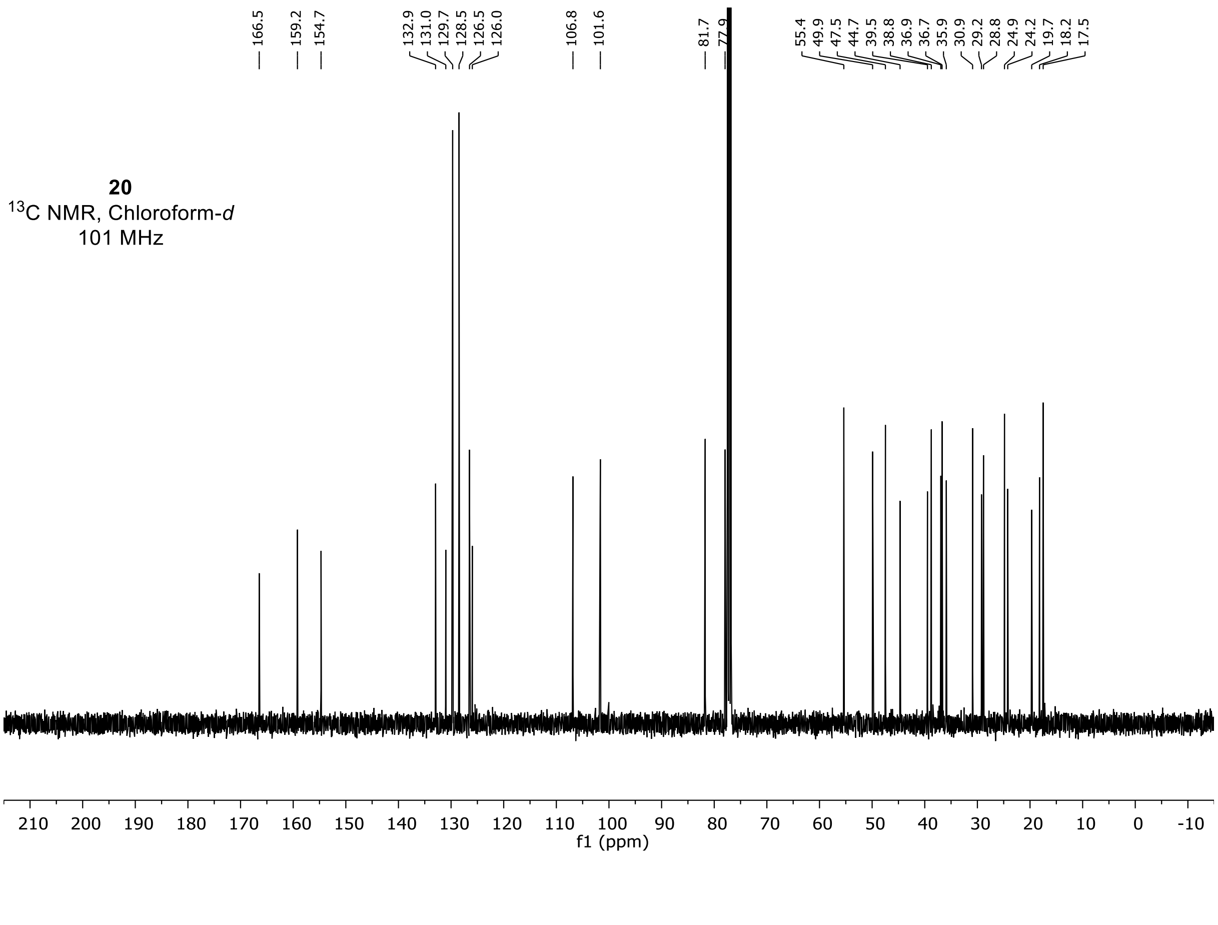

20
^{13}C NMR, Chloroform-*d*
101 MHz

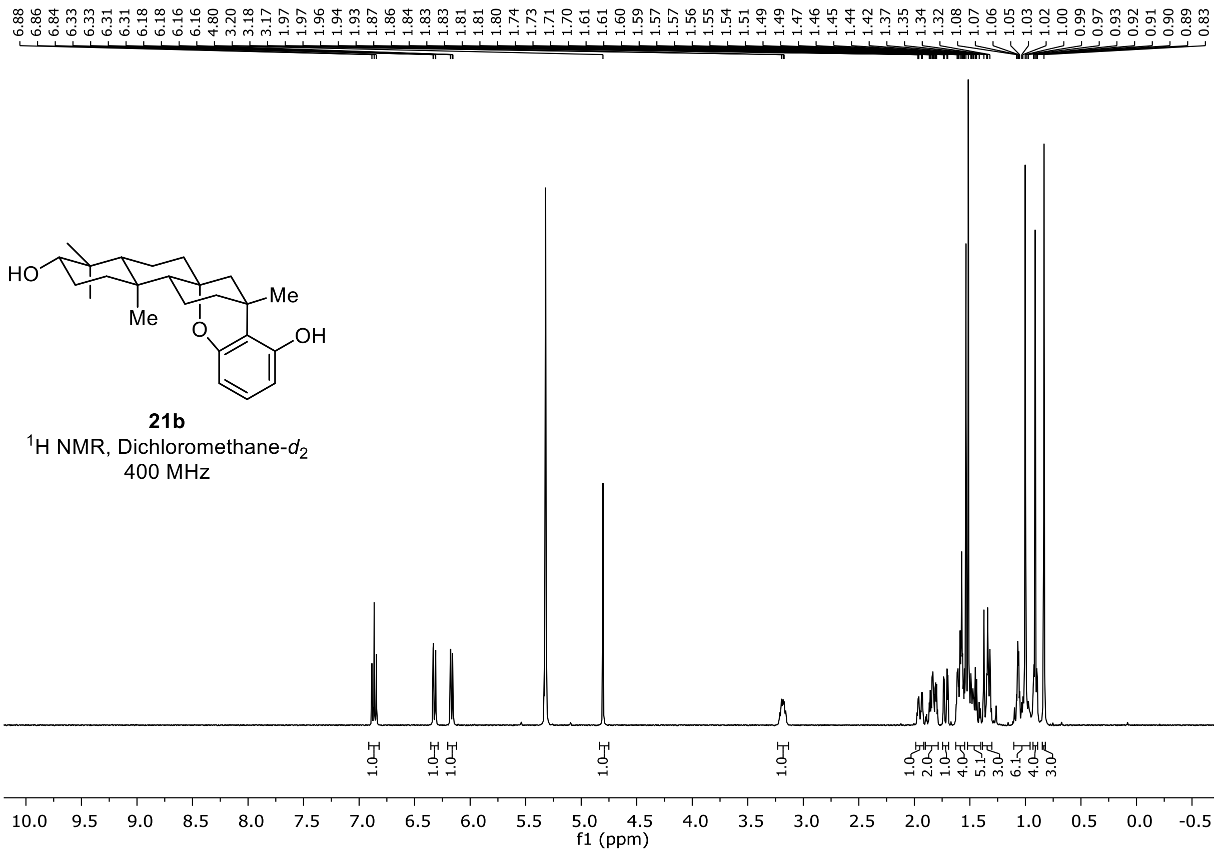
6.88 6.86 6.84 6.33 6.33 6.31 6.31 6.18 6.18 6.16 6.16 4.80 3.20 3.18 3.17 1.97 1.97 1.96 1.94 1.93 1.87 1.86 1.84 1.83 1.83 1.81 1.81 1.80 1.74 1.73 1.71 1.70 1.61 1.61 1.60 1.59 1.57 1.57 1.56 1.55 1.54 1.51 1.49 1.49 1.47 1.46 1.45 1.44 1.42 1.37 1.35 1.34 1.32 1.08 1.07 1.06 1.05 1.03 1.02 1.00 0.99 0.97 0.93 0.92 0.91 0.90 0.89 0.83
HO
Me
Me
O
OH
21b
^{1}H NMR, Dichloromethane-d_2
400 MHz
1.0 1.0 1.0 1.0 1.0 1.0 2.0 1.0 4.0 5.1 3.0 6.1 4.0 3.0
10.0 9.5 9.0 8.5 8.0 7.5 7.0 6.5 6.0 5.5 5.0 4.5 4.0 3.5 3.0 2.5 2.0 1.5 1.0 0.5 0.0 -0.5
f1 (ppm)

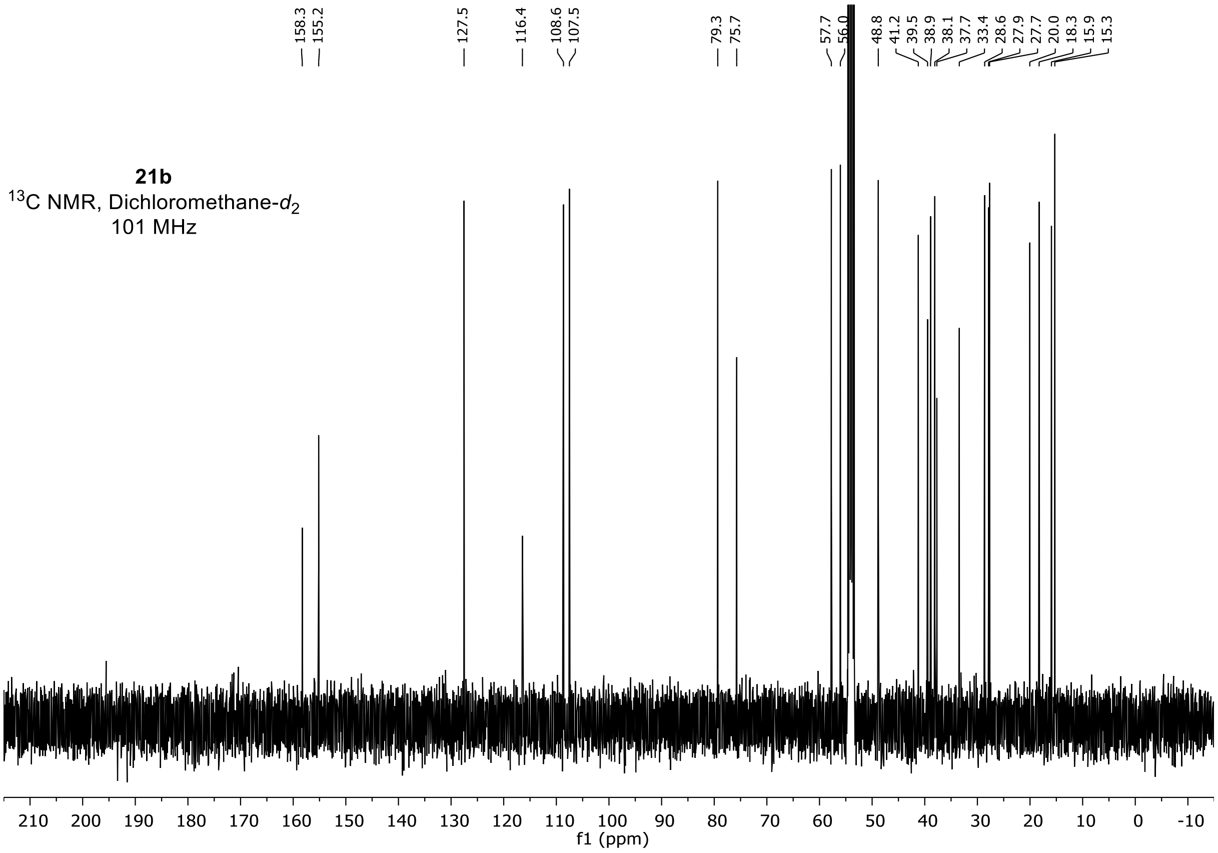
158.3 155.2 127.5 116.4 108.6 107.5 79.3 75.7 57.7 56.0 48.8 41.2 39.5 38.9 38.1 37.7 33.4 28.6 27.9 27.7 20.0 18.3 15.9 15.3
21b
^{13}C NMR, Dichloromethane-d_2
101 MHz
210 200 190 180 170 160 150 140 130 120 110 100 90 80 70 60 50 40 30 20 10 0 -10
f1 (ppm)

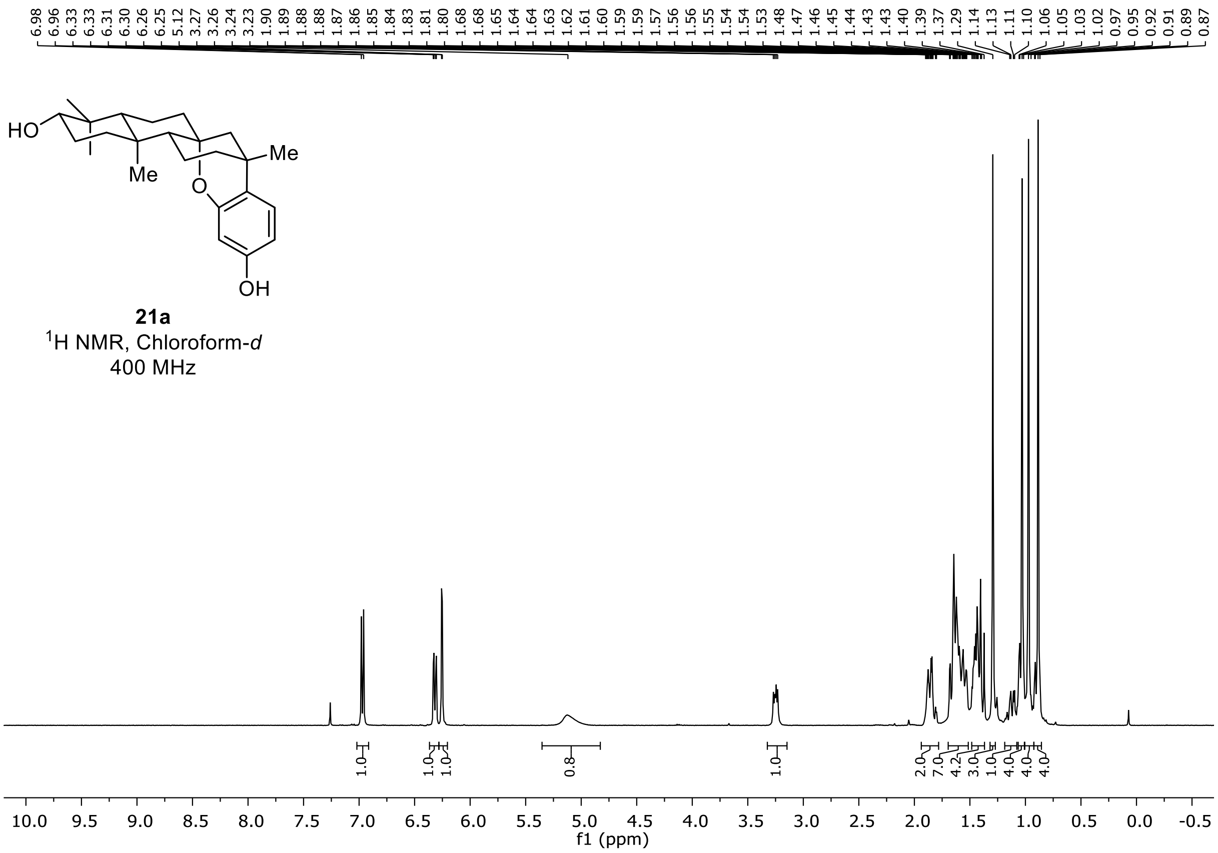

21a
^{1}H NMR, Chloroform-*d*
400 MHz

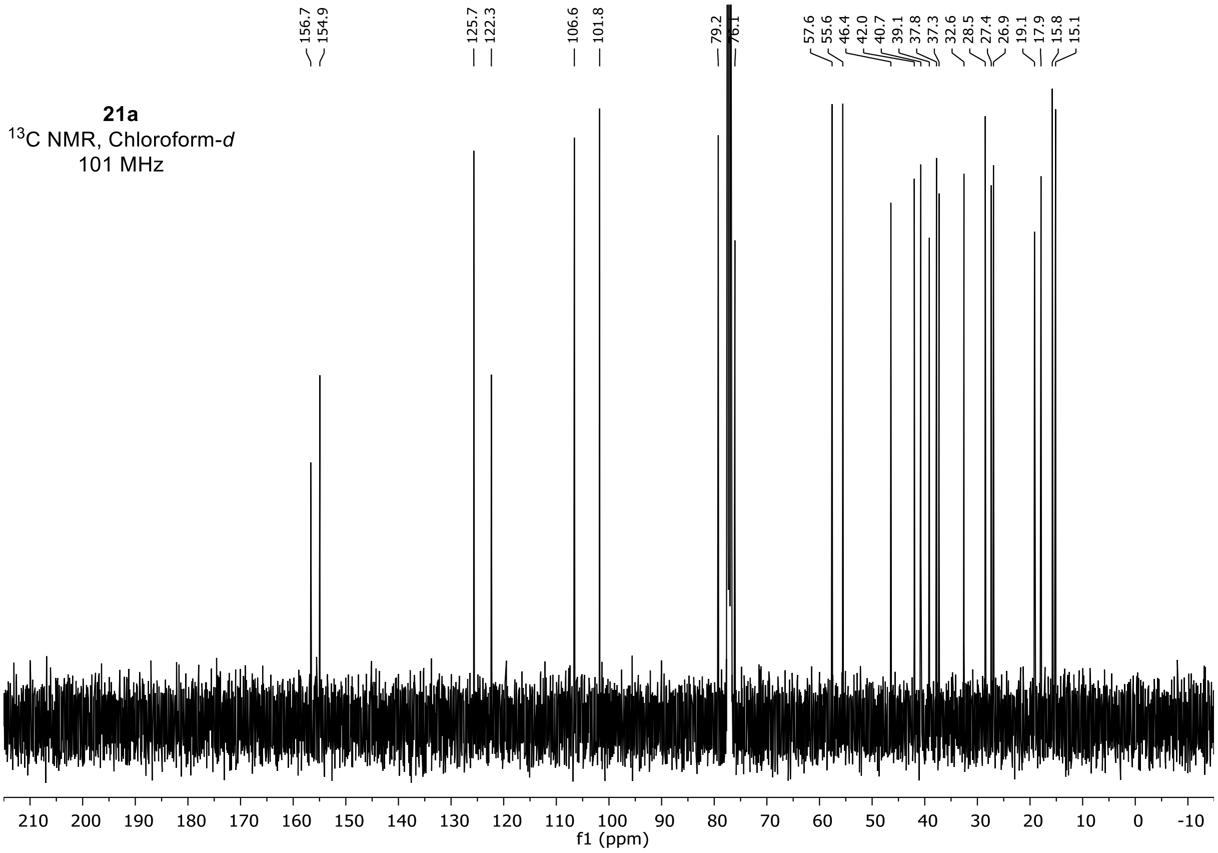

21a
^{13}C NMR, Chloroform-*d*
101 MHz

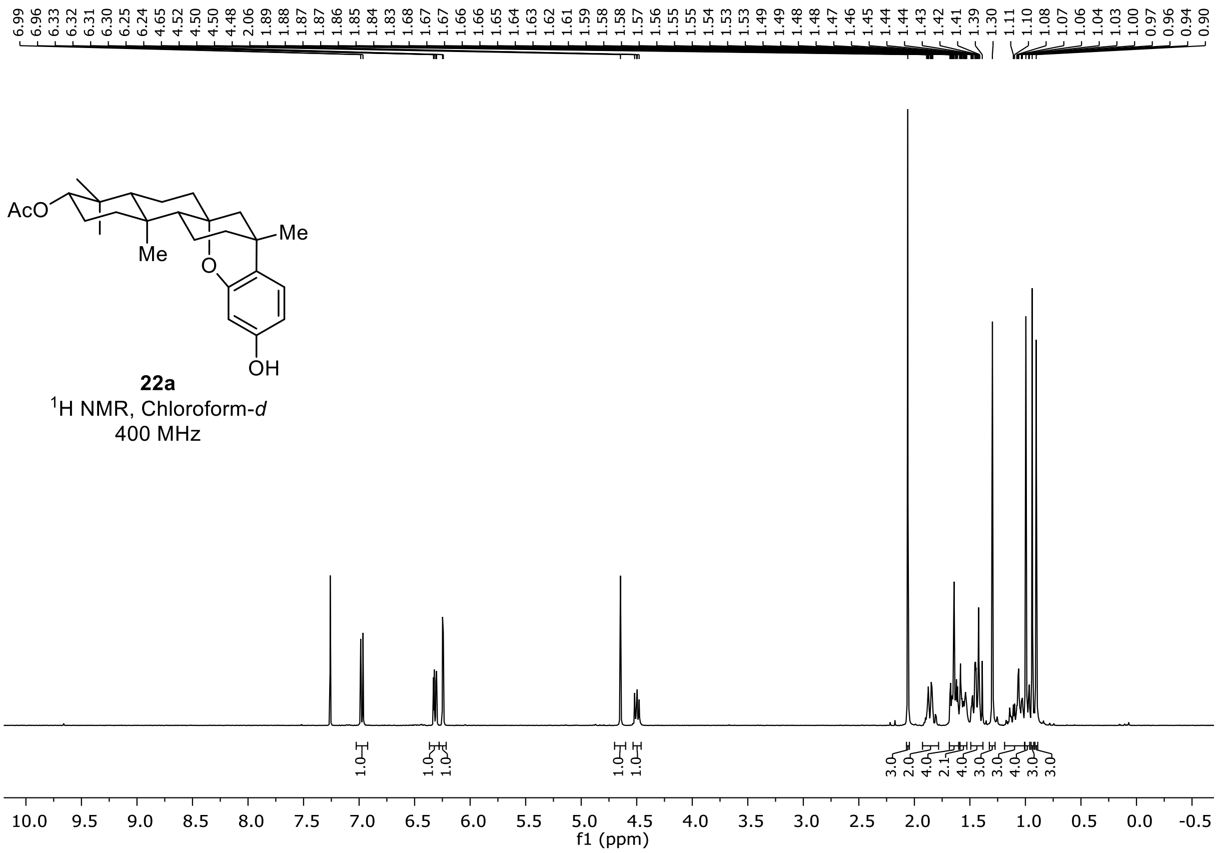
AcO
Me
Me
O
OH
22a
^{1}H NMR, Chloroform-*d*
400 MHz
f1 (ppm)

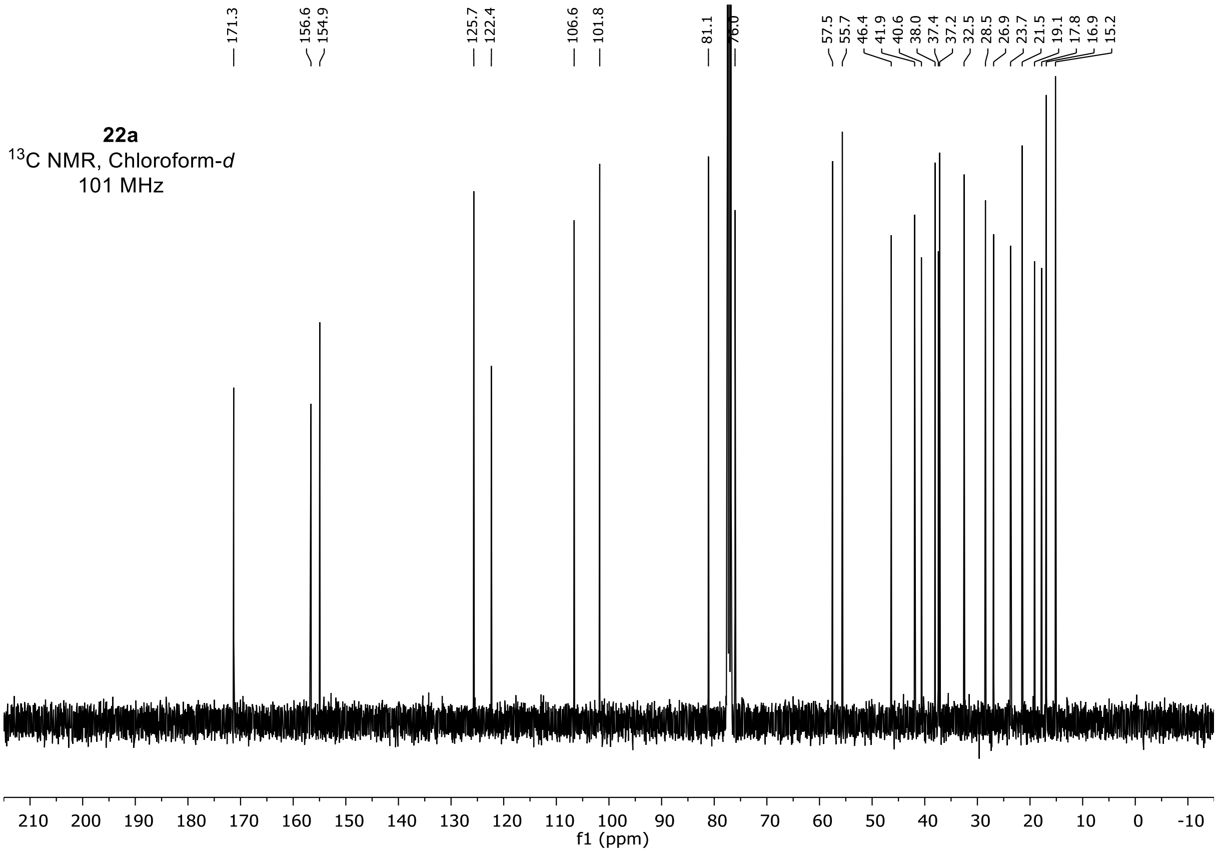
22a
^{13}C NMR, Chloroform-*d*
101 MHz
f1 (ppm)

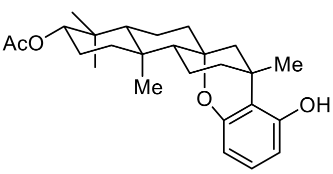

22b
^{1}H NMR, Chloroform-*d*
400 MHz

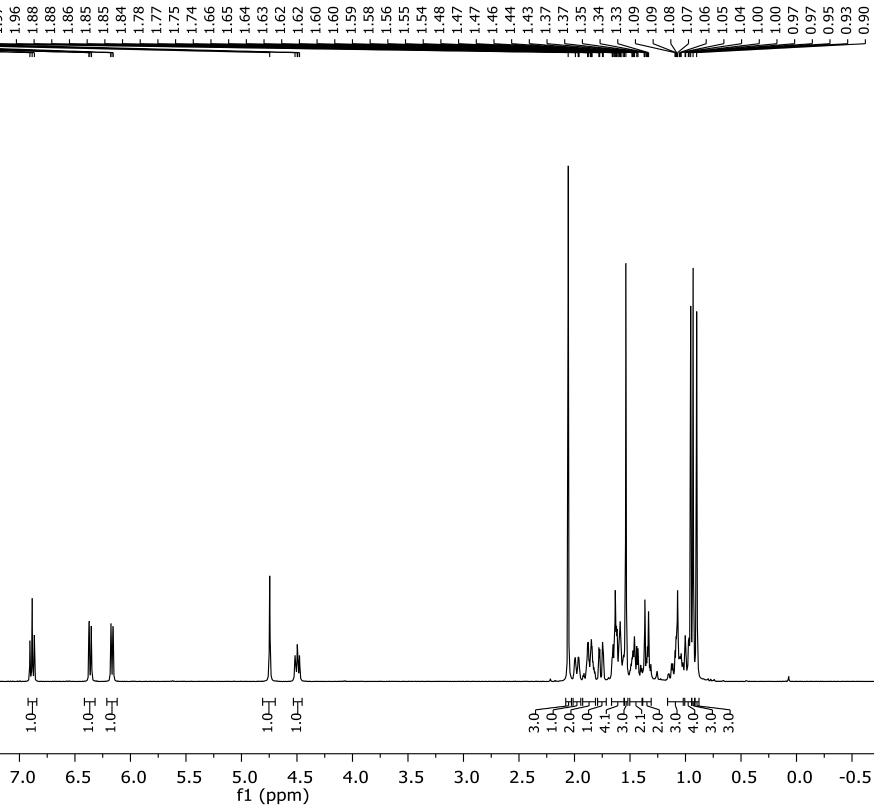

22b
^{13}C NMR, Chloroform-*d*
101 MHz

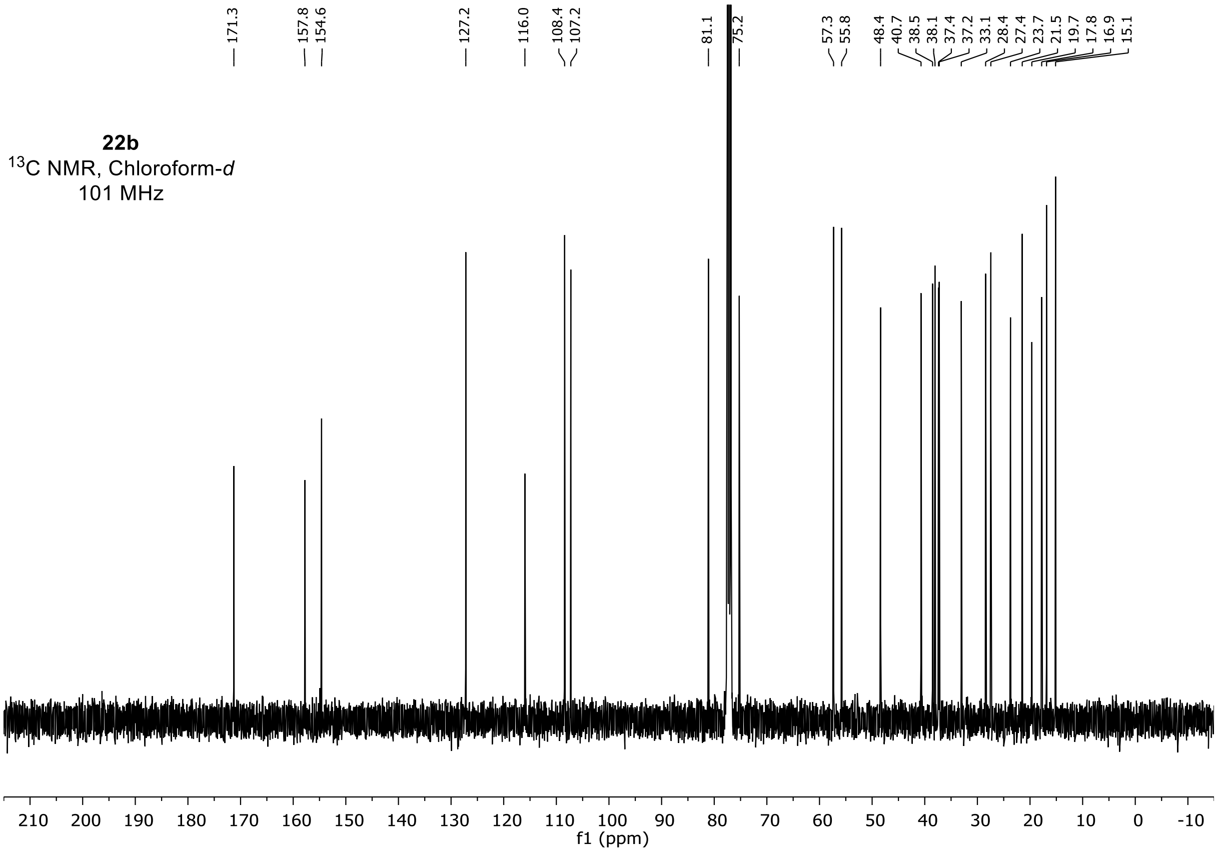

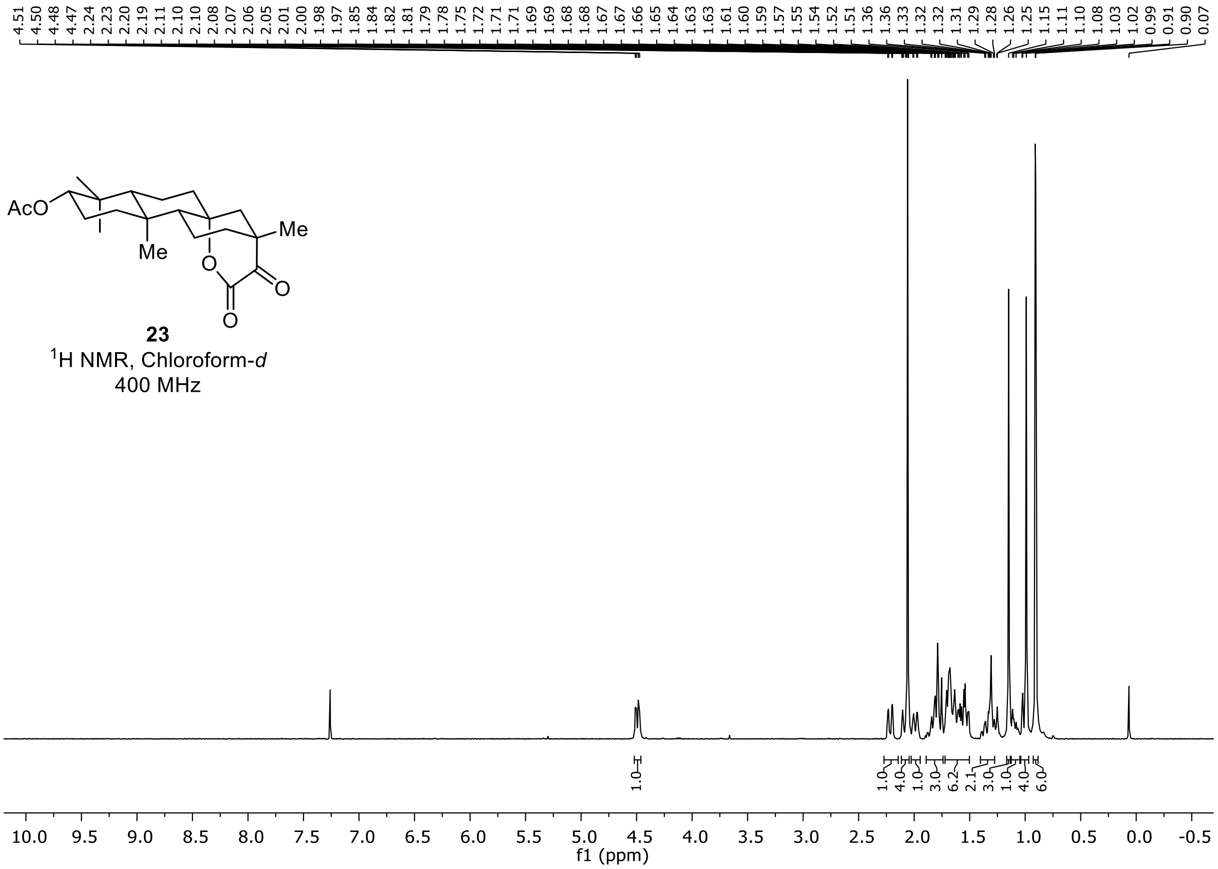

23
^{1}H NMR, Chloroform-*d*
400 MHz

23
^{13}C NMR, Chloroform-*d*
101 MHz

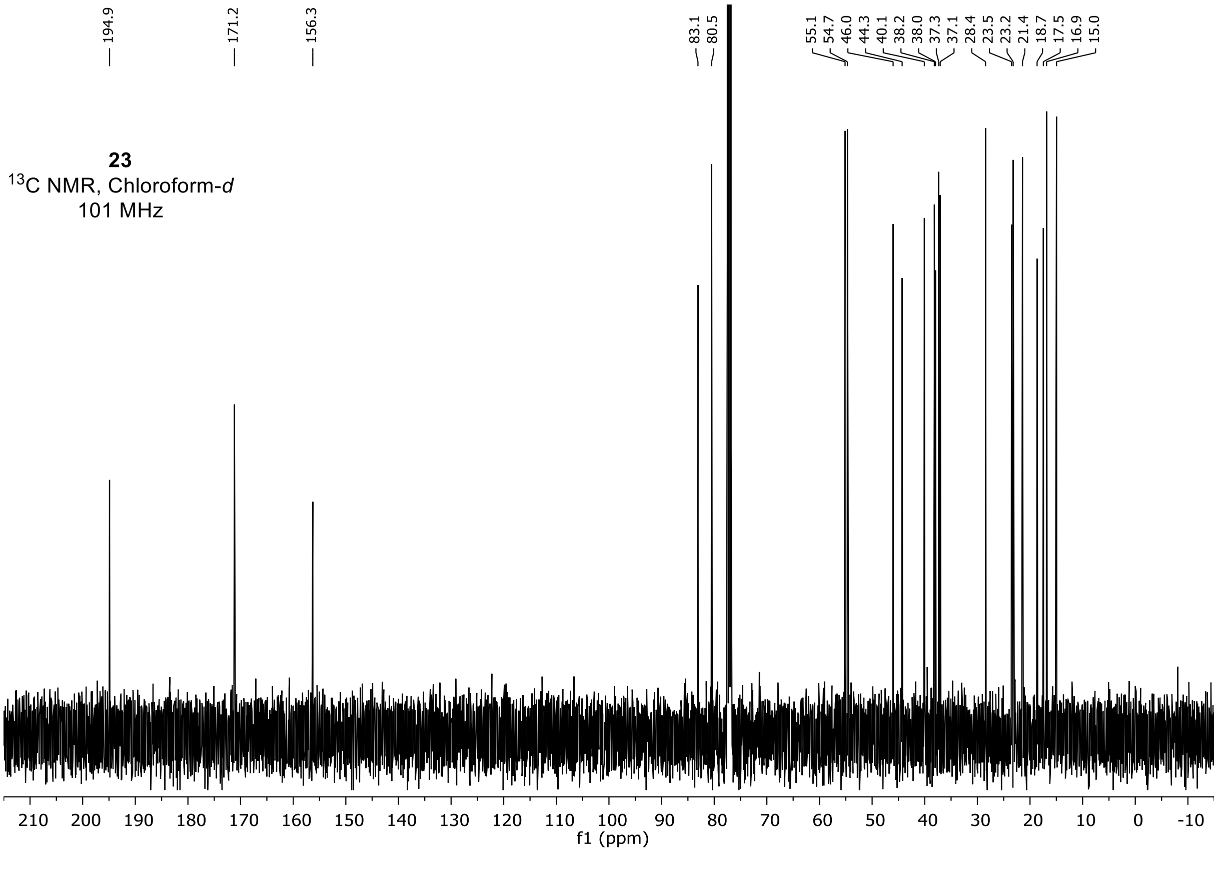

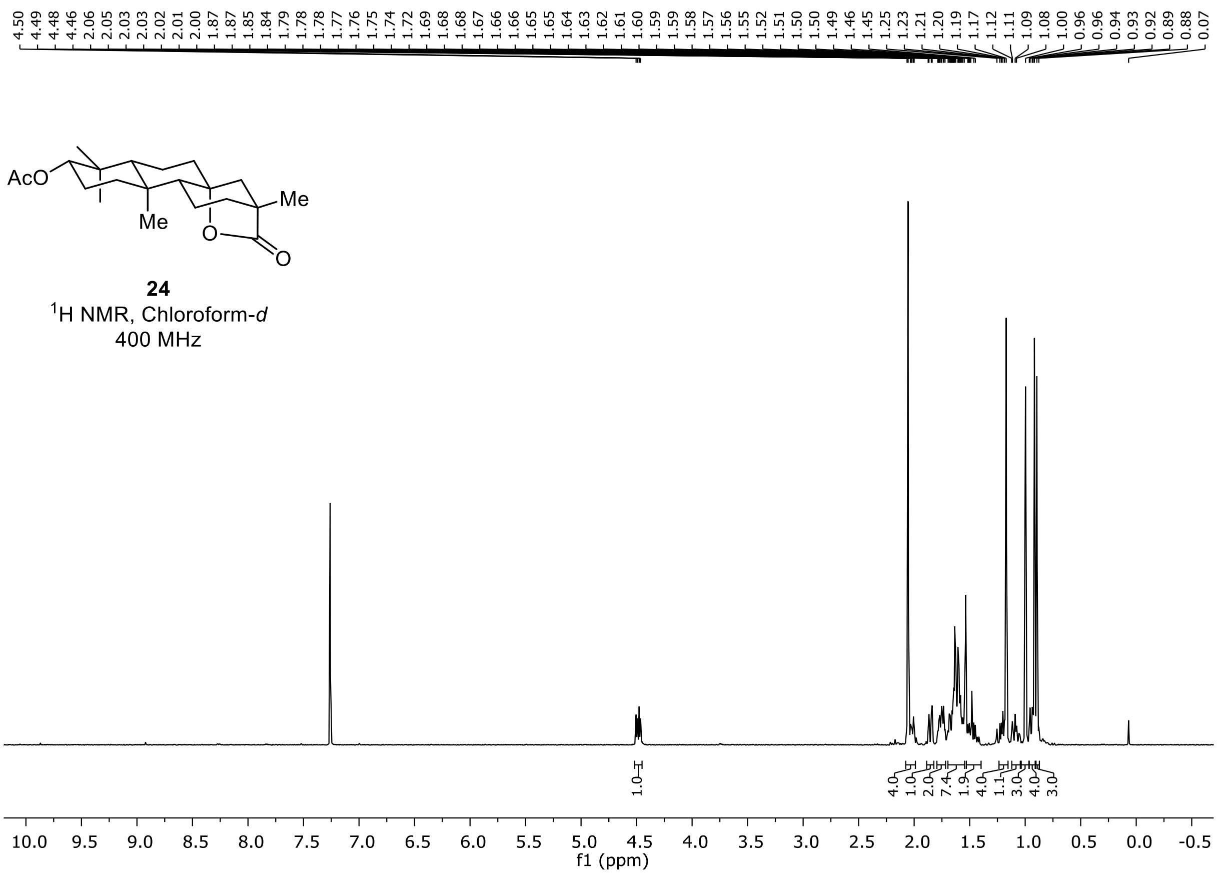
AcO
Me
Me
O
O
24
^{1}H NMR, Chloroform-*d*
400 MHz
Peaks (ppm): 4.50, 4.49, 4.48, 4.46, 2.06, 2.05, 2.03, 2.03, 2.02, 2.01, 2.00, 1.87, 1.87, 1.85, 1.84, 1.79, 1.78, 1.78, 1.77, 1.76, 1.75, 1.74, 1.72, 1.69, 1.68, 1.68, 1.67, 1.66, 1.66, 1.65, 1.65, 1.64, 1.63, 1.62, 1.61, 1.60, 1.59, 1.59, 1.58, 1.57, 1.56, 1.55, 1.52, 1.51, 1.50, 1.50, 1.49, 1.46, 1.45, 1.25, 1.23, 1.21, 1.20, 1.19, 1.17, 1.12, 1.11, 1.09, 1.08, 1.00, 0.96, 0.96, 0.94, 0.93, 0.92, 0.89, 0.88, 0.07
Integrals: 1.0, 4.0, 1.0, 2.0, 7.4, 1.9, 4.0, 1.1, 3.0, 4.0, 3.0
f1 (ppm)

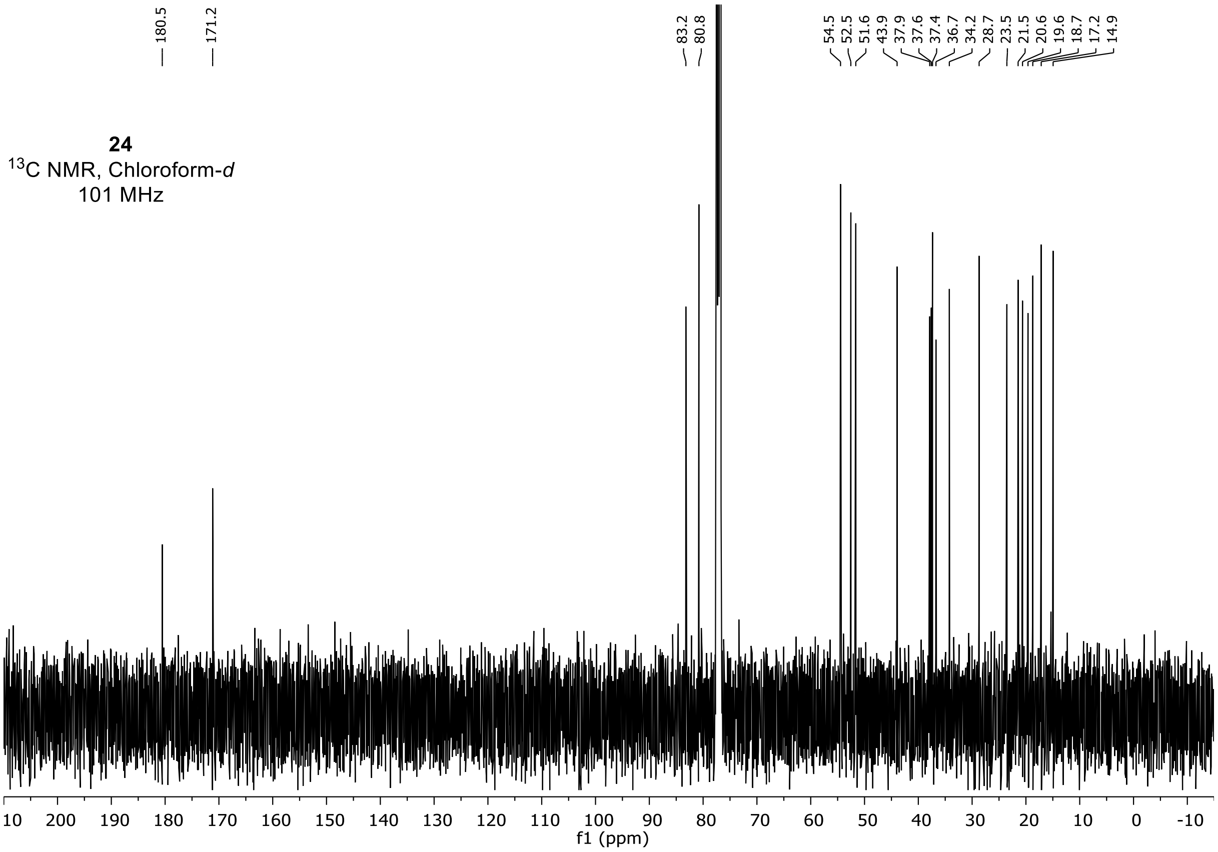
24
^{13}C NMR, Chloroform-*d*
101 MHz
Peaks (ppm): 180.5, 171.2, 83.2, 80.8, 54.5, 52.5, 51.6, 43.9, 37.9, 37.6, 37.4, 36.7, 34.2, 28.7, 23.5, 21.5, 20.6, 19.6, 18.7, 17.2, 14.9
f1 (ppm)

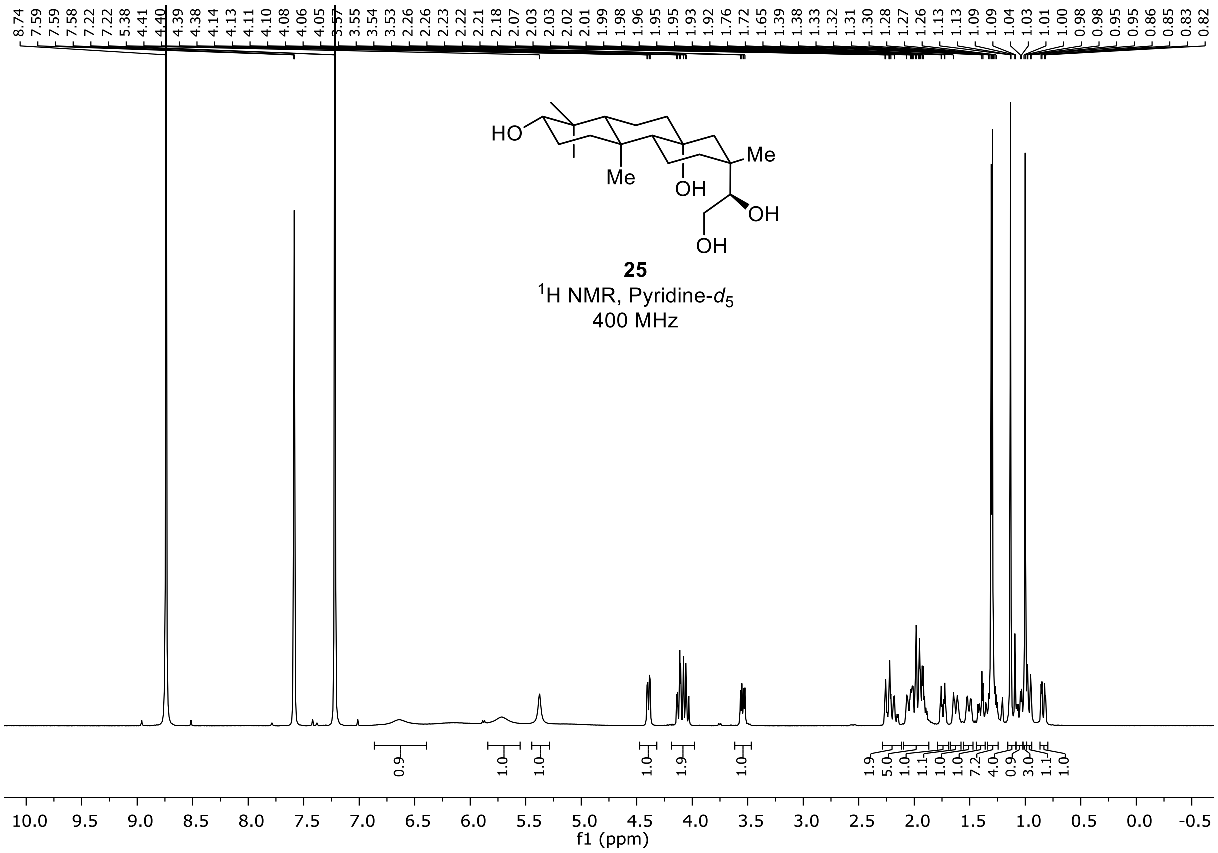
8.74 7.59 7.59 7.58 7.22 7.22 5.38 4.41 4.40 4.39 4.38 4.14 4.13 4.11 4.10 4.08 4.06 4.05 3.57 3.55 3.54 3.53 2.26 2.26 2.23 2.22 2.22 2.21 2.18 2.07 2.03 2.03 2.02 2.01 1.99 1.98 1.96 1.95 1.95 1.93 1.92 1.76 1.72 1.65 1.39 1.38 1.33 1.32 1.31 1.30 1.28 1.27 1.26 1.13 1.13 1.09 1.04 1.03 1.01 1.00 0.98 0.98 0.95 0.95 0.86 0.85 0.83 0.82
HO
Me
Me
OH
OH
OH
25
^{1}H NMR, Pyridine-d_5
400 MHz
0.9 1.0 1.0 1.0 1.9 1.0 1.9 5.0 1.0 1.1 1.0 1.0 7.2 4.0 0.9 3.0 1.1 1.0
10.0 9.5 9.0 8.5 8.0 7.5 7.0 6.5 6.0 5.5 5.0 4.5 4.0 3.5 3.0 2.5 2.0 1.5 1.0 0.5 0.0 -0.5
f1 (ppm)

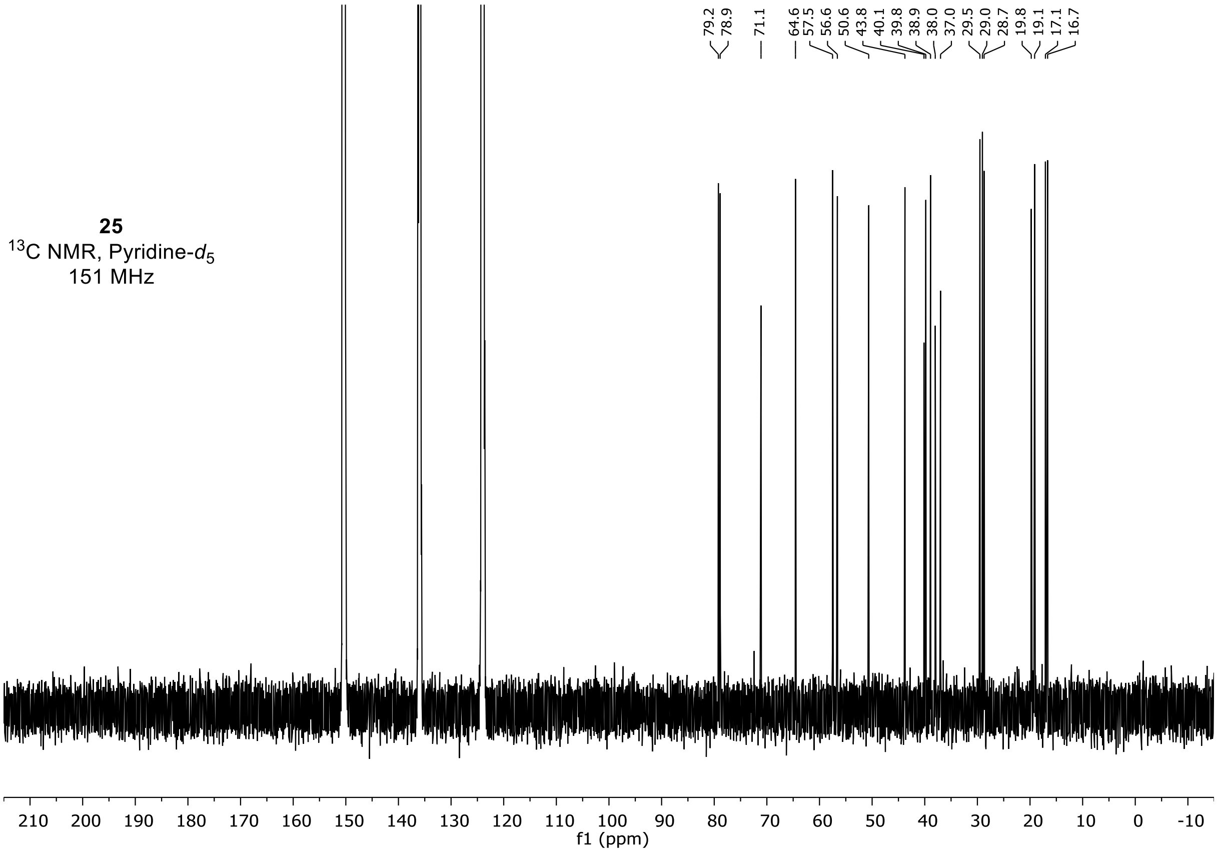
79.2 78.9 71.1 64.6 57.5 56.6 50.6 43.8 40.1 39.8 38.9 38.0 37.0 29.5 29.0 28.7 19.8 19.1 17.1 16.7
25
^{13}C NMR, Pyridine-d_5
151 MHz
210 200 190 180 170 160 150 140 130 120 110 100 90 80 70 60 50 40 30 20 10 0 -10
f1 (ppm)

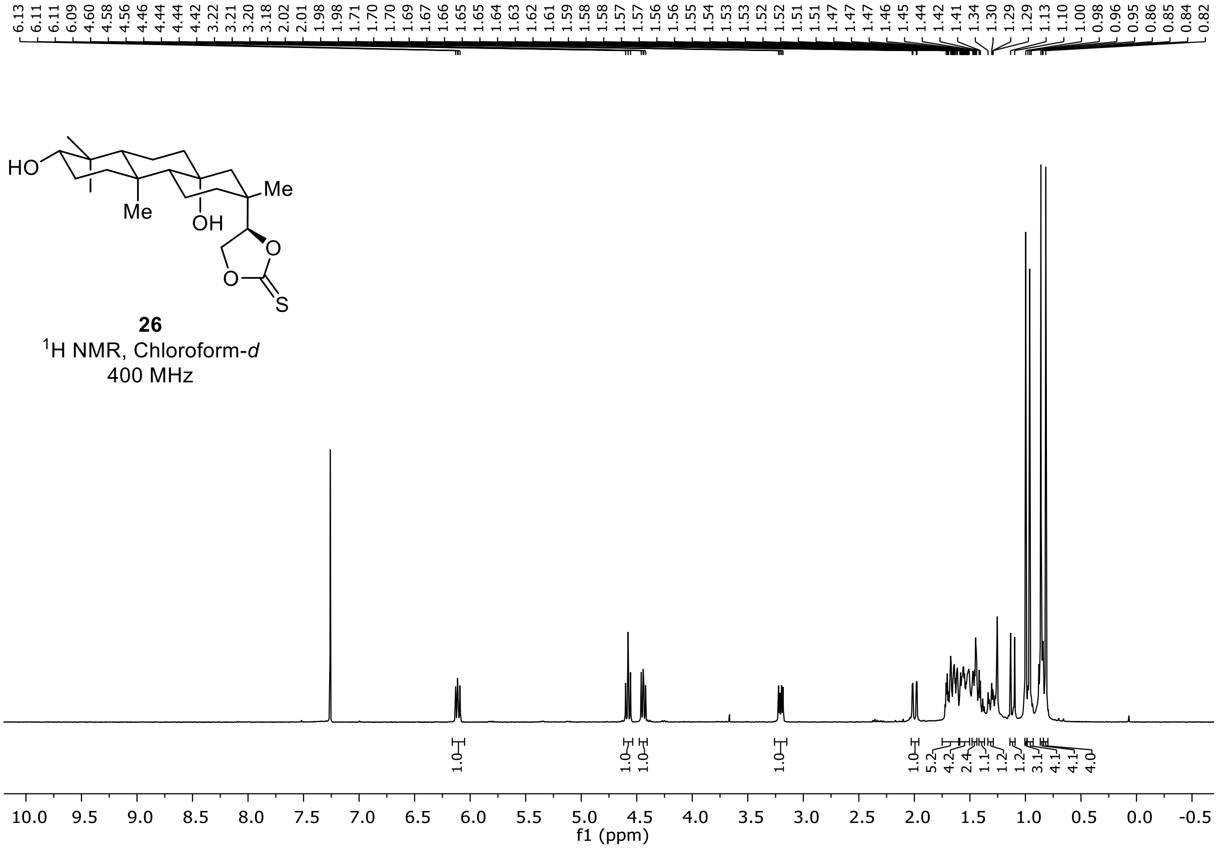

26
^{1}H NMR, Chloroform-*d*
400 MHz

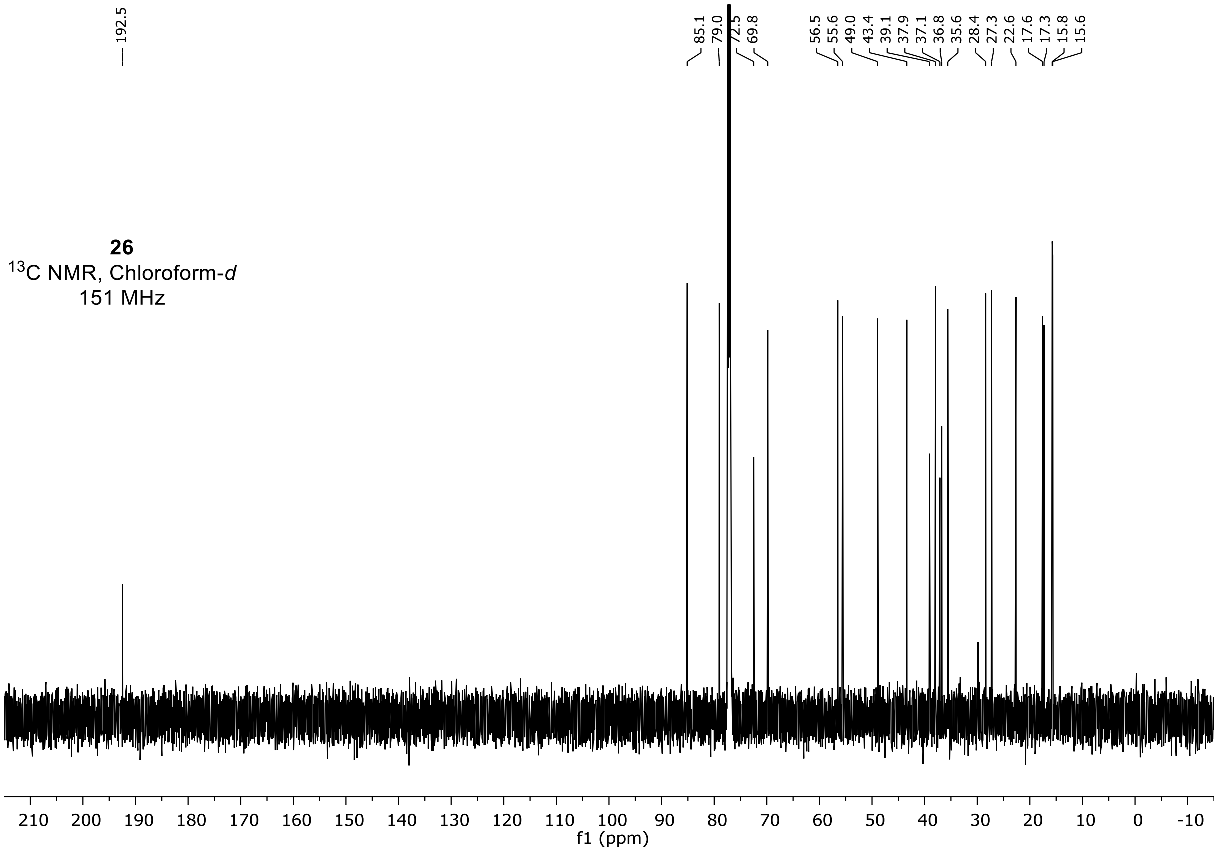

26
^{13}C NMR, Chloroform-*d*
151 MHz

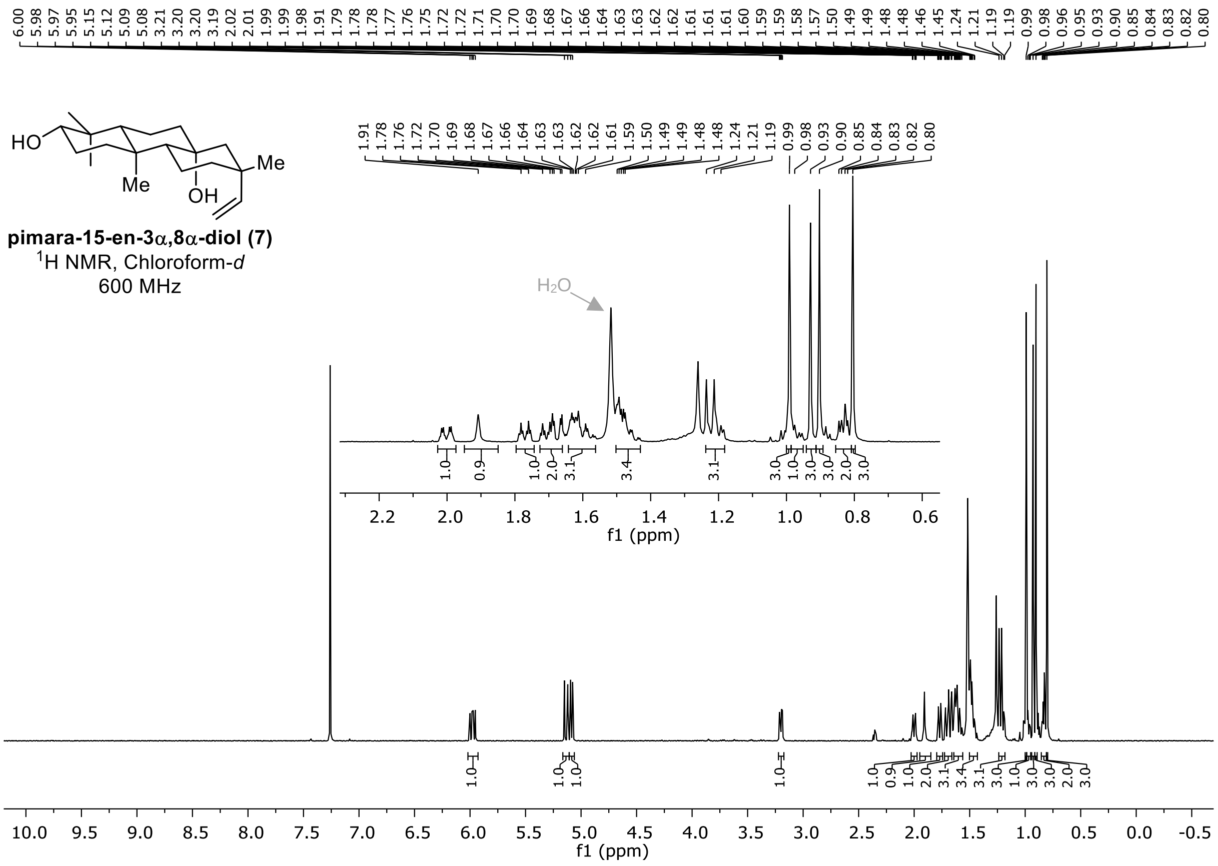
HO
Me
Me
OH
pimara-15-en-3α,8α-diol (7)
1H NMR, Chloroform-d
600 MHz
H2O
f1 (ppm)

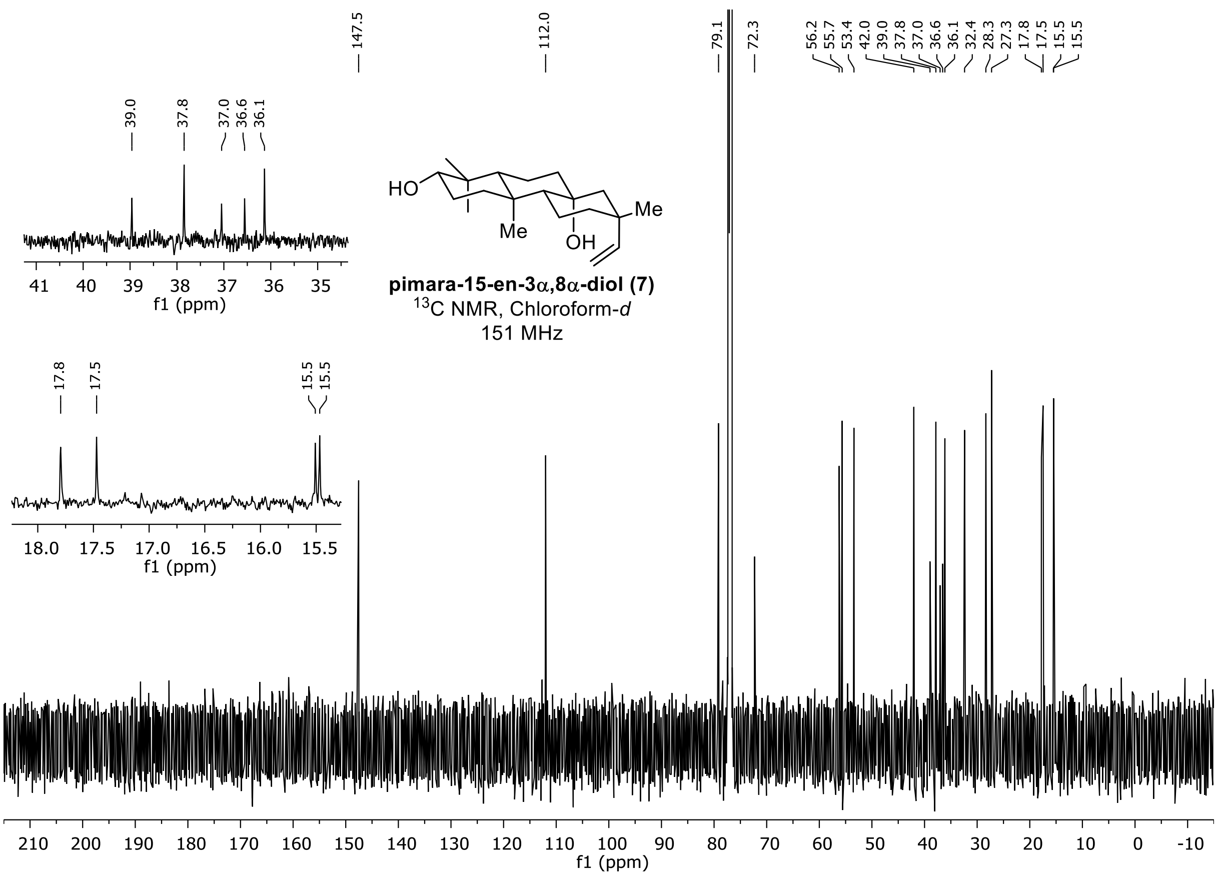
147.5
112.0
79.1
72.3
56.2
55.7
53.4
42.0
39.0
37.8
37.0
36.6
36.1
32.4
28.3
27.3
17.8
17.5
15.5
15.5
HO
Me
Me
OH
pimara-15-en-3α,8α-diol (7)
13C NMR, Chloroform-d
151 MHz
f1 (ppm)

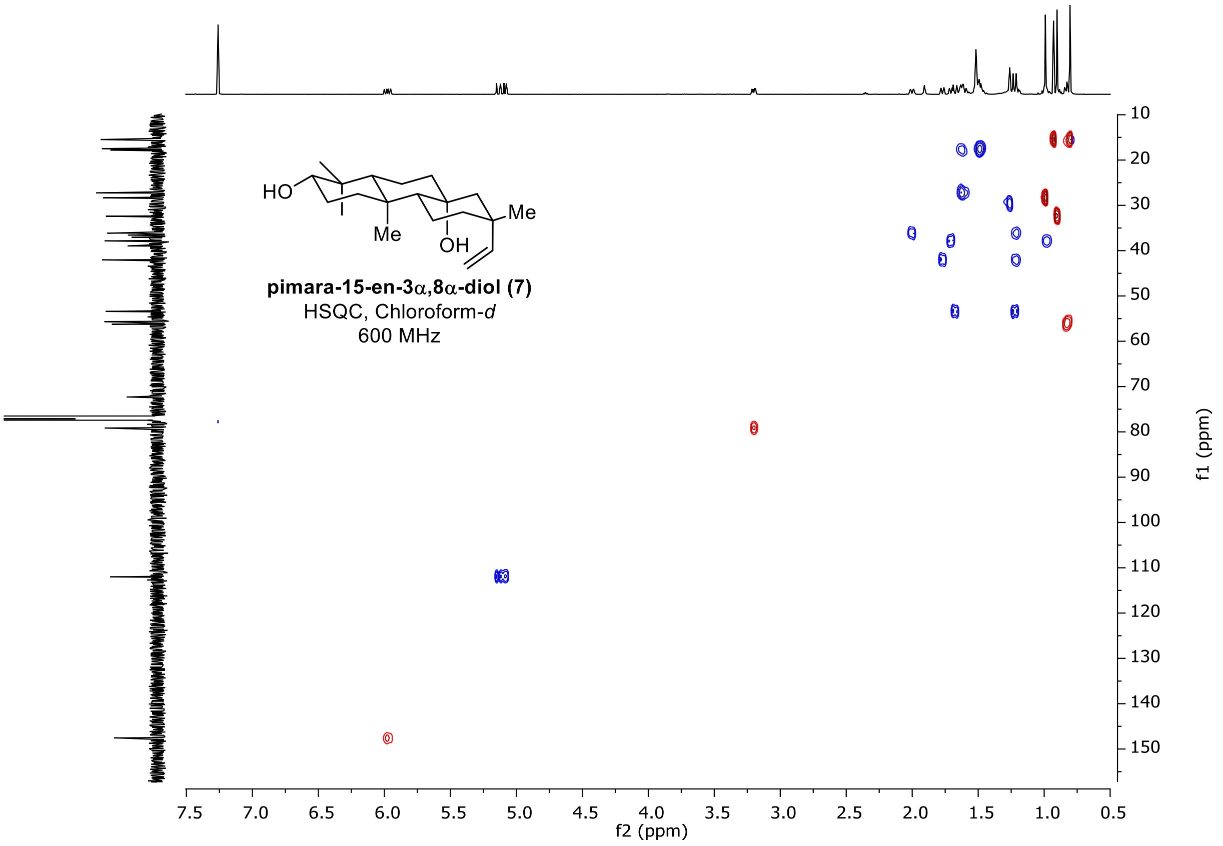

Extension of the HSQC spectrum above:

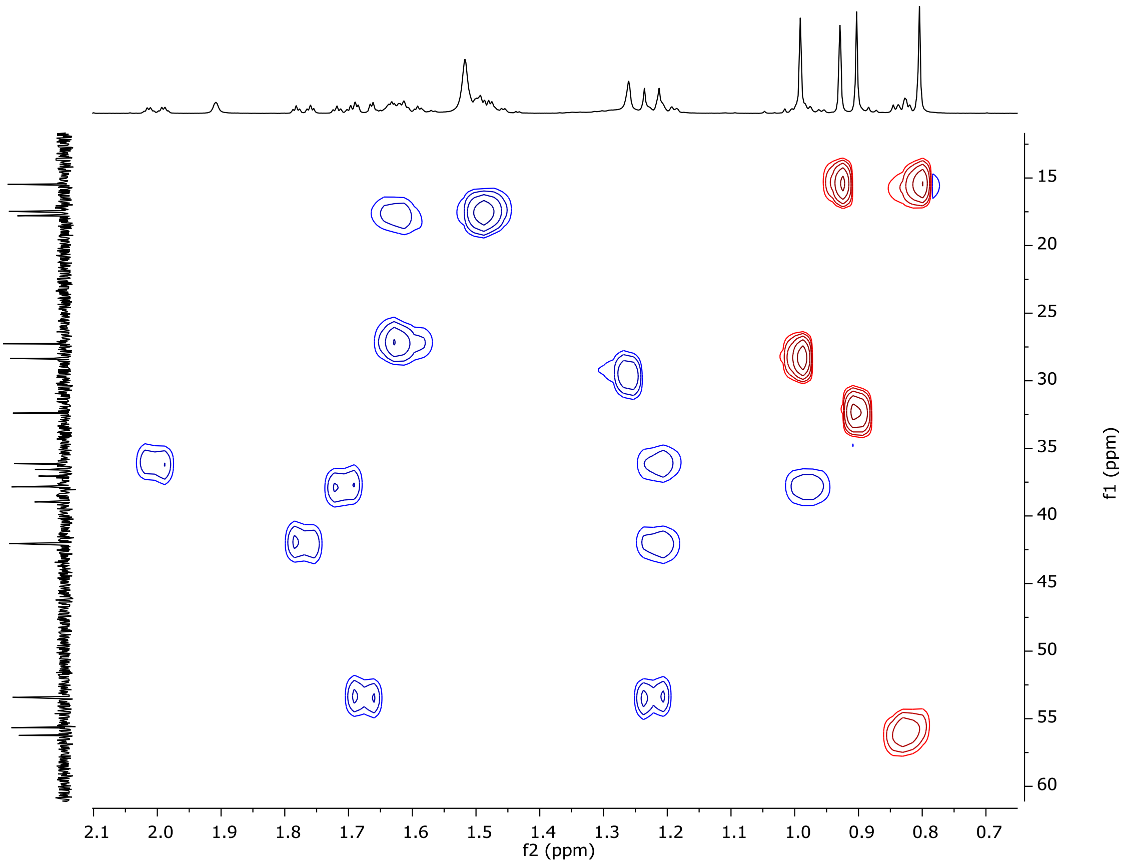

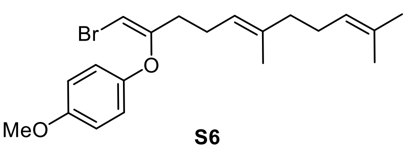

S6
^{1}H NMR, Chloroform-*d*
400 MHz

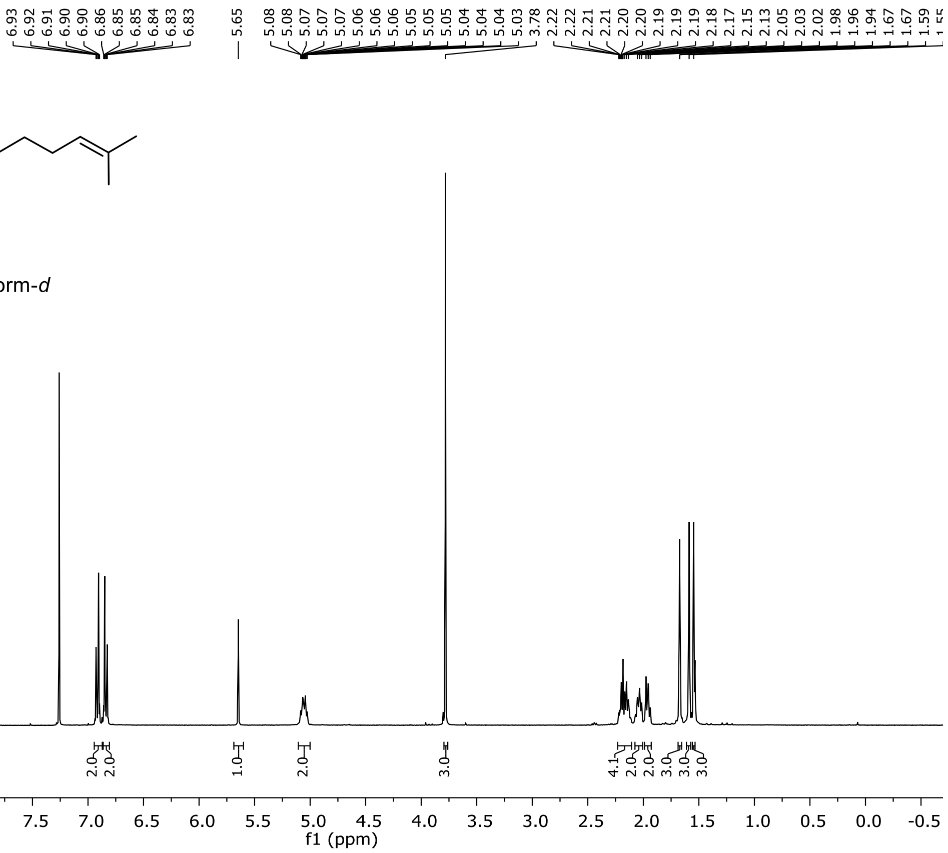

S6
^{13}C NMR, Benzene-d_6
101 MHz

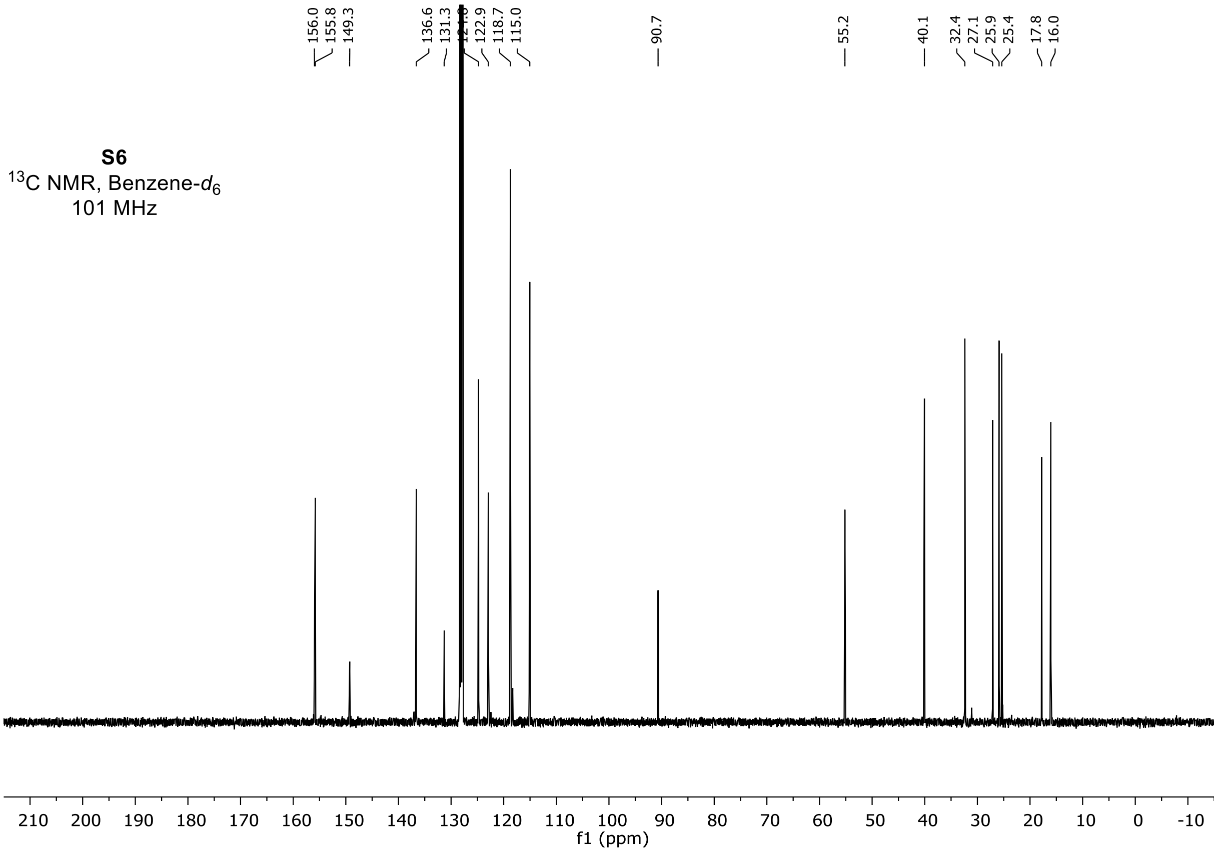

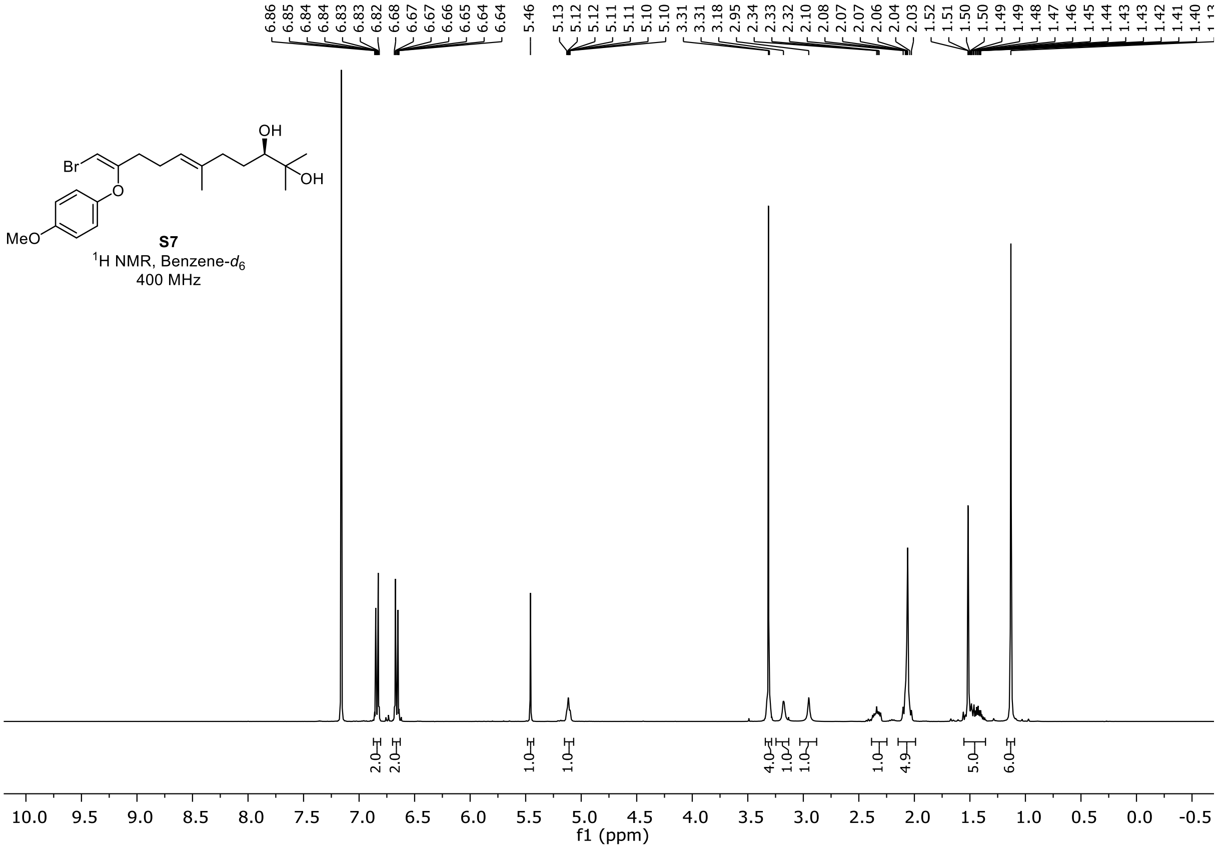

S7
^{1}H NMR, Benzene-d_6
400 MHz

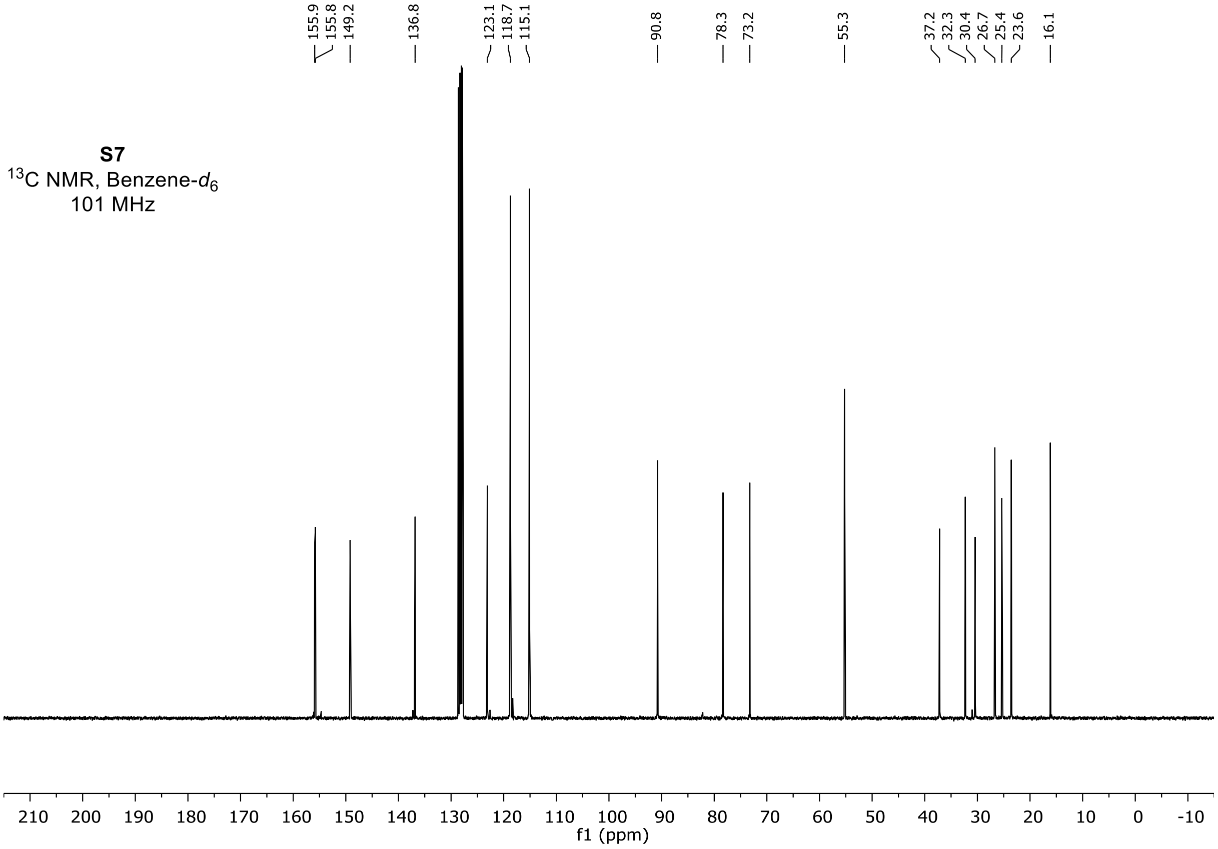

S7
^{13}C NMR, Benzene-d_6
101 MHz

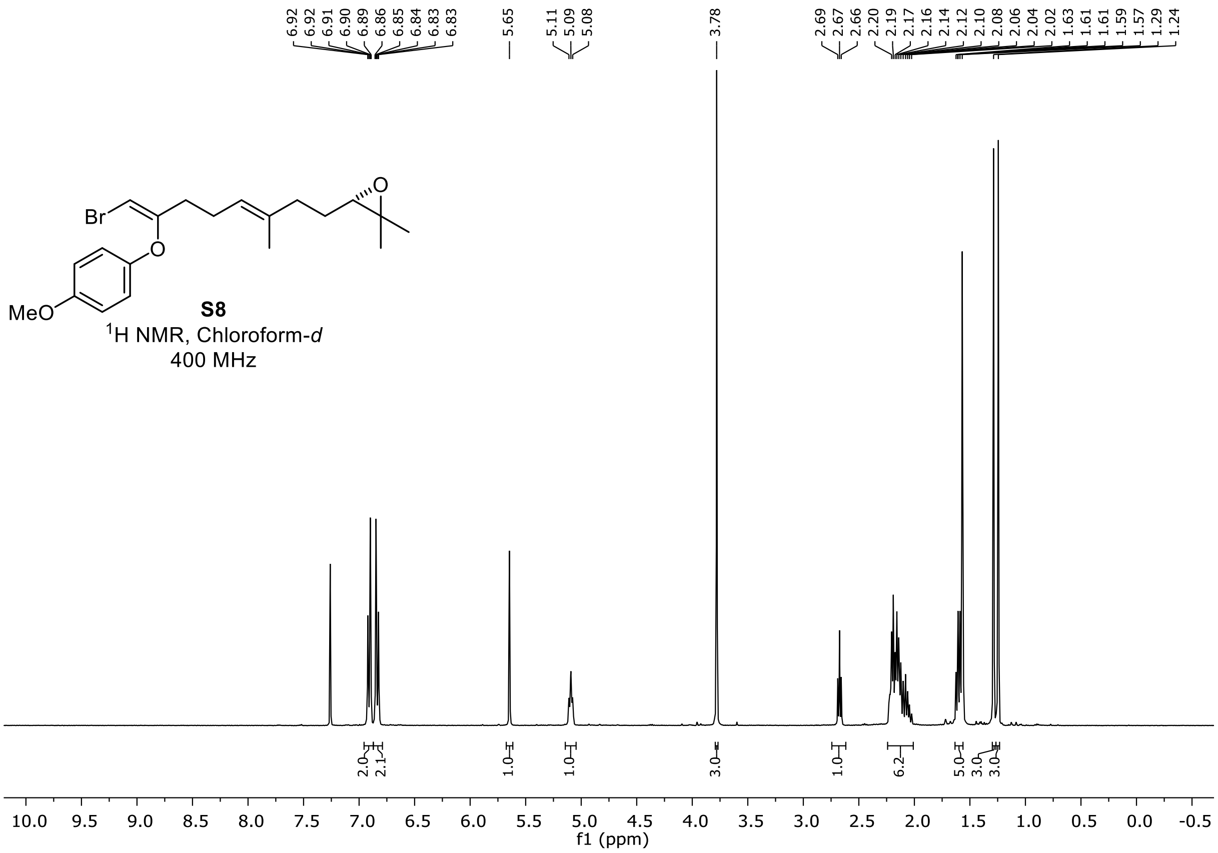
Br
O
O
MeO
S8
^{1}H NMR, Chloroform-*d*
400 MHz
f1 (ppm)

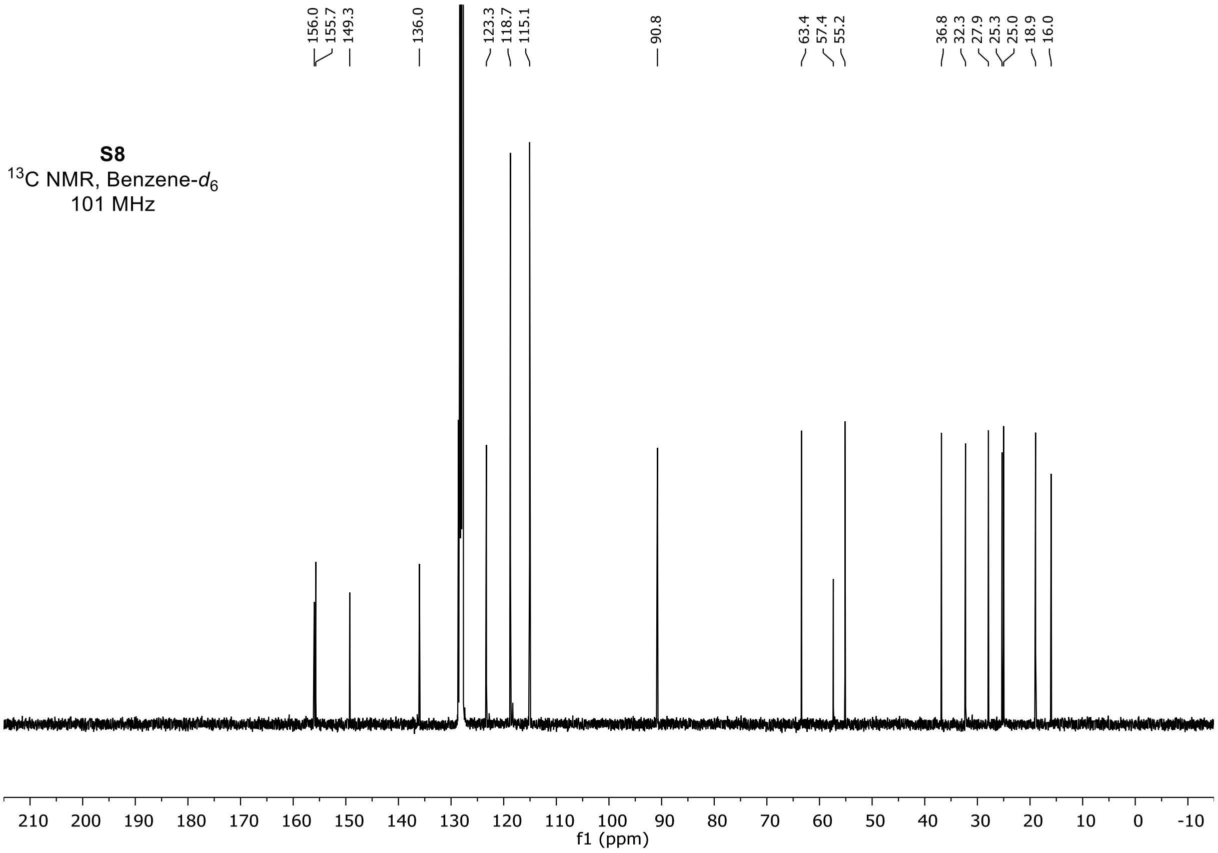
S8
^{13}C NMR, Benzene-*d*$_{6}$
101 MHz
f1 (ppm)

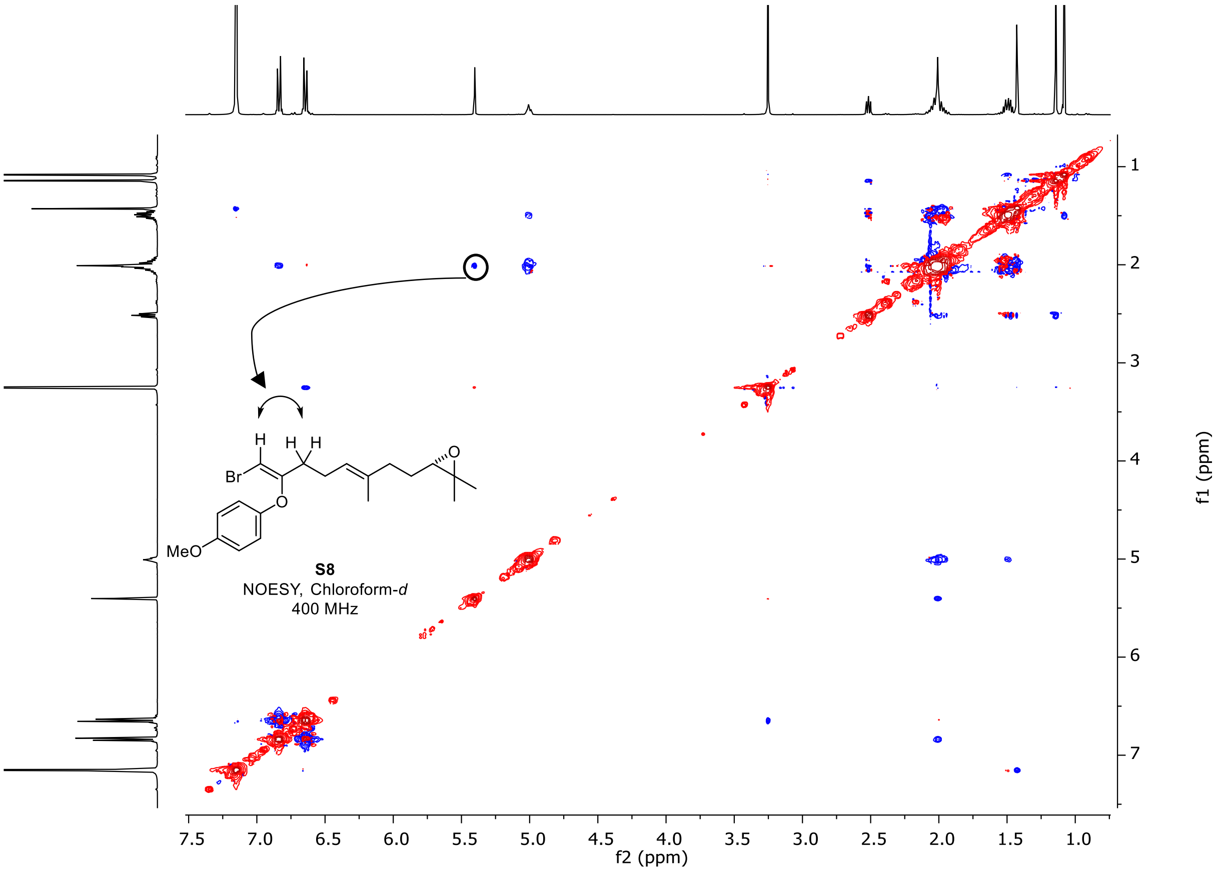
Br
H
H
H
O
O
MeO
S8
NOESY, Chloroform-d
400 MHz
f2 (ppm)
f1 (ppm)
7.5
7.0
6.5
6.0
5.5
5.0
4.5
4.0
3.5
3.0
2.5
2.0
1.5
1.0
1
2
3
4
5
6
7

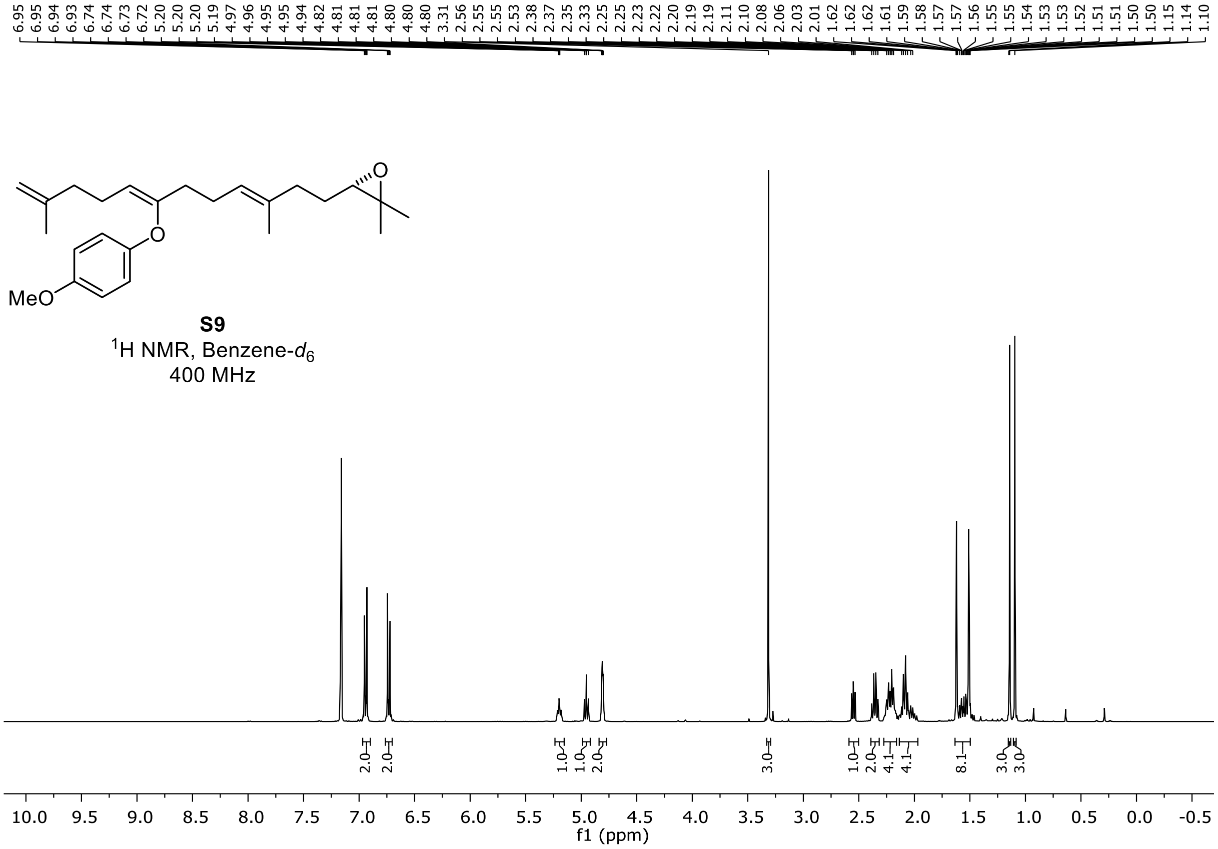

S9
^{1}H NMR, Benzene-d_6
400 MHz

S9
^{13}C NMR, Benzene-d_6
101 MHz

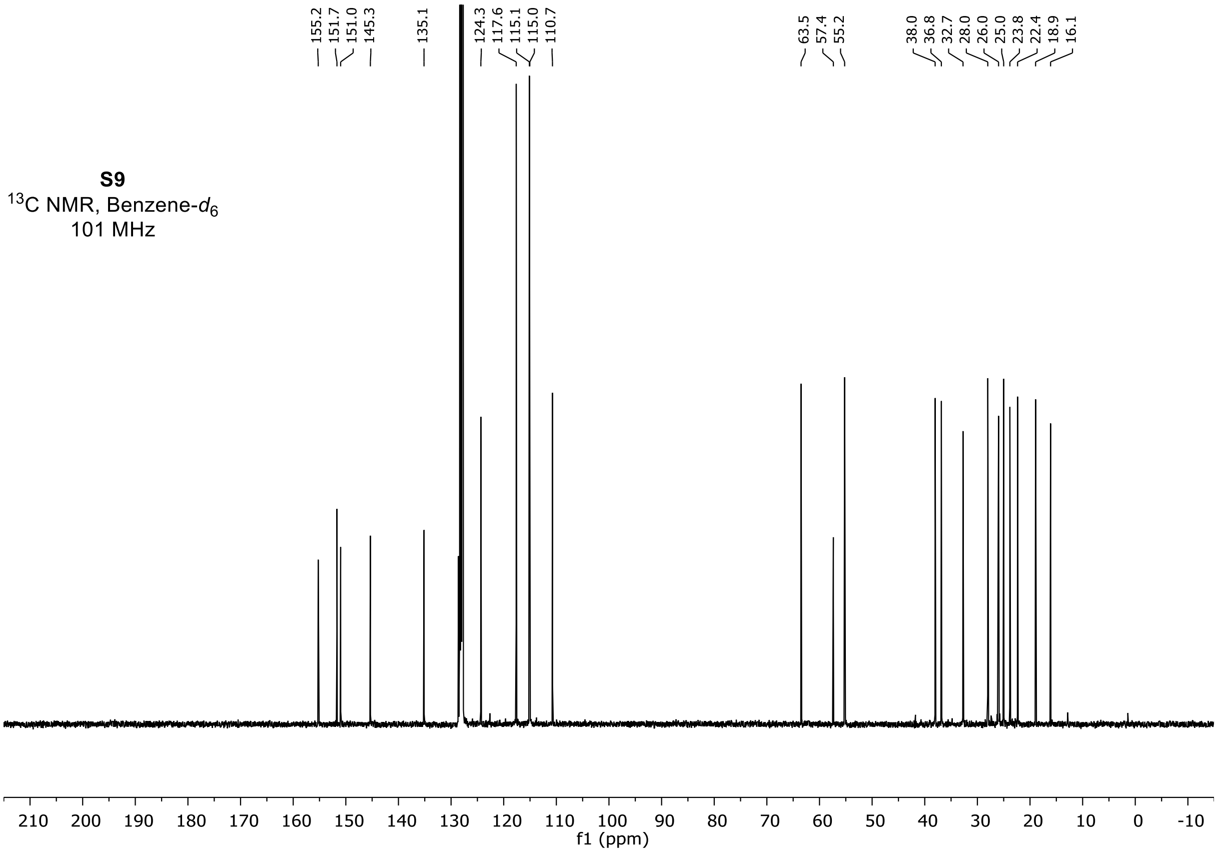

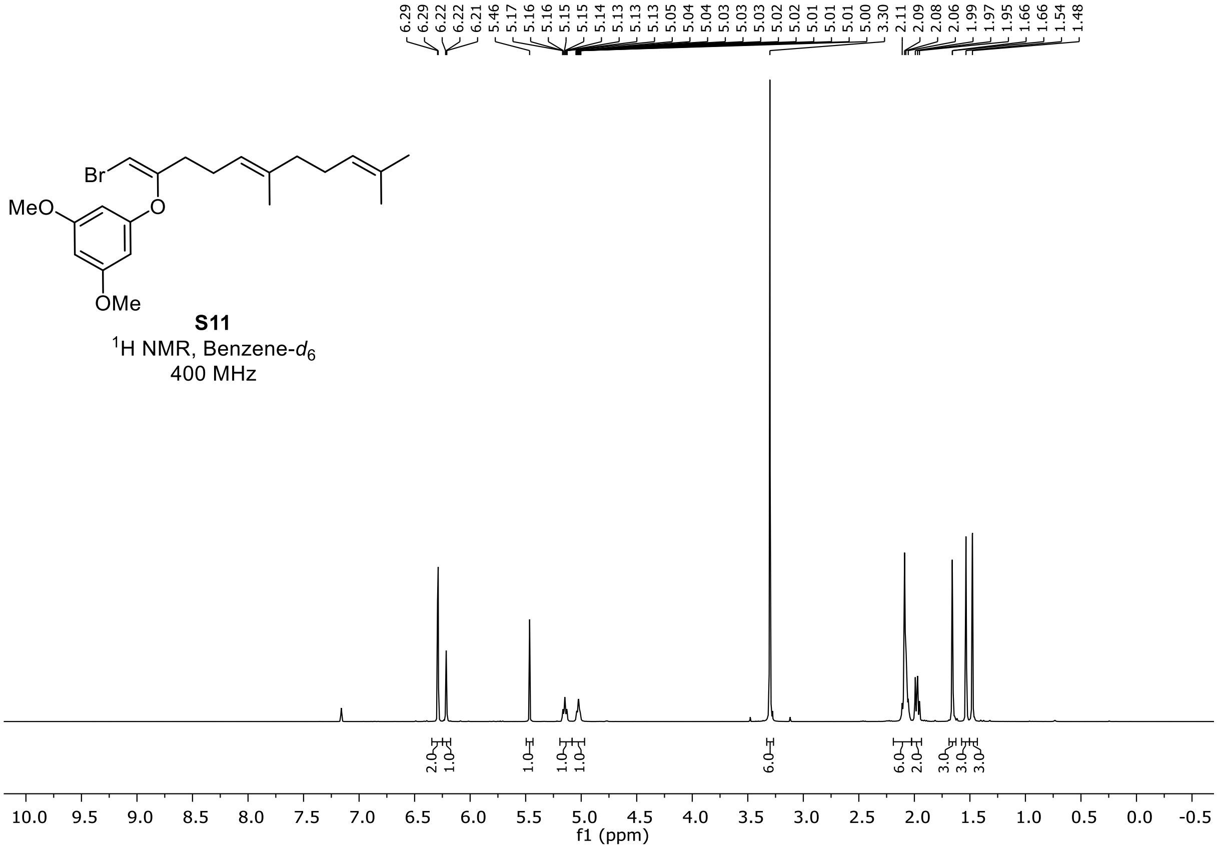
Br
MeO
O
OMe
S11
¹H NMR, Benzene-d6
400 MHz
6.29 6.29 6.22 6.22 6.21 5.46 5.17 5.16 5.16 5.15 5.15 5.14 5.13 5.13 5.13 5.05 5.04 5.04 5.03 5.03 5.03 5.02 5.01 5.01 5.01 5.00 3.30 2.11 2.09 2.08 2.06 1.99 1.97 1.95 1.66 1.54 1.48
2.0 1.0 1.0 1.0 1.0 6.0 6.0 2.0 3.0 3.0 3.0
f1 (ppm)

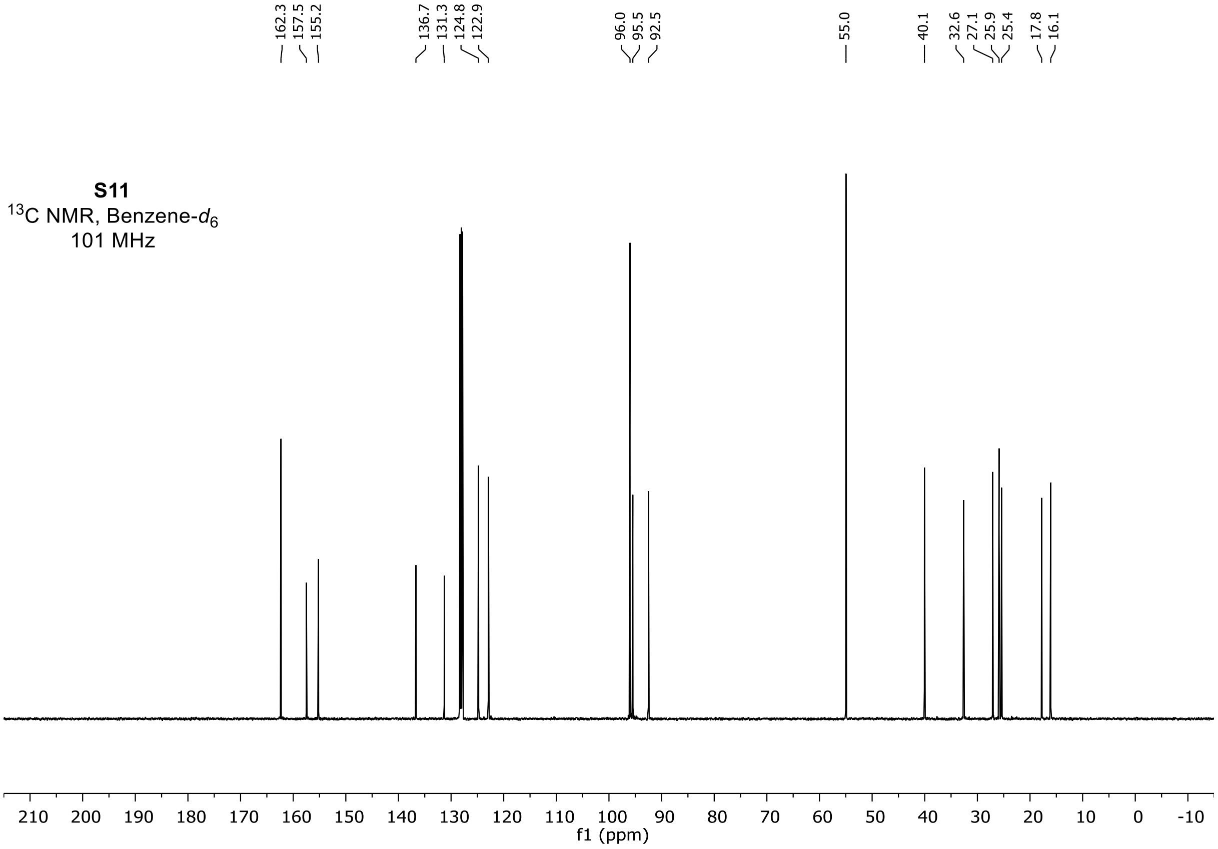
S11
¹³C NMR, Benzene-d6
101 MHz
162.3 157.5 155.2 136.7 131.3 124.8 122.9 96.0 95.5 92.5 55.0 40.1 32.6 27.1 25.9 25.4 17.8 16.1
f1 (ppm)

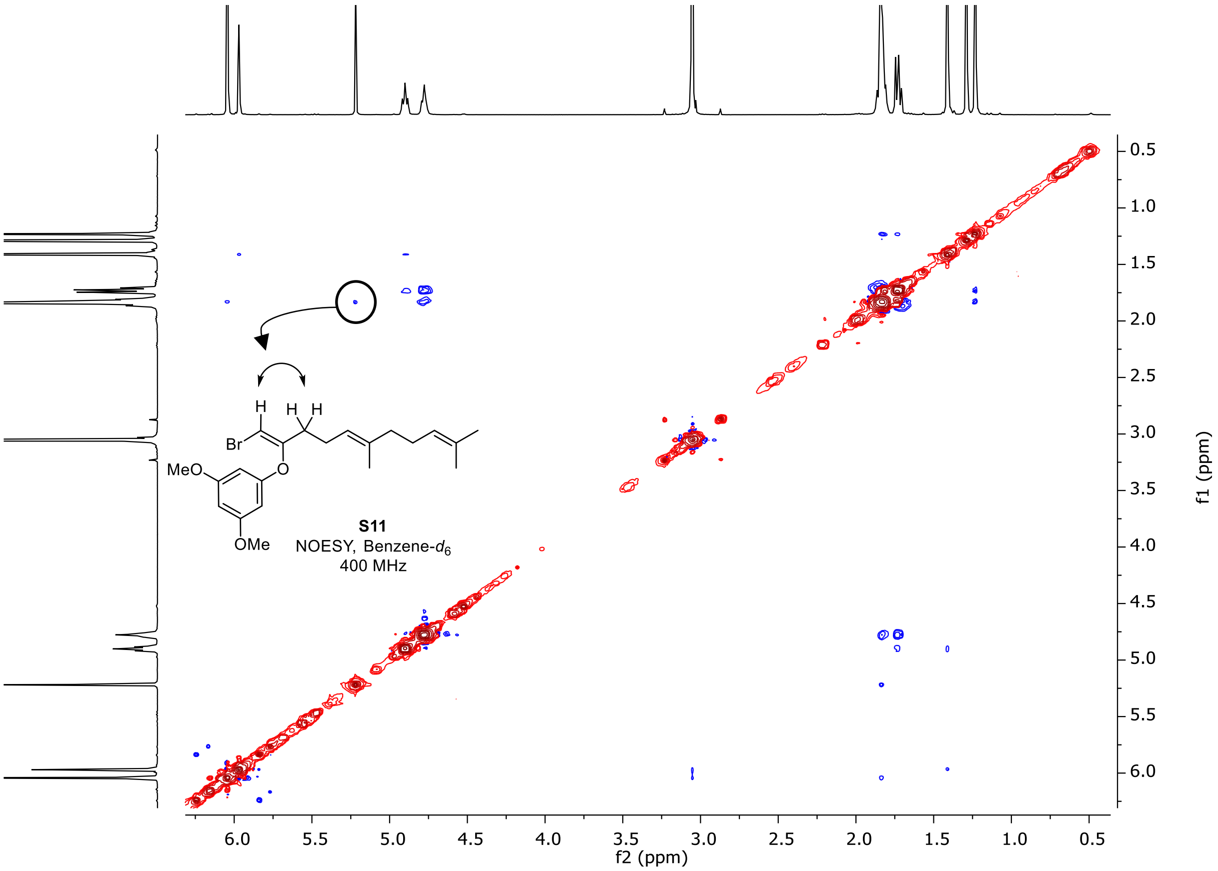
H
H H
Br
O
MeO
OMe
S11
NOESY, Benzene-d_6
400 MHz
f2 (ppm)
f1 (ppm)

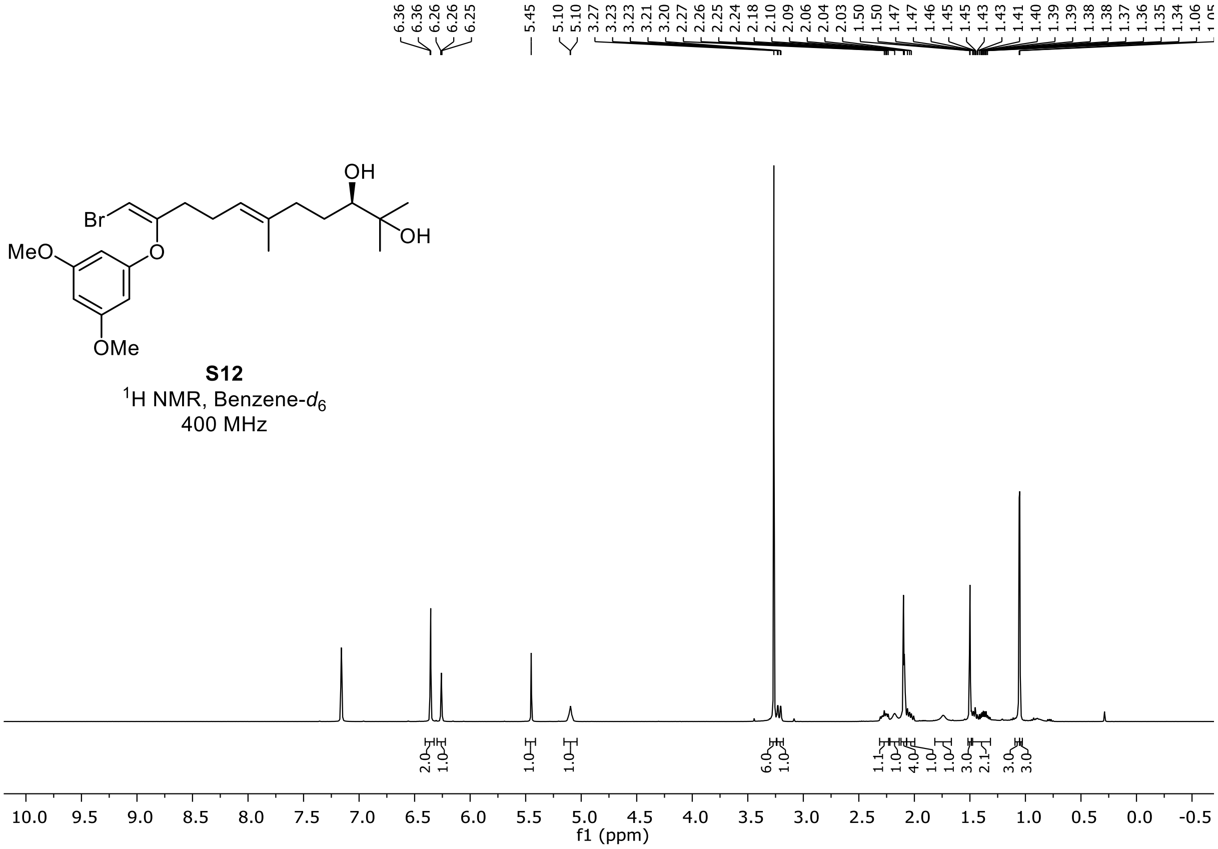
S12
^{1}H NMR, Benzene-d_6
400 MHz
f1 (ppm)

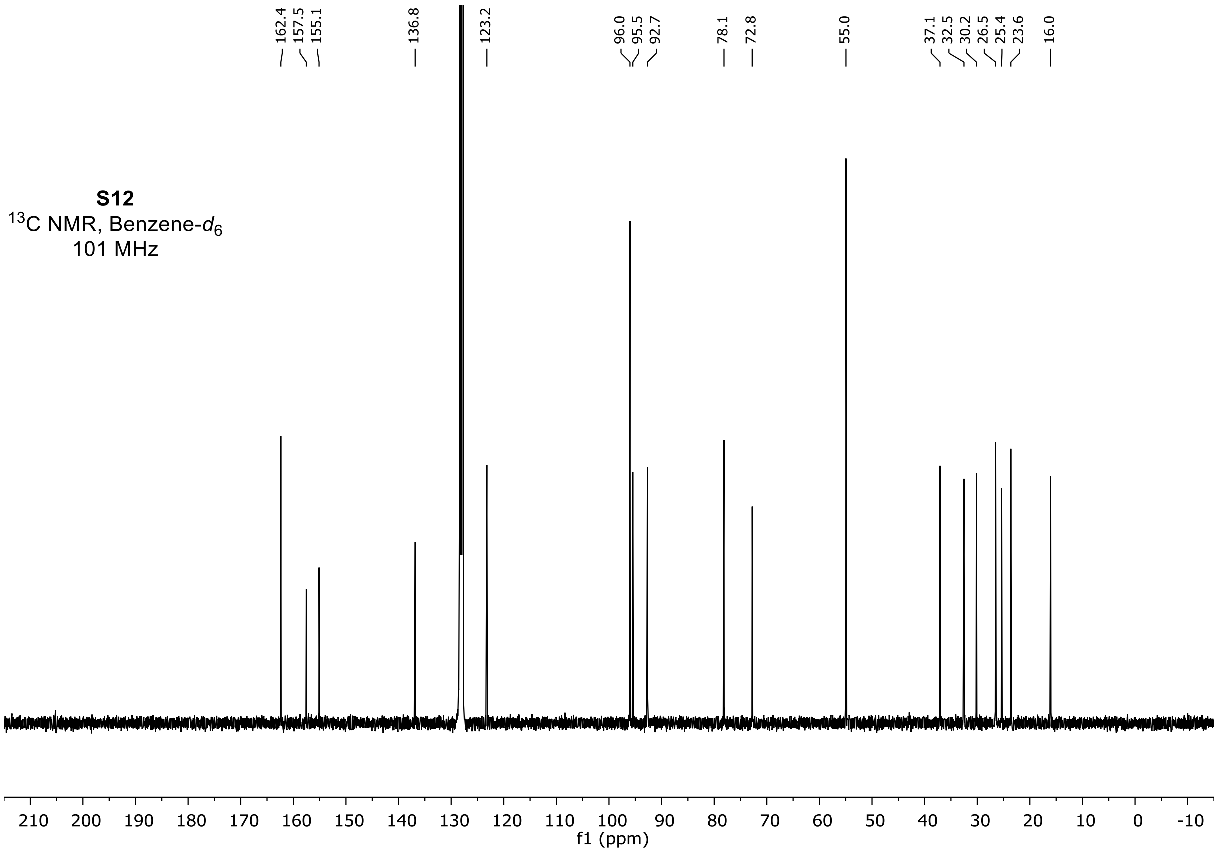
S12
^{13}C NMR, Benzene-d_6
101 MHz
162.4
157.5
155.1
136.8
123.2
96.0
95.5
92.7
78.1
72.8
55.0
37.1
32.5
30.2
26.5
25.4
23.6
16.0
f1 (ppm)

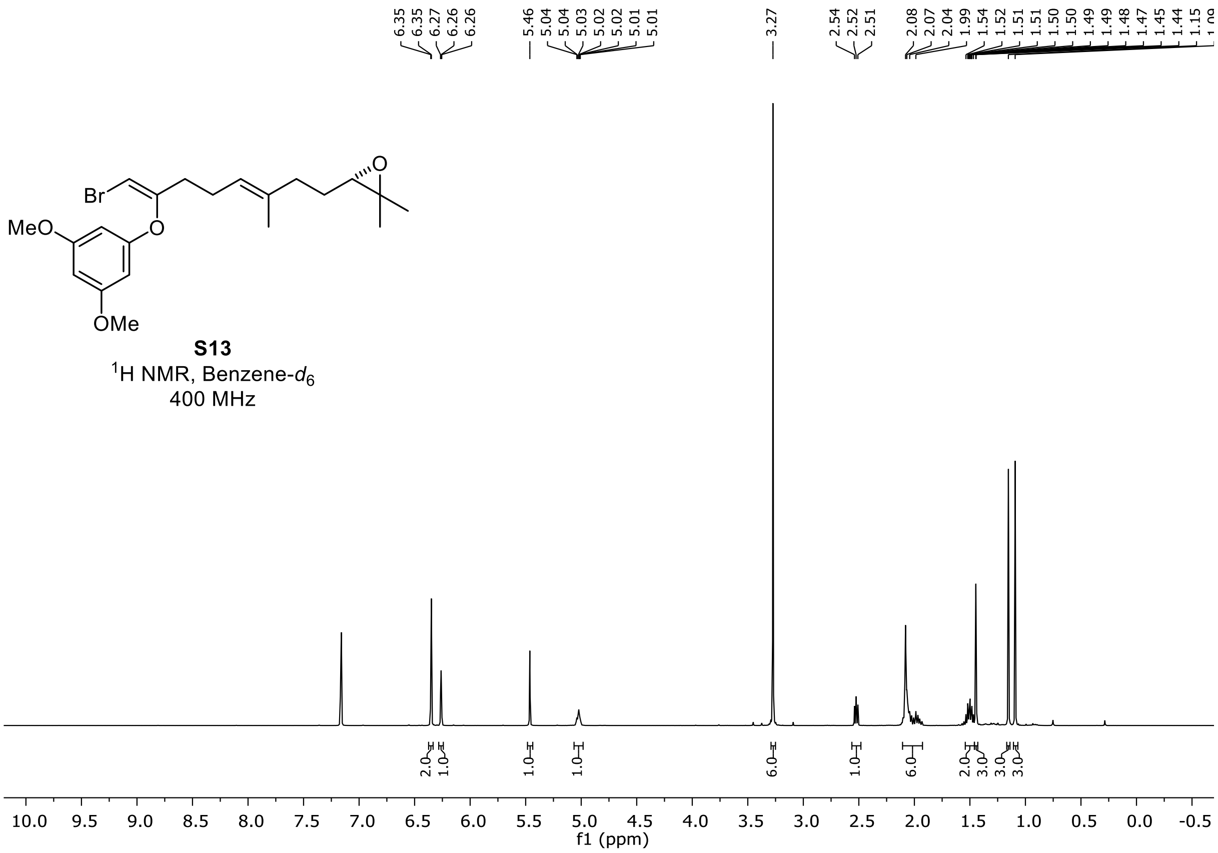

S13
^{1}H NMR, Benzene-d_6
400 MHz

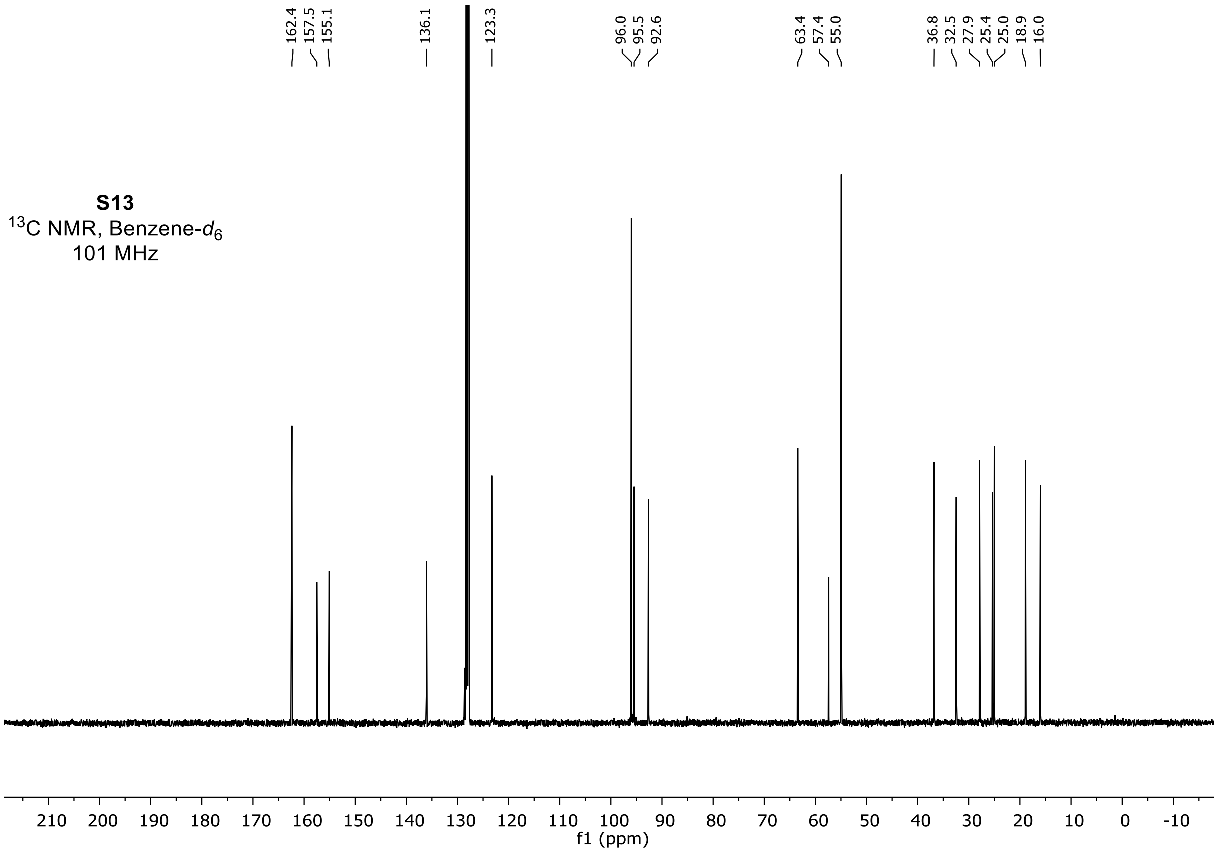

S13
^{13}C NMR, Benzene-d_6
101 MHz

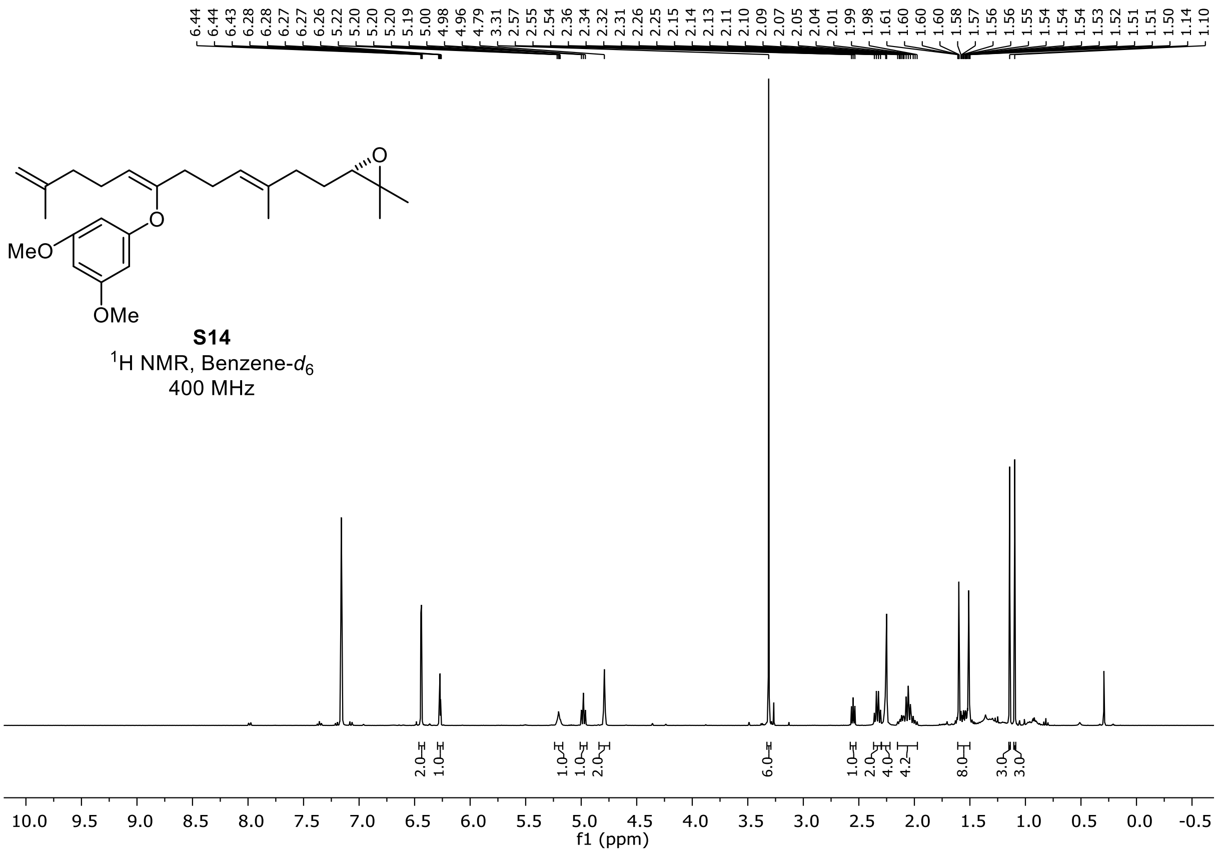
S14
^{1}H NMR, Benzene-d_6
400 MHz
MeO
OMe
O
f1 (ppm)

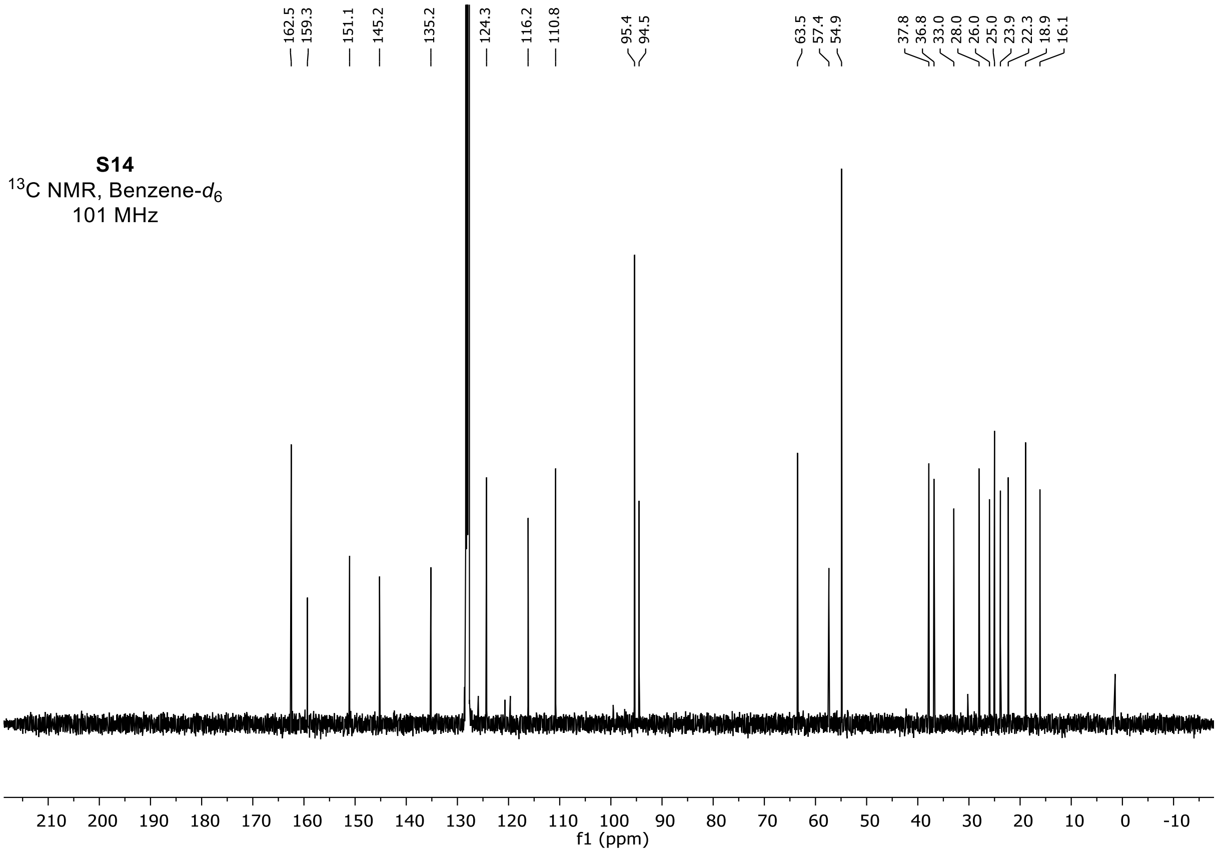
S14
^{13}C NMR, Benzene-d_6
101 MHz
f1 (ppm)

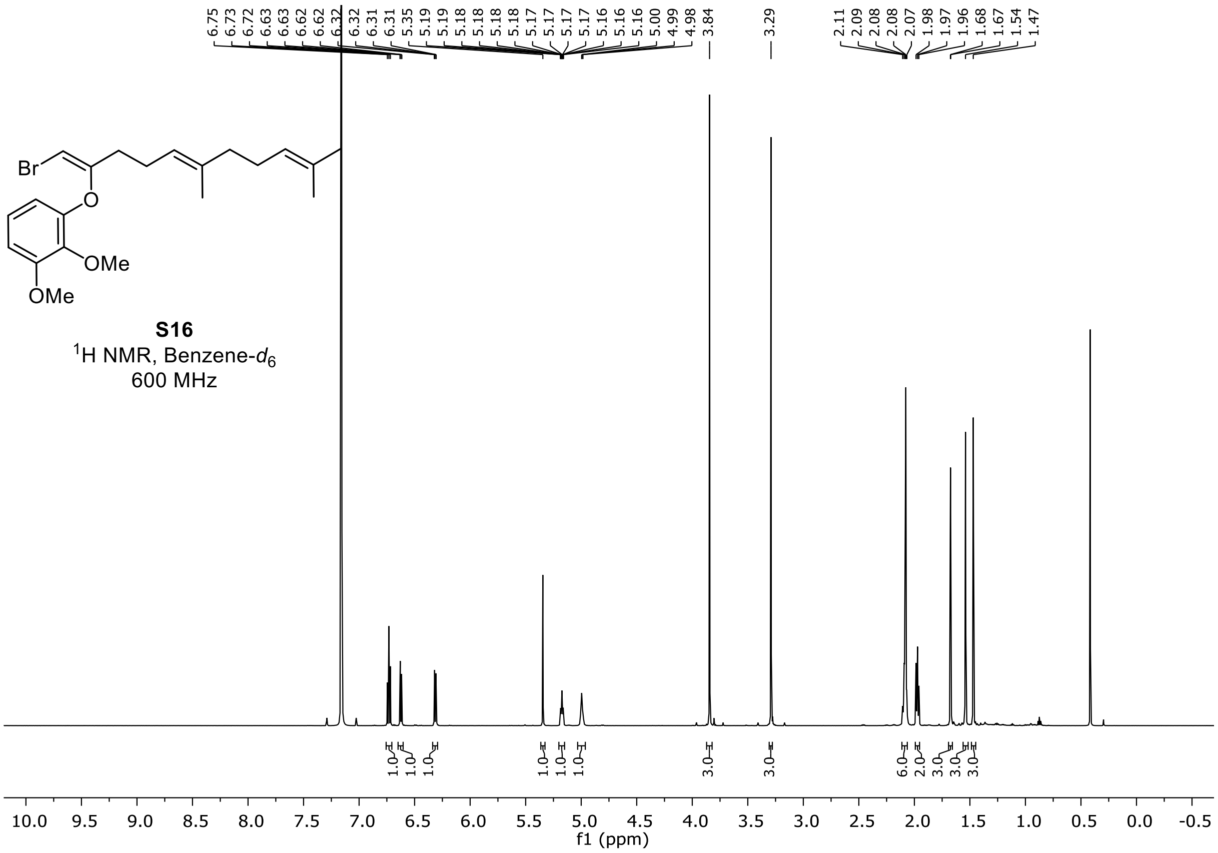
S16
^{1}H NMR, Benzene-d_6
600 MHz
Br
O
OMe
OMe
f1 (ppm)

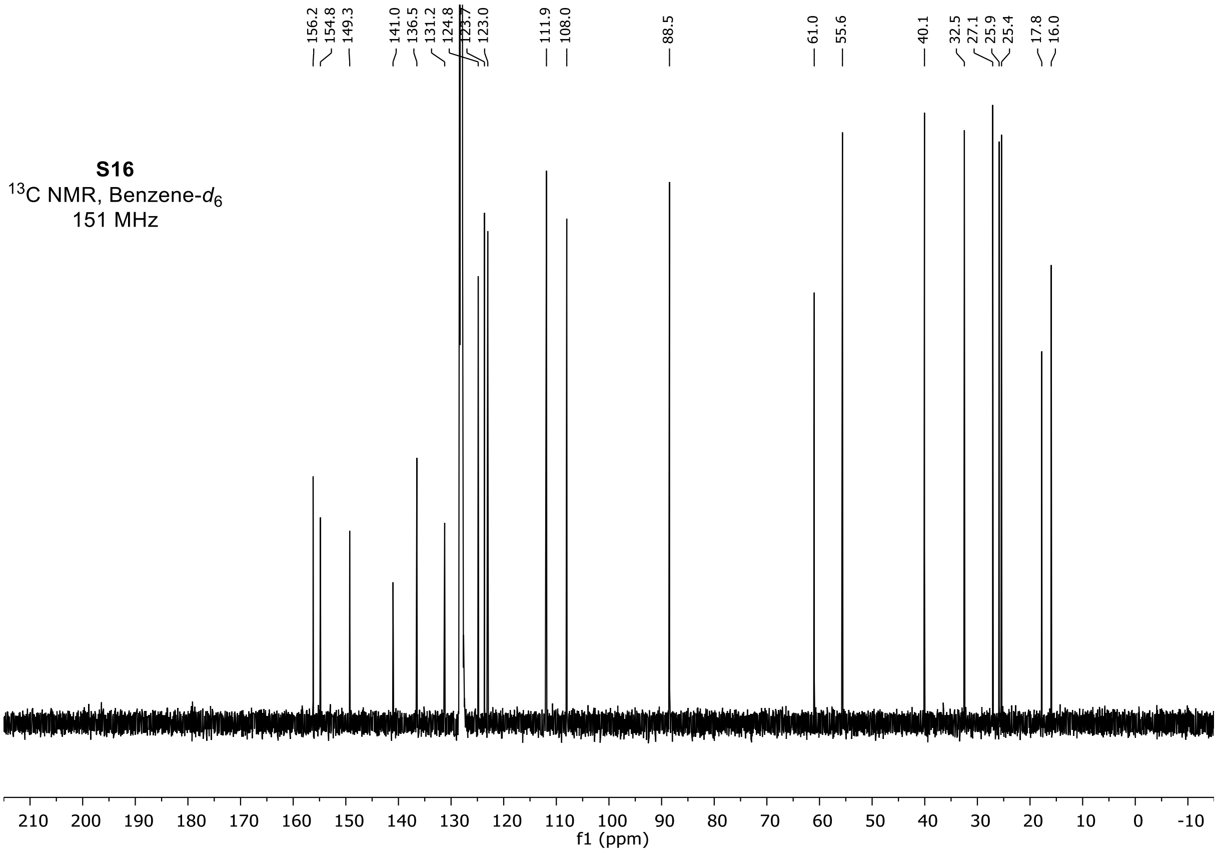
S16
^{13}C NMR, Benzene-d_6
151 MHz
f1 (ppm)

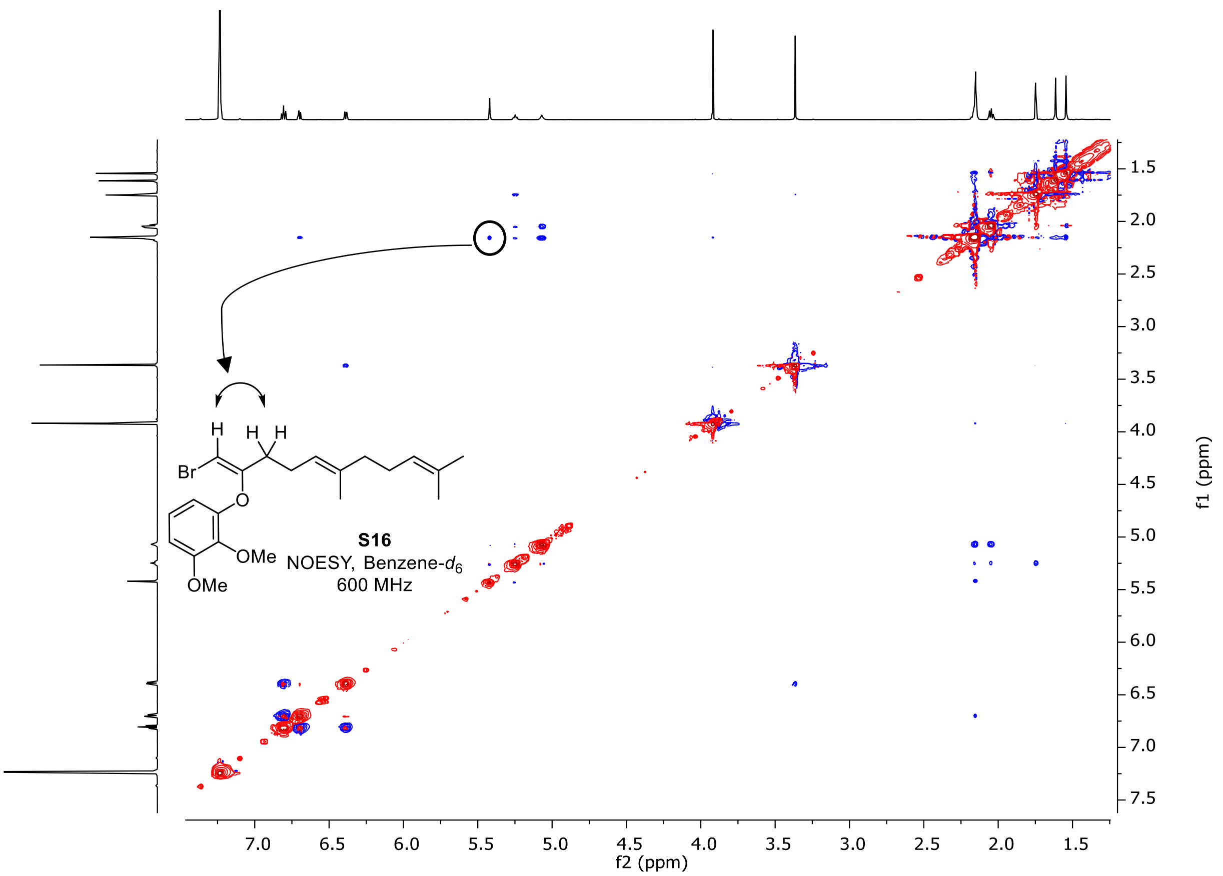
H
H H
Br
O
OMe
OMe
S16
NOESY, Benzene-d_6
600 MHz
f2 (ppm)
f1 (ppm)
7.0
6.5
6.0
5.5
5.0
4.5
4.0
3.5
3.0
2.5
2.0
1.5
7.5

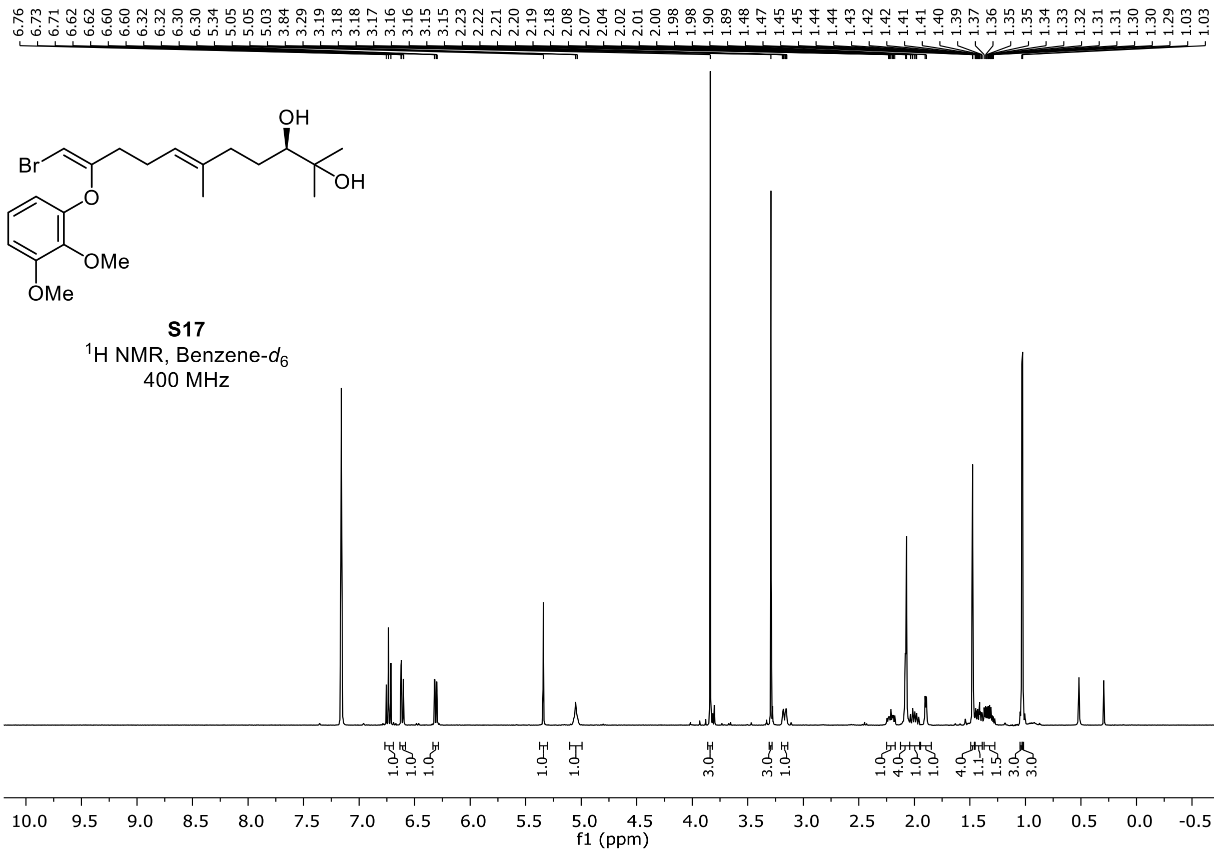
6.76 6.73 6.71 6.62 6.62 6.60 6.60 6.32 6.32 6.30 6.30 5.34 5.05 5.05 5.03 3.84 3.29 3.19 3.18 3.18 3.17 3.16 3.16 3.15 3.15 2.23 2.22 2.21 2.20 2.19 2.18 2.08 2.07 2.04 2.02 2.01 2.00 1.98 1.90 1.89 1.48 1.47 1.45 1.45 1.44 1.44 1.43 1.42 1.42 1.41 1.41 1.40 1.39 1.37 1.36 1.35 1.35 1.34 1.33 1.32 1.31 1.31 1.30 1.30 1.29 1.03 1.03
Br
O
OH
OH
OMe
OMe
S17
1H NMR, Benzene-d6
400 MHz
1.0 1.0 1.0 1.0 1.0 3.0 3.0 1.0 1.0 4.0 1.0 1.0 4.0 1.1 1.5 3.0 3.0
10.0 9.5 9.0 8.5 8.0 7.5 7.0 6.5 6.0 5.5 5.0 4.5 4.0 3.5 3.0 2.5 2.0 1.5 1.0 0.5 0.0 -0.5
f1 (ppm)

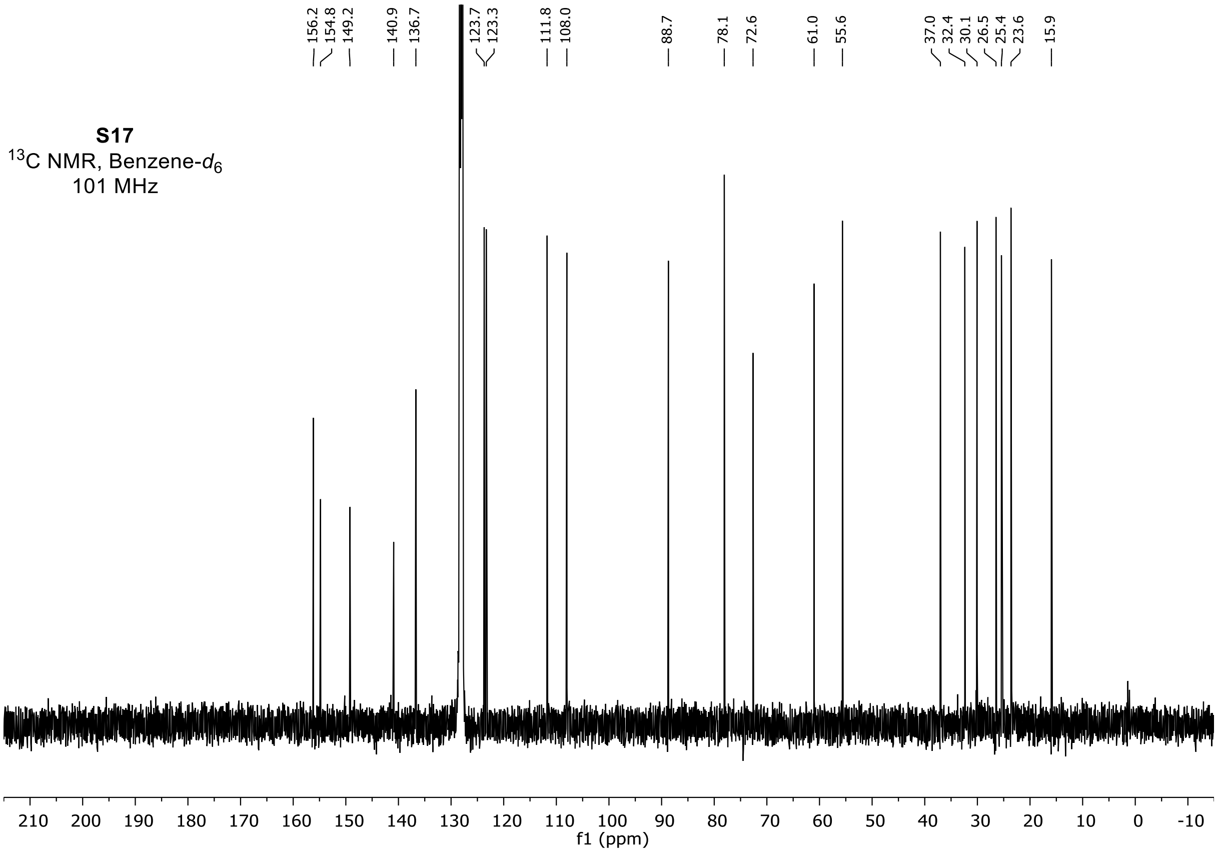
156.2 154.8 149.2 140.9 136.7 123.7 123.3 111.8 108.0 88.7 78.1 72.6 61.0 55.6 37.0 32.4 30.1 26.5 25.4 23.6 15.9
S17
13C NMR, Benzene-d6
101 MHz
210 200 190 180 170 160 150 140 130 120 110 100 90 80 70 60 50 40 30 20 10 0 -10
f1 (ppm)

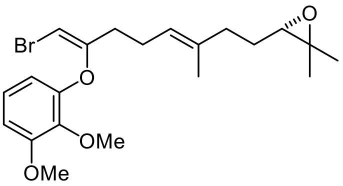

S18
^{1}H NMR, Benzene-d_6
400 MHz

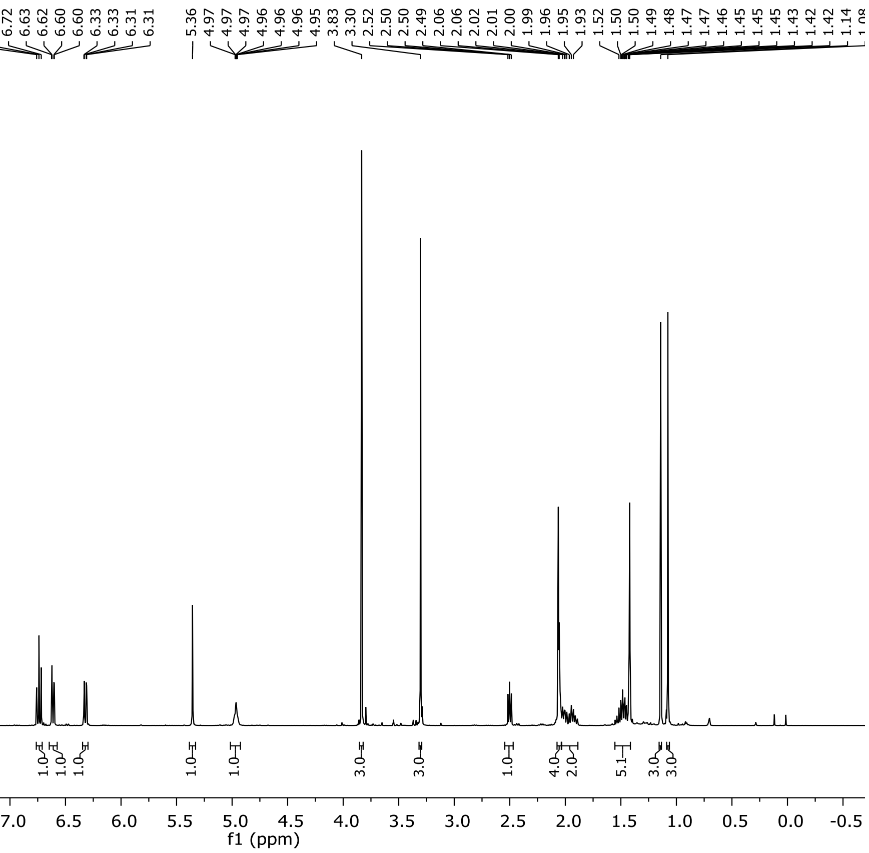

S18
^{13}C NMR, Benzene-d_6
101 MHz

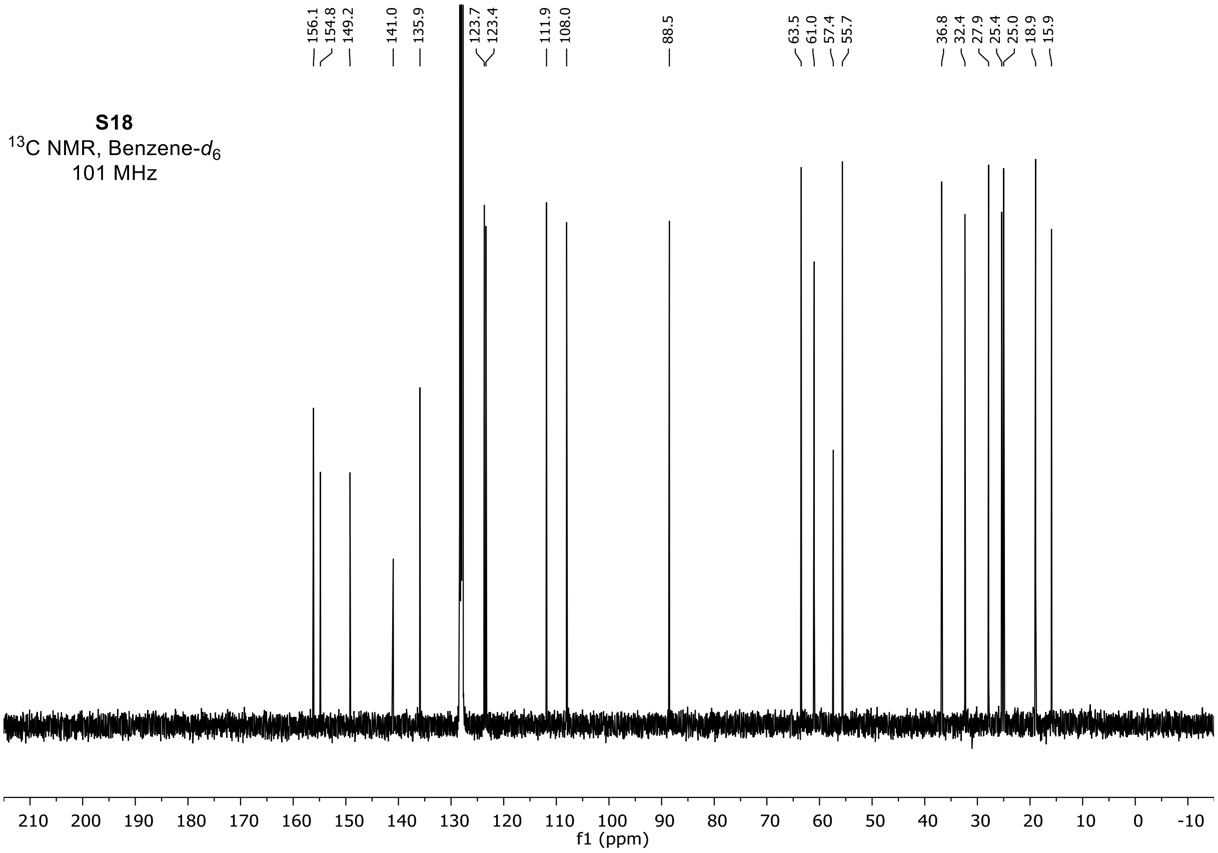

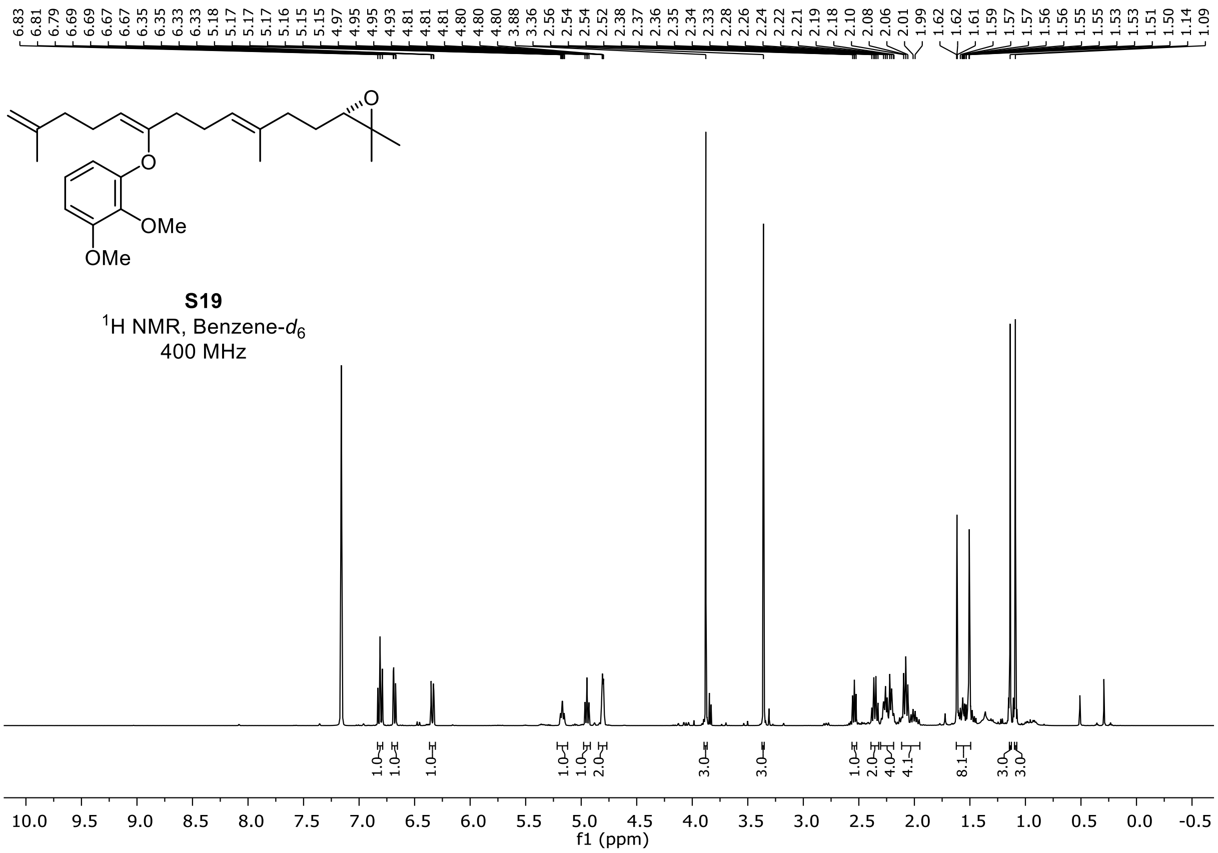
6.83 6.81 6.79 6.69 6.69 6.67 6.67 6.35 6.35 6.33 6.33 5.18 5.17 5.17 5.17 5.16 5.15 5.15 4.97 4.95 4.95 4.93 4.81 4.81 4.81 4.80 4.80 4.80 3.88 3.36 2.56 2.54 2.54 2.52 2.38 2.37 2.36 2.35 2.34 2.33 2.28 2.26 2.24 2.22 2.21 2.19 2.18 2.10 2.08 2.06 2.01 1.99 1.62 1.62 1.61 1.59 1.57 1.57 1.56 1.55 1.55 1.53 1.53 1.51 1.50 1.14 1.09
O
OMe
OMe
O
S19
^{1}H NMR, Benzene-d_6
400 MHz
1.0 1.0 1.0 1.0 1.0 2.0 3.0 3.0 1.0 2.0 4.0 4.1 8.1 3.0 3.0
10.0 9.5 9.0 8.5 8.0 7.5 7.0 6.5 6.0 5.5 5.0 4.5 4.0 3.5 3.0 2.5 2.0 1.5 1.0 0.5 0.0 -0.5
f1 (ppm)

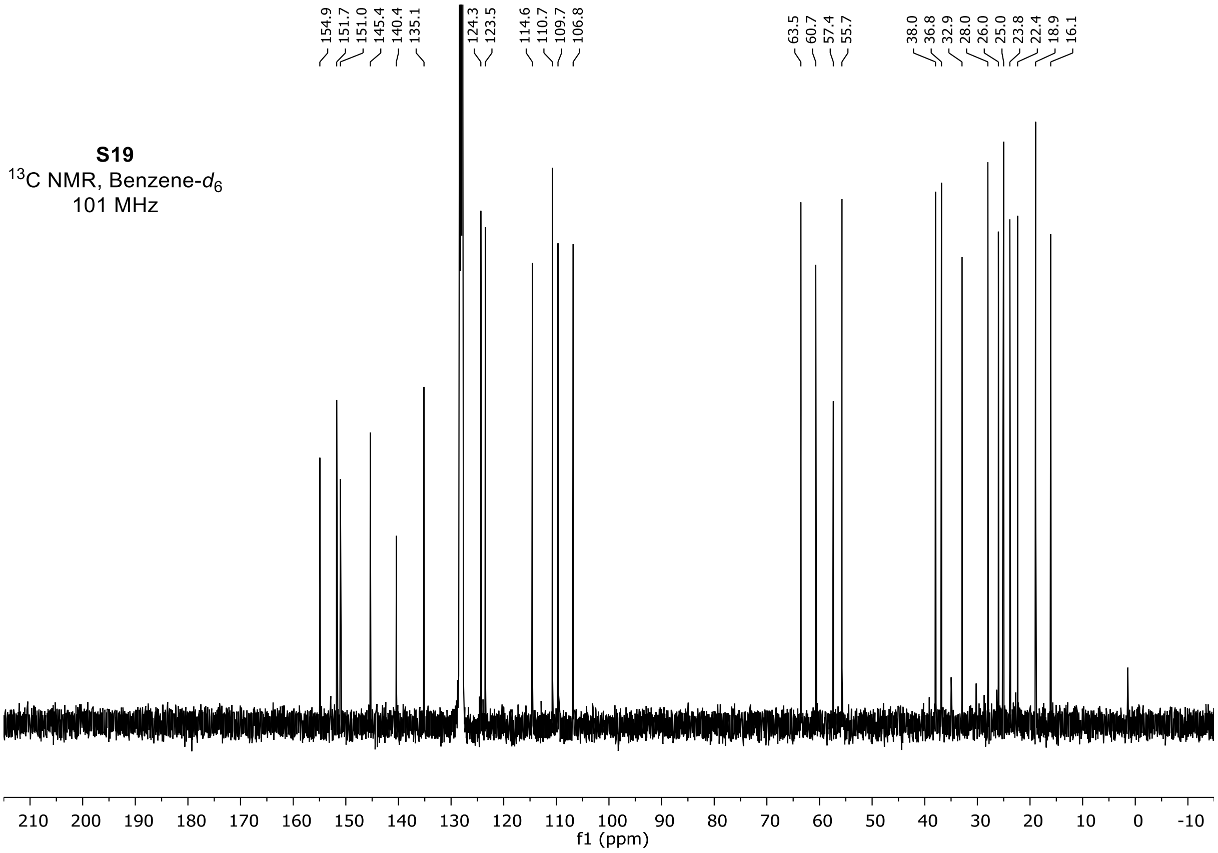
154.9 151.7 151.0 145.4 140.4 135.1 124.3 123.5 114.6 110.7 109.7 106.8 63.5 60.7 57.4 55.7 38.0 36.8 32.9 28.0 26.0 25.0 23.8 22.4 18.9 16.1
S19
^{13}C NMR, Benzene-d_6
101 MHz
210 200 190 180 170 160 150 140 130 120 110 100 90 80 70 60 50 40 30 20 10 0 -10
f1 (ppm)

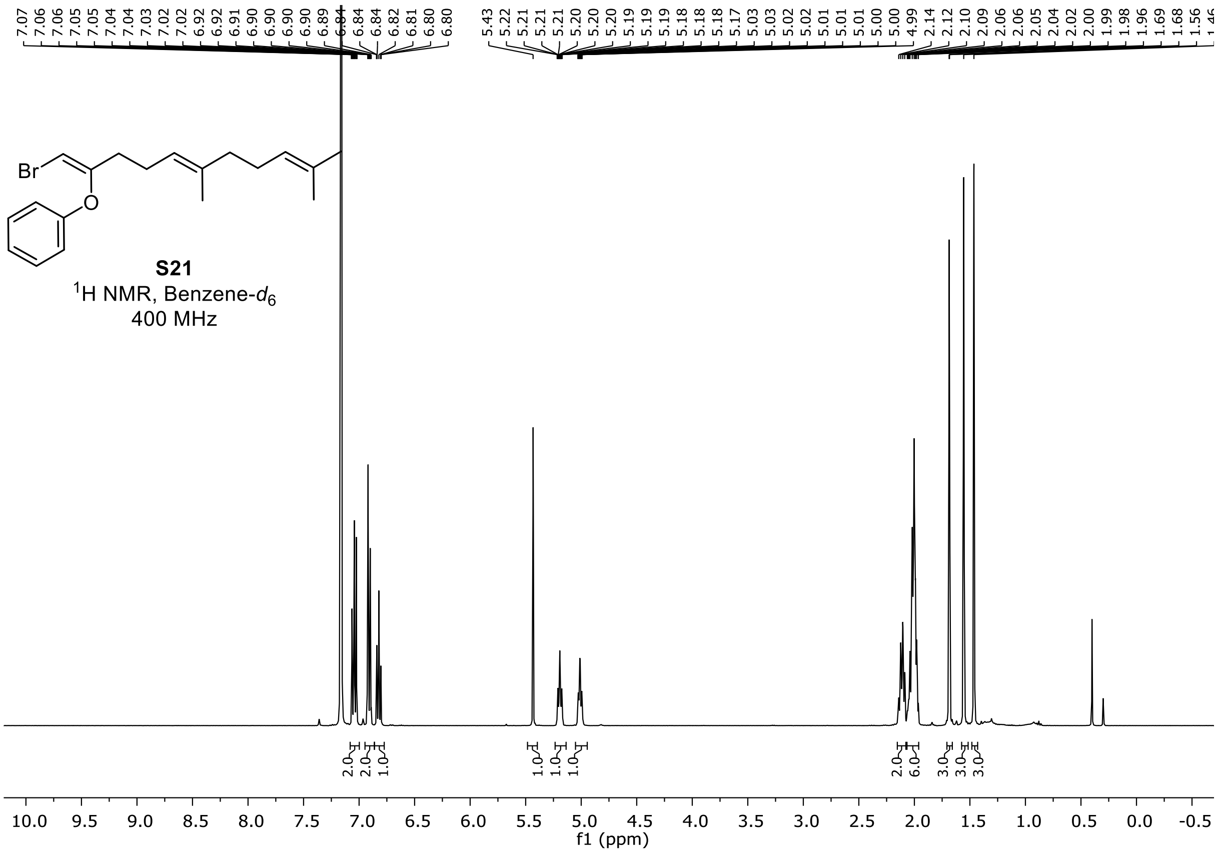
Br
O
S21
1H NMR, Benzene-d6
400 MHz
f1 (ppm)

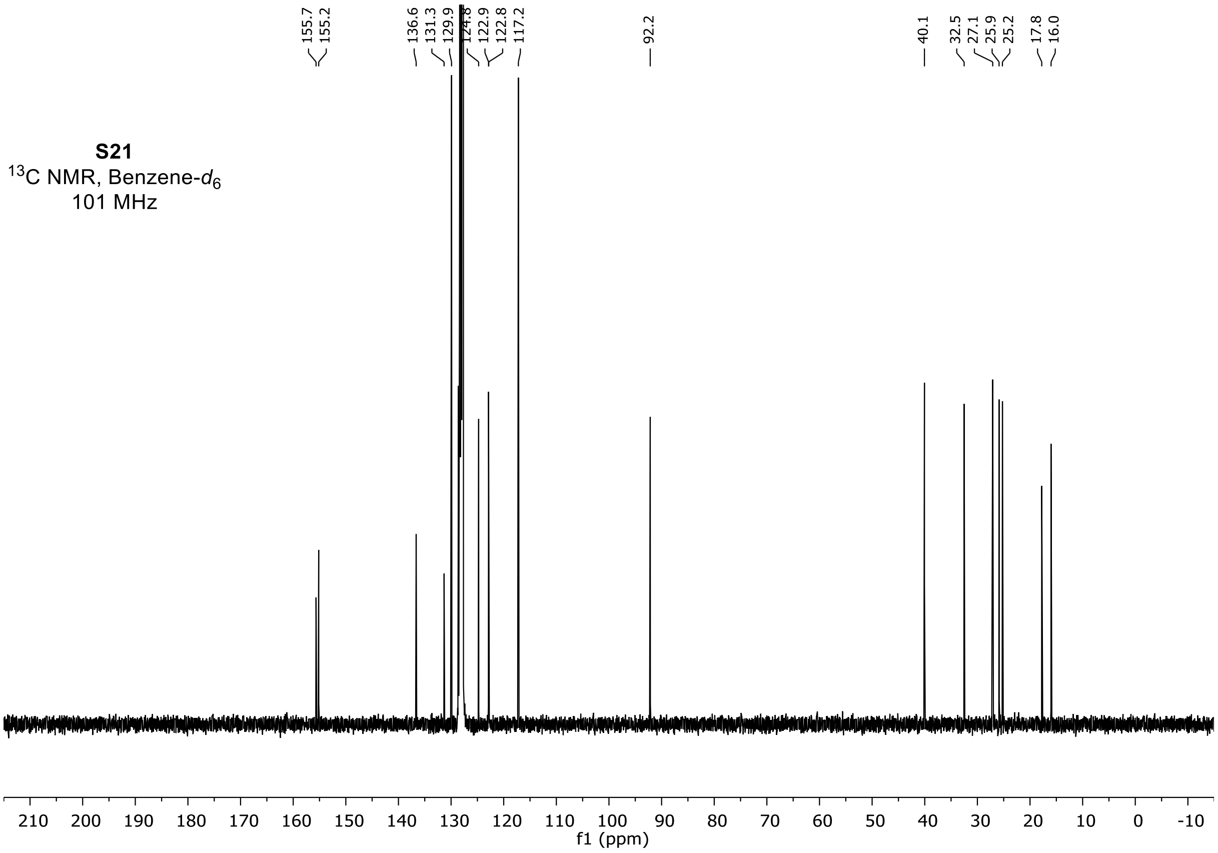
S21
13C NMR, Benzene-d6
101 MHz
155.7
155.2
136.6
131.3
129.9
124.8
122.9
122.8
117.2
92.2
40.1
32.5
27.1
25.9
25.2
17.8
16.0
f1 (ppm)

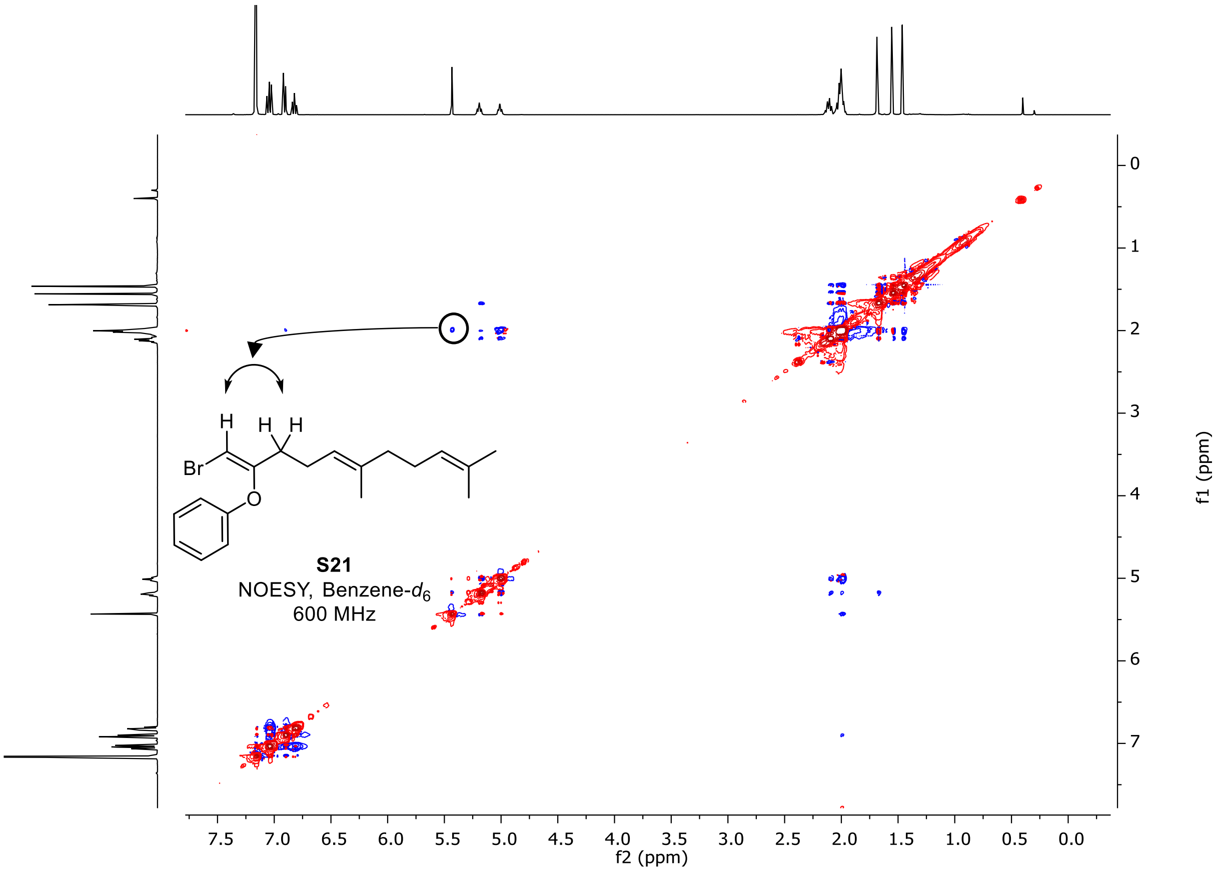
Br
O
H
H H
S21
NOESY, Benzene-d_6
600 MHz
f2 (ppm)
f1 (ppm)

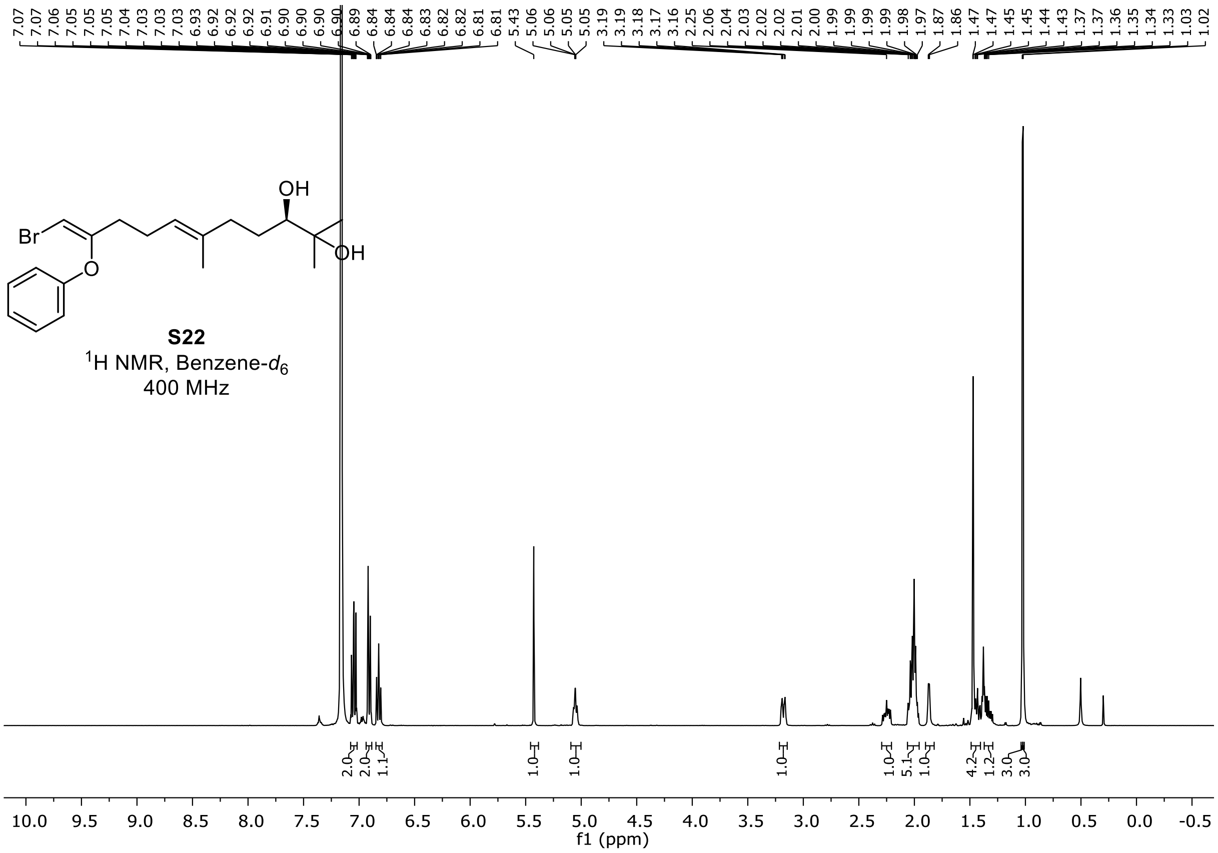
7.07 7.07 7.06 7.05 7.05 7.05 7.04 7.03 7.03 7.03 6.93 6.92 6.92 6.92 6.91 6.90 6.90 6.90 6.90 6.89 6.84 6.84 6.84 6.83 6.82 6.82 6.81 6.81 5.43 5.06 5.06 5.05 5.05 3.19 3.19 3.18 3.17 3.16 2.25 2.06 2.04 2.03 2.02 2.02 2.01 2.00 1.99 1.99 1.99 1.99 1.98 1.97 1.87 1.86 1.47 1.47 1.45 1.45 1.44 1.43 1.37 1.37 1.36 1.35 1.34 1.33 1.03 1.02
OH
Br
O
OH
S22
^{1}H NMR, Benzene-d_6
400 MHz
2.0 2.0 1.1 1.0 1.0 1.0 1.0 5.1 1.0 4.2 1.2 3.0 3.0
10.0 9.5 9.0 8.5 8.0 7.5 7.0 6.5 6.0 5.5 5.0 4.5 4.0 3.5 3.0 2.5 2.0 1.5 1.0 0.5 0.0 -0.5
f1 (ppm)

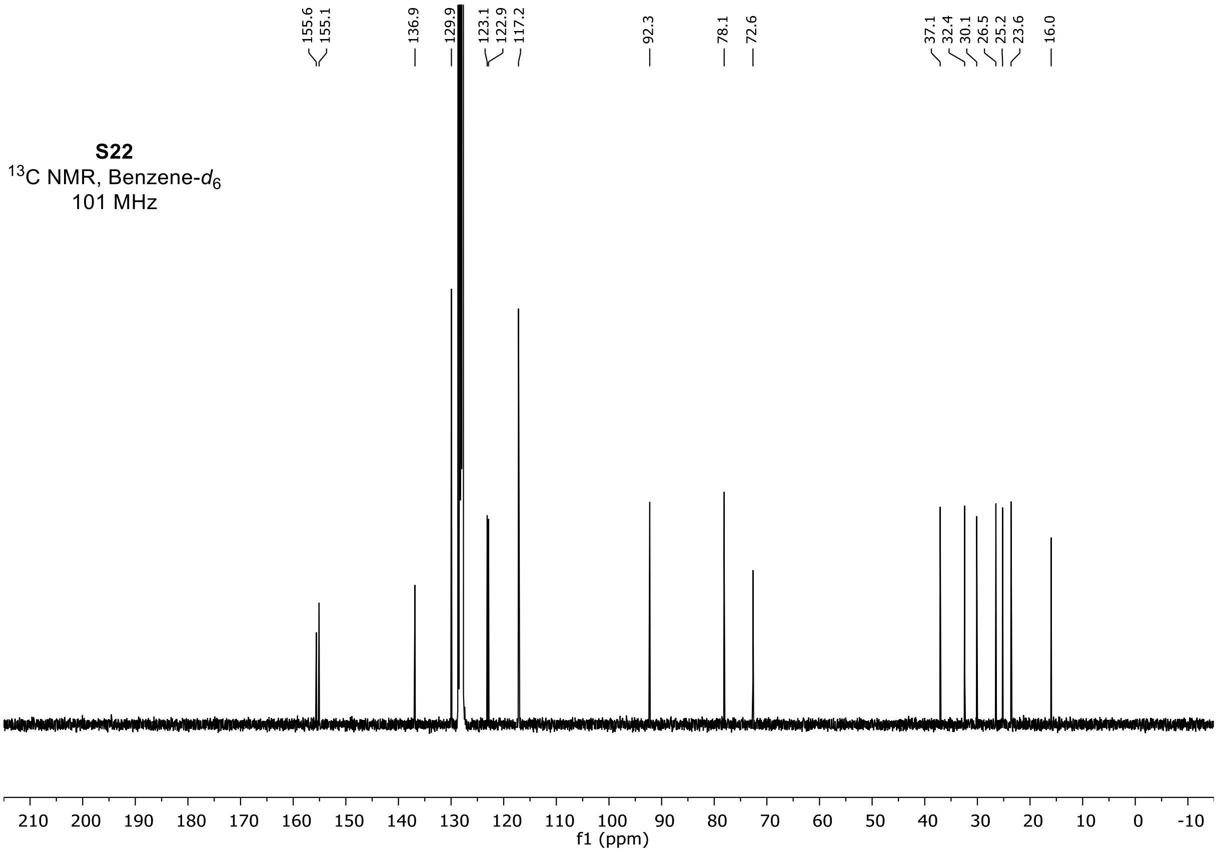
155.6 155.1 136.9 129.9 123.1 122.9 117.2 92.3 78.1 72.6 37.1 32.4 30.1 26.5 25.2 23.6 16.0
S22
^{13}C NMR, Benzene-d_6
101 MHz
210 200 190 180 170 160 150 140 130 120 110 100 90 80 70 60 50 40 30 20 10 0 -10
f1 (ppm)

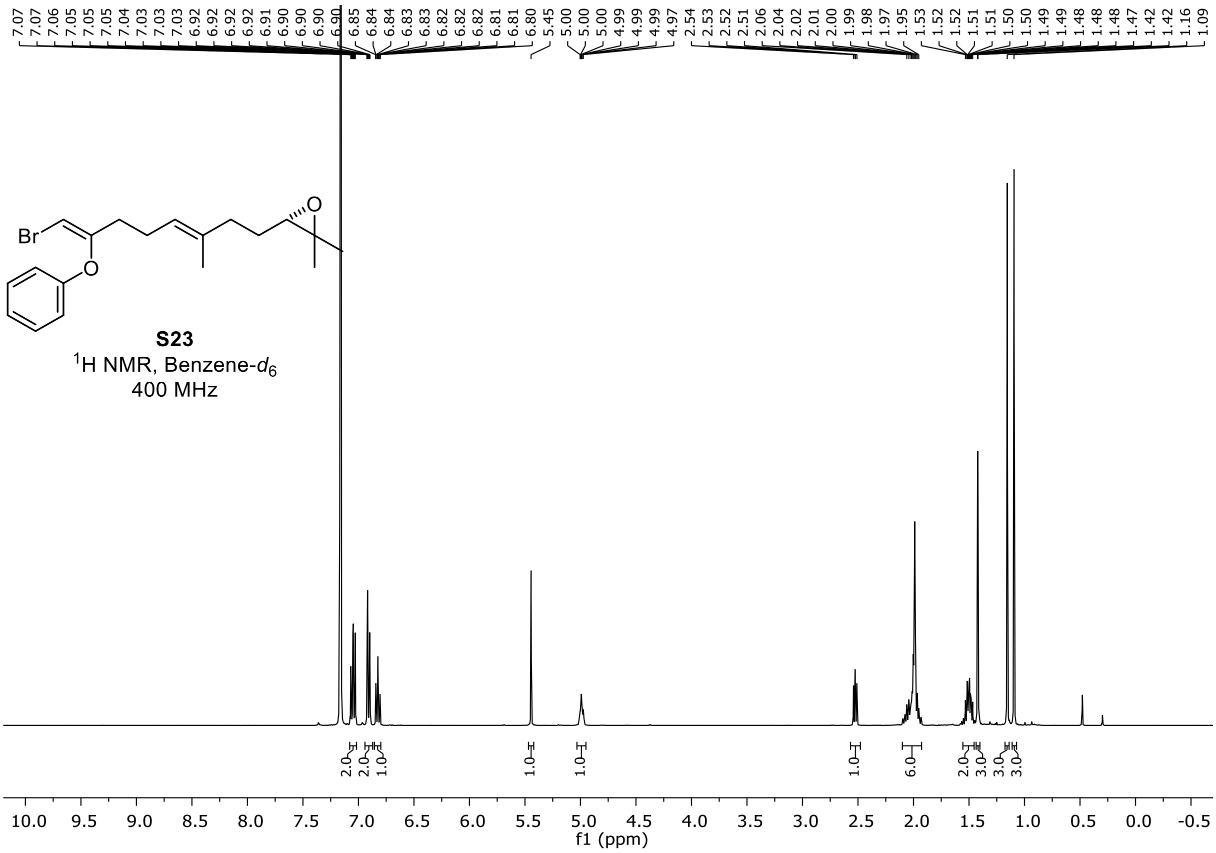
7.07 7.07 7.06 7.05 7.05 7.05 7.04 7.03 7.03 7.03 6.92 6.92 6.92 6.91 6.90 6.90 6.90 6.90 6.85 6.84 6.84 6.83 6.83 6.82 6.82 6.81 6.81 6.80 5.45 5.00 5.00 4.99 4.99 4.99 4.97 2.54 2.53 2.52 2.51 2.06 2.04 2.02 2.01 2.00 1.99 1.98 1.97 1.95 1.53 1.52 1.52 1.51 1.51 1.50 1.50 1.49 1.49 1.48 1.48 1.48 1.47 1.42 1.42 1.16 1.09
Br
O
O
S23
^{1}H NMR, Benzene-d_6
400 MHz
2.0 2.0 1.0 1.0 1.0 1.0 6.0 2.0 3.0 3.0 3.0
10.0 9.5 9.0 8.5 8.0 7.5 7.0 6.5 6.0 5.5 5.0 4.5 4.0 3.5 3.0 2.5 2.0 1.5 1.0 0.5 0.0 -0.5
f1 (ppm)

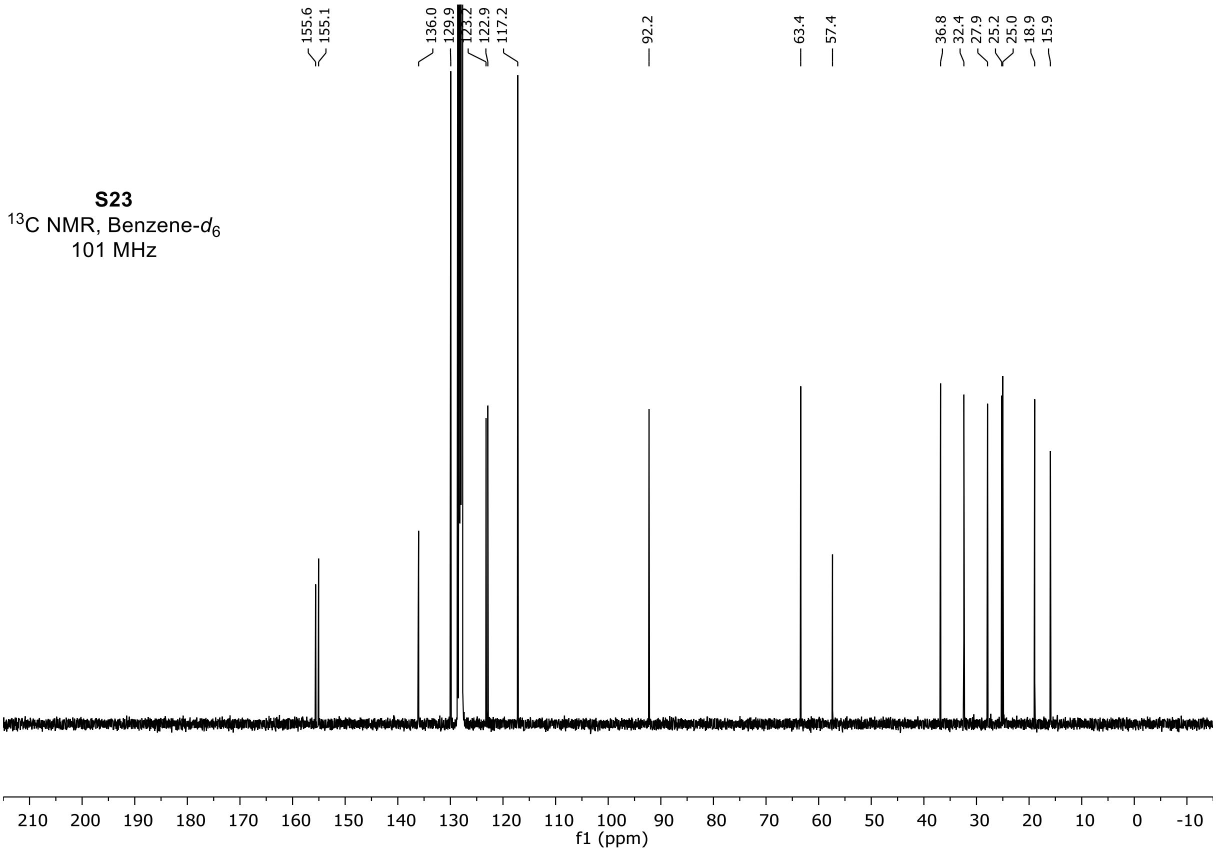
155.6 155.1 136.0 129.9 123.2 122.9 117.2 92.2 63.4 57.4 36.8 32.4 27.9 25.2 25.0 18.9 15.9
S23
^{13}C NMR, Benzene-d_6
101 MHz
210 200 190 180 170 160 150 140 130 120 110 100 90 80 70 60 50 40 30 20 10 0 -10
f1 (ppm)

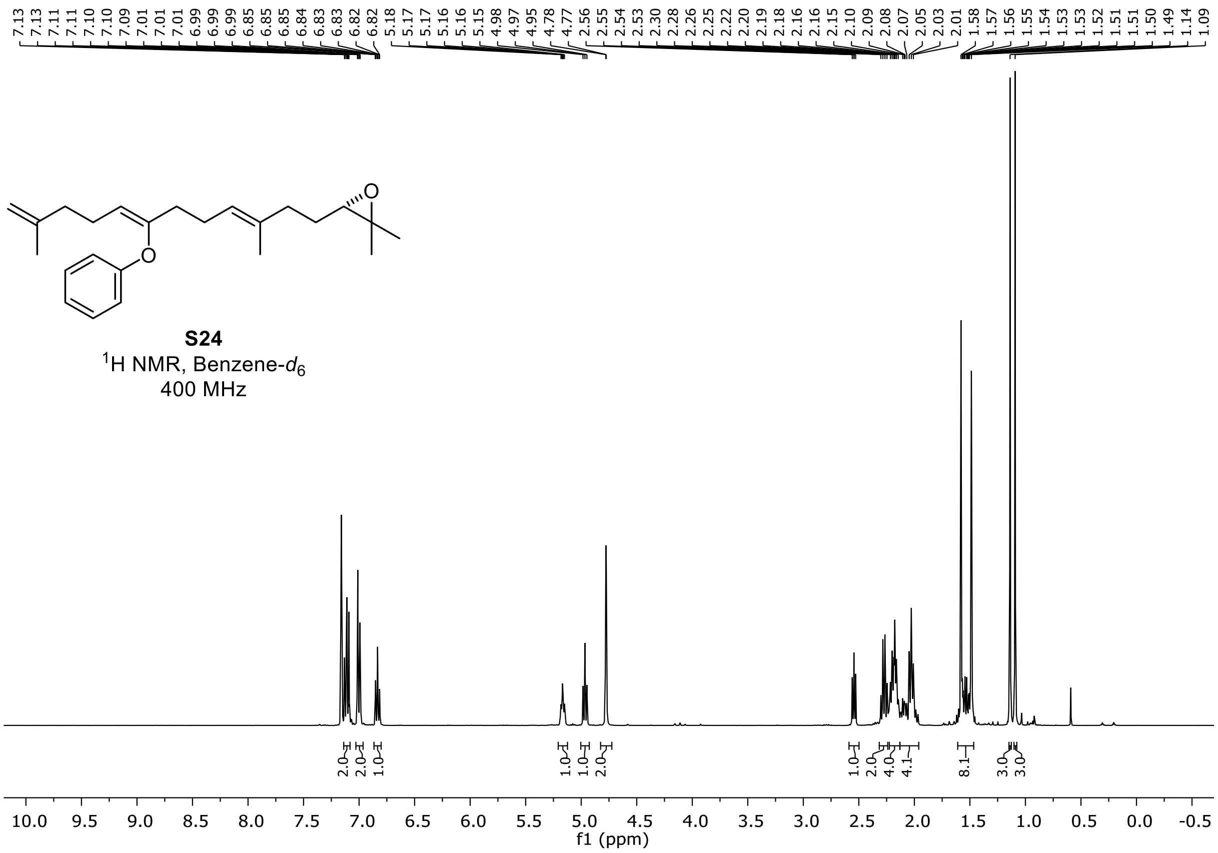

S24
^{1}H NMR, Benzene-d_6
400 MHz

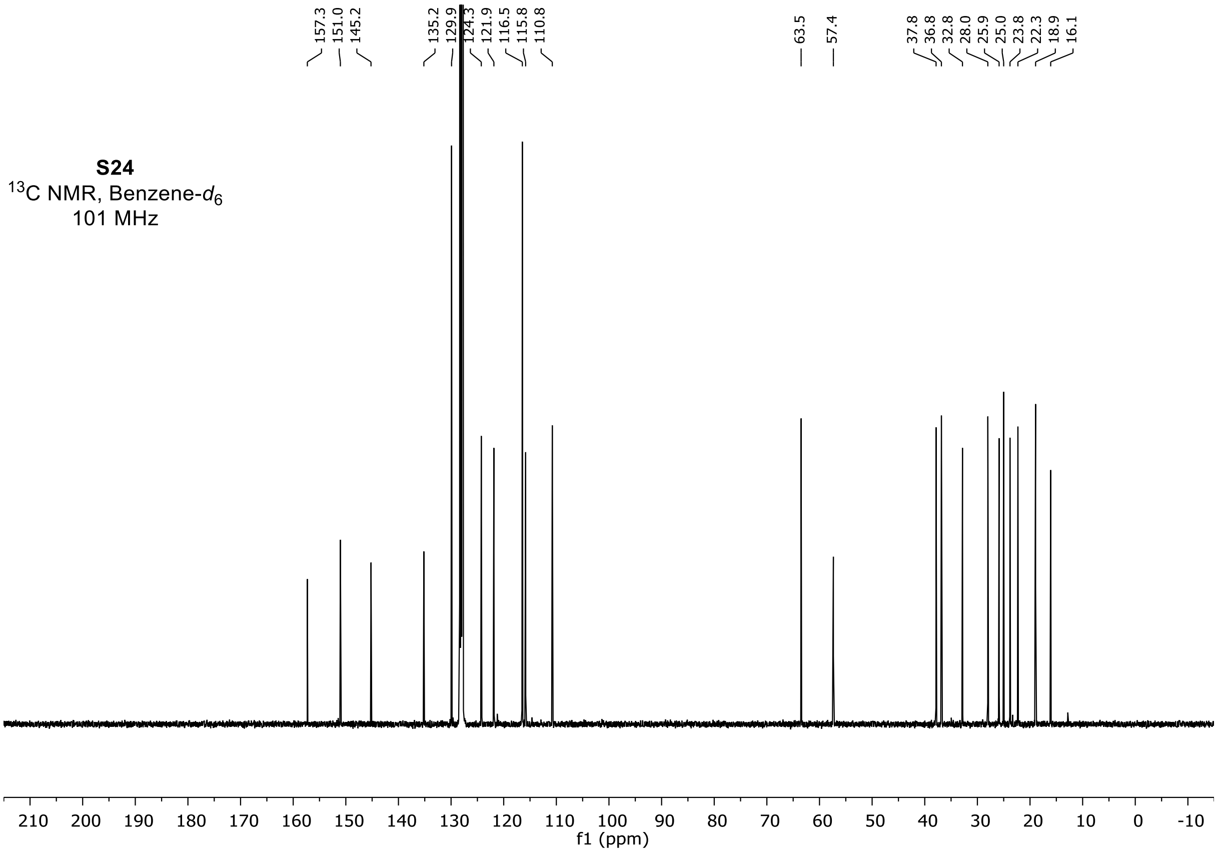

S24
^{13}C NMR, Benzene-d_6
101 MHz

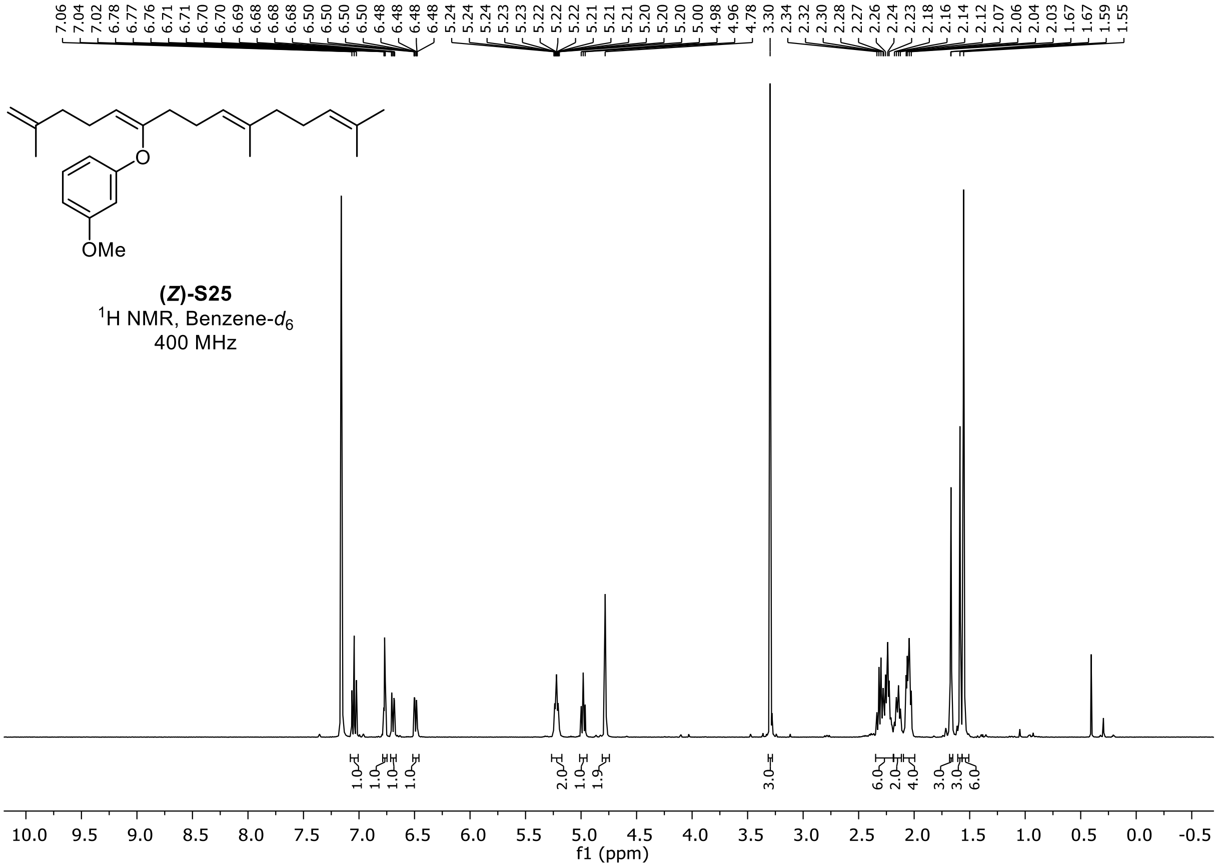
OMe
(Z)-S25
1H NMR, Benzene-d6
400 MHz
f1 (ppm)

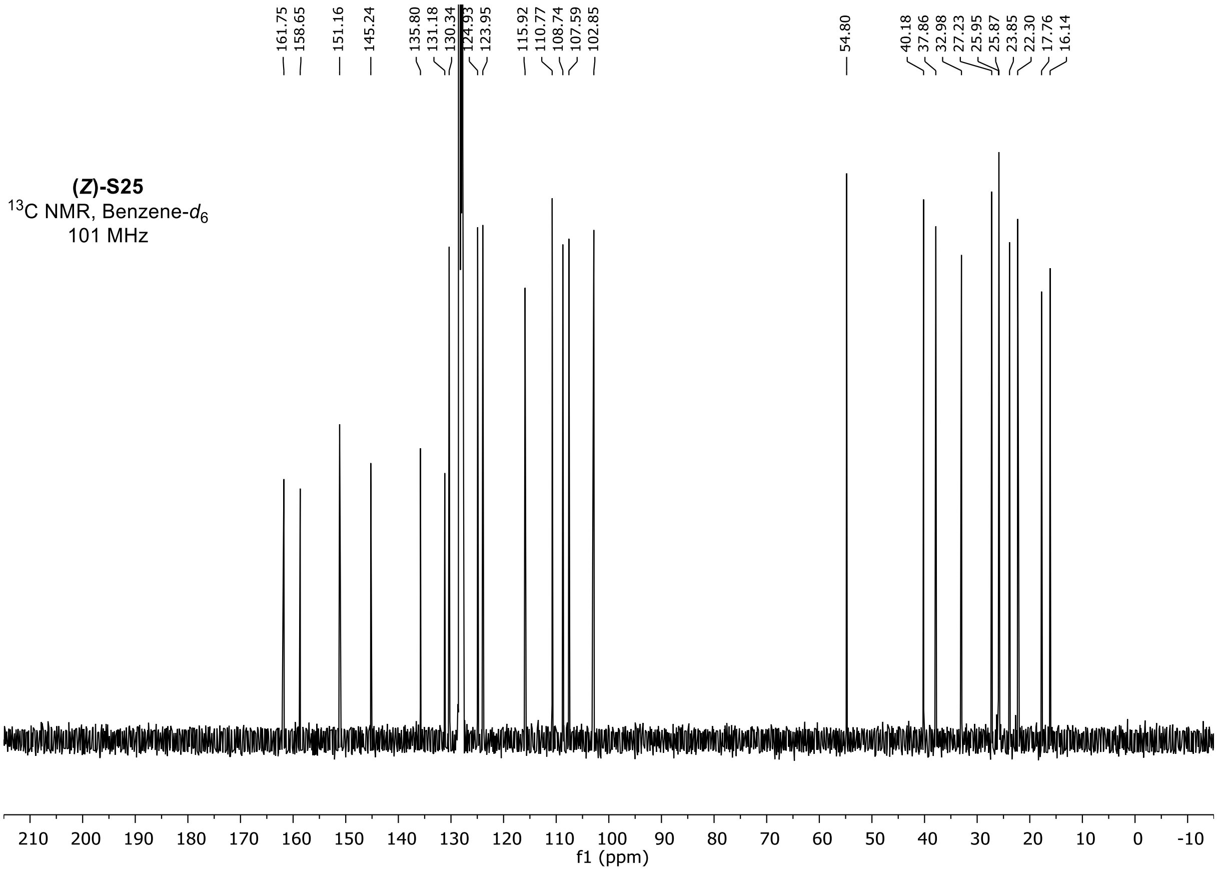
(Z)-S25
13C NMR, Benzene-d6
101 MHz
f1 (ppm)

5 X–Ray data

5.1 Minor regioisomer **8b**

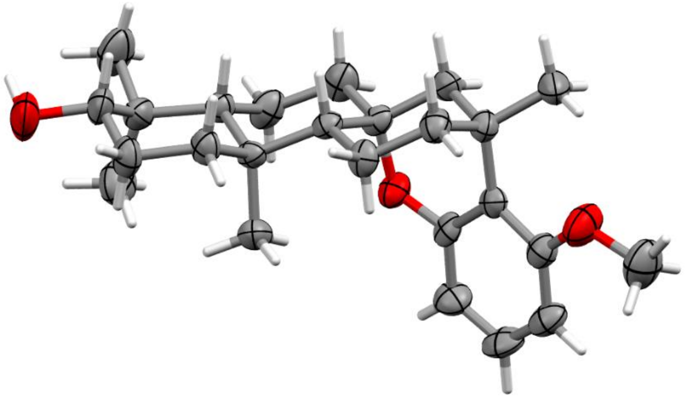

Identification code	compound_8b	
Empirical formula	$C_{25}H_{36}O_3$	
Formula weight	384.54	
Temperature	297(2) K	
Wavelength	0.71073 Å	
Crystal system	Orthorhombic	
Space group	$P2_12_12_1$ (no. 19)	
Unit cell dimensions	a = 7.1860(5) Å	α= 90°.
	b = 13.4417(10) Å	β= 90 °.
	c = 21.8077(17) Å	γ = 90°.
Volume	2106.5(3) $Å^3$	
Z	4	
Density (calculated)	1.213 Mg/m^3	
Absorption coefficient	0.077 mm^{-1}	
F(000)	840	
Crystal size	0.180 x 0.060 x 0.030 mm^3	
Theta range for data collection	2.405 to 21.521°.	
Index ranges	-7<=h<=7, -13<=k<=13, -22<=l<=22	
Reflections collected	12758	
Independent reflections	2431 [R(int) = 0.0685]	
Completeness to theta = 21.521°	99.8 %	
Absorption correction	Semi-empirical from equivalents	
Max. and min. transmission	0.992 and 0.931	
Refinement method	Full-matrix least-squares on F^2	
Data / restraints / parameters	2431 / 1 / 258	
Goodness-of-fit on F^2	1.050	
Final R indices [I>2sigma(I)]	R1 = 0.0410, wR2 = 0.0871	
R indices (all data)	R1 = 0.0576, wR2 = 0.0931	
Absolute structure parameter	0.5(10)	
Extinction coefficient	0.017(2)	
Largest diff. peak and hole	0.132 and -0.130 e.$Å^{-3}$	

5.2 Major regioisomer **8a**

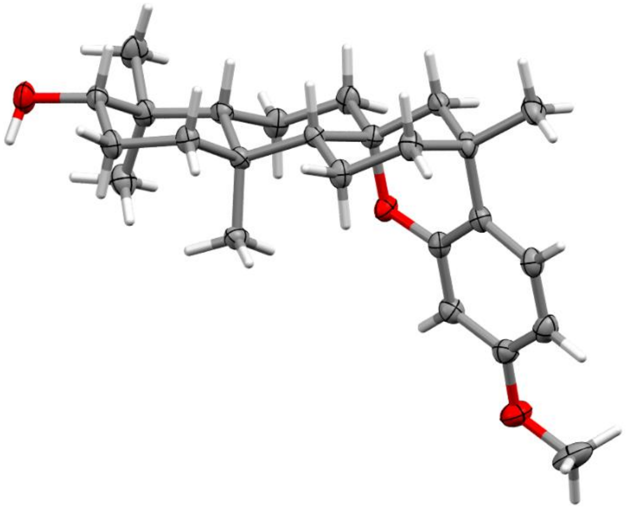

Identification code	compound_8a
Empirical formula	$C_{25}H_{36}O_3$
Formula weight	384.54
Temperature	183(2) K
Wavelength	0.71073 Å
Crystal system	Monoclinic
Space group	$P2_1$ (no. 4)
Unit cell dimensions	a = 11.1048(12) Å α= 90°.<br>b = 7.3792(7) Å β= 97.516(3) °.<br>c = 13.0067(14) Å γ = 90°.
Volume	1056.67(19) Å^3
Z	2
Density (calculated)	1.209 Mg/m^3
Absorption coefficient	0.077 mm^{-1}
F(000)	420
Crystal size	0.300 x 0.200 x 0.200 mm^3
Theta range for data collection	2.270 to 26.000°.
Index ranges	-13<=h<=13, -9<=k<=9, -15<=l<=16
Reflections collected	18571
Independent reflections	4144 [R(int) = 0.0201]
Completeness to theta = 21.521°	99.7 %
Absorption correction	Semi-empirical from equivalents
Max. and min. transmission	0.959 and 0.937
Refinement method	Full-matrix least-squares on F^2
Data / restraints / parameters	4144 / 2 / 258
Goodness-of-fit on F^2	1.012
Final R indices [I>2sigma(I)]	R1 = 0.0296, wR2 = 0.0794
R indices (all data)	R1 = 0.0301, wR2 = 0.0800
Absolute structure parameter	0.15(17)
Extinction coefficient	0.070(9)
Largest diff. peak and hole	0.212 and -0.156 e.Å^{-3}

5.3 Undesired diastereomer **19**

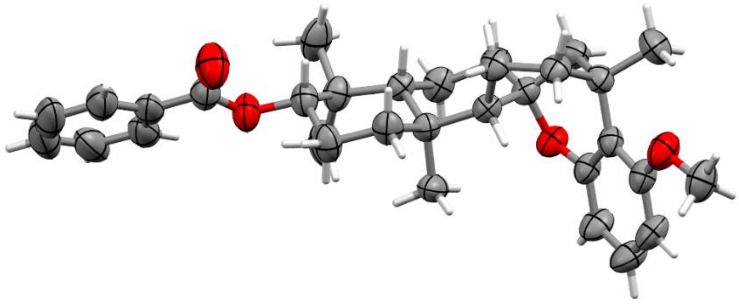

Identification code	compound_19
Empirical formula	$C_{32}H_{40}O_4$
Formula weight	488.64
Temperature	296(2) K
Wavelength	0.71073 Å
Crystal system	Orthorhombic
Space group	$P2_12_12_1$ (no. 19)
Unit cell dimensions	a = 7.3804(4) Å α= 90°.<br>b = 13.3089(8) Å β= 90 °.<br>c = 27.5773(16) Å γ = 90°.
Volume	2708.8(3) $Å^3$
Z	4
Density (calculated)	1.198 Mg/m^3
Absorption coefficient	0.077 mm^{-1}
F(000)	1056
Crystal size	0.180 x 0.180 x 0.120 mm^3
Theta range for data collection	2.693 to 24.998°.
Index ranges	-8<=h<=8, -15<=k<=15, -32<=l<=32
Reflections collected	29271
Independent reflections	4756 [R(int) = 0.0325]
Completeness to theta = 21.521°	99.7 %
Absorption correction	Semi-empirical from equivalents
Max. and min. transmission	0.971 and 0.934
Refinement method	Full-matrix least-squares on F^2
Data / restraints / parameters	4756 / 0 / 327
Goodness-of-fit on F^2	1.077
Final R indices [I>2sigma(I)]	R1 = 0.0477, wR2 = 0.1226
R indices (all data)	R1 = 0.0565, wR2 = 0.1271
Absolute structure parameter	-0.4(3)
Extinction coefficient	0.0132(19)
Largest diff. peak and hole	0.151 and -0.141 e.$Å^{-3}$

5.4 Tetraol **25**

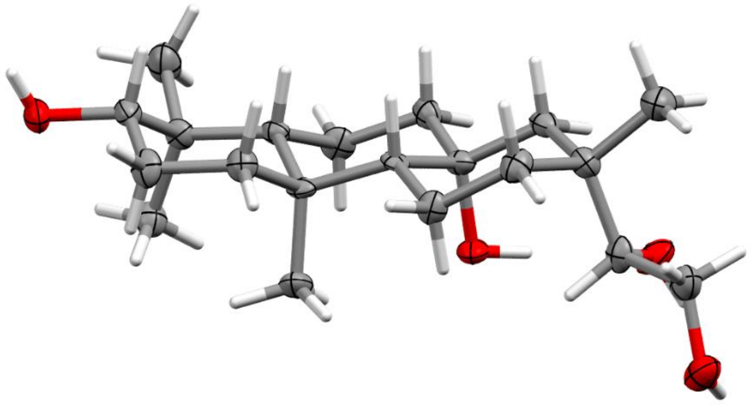

Identification code	compound_25
Empirical formula	$C_{20}H_{36}O_4$
Formula weight	340.49
Temperature	183(2) K
Wavelength	0.71073 Å
Crystal system	Orthorhombic
Space group	$P2_12_12_1$ (no. 19)
Unit cell dimensions	a = 7.415(2) Å α= 90°.<br>b = 11.049(3) Å β= 90 °.<br>c = 22.726(7) Å γ= 90°.
Volume	1861.8(10) Å^3
Z	4
Density (calculated)	1.215 Mg/m^3
Absorption coefficient	0.082 mm^{-1}
F(000)	752
Crystal size	0.120 x 0.060 x 0.020 mm^3
Theta range for data collection	2.571 to 20.497°.
Index ranges	-7<=h<=7, -10<=k<=10, -22<=l<=22
Reflections collected	21214
Independent reflections	1860 [R(int) = 0.1168]
Completeness to theta = 21.521°	99.8 %
Absorption correction	Semi-empirical from equivalents
Max. and min. transmission	0.956 and 0.901
Refinement method	Full-matrix least-squares on F^2
Data / restraints / parameters	1860 / 4 / 237
Goodness-of-fit on F^2	1.086
Final R indices [I>2sigma(I)]	R1 = 0.0395, wR2 = 0.0731
R indices (all data)	R1 = 0.0553, wR2 = 0.0780
Absolute structure parameter	0.4(10)
Extinction coefficient	0.0058(12)
Largest diff. peak and hole	0.178 and -0.128 e.Å^{-3}

6 References

[1] G. M. Sheldrick, *Acta Crystallogr. Sect. Found. Adv.* **2015**, *71*, 3–8.
[2] G. M. Sheldrick, *Acta Crystallogr. Sect. C Struct. Chem.* **2015**, *71*, 3–8.
[3] E. J. Corey, H. A. Kirst, *Tetrahedron Lett.* **1968**, *9*, 5041–5043.
[4] T. Nishikawa, S. Shibuya, S. Hosokawa, M. Isobe, *Synlett* **1994**, *1994*, 485–486.
[5] K. Speck, R. Wildermuth, T. Magauer, *Angew. Chem. Int. Ed.* **2016**, *55*, 14131–14135.
[6] E. J. Corey, M. C. Noe, S. Lin, *Tetrahedron Lett.* **1995**, *36*, 8741–8744.
[7] T. R. Hoye, C. S. Jeffrey, F. Shao, *Nat. Protoc.* **2007**, *2*, 2451–2458.
[8] K. Iwasaki, M. Nakatani, M. Inoue, T. Katoh, *Tetrahedron* **2003**, *59*, 8763–8773.
